# Synthesis of triarylmethanes by silyl radical-mediated cross-coupling of aryl fluorides and arylmethanes†

**DOI:** 10.1039/d3sc00154g

**Published:** 2023-03-21

**Authors:** Jun Zhou, Zhengyu Zhao, Bingyao Jiang, Katsuhiro Yamamoto, Yuji Sumii, Norio Shibata

**Affiliations:** a Department of Nanopharmaceutical Sciences, Nagoya Institute of Technology Gokiso, Showa-ku Nagoya 466-8555 Japan nozshiba@nitech.ac.jp; b Department of Life Science and Applied Chemistry, Nagoya Institute of Technology Gokiso, Showa-ku Nagoya 466-8555 Japan

## Abstract

Although the cross-couplings of aryl halides with diarylmethanes are mostly achieved by transition-metal catalysis, aryl fluorides are rarely used as coupling partners owing to the high inertness of C–F bonds. Herein, we describe the efficient silylboronate-mediated cross-coupling reaction of aryl fluorides with arylalkanes under transition-metal-free, room-temperature conditions. The combination of silylboronate and KO^*t*^Bu is critical for driving a radical process *via* the cleavage of C–F and C–H bonds in two appropriate coupling precursors, resulting in a cross-coupling product. This practical cross-coupling protocol is applicable to a wide variety of aryl fluorides with a C(sp^2^)–F bond. This method can be extended to other coupling partners with a C(sp^3^)–H bond, including diarylmethanes, diarylethanes, and monoarylalkanes. Many di- and triarylalkanes with tertiary or quaternary carbon centers can be obtained easily in moderate to high yields. We believe that the developed silylboronate-mediated cross-coupling method is a valuable contribution to C–F and C–H activation chemistry.

## Abstract 2

Silylboronate-mediated radical cross-coupling of aryl fluorides with arylalkanes is disclosed for the efficient defluoroalkylation enabling a mild construction of a new C–C bond by selectively coupling a C(sp^2^)–F and C(sp^3^)–H bond.

## Graphical abstract

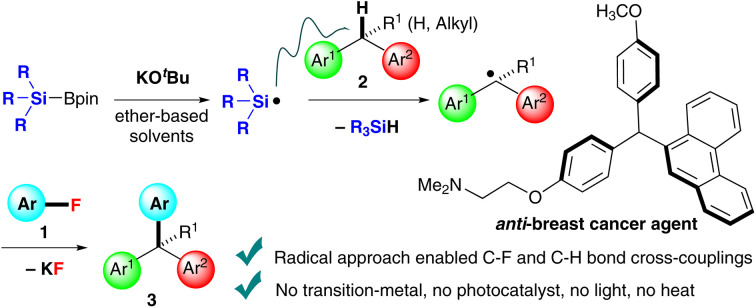

## Introduction

**Fig. 1:**
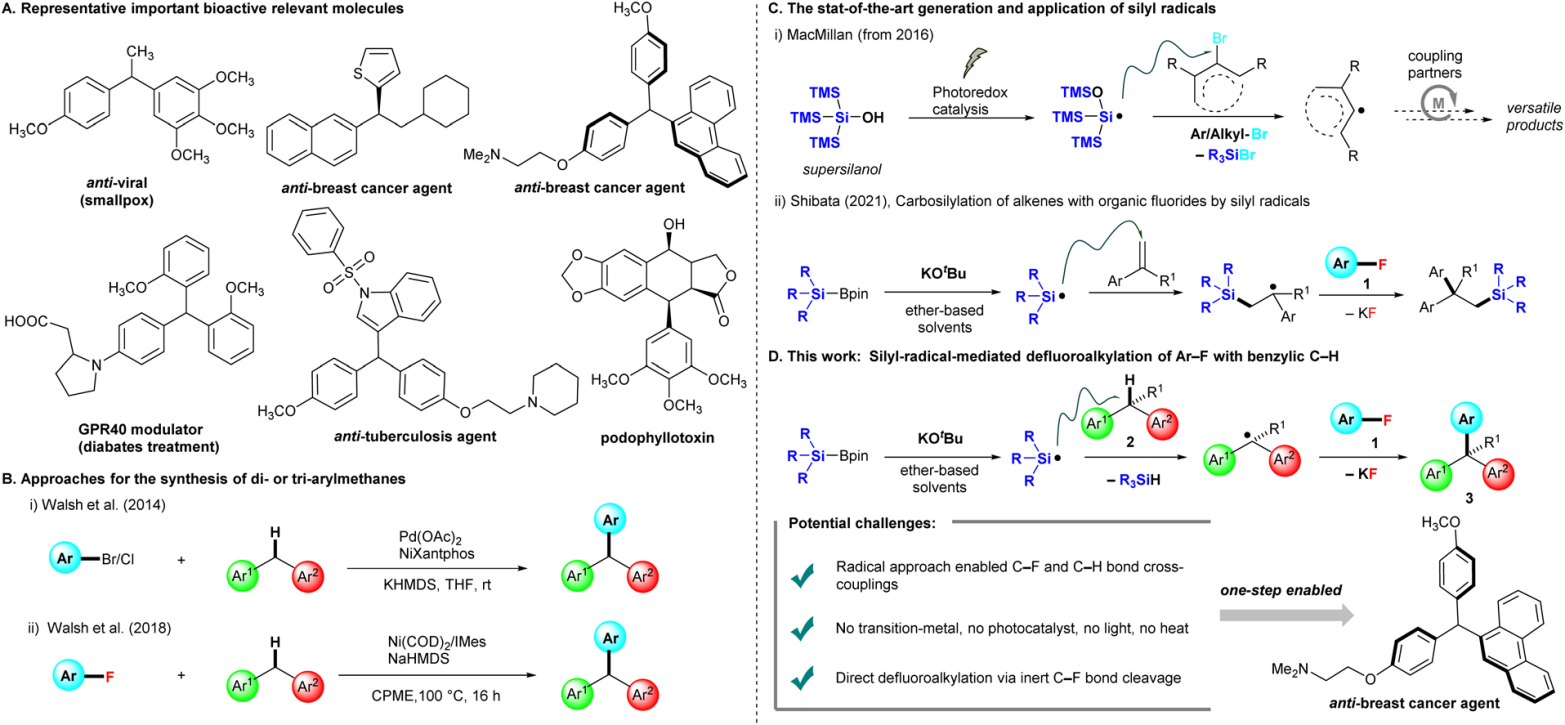
Representative bioactive relevant molecules and approaches for the synthesis of triarylmethanes and variants. (A) Bioactive relevant molecules. (B) Cross-couplings and related reactions of aryl or alkyl halides with diarylalkanes. (C) Generation and applications of silyl radicals. (D) This work: silyl-radical-mediated cross-coupling of benzylic C–H with Ar–F.

Benzylic motifs with a C(sp^3^)–H bond (ArCHR_2_) are present in many bioactive compounds,^1^ and ∼25% of the 200 top-selling pharmaceuticals contain these motifs.^2^ Therefore, the functionalization of such benzylic C–H bonds to new C–C,^3^ C–N,^4^ and C–O^5^ bonds is the logical next step for the further modification of drug candidates. In particular, triarylmethanes (ArCHAr_2_) and diarylalkanes (Ar_2_CHR) are some of the most attractive frameworks targeted for benzylic C–H functionalization, as they widely exist in pharmaceuticals,^6^ functional materials,^7^ and sensing systems.^8^ Several representative triarylmethanes and diarylalkane compounds have been utilized as pharmacological agents for treating viral infection, bacterial infection, breast cancer, and diabetes (Fig. 1A). Friedel–Crafts arylations of diarylmethanols are traditionally used for producing these frameworks, but this chemistry limits nucleophilic and electron-rich arenes and occasionally forms regioisomers.^9^ Walsh *et al.* reported the first Pd-catalyzed cross-couplings of aryl halides (Ar–Br and Ar–Cl) with diarylmethanes, providing triarylmethanes at room temperature.^10^ The conditions of Pd(OAc)_2_, NiXantphos, and KHMDS were able to effectively circumvent the limitations of traditional cross-coupling methods, which require high reaction temperatures (Fig. 1B(i)). Subsequently, several methods for preparing triarylmethanes or diarylalkanes under mild conditions have been reported, most of which involve transition-metal catalysis.^11^ These protocols require aryl halides (Ar–X, X = I, Br, Cl), but aryl fluorides (Ar–F) are not appropriate as cross-coupling precursors because the C–F bond is rather inert and possesses the highest bond dissociation energy in the series. The chemical transformation of fluorinated moieties into other functional groups is a considerable challenge,^12^ In 2018, Walsh *et al.* extended their cross-coupling protocol to aryl fluorides. They found suitable conditions [Ni(COD)_2_ (10 mol%) and 1,3-bis(2,4,6-trimethylphenyl)imidazole-2-ylidene (IMes, 20 mol%) in the presence of NaHMDS (3.0 equiv.) in cyclopentyl methyl ether (CPME)] for producing the desired products, but high temperatures and long reaction times (16 h) were required (Fig. 1B(ii)).^13^

In recent years, MacMillan *et al.* reported that photocatalytically generated silicon-centered radicals from “supersilanol” could effectively abstract a bromine atom from suitable aryl/alkyl bromides to afford the corresponding aryl/alkyl radicals, which are subsequently captured by transition metal catalysis, especially nickel or copper catalysis, to undergo a series of catalytic circles with coupling partners to afford a library of coupling products (Fig. 1C(i)). The state-of-the-art combination of the “supersilyl” group, photoredox catalysis and the transition metal catalysis system has emerged as a powerful strategy in organic chemistry.^14^ In 2021, our group discovered that silicon-centered radicals are effectively generated by mixed silylboronates (R_3_SiBpin) and potassium *tert*-butoxide (KO^*t*^Bu) without either a photoredox system or high temperature, and then enabled a catalyst-free carbosilylation of alkenes with aryl fluorides 1*via* the activation of an inert C–F bond at room temperature (Fig. 1C(ii)).^15^

Inspired by the studies mentioned above^14^ and our studies on C–F bond activation,^15,16^ we herein propose a silylboronate-mediated radical cross-coupling reaction of aryl fluorides 1 with arylalkanes 2*via* the cleavage of both C–F and C–H bonds, which produces triarylmethanes 3 (Fig. 1D). Notably, our approach allows cross-coupling between an aryl C(sp^2^)–F bond in 1 and a benzylic C(sp^3^)–H bond in 2. This coupling reaction proceeds smoothly at room temperature without transition-metal catalysis. Supersilyl, photoredox catalysis, or irradiation with LEDs is not required. The substrate scope of arylalkanes 2 is broad, including diarylmethanes, diarylethanes, and monoarylalkanes. Dihydroanthracene and 9*H*-xanthene are also good coupling partners with aryl fluorides 1, furnishing the desired triarylmethane products 3 in high yields. As many aryl fluorides and arylalkanes are readily available, including complex pharmaceuticals, we expect the radical coupling of aryl fluorides with arylalkanes to be a valuable method for the straightforward preparation of various materials, such as drug candidates and specialty materials.

## Results and discussion

### Silylboronate-mediated cross-coupling reaction of organic fluorides and arylalkanes

The reaction of 4-fluorobiphenyl (1a) with diphenylmethane (2a) was initially investigated as a model reaction (Table 1). We attempted the reaction in the presence of Et_3_SiBpin (2.0 equiv.), Ni(COD)_2_ (10 mol%), and KO^*t*^Bu (3.0 equiv.) in THF at room temperature; these conditions were also used in our previous defluorosilylation of aryl fluorides.^16^ The reaction proceeded as expected, producing 4-benzhydrylbiphenyl (3aa) in 37% yield accompanied by the defluorosilylated product, biphenyl-4-yltriethylsilane (4a) (entry 1). Without Ni(COD)_2_, the yield of 3aa improved to 47% (entry 2). This was followed by base screening (entries 3–6). Weaker bases were not suitable (entries 3 and 4), but strong bases (NaO^*t*^Bu and KHMDS) did not improve the yield (entries 5 and 6). The yield was slightly increased to 49% with the use of 4.0 equiv. of KO^*t*^Bu (entry 7). Significantly, the solvent was found to be crucial for obtaining a high yield of 3aa (entries 8–16), and the use of diglyme resulted in a high yield (95%, entry 16). Notably, diglyme effectively suppressed the formation of byproduct 4a (entry 16). The effects of diglyme can be explained by the fact that K^+^ would be encapsulated by diglyme, giving a more “naked” and stronger base.^17^ Control experiments revealed that no reaction occurred in the absence of KO^*t*^Bu or Et_3_SiBpin (entries 17 and 18). Finally, the reactions were repeated using 0.2 mmol and 4.0 mmol of 1a, respectively, under the same conditions as those for entry 16 to evaluate the scale-up of the coupling process, and product 3aa was successfully obtained in 96% (93% isolated yield, entry 19) and 85% isolated yield (entry 20). Further details of optimization of the conditions are shown in the ESI.†

**Table 1:** Optimization of the defluoronative cross-coupling conditions^a^

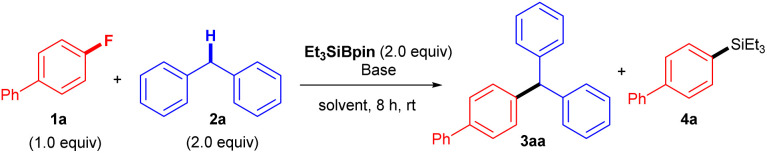				
Entry	Base (equiv.)	Solvent	3aa^b^ (%)	4a (±)
1^c^	KO^*t*^Bu (3.0)	THF	37	+
2	KO^*t*^Bu (3.0)	THF	47	+
3	K_2_CO_3_ (3.0)	THF	—	—
4	Cs_2_CO_3_ (3.0)	THF	—	—
5	NaO^*t*^Bu (3.0)	THF	28	+
6	KHMDS (3.0)	THF	30	+
7	KO^*t*^Bu (4.0)	THF	49	+
8	KO^*t*^Bu (4.0)	*c*-hexane/THF (8/1, v/v)	34	+
9	KO^*t*^Bu (4.0)	*c*-hexane	9	+
10	KO^*t*^Bu (4.0)	Toluene	11	+
11	KO^*t*^Bu (4.0)	Dioxane	Trace	+
12	KO^*t*^Bu (4.0)	DME	36	+
13	KO^*t*^Bu (4.0)	CPME	18	+
14	KO^*t*^Bu (4.0)	MTBE	12	+
15	KO^*t*^Bu (4.0)	DTBE	Trace	+
16	KO^*t*^Bu (4.0)	Diglyme	95	—
17	—	Diglyme	0	—
18^d^	KO^*t*^Bu (4.0)	Diglyme	0	—
19^e^	KO^*t*^Bu (4.0)	Diglyme	96 (93)	—
20^f^	KO^*t*^Bu (4.0)	Diglyme	(85)	—

a Reactions were attempted under indicated reagents and conditions: 1a (17.2 mg, 0.1 mmol), 2a, KO^*t*^Bu and solvent (1.0 mL) reacted at room temperature for 8 h.

b Determined by ^19^F NMR and ^1^H NMR spectroscopy using 3-fluoropyridine as an internal standard. The number in parentheses refers to the isolated yield.

c 10 mol% Ni(COD)_2_ was used.

d Performed without Et_3_SiBpin.

e 0.2 mmol scale was performed.

f 4.0 mmol scale was performed.

### Silylboronate-mediated cross-coupling reaction of organic fluorides and arylalkanes

**Fig. 2:**
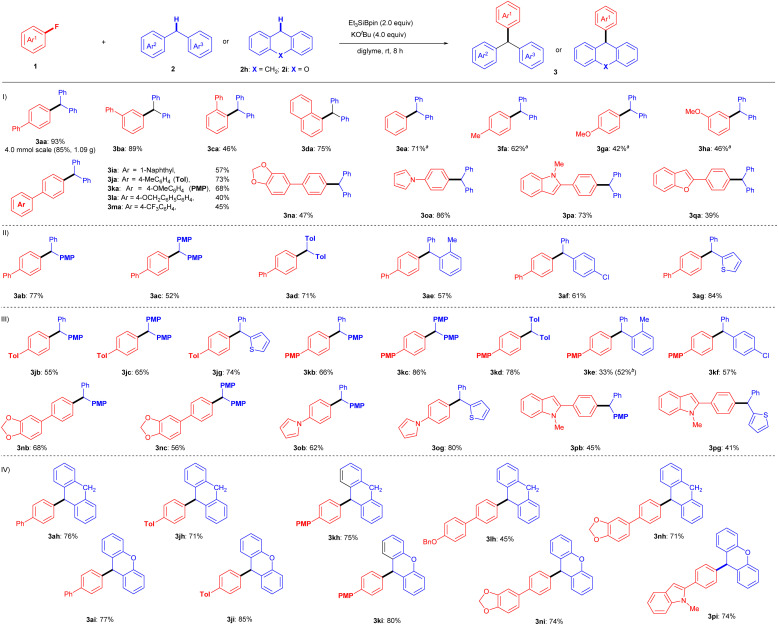
Substrate scope of 1 and 2. (I) Scope of aryl fluorides 1; (II) scope of diarylmethanes 2; (III) scope regards to the combination of 1 and 2; (IV) scope within 1 and DHA or 9*H*-xanthene. Unless otherwise noted, reactions were conducted using 1 (0.2 mmol), 2a (2.0 equiv.), Et_3_SiBpin (2.0 equiv.), KO^*t*^Bu (4.0 equiv.), and diglyme (2.0 mL) at room temperature for 8 h, with isolated yields shown. ^*a*^ Reaction performed using 0.4 mmol of 1. ^*b*^ Reaction performed using Et_3_SiBpin (3.0 equiv.) and KO^*t*^Bu (6.0 equiv.).

With the optimal reaction conditions determined (entry 19, Table 1), the substrate scope of this silylboronate-mediated defluorinative cross-coupling reaction was further investigated (Fig. 2). A range of substituted aryl fluorides 1 were investigated with 2a to assess their generality (Fig. 2(I)). As shown, a wide range of fluoroarenes, including π-extended systems 1a–d, fluorobenzene 1e, and methyl- and methoxy-substituted fluorobenzenes 1f–h, were efficiently coupled with 2a to afford corresponding triarylmethanes 3aa–ha in yields of up to 93%. The yields of 3 were slightly lowered in the coupling reactions of aryl fluorides 1 that were affected by steric hindrance (3ca: 46%) and electron-rich substituents (3ga: 42%; 3ha: 46%). A series of *p*-substituted 4′-fluorobiphenyls 1i–m and dioxole 1n also produced the corresponding triarylmethanes in moderate to good yields under standard conditions: 3ia: 57%, 3ja: 73%, 3ka: 68%, 3la: 40%, 3ma: 45%, and 3na: 47%. These results indicate that the ether (OMe), the benzylic position (OBn), and the C(sp^3^)–F bond of CF_3_ are tolerated in this transformation. In addition, aryl fluorides 1o–q with attached heterocycles were evaluated, as C–H activation might be competitively induced by the heterocyclic moiety. Pyrrole- or indole-containing aryl fluorides 1o and 1p successfully reacted with 2a to furnish 1*H*-pyrrole derivative 3oa (86%) and *N*-methyl-1*H*-indole derivative 3pa (73%), respectively, without any C–H activation product detected. In addition, benzofuran-bearing fluoroarene 1q participated in this cross-coupling reaction, although the yield of coupling product 3qa was only 39%.

We next examined the substrate scope of diarylmethanes 2 in the cross-couplings with 4-fluorobiphenyl (1a) (Fig. 2(II)). Electron-rich phenyl-(4-methoxyphenyl)methane (2b), bis(4-methoxyphenyl)methane (2c), and bis(tolyl)methane (2d) smoothly reacted with 1a under the standard conditions to furnish desired triarylmethanes 3ab (77%), 3ac (52%), and 3ad (71%), respectively. Even sterically hindered *ortho*-methyl-diphenylmethane (2e) produced coupling product 3ae in 57% yield. Notably, Cl-substituted diphenylmethane 3f was tolerated under the same conditions, selectively providing defluorinative coupling product 3af (61%). Furthermore, 2-benzylthiophene (2g) was successfully coupled with 1a under identical reaction conditions to yield 3ag (84%).

We further attempted the coupling reaction for a range of substituted fluoroarenes (1j–p) and substituted diarylmethanes (2b–g) to widen the generality (Fig. 2(III)). Fluoro-biphenyls with Me (1j) or OMe (1k) substituents reacted with diarylmethanes 2b–f under the optimal conditions to furnish the desired triarylmethanes in moderate to good yields (3jb: 55%; 3jc: 65%; 3jg: 74%; 3kb: 66%; 3kc: 86%; 3kd: 78%; 3ke: 33%; 3kf: 57%). The low yield of 3ke can be explained by the steric hindrance of the *o*-Me group in 2e, and the result was improved to 52% with the use of excess Et_3_SiBpin (3.0 equiv.) and KO^*t*^Bu (6.0 equiv.). In addition, dioxole-bearing fluoroarene 1n reacted well with 4-methoxydiphenylmethane (2b) and dianisylmethane (2c) to give triarylmethanes 3nb (68%) and 3nc (56%), respectively. Furthermore, despite possessing several reactive C(sp^2^)–H bonds in their heterocyclic skeletons, the cross-coupling reactions of pyrrole aryl fluoride 1o and indole aryl fluoride 1p proceeded well to furnish heteroaryl-containing products 3ob (62%), 3og (80%), 3pb (45%), and 3pg (41%) *via* C–F bond cleavage, without any of the anticipated C–H cross-coupling reactions in the heteroaromatic moiety.

The interesting aspect of this protocol is the usage of dihydroanthracene (DHA, 2h) as a cross-coupling partner with fluoroarenes (Fig. 2(IV)). DHA is known to act as a radical inhibitor.^18^ Although this transformation should involve a radical process (see the discussion below), the cross-coupling of aryl fluorides 1 with 2h proceeded very well under the standard conditions, giving the desired cross-coupling products in good yields (3ah: 76%; 3jh: 71%; 3kh: 75%; 3lh: 45%; 3nh: 71%). Similarly, 9*H*-xanthene (2i) also produced good results with aryl fluorides 1 under the same conditions (3ai: 77%; 3ji: 85%; 3ki: 80%; 3ni: 74%; 3pi: 74%).

**Fig. 3:**
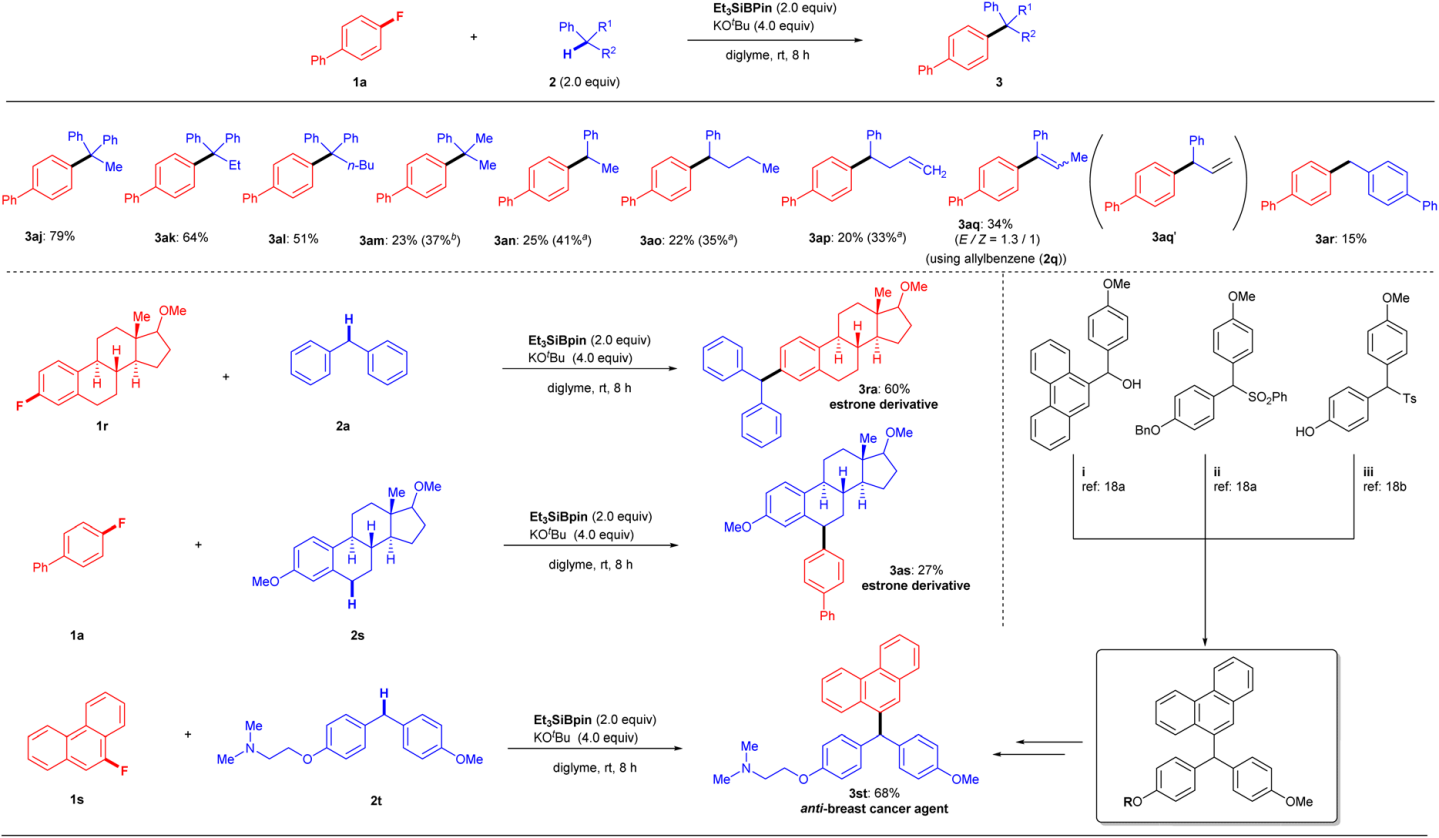
Further scope and limitations of arylalkanes 2. Unless otherwise noted, reactions were conducted with 1 (0.2 mmol), 2 (2.0 equiv.), Et_3_SiBpin (2.0 equiv.), KO^*t*^Bu (4.0 equiv.), and diglyme (2.0 mL) at room temperature for 8 h, with isolated yields shown. ^*a*^ Reaction performed using Et_3_SiBpin (3.0 equiv.) and KO^*t*^Bu (6.0 equiv.).

We next examined another potential limitation of this methodology, focusing on arylalkanes 2 (Fig. 3, top). The cross-coupling of 1a with 1,1-diphenylalkanes 2j–l under the optimal conditions produced products 3aj (79%), 3ak (64%), and 3al (51%), which possessed a quaternary carbon center, in good yields. In comparison, cumene (2m) produced product 3am in only 23% yield. Other arylalkanes with a single aromatic group (2n–p) afforded corresponding cross-coupling products 3an–ap in 20–25% yields (3an: 25%; 3ao: 22%; 3ap: 20%). The low yields of 3 were slightly improved by the use of excess reagents (3am: 37%; 3an: 41%; 3ao: 35%; 3ap: 33%). These results indicated that successful conversion is highly dependent on the stability of the reactive benzylic species. When we attempted the reaction of allylbenzene (2q), we obtained 3aq (*Z*/*E* = 1 : 1.3) in 34% yield instead of the expected coupling product 3aq′. While there are several possibilities for its formation, one is the isomerization of 3aq′ to 3aq under the optimal conditions. Indeed, we obtained 3aq (*Z*/*E* = 1 : 1.3) by treating 3aq′ (independently prepared) under the same KO^*t*^Bu/Et_3_SiBpin conditions. Interestingly, the isomerization of 3aq′ under the base (KO^*t*^Bu) gave 3aq with a different ratio (*Z*/*E* = 1 : 1) (see details in the ESI†). Next, the reaction of 1a with toluene was attempted to confirm this hypothesis. As expected, no desired coupling product was observed. However, when *p*-phenyl-substituted toluene (2r) was used, the desired coupling product 3ar was obtained in 15% yield, supporting the formation of a benzylic radical species (see the Discussion section). Although various substrates 1, 2, and 3 with functional groups and heterocycles have been used successfully (Fig. 2 and 3), some functional limitations were experienced, such as with carbonyls, amines, and free H (OH, NH_2_, *etc.*). These limitations are listed in the ESI.†

### Application of the silylboronate-mediated defluorinative coupling reaction

To highlight the synthetic applications of this silylboronate-mediated defluorinative coupling reaction, we examined the functionalization of several drug derivatives with a fluoroarene moiety or benzylic C–H moiety (Fig. 3, bottom). Estrone-derived fluoroarene 1r successfully underwent a coupling reaction with diphenylmethane 2a to afford desired estrone derivative 3ra in 60% yield. Moreover, the three benzylic C–H bonds containing motif 2s, derived from estrone, were also successfully functionalized at the secondary C–H site using this transformation with 1a to give 3as in 27% yield. Another noteworthy application is that this modular approach also enables the rapid synthesis of anti-breast-cancer agent 3st in one single step rather than several steps.^19^ By simply employing 9-fluorophenanthrene 1s and 2-(4-(4-methoxybenzyl)phenoxy)-*N*,*N*-dimethylethan-1-amine 2t under standard reaction conditions, desired product 3st can be easily fashioned in 68% yield.

**Fig. 4:**
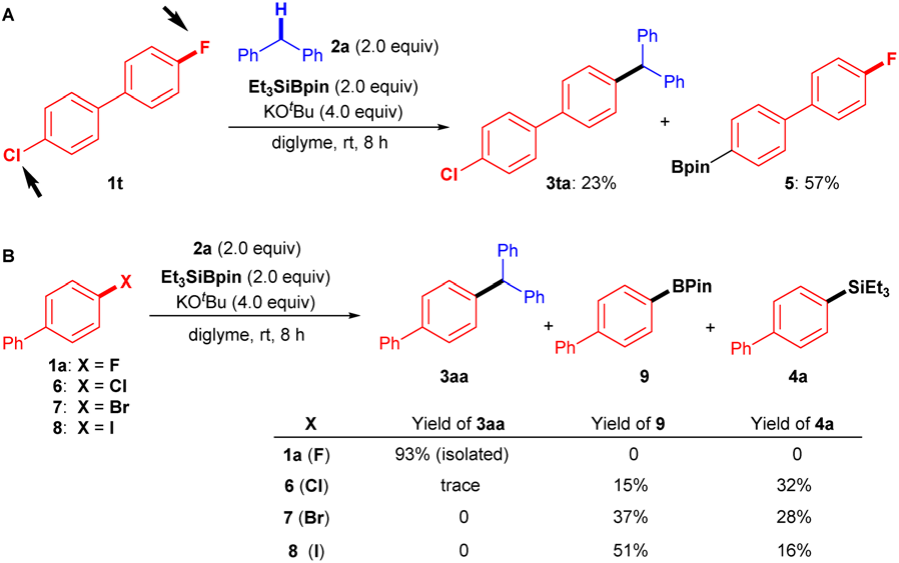
Chemoselectivity and parallel experiments. (A) Chemoselectivity of Ar–F over Ar–Cl. (B) Coupling reactions of 2a with 1a (X = F), 6 (X = Cl), 7 (X = Br), and 8 (X = I).

It should be mentioned that the chemoselectivity of our coupling reaction is slightly poor, whereas it was difficult to efficiently transform 4-chloro-4′-fluoro-1,1′-biphenyl (1t) into desired product 3ta (23%). As a result, borylated product 5 (57%) was obtained preferably *via* C–Cl bond cleavage (Fig. 4A).

However, the advantage of using aryl fluorides as coupling partners over other aryl halides in the present transformation is evident, as shown in the parallel experiments (Fig. 4B). When we attempted the cross-coupling reactions of diphenylmethane (2a) with biphenyl chloride (6), biphenyl bromide (7), and biphenyl iodide (8), mixtures of borylated product 9 and silylated product 4a were detected, with only traces of desired cross-coupling product 3aa observed under the standard conditions.

### Mechanistic study

**Fig. 5:**
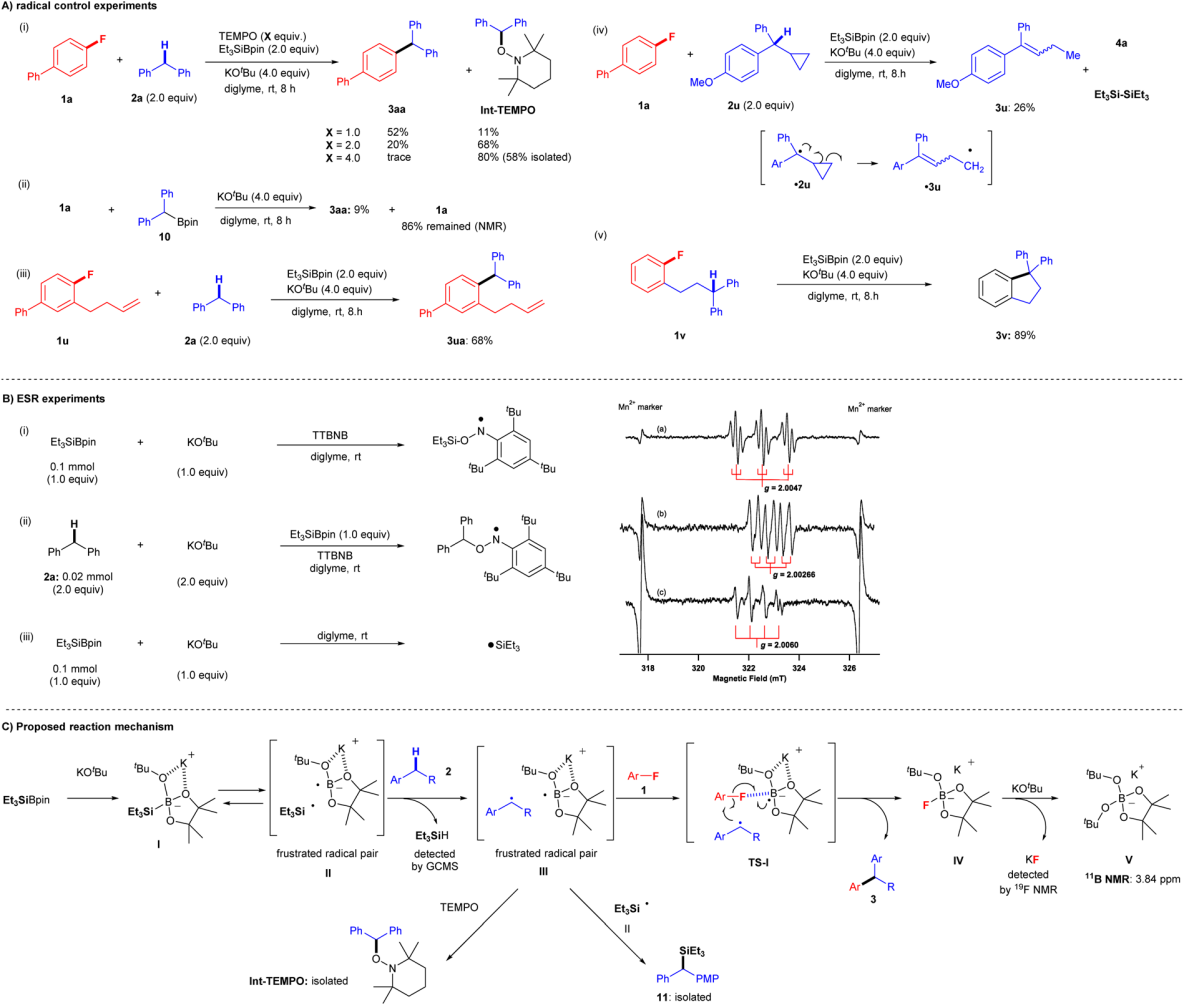
Control experiment study and the proposed reaction mechanism. (A) Radical control experiments. (i) Effect of TEMPO in the silylboronate-mediated coupling reaction. (ii) SNAr conditions using pinBCHPh_2_10 in the presence of KO^*t*^Bu. (iii) Radical cyclization experiments. (iv) Radical ring-opening experiment. (v) Radical cyclization experiment. (B) ESR experiments and chemical structure. (i) and (a) spin-adduct of TTBNB with triethyl silyl radical (anilino-type) and (ii) and (b) spin-adduct of TTBNB with diphenyl methyl radical (anilino-type). (iii) and (c) triethyl silyl radical. (C) Proposed reaction mechanism.

Several observations in the present study led us to believe that this transformation proceeds *via* a single-electron transfer (SET) radical process, but not *via* nucleophilic substitution pathways, such as traditional nucleophilic aromatic substitution (S_N_Ar) and S_N_2 where arylfluorides can act as electrophiles or benzyne precursors,^20–23^ because S_N_Ar and S_N_2 protocols require electron-deficient aryl fluorides under strongly basic conditions. We, therefore, conducted several further experiments to gain insight into the reaction mechanism (Fig. 5). We first examined the coupling reaction of aryl fluoride 1a and diphenylmethane 2a in the presence of (2,2,6,6-tetramethylpiperidin-1-yl)oxyl (TEMPO) (Fig. 5A(i)). Although coupling product 3aa was obtained in 93% yield under the standard conditions, the yields decreased considerably as the amount of TEMPO increased: 52% (1.0 equiv. of TEMPO), 20% (2.0 equiv. of TEMPO), and trace (4.0 equiv. of TEMPO). Moreover, increasing the quantity of TEMPO led to an increase in the yield of 1-(benzhydryloxy)-2,2,6,6-tetramethylpiperidine (Int-TEMPO) from 11% (1.0 equiv. of TEMPO) to 68% (2.0 equiv. of TEMPO) and 80% (4.0 equiv. of TEMPO). These results suggest that the cross-coupling reaction involves a radical species. Ohmiya and co-workers reported the cross-coupling of aryl fluorides by tertiary benzylic organoboronates with KO^*t*^Bu at a high temperature of 120 °C *via* S_N_Ar.^24*a*^ We therefore attempted the reaction of 1a with pinBCHPh_2_10 in the presence of KO^*t*^Bu in diglyme at room temperature (Fig. 5A(ii)). We detected only 9% of 3aa, leaving most of 1a. These results indicate the formation of carbanion from pinBCHPh_2_ and that the S_N_Ar process would not be included.^24*b*^

A standard radical clock experiment using 1-(but-3-en-1-yl)-2-fluorobenzene (1u) was performed with 2a under identical cross-coupling conditions (Fig. 5A(iii)). Significantly, corresponding cross-coupling products 3ua (no cyclization; 68% yield) were obtained. The result is suggestive of C–F bond cleavage proceeding *via* a cascade, concerted process in the final part of the reaction mechanism, without generating a free aryl radical. Moreover, two more radical clock experiments were conducted to give further insight into the reaction process. First, the reaction of 1a and cyclopropyl benzyl derivative 2u was attempted, but the reaction did not yield the corresponding cross-coupling product. Instead, the ring-opening product 3u was isolated (26% yield) presumably *via* a cyclopropyl benzyl radical ˙2u, and we also detected the associated by-products such as 4a and hexaethyldisilane ((Et_3_Si)_2_) (Fig. 5A(iv)). Additionally, an intramolecular cross-coupling reaction was achieved by treatment of diphenylpropyl-substituted fluorobenzene 1v under identical conditions, and cyclization product 3v was furnished in 89% yield (Fig. 5A(v)).

Finally, ESR experiments were carried out to confirm the generation of radicals under the optimized conditions (Fig. 5B). First, an ESR experiment was performed with the spin trap tri-*tert*-butylnitrosobenzene (TTBNB). The ESR spectrum (triple–triplet) for the reaction of Et_3_SiBpin and KO^*t*^Bu in diglyme at room temperature (Fig. 5B(i)(a)) corresponded to that of the spin adduct of the triethylsilyl radical (˙SiEt_3_) trapped by TTBNB. The hyperfine splitting (hfs) constant due to nitrogen (*A*_N_; spin quantum number *I* = 1) was 1.03 mT, and the small splitting constant due to the two hydrogens at the *meta* position of the TTBNB benzene ring (*A*_Hm_; *I* = 1/2) was 0.175 mT. The *g*-value of 2.0047 was assigned to an anilino-type radical (Fig. 5B(i)).^25^ The observed spectrum is the similar triple-triplet spectrum reported for SiEt_3_-TTBNB previously.^26,27^ We next investigated the reaction of diphenylmethane (2a), Et_3_SiBpin, and KO^*t*^Bu in diglyme at room temperature (Fig. 5B(ii)(b)). The ESR spectrum (double-triplet; sextet line) was assigned to a benzyl-type radical (˙CHPh_2_) trapped by TTBNB. The hfs constants *A*_N_ and *A*_Hα_ (due to an α-proton) were 0.62 and 0.34 mT, respectively. The splitting due to the *meta*-hydrogens was too small to be resolved (<0.06 mT). The *g*-value of 2.00266 was assigned to an anilino-type radical (Fig. 5B(ii))^25^ In addition, we investigated the reaction of Et_3_SiBpin and KO^*t*^Bu in diglyme at room temperature (Fig. 5B(iii)(c)). The quartet line with a *g*-value of 2.0060 should be assigned to a silyl radical ˙SiEt_3_. This allowed us to directly detect the generated silyl radical ˙SiEt_3_ (see the ESI† for more details on the discussion of the ESR experiments).

Based on both our experimental results and previous reports,^15,16^ we proposed a reaction mechanism involving a radical-mediated defluorinative cross-coupling reaction (Fig. 5C).^28^Fig. 5C shows a schematic of the mechanism for the representative reaction of aryl fluorides 1 and diarylmethanes 2. First, Et_3_SiBpin reacts with a molecule of KO^*t*^Bu to form intermediate I; the formation of this intermediate was previously confirmed by the Avasare group based on density functional theory calculations.^29^ We also confirmed the existence of intermediate I by ^11^B NMR and ^29^Si NMR spectroscopy.^15,16^ In this step, due to the inherent steric repulsion of this ate complex I, the intermediate I splits into a sterically demanding and frustrated radical pair II^30^ consisting of a triethylsilyl radical (˙SiEt_3_) and boron-radical species *via* the homolytic scission of the Si–B bond. Hydrogen abstraction from diarylmethane 2 by ˙SiEt_3_ affords a frustrated radical pair III accompanied by the formation of HSiEt_3_ (detected by GC-MS). Then, aryl fluorides 1 participate in the cascade process *via* transition state TS-I, where the C–F bond of aryl fluorides 1 is activated by the boron atom in Bpin. Subsequently, the boron-radical side of a frustrated radical pair III in TS-I promotes a radical reaction; aryl fluorides 1 are transformed into aryl radicals *via* C–F bond cleavage by SET.^30–32^ In the meantime, the benzyl radical approaches the aryl radicals. Then, C–C bond formation is completed by the release of IV ([Bpin(O^*t*^Bu)F]K) to furnish desired cross-coupling product 3.^15,16^ Finally, the IV ([Bpin(O^*t*^Bu)F]K) further reacts with the second mole of KO^*t*^Bu to provide a stable V ([Bpin(O^*t*^Bu)_2_]K) (detected by ^11^B NMR) and KF (detected by ^19^F NMR). Some benzyl radical species in the frustrated radical pair III are competitively captured by ˙SiEt_3_ from II, providing 11, which was occasionally detected as a by-product in the experiments. The benzyl radical in III is also evidenced by the formation of Int-TEMPO. The lower yields of the coupling reactions using monoarylalkanes can be understood based on the lower stabilities of the corresponding radical species.

## Conclusions

In summary, we developed the first silylboronate-mediated radical cross-coupling reaction of aryl fluorides with arylalkanes, in which C–F bond cleavage is concomitant with the initial cleavage of a C–H bond to form a new C–C bond. A variety of triaryl- and diarylalkanes were efficiently and smoothly synthesized in moderate to excellent yields under very mild conditions at room temperature. Another important feature of the present coupling system is that it relies on C–F and C–H bond activation occurring at room temperature. This method obviates the use of transition metals and specialized ligands with high reaction temperatures. A radical reaction mechanism was suggested by the experimental results and confirmed by ESR analysis. The library of arylalkanes obtained by this method can be used as valuable scaffolds for pharmaceuticals and functional materials. As many organic fluorides are readily available, including complex pharmaceuticals,^33^ and agrochemicals^34^ we expect the radical coupling of organic fluorides with arylalkanes to be a valuable method for the straightforward preparation of various materials, such as drug candidates and specialty materials.

## Data availability

The data that support the findings of this study are available within the article and the ESI.† Details about materials and methods, experimental procedures, characterization data, mechanistic studies, ESR studies and NMR spectral are included. All relevant data are also available from the authors.

## Author contributions

JZ, ZZ, and BJ performed the experiments and analyzed the data. ZZ and KY performed ESR experiments. JZ, ZZ, BJ, KY, YS, and NS discussed the results. JZ and NS wrote the manuscript. NS supervised the project. All authors contributed to the manuscript and have approved the final version of the manuscript.

## Conflicts of interest

There are no conflicts to declare.

## Supplementary Material

SC-014-D3SC00154G-s001

## References

[cit1] Zhang J., Stanciu C., Wang B., Hussain M. M., Da C.-S., Carroll P. J., Dreher S. D., Walsh P. J. (2011). J. Am. Chem. Soc..

[cit2] Mcgrath N. A., Brichacek M., Njardarson J. T. (2010). J. Chem. Educ..

[cit3] Zhang W., Wang F., McCann S. D., Wang D., Chen P., Stahl S. S., Liu G. (2016). Science.

[cit4] Clark J. R., Feng K., Sookezian A., White M. C. (2018). Nat. Chem..

[cit5] Tanwar L., Börgel J., Ritter T. (2019). J. Am. Chem. Soc..

[cit6] Babu K. N., Massarwe F., Shioukhi I., Masarwa A. (2021). Angew. Chem., Int. Ed..

[cit7] Duxbury D. F. (1993). Chem. Rev..

[cit8] Kim H. N., Lee M. H., Kim H. J., Kim J. S., Yoon J. (2008). Chem. Soc. Rev..

[cit9] Kshatriya R., Jejurkar V. P., Saha S. (2019). Eur. J. Org. Chem..

[cit10] Zhang J., Bellomo A., Trongsiriwat N., Jia T., Carroll P. J., Dreher S. D., Tudge M. T., Yin H., Robinson J. R., Schelter E. J., Walsh P. J. (2014). J. Am. Chem. Soc..

[cit11] Nambo M., Crudden C. M. (2015). ACS Catal..

[cit12] Kiplinger J. L., Richmond T. G., Osterberg C. E. (1994). Chem. Rev..

[cit13] Li J., Wu C., Zhou B., Walsh P. J. (2018). J. Org. Chem..

[cit14] Chan A. Y., Perry I. B., Bissonnette N. B., Buksh B. F., Edwards G. A., Frye L. I., Garry O. L., Lavagnino M. N., Li B. X., Liang Y., Mao E., Millet A., Oakley J. V., Reed N. L., Sakai H. A., Seath C. P., MacMillan D. W. C. (2022). Chem. Rev..

[cit15] Zhou J., Jiang B., Fujihira Y., Zhao Z., Imai T., Shibata N. (2021). Nat. Commun..

[cit16] Cui B., Jia S., Tokunaga E., Shibata N. (2018). Nat. Commun..

[cit17] Saito T., Wang J., Tokunaga E., Tsuzuki S., Shibata N. (2018). Sci. Rep..

[cit18] Yamamoto E., Izumi K., Horita Y., Ukigai S., Ito H. (2014). Top. Catal..

[cit19] Nambo M., Crudden C. M. (2021). Chem. Rec..

[cit20] Caron S., Vazquez E., Wojcik J. M. (2000). J. Am. Chem. Soc..

[cit21] Ueno M., Yonemoto M., Hashimoto M., Wheatley A. E. H., Naka H., Kondo Y. (2007). Chem. Commun..

[cit22] Bizier N. P., Wackerly J. W., Braunstein E. D., Zhang M., Nodder S. T., Carlin S. M., Katz J. L. (2013). J. Org. Chem..

[cit23] Ji X., Huang T., Wu W., Liang F., Cao S. (2015). Org. Lett..

[cit24] Takeda M., Nagao K., Ohmiya H. (2020). Angew. Chem., Int. Ed..

[cit25] Qu B. J., Xu Y. H., Shi W. F., Rånby B. (1992). Macromolecules.

[cit26] Gasanov H. G., Dotdaev S. Kh. (1986). Russ. Chem. Bull..

[cit27] Evans J. C., Hupfiled P., Rowlands C. C., Cray S. E. (1990). J. Chem. Soc., Faraday Trans..

[cit28] As the research progressed (ref. 15 and 16), we gradually modified the reaction mechanisms proposed in each paper based on new results and discussions. We believe that the mechanism presented here is the most appropriate for understanding the reaction process so far

[cit29] Jain P., Pal S., Avasare V. (2018). Organometallics.

[cit30] (e) Holtrop F. , Jupp A. R. and Slootweg J. C., in Frustrated Lewis Pairs, Molecular Catalysis, ed. J. C. Slootweg and A. R. Jupp, Springer, Cham, 2021, vol. 2, pp. 361–385

[cit31] Nocera G., Young A., Palumbo F., Emery K. J., Coulthard G., McGuire T., Tuttle T., Murphy J. A. (2018). J. Am. Chem. Soc..

[cit32] Barham J. P., Coulthard G., Emery K. J., Doni E., Cumine F., Nocera G., John M. P., Berlouis L. E. A., McGuire T., Tuttle T., Murphy J. A. (2016). J. Am. Chem. Soc..

[cit33] Inoue M., Sumii Y., Shibata N. (2020). ACS Omega.

[cit34] Ogawa Y., Tokunaga E., Kobayashi O., Hirai K., Shibata N. (2020). iScience.

