# Supplementary material for: Synthesis of triarylmethanes by silyl radical-mediated cross-coupling of aryl fluorides and arylmethanes
Source: Chem Sci. 2023 Mar 21;14(16):4248–56. doi: 10.1039/d3sc00154g (PMC10132141; doi:10.1039/d3sc00154g)
Supplement: SC-014-D3SC00154G-s001 [file SC-014-D3SC00154G-s001.pdf]

Electronic Supplementary Material (ESI) for Chemical Science.
This journal is © The Royal Society of Chemistry 2023

Electronic Supplementary Information

Synthesis of triarylmethanes by silyl radical-mediated cross-coupling of aryl fluorides and arylmethanes

Jun Zhou,[a] Zhengyu Zhao,[a] Bingyao Jiang,[b] Katsuhiko Yamamoto,[b] Yuji Sumii,[b] and Norio Shibata*[a,b]

[a]Departments of Nanopharmaceutical Sciences, Nagoya Institute of Technology, Gokiso, Showa-ku, Nagoya 466-8555 (Japan).

[b]Departments of Life Science and Applied Chemistry, Nagoya Institute of Technology, Gokiso, Showa-ku, Nagoya 466-8555 (Japan).

*Correspondence and requests for materials should be addressed to N.S. (email: nozshiba@nitech.ac.jp)

Table of Contents

1. General Information 3
1.1 Reaction setup, chromatography, and data analysis 3
1.2 Materials and reagents 3
2. Synthesis of Starting Materials 4
2.1 Synthesis of aryl fluorides **1** 4
2.2 Synthesis of benzylic compounds and 9*H*-fluorene 7
2.3 Synthesis of organic boronates 13
3. Optimization Studies 15
3.1 Table S1. Screening for proper metal catalysts 15
3.2 Table S2. Screening for proper base 15
3.3 Table S3. Screening for the equivalent of $Et_3SiBpin$ 16
3.4 Table S4. Screening for suitable solvent 16
3.5 Table S5. Screening for proper combination of diphenylmethane and $Et_3SiBpin$ 17
4. General Procedures for the Cross-Coupling Reaction of Aryl Fluorides and Arylalkanes........... 18
4.1 General procedure for the optimization of cross-coupling reaction 18
4.2 General procedure for the cross-coupling reaction of aryl fluorides and benzylic C–H bonds 18
4.3 General procedure for the scale-up reaction 18
5. Characterization Data of Cross-Coupling Products 19
6. Preliminary Mechanistic Investigations 43
6.1 The NMR spectroscopic studies 43
6.2 Reaction with radical scavenger 45
6.3 Control experiments involve radical-clock reactions 46
6.4 Radical process regarding to the formation of **3aq** 48
6.5 Control experiment involve nucleophilic aromatic substitution (S_NAr) process 49
6.6 Electron spin resonance (ESR) studies 50
7. Unsuccessful Substrates for the Cross-Coupling Reaction 53
8. NMR Spectra (^1H NMR, ^{13}C NMR and ^{19}F NMR) 54
9. References 125

1. General Information

1.1 Reaction setup, chromatography, and data analysis

All reactions were performed in oven-dried glassware under a positive pressure of nitrogen or argon. Solvents were transferred via syringe and were introduced into the reaction vessels through a rubber septum. All solvents were dried by standard method. All the reactions were monitored by thin-layer chromatography (TLC) carried out on 0.25 mm Merck silica gel (60-F254). The TLC plates were visualized with UV light. All the reaction products were purified by column chromatography and was carried out on a column packed with silica gel 60N spherical neutral size 50-63 mm. The ^{1}H NMR (300 MHz), ^{13}C NMR (75 MHz) and ^{19}F NMR (282 MHz) spectra as for solution in $CDCl_3$ were recorded on a Varian Mercury 300. The ^{1}H NMR (500 MHz) and ^{13}C NMR (126 MHz) spectra as for solution in $CDCl_3$ were recorded on a BRUKER 500 Ultra Shield TR. The ^{1}H NMR (700 MHz) and ^{13}C NMR (176 MHz) spectra as for solution in $CDCl_3$ were recorded on a JEOL RESONANCE ECZ700R. The ^{11}B NMR (225 MHz) spectra as for solution in THF-d^8 were also recorded on a JEOL RESONANCE ECZ700R. The chemical shifts (δ) are expressed in ppm downfield from internal TMS (δ = 0.00) or $CDCl_3$ (δ = 7.26) and coupling constants (*J*) are reported in hertz (Hz). The hexafluorobenzene (C_6F_6) [δ = −162.2 ($CDCl_3$)] was used as internal standard for ^{19}F NMR. The following abbreviations were used to explain the multiplicities: s = singlet, d = doublet, t = triplet, q = quartet, p = pentalet, h = hextet, m = multiplet, br = broad. Mass spectra were recorded on a JEOL JMS-Q1050GC (EI-MS). High resolution mass spectrometry (HRMS) was carried out on an electron impact ionization mass spectrometer with a micro-TOF analyzer and recorded on a Waters, GCT Premier (EI-MS) with a TOF analyzer. Infrared spectra were recorded on a JASCO FT/IR-4100 spectrometer. Melting points were recorded on a BUCHI M-565.

1.2 Materials and reagents

Commercially available chemicals were obtained from Aldrich Chemical Co., Alfa Aesar, TCI and used as received unless otherwise noted. Solvents cyclohexane, toluene, 1,4-dioxane, DMF, DME, diglyme, triglyme, CPME, MTBE, DTBT and THF were dried before use.

2. Synthesis of Starting Materials

2.1 Synthesis of aryl fluorides 1

Fluoroarenes **1a**, **1c**, **1d**, **1e**, **1f**, **1g** and **1h** were purchased from TCI or Sigma Aldrich. **1b**, **1i**, **1j**, **1k**, **1l**, **1m**, **1n**, **1o**, **1p, 1r**, and **1t** were prepared according to the previous work.[1] A typical experimental procedure for the preparation of **1q**, **1s**, **1u** and **1v** were described below.

2-(4-Fluorophenyl)benzofuran (1q)

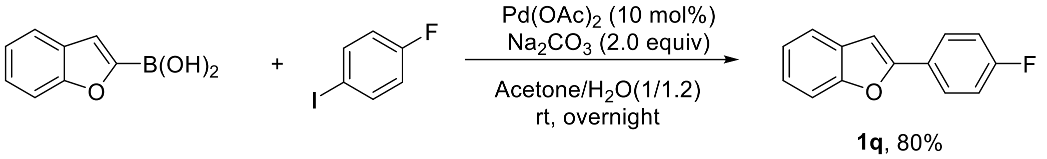

1q, 80%

The title product **1q** was synthesized according to the known procedures with modifies.[2]

A dried flask was charged with 2-benzofuranylboronic acid (607 mg, 3.75 mmol), 4-fluoroiodobenzene (0.4 mL, 3.41 mmol), $Pd(OAc)_2$ (76.6 mg, 0.34 mmol) and Na_2CO_3 (723 mg, 6.82 mmol) in acetone/H_2O (44 mL, *v*/*v* = 1/1.2). The mixture was stirred at room temperature overnight and the reaction progress was monitored by TLC. Then the mixture was added water 20 mL, and then extracted with dichloromethane. The combined organic phases were washed with brine and dried over Na_2SO_4. After concentrated under reduced pressure, the residue was purified by column chromatography on silica gel (*n*-hexane) to give title compound as white solid (579 mg, yield: 80%).

^{1}H NMR (300 MHz, $CDCl_3$) δ 7.89 – 7.76 (m, 2H), 7.63 – 7.46 (m, 2H), 7.32 – 7.19 (m, 2H), 7.20 – 7.07 (m, 2H), 6.95 (s, 1H).

^{19}F NMR (282 MHz, $CDCl_3$) δ -111.87 – -113.96 (m, 1F).

MS(EI): *m/z* 212 $[M]^+$.

The chemical shifts were consistent with those reported in the literature.[2]

9-fluorophenanthrene (1s)

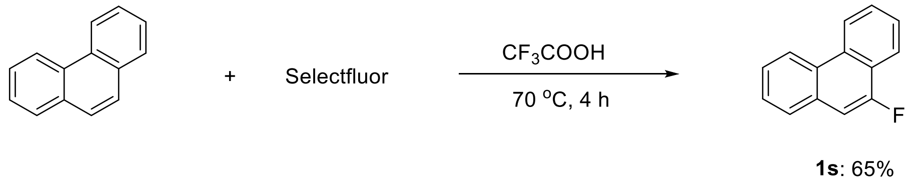

1s: 65%

The title product **1s** was synthesized according to the known procedures with modifies.[3]

A dried flask was charged with phenanthrene (0.5 g, 3.0 mmol) and Selectfluor® (1.2 g, 3.3 mmol) in trifluoroacetic acid (15 mL). The mixture was stirred at 70 °C for 4 h. After the reaction finished, remove the solvent trifluoroacetic acid under reduced pressure, then poured the residue into ice water and extracted with dichloromethane. The combined organic phases were washed with brine and dried over Na_2SO_4. After concentrated under reduced pressure, the residue was purified by column chromatography on silica gel (pentane) to give title compound as white solid (380 mg, yield: 65%).

^{1}H NMR (300 MHz, $CDCl_3$) δ 8.75 – 8.58 (m, 2H), 8.26 – 8.12 (m, 1H), 7.88 – 7.79 (m, 1H), 7.79 – 7.62 (m, 2H), 7.66 – 7.56 (m, 2H), 7.39 (d, *J* = 11.6 Hz, 1H).

^{19}F NMR (282 MHz, $CDCl_3$) δ -125.43 (d, *J* = 11.8 Hz, 1F).
MS(EI): *m/z* 196 $[M]^+$.
The chemical shifts were consistent with those reported in the literature.[3]

3-(3-Butenyl)-4-fluorobiphenyl (1u)

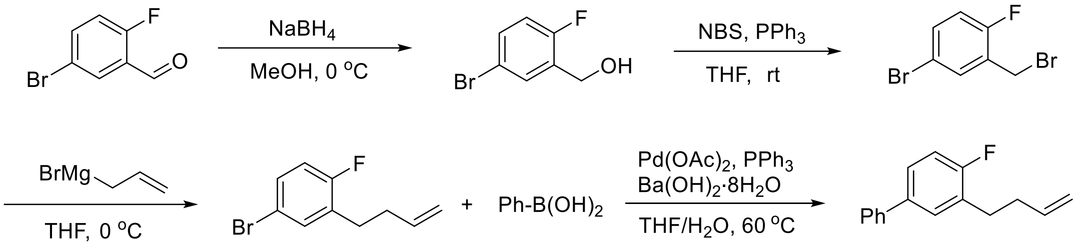

1u, 92%

The title product **1u** was synthesized according to the known procedures with modifies.[4]

To a solution of 5-bromo-2-fluorobenzaldehyde (2.03 g, 10 mmol, 1.0 equiv) in methanol (25 mL) was added sodium borohydride (0.54 g, 14 mmol, 1.4 equiv) at 0 °C. After warming to room temperature, the reaction mixture was stirred for 1 h at the same temperature. To the mixture was added aqueous hydrochloric acid (1.0 M, 15 mL) at 0 °C. The mixture was stirred for 10 min at room temperature and extracted with Et_2O. The combined organic layer was washed with brine and dried over Na_2SO_4. After concentrated under reduced pressure to give crude mixture, which was used for the next step without further purification.

To a solution of the crude mixture in THF (20 mL) were added triphenylphosphine (2.8 g, 10 mmol, 1.0 equiv) and *N*-bromosuccinimide (1.82 g, 10 mmol, 1.0 equiv) sequentially at 0 °C. After stirring for 45 min at room temperature, saturated aqueous sodium thiosulfate (10 mL) was added and diluted with Et_2O at 0 °C and stirred for 10 min. After warming to room temperature and filtration through a pad of Celite©, the filtrate was concentrated under reduced pressure to give a crude mixture, which was purificated to give 1-bromo-3-(bromomethyl)-4-fluorobenzene (1.84 g, 69%).

To a solution of 1-bromo-3-(bromomethyl)-4-fluorobenzene (0.92 g, 6.9 mmol) in THF (20 mL) was dropwise added freshly prepared allylmagnesium bromide (0.3 M solution in Et_2O, 50 mL, 15.0 mmol, 2.2 equiv) at 0 °C. After warming to room temperature, the reaction mixture was stirred for 30 min at the same temperature. To this was added saturated aqueous ammonium chloride at 0 °C. After warming to room temperature, the mixture was extracted with Et_2O. The combined organic layer was washed with brine and dried over Na_2SO_4. Then concentrated under reduced pressure to give a crude mixture, which was purificated to give 1-bromo-3-(3-butenyl)-4-fluorobenzene (1.2 g, 77%).

To a solution of 1-bromo-3-(3-butenyl)-4-fluorobenzene (1.0 g, 5 mmol) in THF (25 mL) and water (5 mL) added phenylboronic acid (0.73 g, 6.0 mmol, 1.2 equiv), palladium(II) acetate (28 mg, 0.13 mmol, 0.05 equiv), triphenylphosphine (131 mg, 0.5 mmol, 0.2 equiv), and barium hydroxide octahydrate (3.15 g, 10 mmol, 2.0 equiv) at room temperature. After stirring for 16 h under 60 °C, the reaction mixture was cooled to room temperature, and filtered through a pad of Celite©. The filtrate was then concentrated and extracted with ethyl acetate (EtOAc). The combined organic layer was washed with brine and dried over Na_2SO_4. After filtration, the filtrate was concentrated under reduced pressure and then purificated by silica-gel column chromatography (*n*-hexane/EtOAc = 30/1) to give **1u** (1.04 g, yield: 92%) as a colorless oil.

^{1}H NMR (500 MHz, $CDCl_3$) δ 7.61 – 7.55 (m, 2H), 7.50 – 7.44 (m, 3H), 7.44 – 7.40 (m, 1H), 7.41 – 7.34 (m, 1H), 7.11 (dd, *J* = 9.7, 8.4 Hz, 1H), 6.06 – 5.83 (m, 1H), 5.22 – 4.98 (m, 2H), 2.85 (td, *J* = 7.6, 1.1 Hz, 2H), 2.55 – 2.39 (m, 2H).

^{13}C NMR (126 MHz, $CDCl_3$) δ 161.0 (d, *J* = 245.6 Hz), 140.6, 137.8, 137.3, 129.6, 128.9, 127.3, 127.2, 127.1, 126.4, 115.6 (d, *J* = 22.7 Hz), 115.4, 34.4, 28.9.

^{19}F NMR (282 MHz, $CDCl_3$) δ -121.85 (q, *J* = 7.2 Hz, 1F).

MS(EI): *m/z* 226 $[M]^+$.

The chemical shifts were consistent with those reported in the literature.[4]

(3-(2-Fluorophenyl)propane-1,1-diyl)dibenzene (1v)

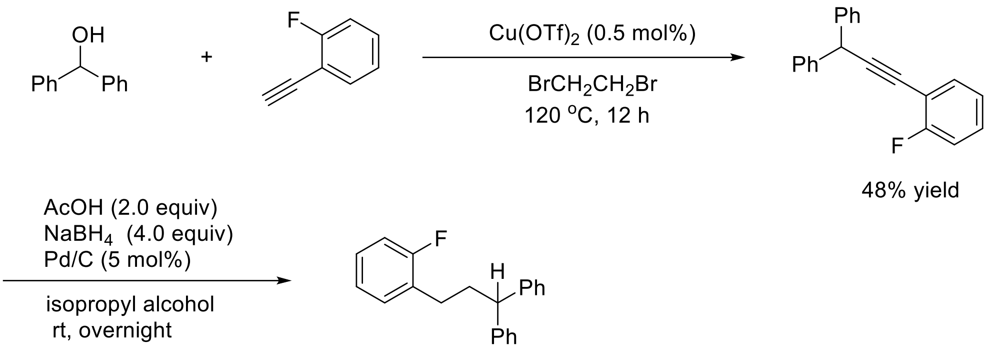

The cross-coupling intermediate was synthesized according to the known procedures.[5]

In an oven-dried flask was charged with diphenylmethanol (0.92 g, 5.0 mmol), 1-ethynyl-2-fluorobenzene (0.62 mL, 5.5 mmol), $Cu(OTf)_2$ (9 mg, 0.025 mmol), and $BrCH_2CH_2Br$ (5.0 mL). The reaction flask was heated in an oil bath at 120 °C for 12 h. Cooled to room temperature, diluted with H_2O, and extracted with Et_2O. The organic layers were combined, dried over Na_2SO_4, and concentrated to give the crude, which was further purified by flash chromatography on silica gel (*n*-hexane/DCM = 10/1) to give the cross-coupling intermediate (690 mg, 48% yield).

In an oven-dried flask was charged with (3-(2-fluorophenyl)prop-2-yne-1,1-diyl)dibenzene (572 mg, 2.0 mmol), Pd/C (10% *w*/*w*, 110 mg, 0.1 mmol), and isopropyl alcohol (10 mL). Refill the sealed flask with N_2 for 3 times and stirred at room temperature. Then AcOH (228 µL, 4.0 equiv) and sodium tetrahydroborate (302 mg, 8.0 equiv) were added to the stirring mixture. The reaction mixture was stirred at room temperature for 30 min then quenched with aqueous HCl (0.1 N) until no further gas evolues. Followed by adjust the solution pH to 10 by using aqueous NaOH (1 N) solution, diluted with H_2O, and extracted with Et_2O. The organic layers were combined, dried over Na_2SO_4, and concentrated to give the crude, which was further purified by flash chromatography on silica gel (*n*-hexane/EtOAc = 10/1) to give the titled compound as a colorless oil (543 mg, 93% yield).

^{1}H NMR (300 MHz, $CDCl_3$) δ 7.42 – 6.87 (m, 14H), 3.96 (t, *J* = 7.8 Hz, 1H), 2.62 (t, *J* = 7.8 Hz, 2H), 2.38 (q, *J* = 7.9 Hz, 2H).

^{19}F NMR (282 MHz, $CDCl_3$) δ -118.55 (p, *J* = 6.2 Hz, 1F).

MS(EI): *m/z* 290 $[M]^+$.

The chemical shifts were consistent with those reported in the literature.[6]

2.2 Synthesis of benzylic compounds and 9*H*-fluorene

Annulated diarylmethane derivatives 9,10-dihydroanthracene (**2h**), 9*H*-xanthene (**2i**), and 1,1-diphenylethane (**2j**), cumene (**2m**), ethylbenzene (**2n**), butylbenzene (**2o**), 4-phenyl-1-butene (**2p**), allylbenzene (**2q**) were purchased from TCI or Sigma Aldrich. A typical known experimental procedure for the preparation of diarylmethanes **2a**, **2b**, **2c**, **2d**, **2e**, **2f**, **2g**, **9*H*-fluorene**, **2k**, **2l**, **2s**, **2t**, **2u** and **3aq'** were described below.

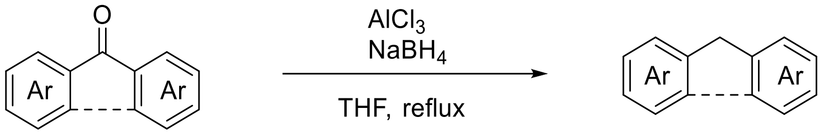

General procedure A: The diarylmethanes was synthesized according to the known procedures with modifies.[7] A dried flask was charged with benzophenones, sodium borohydride, and anhydrous aluminum chloride in anhydrous THF. The mixture was stirred under reflux for 2 h and the reaction progress was monitored by TLC. After the ketones fully consumed, the mixture was cooled to room temperature. To this mixture was slowly added water 10 mL, and then extracted with EtOAc. The combined organic layer was washed with brine and dried over Na_2SO_4. After filtration, the filtrate was concentrated under reduced pressure to give the crude, which was purified by column chromatography on silica gel (*n*-hexane) to give corresponding diarylmethanes **2** or **9*H*-fluorene**.

Diphenylmethane (2a)

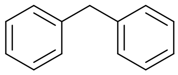

According to **General Procedure A**, benzophenone (1.82 g, 10 mmol), sodium borohydride (1.90 g, 50 mmol), anhydrous aluminum chloride (4.0 g, 30 mmol), and anhydrous THF (50 mL) were used. The title product **2a** was isolated as a white solid after flash chromatography (1.66 g, yield: 99%).

^{1}H NMR (300 MHz, $CDCl_3$) δ 7.51 – 6.99 (m, 10H), 4.03 (s, 2H).

MS(EI): *m/z* 168 $[M]^+$.

The chemical shifts were consistent with those reported in the literature.[7]

4-Methoxydiphenylmethane (2b)

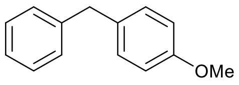

According to **General Procedure A**, 4-methoxybenzophenone (3.18 g, 15 mmol), sodium borohydride (2.85 g, 75 mmol), anhydrous aluminum chloride (6.00 g, 45 mmol), and anhydrous THF (60 mL) were used. The title product **2b** was isolated as a white solid after flash chromatography (2.64 g, yield: 89%).

^{1}H NMR (300 MHz, $CDCl_3$) δ 7.33 – 7.23 (m, 2H), 7.21 – 7.14 (m, 3H), 7.10 (d, *J* = 8.4 Hz, 2H), 6.88 – 6.79 (m, 2H), 3.92 (s, 2H), 3.77 (s, 3H).

MS(EI): *m/z* 198 $[M]^+$.

The chemical shifts were consistent with those reported in the literature.[8]

Dianisylmethan (2c)

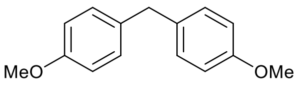

According to **General Procedure A**, 4,4'-diMethoxybenzophenone (0.97 g, 4 mmol), sodium borohydride (0.76 g, 20 mmol), anhydrous aluminum chloride (1.60 g, 12 mmol), and anhydrous THF (20 mL) were used. The title product **2c** was isolated as a white solid after flash chromatography (1.96 g, yield: 86%).

^{1}H NMR (300 MHz, $CDCl_3$) δ 7.13 (d, *J* = 8.2 Hz, 4H), 6.86 (d, *J* = 8.5 Hz, 4H), 3.90 (s, 2H), 3.81 (s, 6H).

MS(EI): *m/z* 228 $[M]^+$.

The chemical shifts were consistent with those reported in the literature.[9]

Di-*p*-tolylmethane (2d)

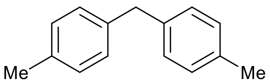

According to **General Procedure A**, 4,4'-dimethylbenzophenone (1.05 g, 5 mmol), sodium borohydride (0.95 g, 25 mmol), anhydrous aluminum chloride (2.00 g, 15 mmol), and anhydrous THF (25 mL) were used. The title product **2d** was isolated as a white solid after flash chromatography (1.90 g, yield: 97%).

^{1}H NMR (300 MHz, $CDCl_3$) δ 7.02 (s, 8H), 3.88 (s, 2H), 2.29 (s, 6H).

MS(EI): *m/z* 196 $[M]^+$.

The chemical shifts were consistent with those reported in the literature.[9]

1-Benzyl-2-methylbenzene (2e)

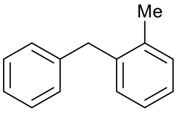

According to **General Procedure A**, 2-methylbenzophenone (1.96 g, 10 mmol), sodium borohydride (1.90 g, 50 mmol), anhydrous aluminum chloride (4.00 g, 30 mmol), and anhydrous THF (50 mL) were used. The title product **2e** was isolated as a white solid after flash chromatography (1.50 g, yield: 82%).

^{1}H NMR (300 MHz, $CDCl_3$) δ 7.52 – 6.73 (m, 9H), 3.98 (s, 2H), 2.23 (s, 3H).

MS(EI): *m/z* 182 $[M]^+$.

The chemical shifts were consistent with those reported in the literature.[10]

1-Benzyl-4-chlorobenzene (2f)

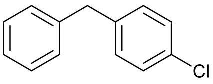

According to **General Procedure A**, 4-chlorobenzophenone (2.16 g, 10 mmol), sodium borohydride (1.90 g, 50 mmol), anhydrous aluminum chloride (4.00 g, 30 mmol), and anhydrous THF (50 mL) were used. The title product **2f** was isolated as a white solid after flash chromatography (1.47 g, yield: 73%).

^{1}H NMR (300 MHz, $CDCl_3$) δ 7.34 – 7.23 (m, 4H), 7.23 – 7.14 (m, 5H), 3.98 (s, 2H).

MS(EI): *m/z* 202 $[M]^+$.

The chemical shifts were consistent with those reported in the literature.[11]

2-Benzylthiophene (2g)

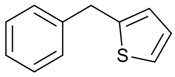

According to **General Procedure A**, 2-benzoylthiophene (1.88 g, 10 mmol), sodium borohydride (1.90 g, 50 mmol), anhydrous aluminum chloride (4.00 g, 30 mmol), and anhydrous THF (50 mL) were used. The title product **2g** was isolated as a colorless oil after flash chromatography (1.60 g, yield: 91%).

^{1}H NMR (300 MHz, $CDCl_3$) δ 7.32 – 7.14 (m, 5H), 7.08 (dd, *J* = 5.1, 1.2 Hz, 1H), 6.88 (dd, *J* = 5.1, 3.4 Hz, 1H), 6.78 – 6.73 (m, 1H), 4.10 (s, 2H).

MS(EI): *m/z* 174 $[M]^+$.

The chemical shifts were consistent with those reported in the literature.[12]

9*H*-Fluorene

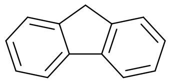

According to **General Procedure A**, 9-fluorenone (1.80 g, 10 mmol), sodium borohydride (1.90 g, 50 mmol), anhydrous aluminum chloride (4.00 g, 30 mmol), and anhydrous THF (50 mL) were used. The title product was isolated as a white solid after flash chromatography (1.54 g, yield: 93%).

^{1}H NMR (300 MHz, $CDCl_3$) δ 7.81 (d, *J* = 7.0 Hz, 2H), 7.57 (d, *J* = 6.7 Hz, 2H), 7.51 – 7.04 (m, 4H), 3.93 (s, 2H).

MS(EI): *m/z* 166 $[M]^+$.

The chemical shifts were consistent with those reported in the literature.[13]

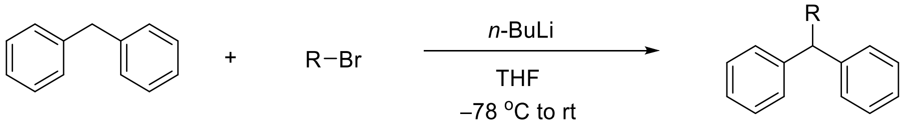

General procedure B: The diarylmethane derivatives was synthesized according to the known procedures with modifies.[14] A dried flask was charged with diphenylmethane and anhydrous THF. The mixture was stirred under –78 °C, and then *n*-butyllithium (1.6 M in *n*-hexane) was added dropwise, after addition finished, the mixture was allowed to warm up to 0 °C and keep stirring for 1 h. Then alkyl bromide in THF (1.0 M) was added dropwise and keep stirring for another 1 h under 0 °C followed by stirring at room temperature for 24 h. To this mixture was slowly added water, and then extracted with EtOAc. The combined organic layer was washed with brine, and dried over Na_2SO_4. After filtration, the filtrate was concentrated under reduced pressure to give the crude, which was purified by column chromatography on silica gel (*n*-hexane) to give corresponding diarylmethane derivatives **2k** and **2l**.

Propane-1,1-diyldibenzene (2k)

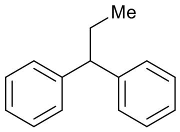

According to **General Procedure B**, diphenylmethane (1.68 g, 10 mmol), *n*-butyllithium (12.5 mL, 20 mmol), anhydrous ethyl bromide (1.11 mL, 15 mmol), and anhydrous THF (20 mL) were used. The title product **2k** was isolated as a white solid after flash chromatography (1.55 g, yield: 79%).

^{1}H NMR (300 MHz, $CDCl_3$) δ 7.38 – 7.03 (m, 10H), 3.79 (t, *J* = 7.8 Hz, 1H), 2.08 (p, *J* = 7.4 Hz, 2H), 0.90 (t, *J* = 7.3 Hz, 3H).

MS(EI): *m/z* 196 $[M]^+$.

The chemical shifts were consistent with those reported in the literature.[15]

Pentane-1,1-diyldibenzene (2l)

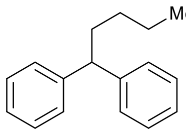

According to **General Procedure B**, diphenylmethane (1.68 g, 10 mmol), *n*-butyllithium (12.5 mL, 20 mmol), anhydrous butyl bromide (1.60 mL, 15 mmol), and anhydrous THF (20 mL) were used. The title product **2l** was isolated as a white solid after flash chromatography (2.0 g, yield: 89%).

^{1}H NMR (300 MHz, $CDCl_3$) δ 7.37 – 7.08 (m, 10H), 3.88 (t, *J* = 7.8 Hz, 1H), 2.04 (q, *J* = 7.7 Hz, 2H), 1.44 – 1.14 (m, 4H), 0.86 (t, *J* = 7.1 Hz, 3H).

MS(EI): *m/z* 224 $[M]^+$.

The chemical shifts were consistent with those reported in the literature.[16]

(8*R*,9*S*,13*S*,14*S*)-3,17-Dimethoxy-13-methyl-7,8,9,11,12,13,14,15,16,17-decahydro-6*H*-cyclopenta[*a*]phenanthrene (2s)

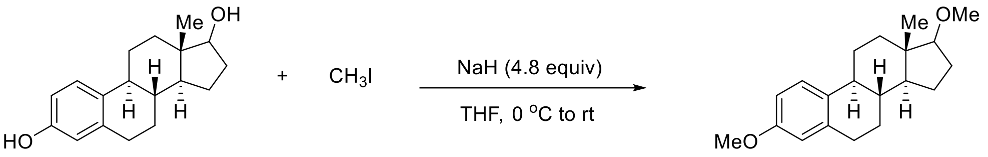

2s: 86%

To a flame dried flask, NaH (0.23 g, 9.6 mmol, 60% in mineral oil) was added to a solution of α-estradiol (0.544 g, 2.0 mmol) in THF (25 mL) under 0 °C, and the mixture was keep stirring for 30 min. A solution of iodomethane (1.16 mL, 18.6 mmol) in THF (5 mL) was added dropwise and then stirred at room temperature overnight. After the reaction finished ice water was added, quenched with saturated ammonium chloride solution and the mixture was extracted with EtOAc. The organic layer was combined and washed with brine, then dried over Na_2SO_4. Filtered and evaporated under reduced pressure. The crude was purified by column chromatography on silica gel (*n*-hexane/EtOAc = 20/1) to give titled compound as a white solid (0.52 g, yield: 86%).

^{1}H NMR (300 MHz, $CDCl_3$) δ 7.21 (d, *J* = 8.6 Hz, 1H), 6.71 (dd, *J* = 8.6, 2.8 Hz, 1H), 6.63 (d, *J* = 2.8 Hz, 1H), 3.78 (s, 3H), 3.39 (s, 3H), 3.32 (t, *J* = 8.3 Hz, 1H), 2.95 – 2.74 (m, 2H), 2.37 – 2.13 (m, 2H), 2.10 – 1.99 (m, 2H), 1.93 – 1.82 (m, 1H), 1.76 – 1.60 (m, 1H), 1.59 – 1.18 (m, 7H), 0.79 (s, 3H).

MS(EI): *m/z* 300 $[M]^+$.

The chemical shifts were consistent with those reported in the literature.[17]

2-(4-(4-Methoxybenzyl)phenoxy)-*N*,*N*-dimethylethan-1-amine (2t)

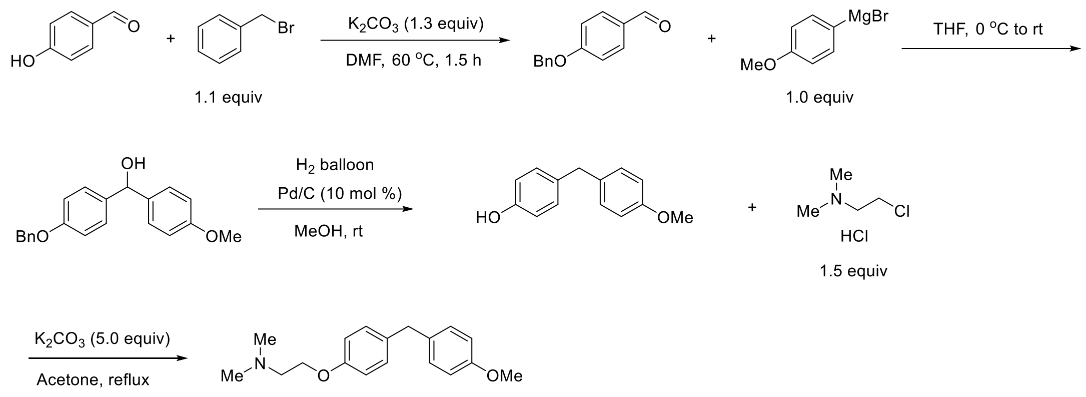

2t: 89%

The diarylmethane derivative **2t** was synthesized according to the known procedures with modifies.[18]

To a flame dried flask were added 4-hydroxybenzaldehyde (1.22 g, 10.0 mmol), benzyl bromide (1.3 mL, 11.0 mmol), K_2CO_3 (1.8 g, 13.0 mmol) and DMF (5 mL). Seal and stir the mixture at 60 °C for 1.5 h. Then quench the reaction with saturated ammonium chloride solution after cooled to room temperature. Extracted with CH_2Cl_2, and dry the combined organic extracts over Na_2SO_4, filtered and evaporated under reduced pressure. The crude was purified by column chromatography on silica gel (*n*-hexane/EtOAc = 20/1) to give 4-(benzyloxy)benzaldehyde as a white solid (1.70 g, 80%).

To a flame dried flask was added 4-(benzyloxy)benzaldehyde (1.65 g, 7.75 mmol) and THF (10 mL) at room temperature under N_2 atmosphere, and the mixture was slowly added freshly prepared 4-methoxyphenylmagnesium bromide solution (7.8 mL, 7.8 mmol, approximately 1.0 M in THF) at 0 °C, and then warm up to room temperature while keep stirring overnight. After the reaction finished, quenched with saturated ammonium chloride solution, and the mixture was extracted with EtOAc. The organic layer was combined and washed with brine, then dried over Na_2SO_4. Filtered and evaporated under reduced pressure. The crude was purified by column chromatography on silica gel (*n*-hexane/EtOAc = 10/1) to give (4-(benzyloxy)phenyl)(4-methoxyphenyl)methanol as a white solid (1.80 g, 73%).

To a flame dried flask was added (4-(benzyloxy)phenyl)(4-methoxyphenyl)methanol (1.28 g, 4.0 mmol), methanol (30 mL), and then Pd/C (40 mg). The mixture was stirred under hydrogen atmosphere at room temperature for 3 h. After the catalyst was removed, filtrate was evaporated in vacuo to give 4-(4-methoxybenzyl)phenol as a white solid (0.66 g, 77%).

To a flame dried flask was added 4-(4-methoxybenzyl)phenol (0.428 g, 2.0 mmol), 2-chloro-*N*,*N*-dimethylethanamine hydrochloride (0.432 g, 3 mmol), anhydrous acetone (30 mL), and then K_2CO_3 (1.38 g, 10 mmol). The mixture was stirred while reflux overnight. After the reaction finished, the mixture was filtered through a short pad of silica gel, washed with EtOAc, the filtrate was evaporated in vacuo give the crude, which was purified by column chromatography on silica gel (EtOAc) to give the titled compound as a white solid (0.60 g, yield: 89%).

^{1}H NMR (300 MHz, $CDCl_3$) δ 7.09 (dd, *J* = 8.6, 3.6 Hz, 4H), 6.84 (dd, *J* = 8.6, 6.4 Hz, 4H), 4.04 (t, *J* = 5.8 Hz, 2H), 3.87 (s, 2H), 3.78 (s, 3H), 2.72 (t, *J* = 5.8 Hz, 2H), 2.34 (s, 6H).

^{13}C NMR (75 MHz, $CDCl_3$) δ 158.0, 157.3, 133.9, 133.8, 129.8, 129.7, 114.6, 113.9, 66.1, 58.5, 55.3, 46.0, 40.2.

MS(EI): *m/z* 285 $[M]^+$.

The chemical shifts were consistent with those reported in the literature.[18]

1-(Cyclopropyl(phenyl)methyl)-4-methoxybenzene (2u)

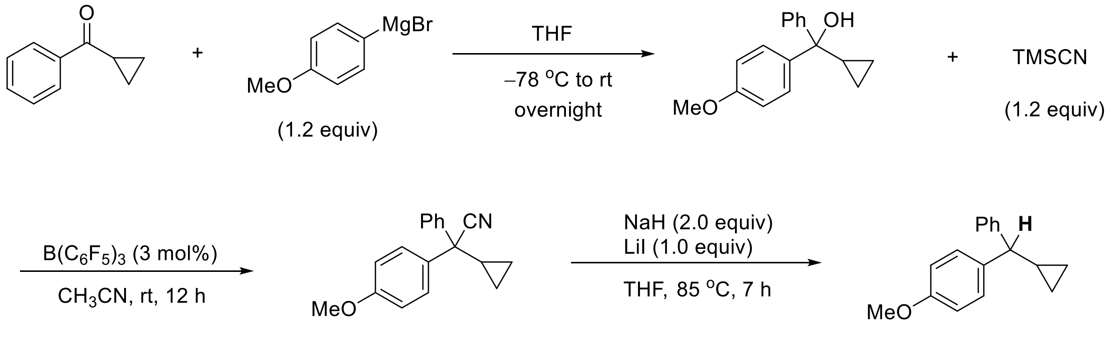

2u: 95%

The cyclopropyl-containing diarylmethane derivative **2u** was synthesized according to the known procedures with modifies.[19]

To a solution of cyclopropyl(phenyl)methanone (1.46 g, 10.0 mmol) in anhydrous THF (10 mL) was added (4-methoxyphenyl)magnesium bromide (freshly prepared from 1-bromo-4- methoxybenzene (2.24 g, 12.0 mmol) and Mg (0.37 g, 15.0 mmol) in 10 mL of THF) dropwise at –78 °C. The reaction was then allowed to warm up to room temperature and stirred for 2 h. After completion, the reaction was quenched with aqueous HCl (1 N) at 0 °C and the organic materials were extracted twice with diethyl ether. The combined organic layers were washed with brine, dried over anhydrous Na_2SO_4, filtered, and concentrated in vacuo. Then the obtained residue was purified over silica gel flash column chromatography (*n*-hexane/EtOAc: 4/1) to give cyclopropyl(4-methoxyphenyl)(phenyl)methanol as a white solid (2.33 g, 91%).

To a stirred solution of cyclopropyl(4-methoxyphenyl)(phenyl)methanol (1.27 g, 5.0 mmol) and $B(C_6F_5)_3$ (77 mg, 0.15 mmol) in CH_3CN (5 mL) was added dropwise trimethylsilyl cyanide (0.6 g, 6.0 mmol). The reaction mixture was stirred at room temperature for 12 h. Volatile materials were removed in vacuo and the crude residue was purified by silica gel flash column chromatography (*n*-hexane/EtOAc: 8/1) to give 2-cyclopropyl-2-(4-methoxyphenyl)-2-phenylacetonitrile as a colorless oil (1.04 g, 79%).

To a mixture of NaH (60% dispersion in mineral oil; 240 mg, 6.0 mmol) and LiI (402 mg, 3.0 mmol) in an oven-dried flask was added a solution of 2-cyclopropyl-2-(4-methoxyphenyl)-2-phenylacetonitrile (0.79 g, 3.0 mmol) in THF (10 mL). The reaction mixture was stirred at 85 °C overnight. After cooling to room temperature, the reaction was then quenched with water at 0 °C and extracted with Et_2O. The combined organic layers were washed with brine, dried over Na_2SO_4. Concentrated and the crude residue was purified by silica gel flash column chromatography (*n*-hexane/EtOAc: 20/1) to give titled compound as a colorless oil (680 mg, 95%).

^{1}H NMR (300 MHz, $CDCl_3$) δ 7.32 – 7.23 (m, 4H), 7.21 – 7.13 (m, 3H), 6.90 – 6.74 (m, 2H), 3.76 (s, 3H), 3.16 (d, *J* = 9.5 Hz, 1H), 1.42 – 1.25 (m, 1H), 0.73 – 0.53 (m, 2H), 0.34 – 0.17 (m, 2H).

MS(EI): *m/z* 238 $[M]^+$.

The chemical shifts were consistent with those reported in the literature.[19]

4-(1-Phenylallyl)biphenyl (3aq')

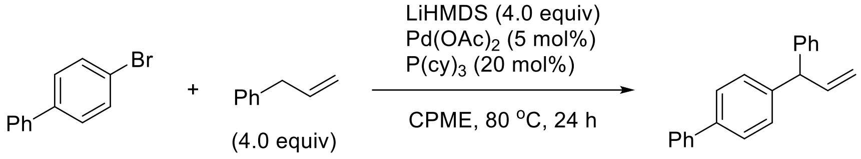

3aq': 70%

The title compound **3aq'** was synthesized according to the known procedures with modifies.[20]

In a nitrogen filled glovebox, to an oven-dried flask was charged with $LiN(SiMe_3)_2$ (3.34 g, 20 mmol), $Pd(OAc)_2$ (57 mg, 0.25 mmol) and $P(cy)_3$ (280 mg, 1.0 mmol), then dry CPME (25 mL) was added to the mixture. After stirring for 5 min at room temperature, allylbenzene (2.7 mL, 20 mmol) was added to the reaction mixture followed by biphenyl bromide (1.17 g, 5 mmol). The reaction mixture was stirred in an oil-bath for 24 h at 80 °C, cooled, quenched with few drops of H_2O, diluted with 10 mL of EtOAc, and filtered over a pad of silica. The pad was washed with EtOAc, and the solution was concentrated in vacuo. The crude was purified by silica gel flash column chromatography (*n*-hexane/EtOAc: 50/1) to give titled compound as a colorless oil (0.95 g, 70%).

^{1}H NMR (300 MHz, $CDCl_3$) δ 7.64 – 7.47 (m, 4H), 7.46 – 7.36 (m, 2H), 7.36 – 7.14 (m, 8H), 6.44 – 6.21 (m, 1H), 5.24 (d, J = 9.2 Hz, 1H), 5.03 (d, J = 17.0 Hz, 1H), 4.76 (d, J = 6.6 Hz, 1H).

^{13}C NMR (75 MHz, $CDCl_3$) δ 143.3, 142.5, 141.0, 140.6, 139.4, 129.1, 128.9, 128.7, 128.6, 127.3, 127.2, 126.6, 116.6, 54.8.

IR (KBr): 3082, 3058, 3028, 2977, 2885, 1636, 1600, 1489, 1449, 1407, 1269, 1072, 1008, 994, 917, 849, 762, 697 cm^{-1}.

HRMS (EI) [$C_{21}H_{18}$] $[M]^+$ calculated: 270.1409, found: 270.1412.

2.3 Synthesis of organic boronates

Synthesis of triethyl(4,4,5,5-tetramethyl-1,3,2-dioxaborolan-2-yl)silane ($Et_3SiBpin$)[1]

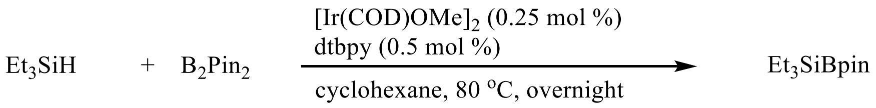

Silylboranate $Et_3SiBpin$ were prepared according to procedures previously reported.[1]

An oven-dried flask was charged with $[Ir(COD)OMe]_2$ (66.3 mg, 0.1 mmol), dtbpy (54.0 mg, 0.2 mmol), B_2pin_2 (10.0 g, 40 mmol), cyclohexane (10.0 mL), and triethylsilane (25.0 mL, 80 mmol) inside a nitrogen filled glovebox. The resulting dark brown solution was heated at 80 °C overnight outside the glovebox. After being cooled to room temperature, the crude reaction mixture was concentrated in vacuo, and the residue was purified by flash column chromatography to afford the $Et_3SiBpin$ as colorless oil (7.25 g, yield: 75%).

^{1}H NMR (300 MHz, $CDCl_3$) δ 1.22 (s, 12H), 0.95 (t, J = 8.0 Hz, 9H), 0.59 (dd, J = 15.8, 7.9 Hz, 6H).

MS(EI): *m/z* 242 $[M]^+$.

The chemical shifts were consistent with previous reported data.[1]

2-Benzhydryl-4,4,5,5-tetramethyl-1,3,2-dioxaborolane (10)

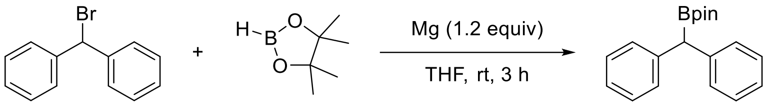

10: 63%

The synthetic procedure of **10** was followed by the reported procedures.[21]

An oven-dried flask was charged with magnesium turnings (290 mg, 12 mmol), which was activated by addition of iodine crystals and warming until iodine sublimed. The flask was cooled to room temperature and was protected by Ar balloon. Dry THF (25 mL) was added to the flask, followed by the addition of neat pinacolborane (1.2 mL, 12 mmol). bromodiphenylmethane (2.46 g, 10 mmol) diluted in dry THF (5 mL) was then added dropwise over 10 min at room temperature. After stirring for 3 h, the reaction mixture was cooled to 0 °C and acidified with aqueous HCl (15 mL, 3 N) (**Caution!** H_2 gas evolution). After stirring for 10 min, the reaction mixture was warmed to room temperature and keep stirring for an additional 30 min. The reaction mixture was then extracted with diethyl ether, dried over anhydrous Na_2SO_4, filtered, and concentrated in vacuo. Then the obtained residue was purified over silica gel flash column chromatography (*n*-hexane/EtOAc: 40/1) to give the titled compound as a white solid (1.06 g, 63%).
^{1}H NMR (300 MHz, $CDCl_3$) δ 7.34 – 7.10 (m, 10H), 3.87 (s, 1H), 1.23 (s, 12H).
MS(EI): *m*/*z* 294 $[M]^+$.
The chemical shifts were consistent with previous reported data.[22]

3. Optimization Studies

3.1 Table S1. Screening for proper metal catalysts[a]

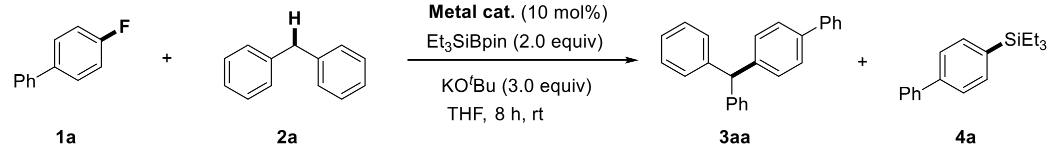

Entry	**2a** (equiv)	**Metal Cat.**	Yield of **3aa**[b]	**4a**
1	2.0	$Ni(COD)_2$	37%	+
2	2.0	$NiBr_2$·Diglyme	24%	+
3	2.0	$NiCl_2(PPh_3)_2$	33%	+
4	2.0	CuI	41%	+
5	2.0	$CoCl_2$	38%	+
**6**	**2.0**	--	**47%**	+

[a] Reactions were attempted under indicated reagents and conditions: **1a** (17.2 mg, 0.1 mmol), **2a** (33.6 mg, 0.2 mmol), metal catalyst (10 mol%), Et_3SiBpin (48.4 mg, 0.2 mmol), KO*t*Bu (33.6 mg, 0.3 mmol) in dry THF (1.0 mL) were reacted at room temperature for 8 h. [b] Determined by ^{19}F NMR and ^{1}H NMR spectroscopy using 3-fluoropyridine as an internal standard.

3.2 Table S2. Screening for proper base[a]

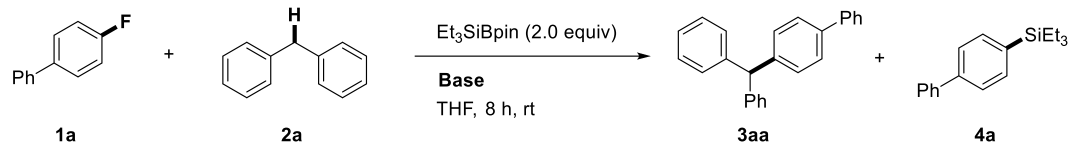

Entry	**2a** (equiv)	Base (equiv)	Yield of **3aa**[b]	**4a**
1	2.0	KO*t*Bu (3.0)	47%	+
2	2.0	K_2CO_3 (3.0)	--	--
3	2.0	NaO*t*Bu (3.0)	28%	+
4	2.0	KHMDS (3.0)	30%	+
5	2.0	Cs_2CO_3 (3.0)	--	--
**6**	**2.0**	**KO*t*Bu (4.0)**	**49%**	+
7	2.0	--	--	--

[a] Reactions were attempted under indicated reagents and conditions: **1a** (17.2 mg, 0.1 mmol), **2a** (33.6 mg, 0.2 mmol), Et_3SiBpin (48.4 mg, 0.2 mmol), base in dry THF (1.0 mL) were reacted at room temperature for 8 h. [b] Determined by ^{19}F NMR and ^{1}H NMR spectroscopy using 3-fluoropyridine as an internal standard.

3.3 Table S3. Screening for the equivalent of **Et_3SiBpin**[a]

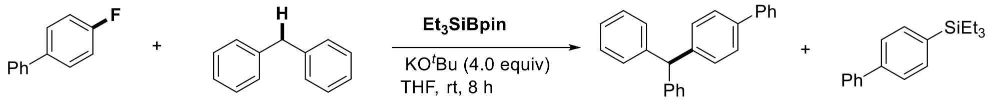

1a **2a** **3aa** **4a**

Entry	**2a** (equiv)	**Et_3SiBpin** (equiv)	Yield of **3aa**[b]	**4a**
1	2.0	1.0	14%	+
2	2.0	2.0	49%	+
3	2.0	3.0	76%	+
4	2.0	--	--	--

[a] Reactions were attempted under indicated reagents and conditions: **1a** (17.2 mg, 0.1 mmol), **2a** (33.6 mg, 0.2 mmol), Et_3SiBpin, KO^{t}Bu (45 mg, 0.4 mmol) in dry THF (1.0 mL) were reacted at room temperature for 8 h. [b] Determined by ^{19}F NMR and ^{1}H NMR spectroscopy using 3-fluoropyridine as an internal standard.

3.4 Table S4. Screening for suitable **solvent**[a]

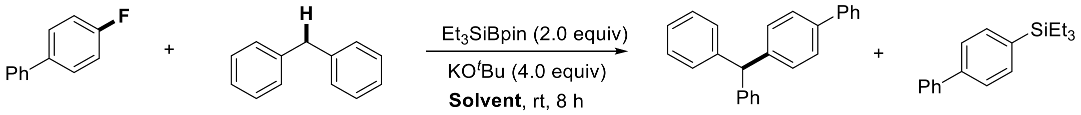

1a **2a** **3aa** **4a**

Entry	**2a** (equiv)	**solvent**	Yield of **3aa**[b]	**4a**
1	2.0	Cyclohexane/THF (8/1, *v/v*)	34%	+
2	2.0	Cyclohexane	9%	+
3	2.0	Toluene	11%	+
4	2.0	Dioxane	trace	+
5	2.0	DME	36%	+
6	2.0	CPME	18%	+
7	2.0	MTBE	12%	+
8	2.0	DTBT	trace	+
**9**	**2.0**	**diglyme**	**95%**	**--**
10	2.0	triglyme	64%	--

[a] Reactions were attempted under indicated reagents and conditions: **1a** (17.2 mg, 0.1 mmol), **2a** (33.6 mg, 0.2 mmol), Et_3SiBpin (48.4 mg, 0.2 mmol), KO^{t}Bu (45 mg, 0.4 mmol) in corresponding solvent (1.0 mL) were reacted at room temperature for 8 h.

[b] Determined by ^{19}F NMR and ^{1}H NMR spectroscopy using 3-fluoropyridine as an internal standard.

3.5 Table S5. Screening for proper combination of diphenylmethane and $Et_3SiBpin^a$

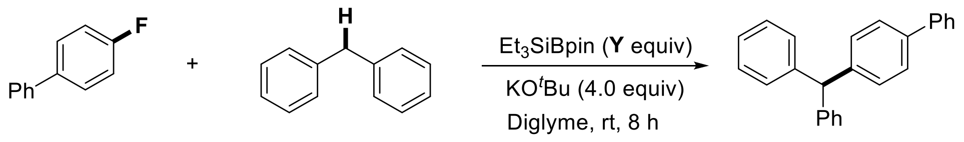

1a **2a** (**X** equiv) **3aa**

Entry	**2a** (**X** equiv)	**Et3SiBpin** (**Y** equiv)	Yield of **3aa**[b]
1	2.0	2.0	95%
2	1.5	2.0	91%
3	1.2	2.0	83%
4	1.5	3.0	95%
5[c]	2.0	2.0	96%(93%)

[a] Reactions were attempted under indicated reagents and conditions: **1a** (17.2 mg, 0.1 mmol), **2a**, $Et_3SiBpin$, KO*t*Bu (45 mg, 0.4 mmol) in dry diglyme (1.0 mL) were reacted at room temperature for 8 h. [b] Determined by ^{19}F NMR and ^{1}H NMR spectroscopy using 3-fluoropyridine as an internal standard. The number in parentheses referred to the isolated yield. [c] 0.2 mmol scale were performed.

4. General Procedures for the Cross-Coupling Reaction of Aryl Fluorides and Arylalkanes

4.1 General procedure for the optimization of cross-coupling reaction

General procedure C: In a N_2 filled glovebox, to a flame-dried screw-capped test tube was added 4-fluorobiphenyl **1a** (17.2 mg, 0.1 mmol, 1.0 equiv), silyl boronates, metal catalysts (10 mol %) or without metal catalysts, diphenylmethane **2a**, base and solvent (1.0 mL) sequentially. The tube then was sealed and removed from the glovebox. The solution was stirred at room temperature for 8 h. The reaction was diluted with Et_2O (5 mL), quenched with H_2O (5 mL), then extracted with Et_2O, washed with brine, dried over Na_2SO_4, then concentrated under vacuum, followed by 3-fluoropyridine (8.6 μL, 0.1 mmol) as an internal standard. After NMR analysis. The mixture was then concentrated again to give the crude, which was purified by column chromatography on silica gel to give the 4-benzhydrylbiphenyl **3aa**.

4.2 General procedure for the cross-coupling reaction of aryl fluorides and benzylic C–H bonds

General procedure D: In a N_2 filled glovebox, to a flame-dried screw-capped test tube was added aryl fluorides **1** (0.2 mmol, 1.0 equiv), silyl boronates $Et_3SiBpin$ (0.4 mmol, 2.0 equiv), arylalkanes **2** (0.4 mmol, 2.0 equiv), KO*t*Bu (89.6 mg, 0.8 mmol, 4.0 equiv) and dry diglyme (2.0 mL) sequentially. The tube then was sealed and removed from the glovebox. The solution was stirred at room temperature for 8 h. The reaction was diluted with Et_2O (5 mL), quenched with H_2O (5 mL), then extracted with Et_2O, washed with brine, dried over Na_2SO_4, then concentrated under vacuum, followed by adding 3-fluoropyridine (8.6 μL, 0.1 mmol) as an internal standard. After NMR analysis was conducted. The mixture was then concentrated again to give the crude, which was purified by column chromatography on silica gel to give the corresponding triarylalkanes or diarylalkanes **3**.

4.3 General procedure for the scale-up reaction

General procedure E: In a N_2 filled glovebox, to a flame-dried flask was added 4-fluorobiphenyl **1a** (0.688 g, 4.0 mmol, 1.0 equiv), KOtBu (1.80 g, 16.0 mmol, 4.0 equiv), dry diglyme (40 mL), and diphenylmethane **2a** (1.33 mL, 8.0 mmol, 2.0 equiv), sequentially. Then a solution of $Et_3SiBpin$ (1.94 g, 8.0 mmol, 2.0 equiv) in 5.0 mL dry diglyme was added to the flask slowly over 30 minutes after the flask was sealed and moved out from the glovebox. The solution was stirred at room temperature for 8 h. The reaction mixture was diluted with Et_2O (100.0 mL), quenched with saturated NH_4Cl solution, then extracted with Et_2O, washed with water and brine, dried over Na_2SO_4, then concentrated under vacuum to give the crude, which was purified by column chromatography on silica gel to give the corresponding triarylmethane **3aa** (1.09 g, 85% yield).

5. Characterization Data of Cross-Coupling Products

4-Benzhydrylbiphenyl (3aa)

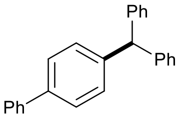

Compound **3aa** was prepared according to the general procedure **D** start from 4-fluorobiphenyl (34.4 mg, 0.2 mmol), and purified by silica gel column chromatography (*n*-hexane/DCM = 10/1) as a white solid (59.5 mg, yield: 93%).

^{1}H NMR (300 MHz, $CDCl_3$) δ 7.65 – 7.49 (m, 4H), 7.44 (t, *J* = 7.6 Hz, 2H), 7.38 – 7.00 (m, 13H), 5.61 (s, 1H).

MS(EI): *m/z* 320 $[M]^+$.

The chemical shifts were consistent with those reported in the literature.[23]

3-Benzhydrylbiphenyl (3ba)

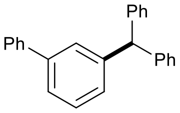

Compound **3ba** was prepared according to the general procedure **D** start from 3-fluorobiphenyl (34.4 mg, 0.2 mmol), and purified by silica gel column chromatography (*n*-hexane/DCM = 10/1) as a white solid (57.0 mg, yield: 89%).

m.p. = 79.1 – 79.9 ^oC.

^{1}H NMR (500 MHz, $CDCl_3$) δ 7.58 – 7.52 (m, 2H), 7.51 – 7.46 (m, 1H), 7.46 – 7.35 (m, 4H), 7.37 – 7.29 (m, 5H), 7.29 – 7.22 (m, 2H), 7.23 – 7.17 (m, 4H), 7.13 (d, *J* = 7.7 Hz, 1H), 5.65 (s, 1H).

^{13}C NMR (126 MHz, $CDCl_3$) δ 144.6, 143.9, 141.33, 141.28, 129.6, 128.9, 128.8, 128.6, 128.49, 128.47, 127.4, 127.3, 126.5, 125.3, 57.1.

IR (KBr): 3056, 3027, 1597, 1495, 1449, 1077, 1030, 796, 756, 698, 607 cm^{-1}.

HRMS (EI) $[C_{25}H_{20}]$ $[M]^+$ calculated: 320.1565, found: 320.1577.

2-Benzhydrylbiphenyl (3ca)

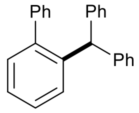

Compound **3ca** was prepared according to the general procedure **D** start from 2-fluorobiphenyl (34.4 mg, 0.2 mmol), and purified by silica gel column chromatography (*n*-hexane/DCM = 10/1) as a white solid (29.4 mg, yield: 46%).

^{1}H NMR (300 MHz, $CDCl_3$) δ 7.40 – 7.17 (m, 12H), 7.21 – 7.11 (m, 3H), 7.08 – 6.96 (m, 4H), 5.62 (s, 1H).

MS(EI): *m/z* 320 $[M]^+$.

The chemical shifts were consistent with those reported in the literature.[24]

1-Benzhydrylnaphthalene (3da)

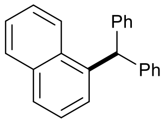

Compound **3da** was prepared according to the general procedure **D** start from 1-fluoronaphthalene (26.0 μL, 0.2 mmol), and purified by silica gel column chromatography (*n*-hexane/DCM = 10/1) as a white solid (44.1 mg, yield: 75%).

^{1}H NMR (300 MHz, $CDCl_3$) δ 8.04 (d, *J* = 7.6 Hz, 1H), 7.90 (dd, *J* = 7.5, 2.0 Hz, 1H), 7.79 (d, *J* = 8.2 Hz, 1H), 7.50 – 7.42 (m, 2H), 7.41 – 7.24 (m, 7H), 7.21 – 7.11 (m, 4H), 7.00 (d, *J* = 7.2 Hz, 1H), 6.33 (s, 1H).

MS(EI): *m/z* 274 $[M]^+$.

The chemical shifts were consistent with those reported in the literature.[25]

Triphenylmethane (3ea)

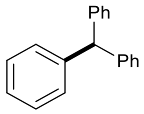

Compound **3ea** was prepared according to the general procedure **D** start from fluorobenzene (38.0 µL, 0.4 mmol), and purified by silica gel column chromatography (*n*-hexane) as a white solid (69.1 mg, yield: 71%).

^{1}H NMR (300 MHz, $CDCl_3$) δ 7.47 – 6.95 (m, 15H), 5.59 (s, 1H).

MS(EI): *m/z* 244 $[M]^+$.

The chemical shifts were consistent with those reported in the literature.[26]

1-Benzhydryl-4-methylbenzene (3fa)

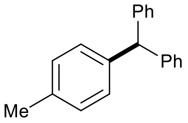

Compound **3fa** was prepared according to the general procedure **D** start from 4-fluorotoluene (44.0 µL, 0.4 mmol), and purified by silica gel column chromatography (*n*-hexane) as a white solid (64.5 mg, yield: 62%).

^{1}H NMR (300 MHz, $CDCl_3$) δ 7.38 – 7.20 (m, 6H), 7.20 – 7.08 (m, 6H), 7.08 – 7.00 (m, 2H), 5.55 (s, 1H), 2.35 (s, 3H).

MS(EI): *m/z* 258 $[M]^+$.

The chemical shifts were consistent with those reported in the literature.[27]

4-Benzhydrylanisole (3ga)

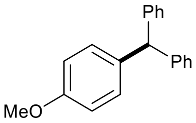

Compound **3ga** was prepared according to the general procedure **D** start from 4-fluoroanisole (45.0 µL, 0.4 mmol), and purified by silica gel column chromatography (*n*-hexane/DCM = 5/1) as a white solid (46.5 mg, yield: 42%).

^{1}H NMR (300 MHz, $CDCl_3$) δ 7.41 – 7.29 (m, 4H), 7.27 (dd, *J* = 6.9, 2.0 Hz, 2H), 7.20 – 7.14 (m, 4H), 7.12 – 7.03 (m, 2H), 6.88 (dd, *J* = 8.7, 2.0 Hz, 2H), 5.56 (s, 1H), 3.82 (s, 3H).

MS(EI): *m/z* 274 $[M]^+$.

The chemical shifts were consistent with those reported in the literature.[28]

3-Benzhydrylanisole (3ha)

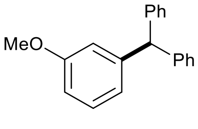

Compound **3ha** was prepared according to the general procedure **D** start from 3-fluoroanisole (45.5 µL, 0.4 mmol), and purified by silica gel column chromatography (*n*-hexane/DCM = 6/1) as a white solid (50.4 mg, yield: 46%).

^{1}H NMR (300 MHz, $CDCl_3$) δ 7.36 – 7.23 (m, 4H), 7.28 – 7.16 (m, 3H), 7.19 – 7.09 (m, 4H), 6.82 – 6.65 (m, 3H), 5.53 (s, 1H), 3.75 (s, 3H).

MS(EI): *m/z* 274 $[M]^+$.

The chemical shifts were consistent with those reported in the literature.[28]

1-(4-Benzhydrylphenyl)naphthalene (3ia)

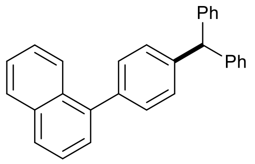

Compound **3ia** was prepared according to the general procedure **D** start from 1-(4-fluorophenyl)naphthalene (44.4 mg, 0.2 mmol), and purified by silica gel column chromatography (*n*-hexane/DCM = 8/1) as a white solid (42.3 mg, yield: 57%).

m.p. = 148.0 – 149.3 ^{o}C.

^{1}H NMR (500 MHz, $CDCl_3$) δ 8.00 (dd, *J* = 8.5, 1.1 Hz, 1H), 7.94 (dd, *J* = 8.2, 1.3 Hz, 1H), 7.89 (d, *J* = 8.1 Hz, 1H), 7.59 – 7.49 (m, 2H), 7.50 – 7.44 (m, 4H), 7.42 – 7.35 (m, 4H), 7.33 – 7.25 (m, 8H), 5.70 (s, 1H).

^{13}C NMR (126 MHz, $CDCl_3$) δ 144.0, 143.0, 140.1, 138.8, 133.9, 131.7, 130.1, 129.6, 129.4, 128.5, 128.4, 127.7, 127.1, 126.5, 126.2, 126.1, 125.9, 125.5, 56.8.

IR (KBr): 3057, 3026, 1599, 1496, 1450, 1395, 1109, 1033, 964, 842, 798, 781, 754, 703, 614 cm^{-1}.

HRMS (EI) $[C_{29}H_{22}]$ $[M]^+$ calculated: 370.1722, found: 370.1714.

4-Benzhydryl-4'-methylbiphenyl (3ja)

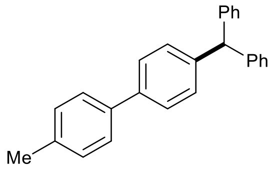

Compound **3ja** was prepared according to the general procedure **D** start from 4'-fluoro-4-methylbiphenyl (37.2 mg, 0.2 mmol), and purified by silica gel column chromatography (*n*-hexane/DCM = 10/1) as a white solid (48.9 mg, yield: 73%).

m.p. = 122.1 – 123.3 ^{o}C.

^{1}H NMR (500 MHz, $CDCl_3$) δ7.52 – 7.45 (m, 4H), 7.29 (dd, *J* = 8.1, 6.8 Hz, 4H), 7.24 – 7.19 (m, 4H), 7.18 – 7.13 (m, 6H), 5.58 (s, 1H), 2.37 (s, 3H).

^{13}C NMR (126 MHz, $CDCl_3$) δ 144.0, 142.8, 139.2, 138.1, 137.0, 129.9, 129.6, 128.5, 127.0, 126.95, 126.5, 56.7, 21.2.

IR (KBr): 3026, 2916, 1606, 1597, 1492, 1445, 1399, 1254, 1130, 1032, 800, 741, 702, 606 cm^{-1}.

HRMS (EI) $[C_{26}H_{22}]$ $[M]^+$ calculated: 334.1722, found: 334.1732.

4-Benzhydryl-4'-methoxybiphenyl (3ka)

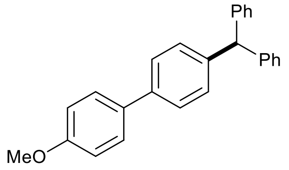

Compound **3ka** was prepared according to the general procedure **D** start from 4-fluoro-4'-methoxybiphenyl (40.5 mg, 0.2 mmol), and purified by silica gel column chromatography (*n*-hexane/DCM = 8/1) as a white solid (47.6 mg, yield: 68%).

m.p. = 112.4 – 114.1 °C.

^{1}H NMR (500 MHz, $CDCl_3$) δ 7.41 (d, *J* = 8.7 Hz, 2H), 7.38 (d, *J* = 8.3 Hz, 2H), 7.20 (t, *J* = 7.5 Hz, 4H), 7.16 – 7.09 (m, 2H), 7.10 – 7.04 (m, 6H), 6.86 (d, *J* = 8.8 Hz, 2H), 5.48 (s, 1H), 3.73 (s, 3H).

^{13}C NMR (126 MHz, $CDCl_3$) δ 159.2, 144.0, 142.5, 138.9, 133.5, 129.9, 129.6, 128.5, 128.1, 126.7, 126.5, 114.3, 56.6, 55.4.

IR (KBr): 3056, 3024, 2839, 1606, 1497, 1447, 1274, 1250, 1180, 1036, 826, 806, 757, 743, 700, 605 cm^{-1}.

HRMS (EI) [$C_{26}H_{22}O$] $[M]^+$ calculated: 350.1671, found: 350.1661.

4-Benzhydryl-4'-(benzyloxy)-biphenyl (3la)

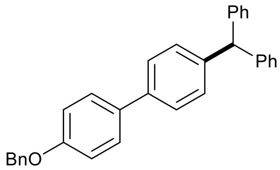

Compound **3la** was prepared according to the general procedure **D** start from 4-(benzyloxy)-4'-fluorobiphenyl (55.6 mg, 0.2 mmol), and purified by silica gel column chromatography (*n*-hexane/DCM = 6/1) as a white solid (34.1 mg, yield: 40%).

m.p. = 143.7 – 144.5 °C.

^{1}H NMR (500 MHz, $CDCl_3$) δ 7.56 (d, *J* = 8.7 Hz, 2H), 7.54 – 7.48 (m, 4H), 7.44 (t, *J* = 7.6 Hz, 2H), 7.40 – 7.32 (m, 5H), 7.28 (d, *J* = 6.1 Hz, 2H), 7.24 – 7.18 (m, 6H), 7.08 (d, *J* = 8.8 Hz, 2H), 5.63 (s, 1H), 5.15 (s, 2H).

^{13}C NMR (126 MHz, $CDCl_3$) δ 158.4, 144.0, 142.6, 138.9, 137.1, 133.8, 129.9, 129.6, 128.8, 128.5, 128.2, 128.1, 127.6, 126.7, 126.5, 115.2, 70.2, 56.6.

IR (KBr): 3060, 3028, 2881, 1606, 1497, 1450, 1384, 1273, 1250, 1176, 1042, 843, 807, 762, 743, 713, 606 cm^{-1}.

HRMS (EI) [$C_{32}H_{26}O$] $[M]^+$ calculated: 426.1984, found: 426.1971.

4-Benzhydryl-4'-(trifluoromethyl)biphenyl (3ma)

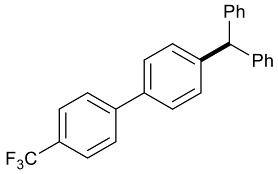

Compound **3ma** was prepared according to the general procedure **D** start from 4-fluoro-4'-(trifluoromethyl)biphenyl (38.0 µL, 0.2 mmol), and purified by silica gel column chromatography (*n*-hexane) as a light-yellow oil (34.9 mg, yield: 45%).

^{1}H NMR (300 MHz, $CDCl_3$) δ 7.67 (s, 4H), 7.52 (d, *J* = 8.2 Hz, 2H), 7.35 – 7.27 (m, 4H), 7.28 – 7.18 (m, 4H), 7.15 (d, *J* = 7.2 Hz, 4H), 5.60 (s, 1H).

^{13}C NMR (176 MHz, $CDCl_3$) δ 144.5, 144.3, 143.7, 137.8, 130.2, 129.6, 128.6, 127.4, 127.3, 126.6, 125.8 (q, *J* = 3.9 Hz), 124.5 (q, *J* = 271.7 Hz), 56.7.

^{19}F NMR (282 MHz, $CDCl_3$) δ -62.88 (s, 3F).

IR (KBr): 3056, 2974, 2912, 1617, 1495, 1326, 1166, 1126, 1071, 1007, 835, 700 cm^{-1}.

HRMS (EI) [$C_{26}H_{19}F_3$] $[M]^+$ calculated: 388.1439, found: 388.1474.

5-(4-Benzhydrylphenyl)benzo[*d*][1,3]dioxole (3na)

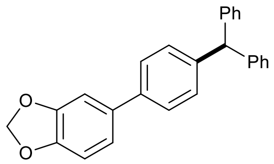

Compound **3na** was prepared according to the general procedure **D** start from 5-(4-fluorophenyl)benzo[*d*][1,3]dioxole (43.2 mg, 0.2 mmol), and purified by silica gel column chromatography (*n*-hexane/DCM = 5/1) as a white solid (34.3 mg, yield: 47%).

m.p. = 127.4 – 128.2 ^{o}C.

^{1}H NMR (500 MHz, $CDCl_3$) δ 7.44 (d, *J* = 8.2 Hz, 2H), 7.34 – 7.29 (m, 4H), 7.26 – 7.22 (m, 2H), 7.18 – 7.15 (m, 6H), 7.08 – 7.04 (m, 2H), 6.87 (d, *J* = 7.9 Hz, 1H), 5.99 (s, 2H), 5.59 (s, 1H).

^{13}C NMR (126 MHz, $CDCl_3$) δ 148.2, 147.1, 144.0, 142.8, 139.0, 135.4, 129.9, 129.6, 128.5, 126.9, 126.5, 120.6, 108.7, 107.7, 101.2, 56.6.

IR (KBr): 3056, 3026, 2877, 1599, 1501, 1481, 1341, 1265, 1225, 1040, 800, 738, 701, 625 cm^{-1}.

HRMS (EI) [$C_{26}H_{20}O_2$] $[M]^+$ calculated: 364.1463, found: 364.1474.

1-(4-Benzhydrylphenyl)-1*H*-pyrrole (3oa)

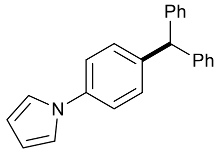

Compound **3oa** was prepared according to the general procedure **D** start from 1-(4-fluorophenyl)-1*H*-pyrrole (32.2 mg, 0.2 mmol), and purified by silica gel column chromatography (*n*-hexane/DCM = 5/1) as a white solid (53.2 mg, yield: 86%).

^{1}H NMR (500 MHz, $CDCl_3$) δ 7.38 – 7.32 (m, 6H), 7.30 – 7.26 (m, 2H), 7.23 – 7.14 (m, 6H), 7.10 (t, *J* = 2.2 Hz, 2H), 6.37 (t, *J* = 2.2 Hz, 2H), 5.61 (s, 1H).

IR (KBr): 3068, 3039, 3022, 2871, 1611, 1517, 1485, 1324, 1119, 1068, 1033, 1020, 758, 725, 704, 606 cm^{-1}.

MS(EI): *m/z* 309 $[M]^+$.

The chemical shifts were consistent with those reported in the literature.[25]

2-(4-Benzhydrylphenyl)-1-methyl-1*H*-indole (3pa)

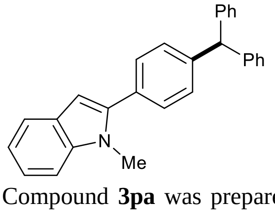

Compound **3pa** was prepared according to the general procedure **D** start from 2-(4-fluorophenyl)-1-methyl-1*H*-indole (45.0 mg, 0.2 mmol), and purified by silica gel column chromatography (*n*-hexane/DCM = 8/1) as a white solid (54.5 mg, yield: 73%).

m.p. = 168.3 – 169.4 ^{o}C.

^{1}H NMR (500 MHz, $CDCl_3$) δ 7.65 (d, *J* = 7.9 Hz, 1H), 7.49 – 7.43 (m, 2H), 7.41 – 7.31 (m, 5H), 7.31 – 7.22 (m, 5H), 7.24 – 7.18 (m, 4H), 7.20 – 7.13 (m, 1H), 6.58 (d, *J* = 0.9 Hz, 1H), 5.64 (s, 1H), 3.77 (s, 3H).

^{13}C NMR (126 MHz, $CDCl_3$) δ 143.8, 143.78, 141.5, 138.5, 130.9, 129.7, 129.6, 129.4, 128.6, 128.1, 126.6, 121.7, 120.6, 120.0, 109.7, 101.7, 56.8, 31.4.

IR (KBr): 3057, 3023, 2935, 1599, 1493, 1465, 1432, 1337, 1316, 1110, 1032, 842, 777, 749, 702 cm^{-1}.

HRMS (EI) [$C_{28}H_{23}N$] $[M]^+$ calculated: 373.1830, found: 373.1841.

2-(4-Benzhydrylphenyl)benzofuran (3qa)

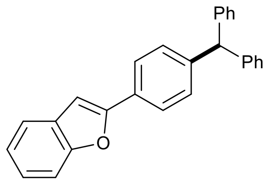

Compound **3qa** was prepared according to the general procedure **D** start from 2-(4-fluorophenyl)benzofuran (42.4 mg, 0.2 mmol), and purified by silica gel column chromatography (*n*-hexane/DCM = 10/1) as a white solid (28.2 mg, yield: 39%).

m.p. = 147.7 – 148.7 ^oC.

^{1}H NMR (500 MHz, $CDCl_3$) δ 7.79 (d, *J* = 8.0 Hz, 2H), 7.57 (d, *J* = 7.5 Hz, 1H), 7.52 (d, *J* = 8.1 Hz, 1H), 7.32 (t, *J* = 7.5 Hz, 4H), 7.28 – 7.20 (m, 6H), 7.16 (d, *J* = 7.6 Hz, 4H), 6.99 (s, 1H), 5.60 (s, 1H).

^{13}C NMR (126 MHz, $CDCl_3$) δ 156.0, 155.0, 144.7, 143.7, 130.0, 129.6, 129.4, 128.7, 128.6, 126.6, 125.1, 124.3, 123.0, 121.0, 111.3, 101.2, 56.8.

IR (KBr): 3080, 3024, 2854, 1598, 1493, 1452, 1257, 1172, 1033, 796, 738, 699, 603 cm^{-1}.

HRMS (EI) [$C_{27}H_{20}O$] $[M]^+$ calculated: 360.1514, found: 360.1516.

4-((4-Methoxyphenyl)(phenyl)methyl)biphenyl (3ab)

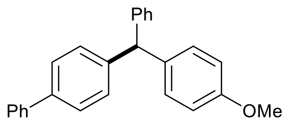

Compound **3ab** was prepared according to the general procedure **D** start from 4-fluorobiphenyl (34.4 mg, 0.2 mmol), and purified by silica gel column chromatography (*n*-hexane/DCM = 6/1) as a white solid (53.9 mg, yield: 77%).

^{1}H NMR (300 MHz, $CDCl_3$) δ 7.59 (dd, *J* = 18.9, 7.6 Hz, 4H), 7.46 (t, *J* = 7.4 Hz, 2H), 7.40 – 7.02 (m, 10H), 6.89 (d, *J* = 8.2 Hz, 2H), 5.59 (s, 1H), 3.82 (s, 3H).

MS(EI): *m/z* 350 $[M]^+$.

The chemical shifts were consistent with those reported in the literature.[29]

4-(Bis(4-methoxyphenyl)methyl)biphenyl (3ac)

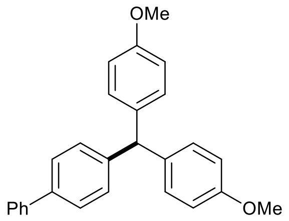

Compound **3ac** prepared according to the general procedure **D** start from 4-fluorobiphenyl (34.4 mg, 0.2 mmol), and purified by silica gel column chromatography (*n*-hexane/DCM = 5/1) as a white solid (39.6 mg, yield: 52%).

m.p. = 108.4 – 110.3 ^oC.

^{1}H NMR (500 MHz, $CDCl_3$) δ 7.61 (dd, *J* = 8.3, 1.3 Hz, 2H), 7.54 (d, *J* = 8.3 Hz, 2H), 7.48 – 7.42 (m, 2H), 7.39 – 7.32 (m, 1H), 7.21 (d, *J* = 8.1 Hz, 2H), 7.14 – 7.05 (m, 4H), 6.92 – 6.83 (m, 4H), 5.52 (s, 1H), 3.81 (s, 6H).

^{13}C NMR (126 MHz, $CDCl_3$) δ 158.1, 143.9, 141.0, 139.1, 136.5, 130.4, 129.8, 128.8, 127.2, 127.1, 127.1, 113.8, 55.4, 55.0.

IR (KBr): 3056, 3028, 2834, 1607, 1582, 1505, 1487, 1303, 1241, 1175, 1031, 824, 806, 739, 698, 580 cm^{-1}.

HRMS (EI) [$C_{27}H_{24}O_2$] $[M]^+$ calculated: 380.1776, found: 380.1759.

4-(Di-*p*-tolylmethyl)biphenyl (3ad)

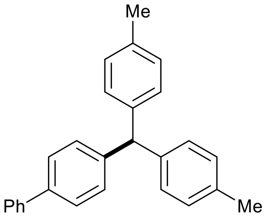

Compound **3ad** was prepared according to the general procedure **D** start from 4-fluorobiphenyl (34.4 mg, 0.2 mmol), and purified by silica gel column chromatography (*n*-hexane/DCM = 10/1) as a white solid (49.4 mg, yield: 71%).

m.p. = 117.3 – 118.5 ^{o}C.

^{1}H NMR (500 MHz, $CDCl_3$) δ 7.63 – 7.57 (m, 2H), 7.56 – 7.50 (m, 2H), 7.44 (t, *J* = 7.7 Hz, 2H), 7.38 – 7.31 (m, 1H), 7.21 (d, *J* = 8.2 Hz, 2H), 7.14 (d, *J* = 7.9 Hz, 4H), 7.07 (d, *J* = 8.2 Hz, 4H), 5.54 (s, 1H), 2.36 (s, 6H).

^{13}C NMR (126 MHz, $CDCl_3$) δ 143.6, 141.2, 141.1, 139.1, 135.9, 129.9, 129.4, 129.2, 128.8, 127.2, 127.15, 127.1, 55.9, 21.2.

IR (KBr): 3049, 3026, 2918, 1599, 1510, 1487, 1447, 1021, 809, 755, 739, 697, 575 cm^{-1}.

HRMS (EI) [$C_{27}H_{24}$] $[M]^+$ calculated: 348.1878, found: 348.1886.

4-(Phenyl(*o*-tolyl)methyl)biphenyl (3ae)

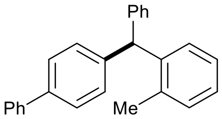

Compound **3ae** was prepared according to the general procedure **D** start from 4-fluorobiphenyl (34.4 mg, 0.2 mmol), and purified by silica gel column chromatography (*n*-hexane/DCM = 10/1) as a white solid (38.0 mg, yield: 57%).

m.p. = 112.8 – 114.2 ^{o}C.

^{1}H NMR (500 MHz, $CDCl_3$) δ 7.57 (dd, *J* = 8.2, 1.3 Hz, 2H), 7.50 (d, *J* = 8.3 Hz, 2H), 7.44 – 7.37 (m, 2H), 7.35 – 7.25 (m, 3H), 7.25 – 7.19 (m, 1H), 7.20 – 7.06 (m, 7H), 6.86 (dd, *J* = 7.5, 1.5 Hz, 1H), 5.70 (s, 1H), 2.24 (s, 3H).

^{13}C NMR (126 MHz, $CDCl_3$) δ 143.5, 142.7, 142.4, 141.0, 139.2, 136.8, 130.6, 130.1, 129.8, 129.6, 128.9, 128.5, 127.3, 127.1, 126.6, 126.5, 125.9, 53.3, 20.1.

IR (KBr): 3059, 3026, 1599, 1487, 1450, 1380, 1289, 1076, 1029, 1009, 812, 760, 736, 655, 610 cm^{-1}.

HRMS (EI) [$C_{26}H_{22}$] $[M]^+$ calculated: 334.1722, found: 334.1718.

4-((4-Chlorophenyl)(phenyl)methyl)biphenyl (3af)

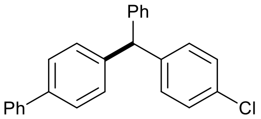

Compound **3af** was prepared according to the general procedure **D** start from 4-fluorobiphenyl (34.4 mg, 0.2 mmol), and purified by silica gel column chromatography (*n*-hexane/DCM = 10/1) as a white solid (43.2 mg, yield: 61%).

m.p. = 98.9 – 100.3 ^{o}C.

^{1}H NMR (500 MHz, $CDCl_3$) δ 7.56 (dd, *J* = 8.3, 1.3 Hz, 2H), 7.53 – 7.49 (m, 2H), 7.41 (dd, *J* = 8.4, 7.0 Hz, 2H), 7.34 – 7.28 (m, 3H), 7.26 (d, *J* = 8.5 Hz, 2H), 7.24 – 7.20 (m, 1H), 7.17 – 7.10 (m, 4H), 7.10 – 7.05 (m, 2H), 5.54 (s, 1H).

^{13}C NMR (126 MHz, $CDCl_3$) δ 143.4, 142.6, 142.5, 140.8, 139.5, 132.4, 130.9, 129.9, 129.5, 128.9, 128.6, 127.4, 127.3, 127.1, 126.7, 56.0.

IR (KBr): 3059, 3026, 1599, 1487, 1450, 1380, 1289, 1076, 1029, 1009, 812, 760, 736, 655, 610 cm^{-1}.

HRMS (EI) [$C_{25}H_{19}Cl$] $[M]^+$ calculated: 354.1175, found: 354.1181.

2-(Biphenyl-4-yl(phenyl)methyl)thiophene (3ag)

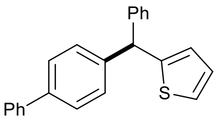

Compound **3ag** was prepared according to the general procedure **D** start from 4-fluorobiphenyl (34.4 mg, 0.2 mmol), and purified by silica gel column chromatography (*n*-hexane) as a white solid (54.5 mg, yield: 84%).

m.p. = 99.2 – 99.8 ^{o}C.

^{1}H NMR (500 MHz, $CDCl_3$) δ 7.56 (dd, *J* = 8.3, 1.3 Hz, 2H), 7.54 – 7.50 (m, 2H), 7.43 – 7.37 (m, 2H), 7.34 – 7.21 (m, 8H), 7.22 – 7.19 (m, 1H), 6.93 (dd, *J* = 5.2, 3.5 Hz, 1H), 6.72 (dt, *J* = 3.5, 1.2 Hz, 1H), 5.71 (s, 1H).

^{13}C NMR (126 MHz, $CDCl_3$) δ 147.9, 143.9, 143.0, 140.9, 139.7, 129.3, 129.0, 128.9, 128.6, 127.3, 127.2, 127.2, 126.9, 126.8, 126.6, 124.7, 51.9.

IR (KBr): 3060, 3028, 1601, 1486, 1452, 1408, 1265, 1113, 846, 762, 740, 698, 623 cm^{-1}.

HRMS (EI) [$C_{23}H_{18}S$] $[M]^+$ calculated: 326.1129, found: 326.1130.

4-((4-Methoxyphenyl)(phenyl)methyl)-4'-methylbiphenyl (3jb)

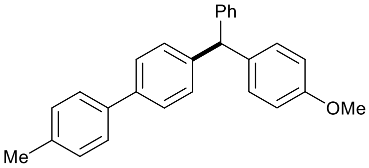

Compound **3jb** was prepared according to the general procedure **D** start from 4'-fluoro-4-methylbiphenyl (37.2 mg, 0.2 mmol), and purified by silica gel column chromatography (*n*-hexane/DCM = 5/1) as a light-yellow oil (39.7 mg, yield: 55%).

^{1}H NMR (500 MHz, $CDCl_3$) δ 7.54 (dd, *J* = 10.3, 7.9 Hz, 4H), 7.35 (t, *J* = 7.5 Hz, 2H), 7.28 (d, *J* = 8.1 Hz, 3H), 7.25 – 7.19 (m, 4H), 7.13 (d, *J* = 8.5 Hz, 2H), 6.90 (d, *J* = 8.7 Hz, 2H), 5.59 (s, 1H), 3.84 (s, 3H), 2.44 (s, 3H).

^{13}C NMR (126 MHz, $CDCl_3$) δ 158.2, 144.4, 143.2, 139.1, 138.1, 137.0, 136.2, 130.5, 129.8, 129.6, 129.5, 128.5, 127.0, 126.9, 126.4, 113.8, 55.8, 55.4, 21.2.

IR (KBr): 3051, 3025, 2835, 1608, 1581, 1505, 1450, 1302, 1244, 1176, 1031, 807, 739, 699, 583 cm^{-1}.

HRMS (EI) [$C_{27}H_{24}O$] $[M]^+$ calculated: 364.1827, found: 364.1833.

4-(Bis(4-methoxyphenyl)methyl)-4'-methylbiphenyl (3jc)

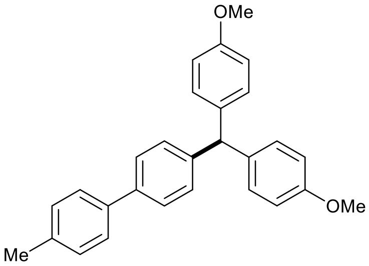

Compound **3jc** was prepared according to the general procedure **D** start from 4'-fluoro-4-methylbiphenyl (37.2 mg, 0.2 mmol), and purified by silica gel column chromatography (*n*-hexane/DCM = 5/1) as a light-yellow oil (51.1 mg, yield: 65%).

^{1}H NMR (300 MHz, $CDCl_3$) δ 7.53 – 7.44 (m, 4H), 7.22 (d, *J* = 7.3 Hz, 2H), 7.15 (d, *J* = 7.9 Hz, 2H), 7.05 (d, *J* = 8.5 Hz, 4H), 6.83 (d, *J* = 8.7 Hz, 4H), 5.48 (s, 1H), 3.78 (s, 6H), 2.38 (s, 3H).

^{13}C NMR (75 MHz, $CDCl_3$) δ 158.1, 143.5, 139.0, 138.1, 137.0, 136.5, 130.4, 129.8, 129.6, 127.0, 126.9, 113.8, 55.4, 55.0, 21.2.

IR (KBr): 3025, 2952, 2834, 1609, 1582, 1505, 1463, 1301, 1253, 1177, 1037, 809, 577 cm^{-1}.

HRMS (EI) [$C_{28}H_{26}O_2$] [M]$^+$ calculated: 394.1933, found: 394.1939.

2-((4'-Methyl-biphenyl]-4-yl)(phenyl)methyl)thiophene (3jg)

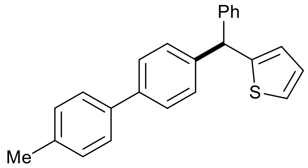

Compound **3jg** was prepared according to the general procedure **D** start from 4'-fluoro-4-methylbiphenyl (37.2 mg, 0.2 mmol), and purified by silica gel column chromatography (*n*-hexane) as a white solid (50.2 mg, yield: 74%).

m.p. = 96.5 – 97.0 ^{o}C.

^{1}H NMR (500 MHz, $CDCl_3$) δ 7.53 – 7.48 (m, 2H), 7.46 (d, *J* = 8.2 Hz, 2H), 7.32 – 7.28 (m, 2H), 7.28 – 7.17 (m, 8H), 6.93 (dd, *J* = 5.2, 3.5 Hz, 1H), 6.72 (dt, *J* = 3.5, 1.2 Hz, 1H), 5.70 (s, 1H), 2.37 (s, 3H).

^{13}C NMR (126 MHz, $CDCl_3$) δ 148.0, 143.9, 142.7, 139.6, 138.0, 137.1, 129.6, 129.3, 129.0, 128.6, 127.03, 127.0, 126.9, 126.7, 126.5, 124.7, 51.9, 21.2.

IR (KBr): 3056, 3025, 2918, 1599, 1496, 1451, 1264, 1229, 1006, 858, 808, 736, 700, 600 cm^{-1}.

HRMS (EI) [$C_{24}H_{20}S$] [M]$^+$ calculated: 340.1286, found: 340.1300.

4-Methoxy-4'-((4-methoxyphenyl)(phenyl)methyl)biphenyl (3kb)

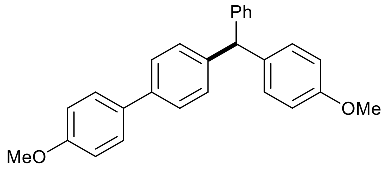

Compound **3kb** was prepared according to the general procedure **D** start from 4-fluoro-4'-methoxybiphenyl (40.5 mg, 0.2 mmol), and purified by silica gel column chromatography (*n*-hexane/DCM = 5/1) as a light yellow solid (50.1 mg, yield: 66%).

m.p. = 111.9 – 112.1 ^{o}C.

^{1}H NMR (500 MHz, $CDCl_3$) δ 7.53 (d, *J* = 8.8 Hz, 2H), 7.48 (d, *J* = 8.3 Hz, 2H), 7.35 – 7.28 (m, 2H), 7.23 (t, *J* = 7.3 Hz, 1H), 7.17 (d, *J* = 8.3 Hz, 4H), 7.08 (d, *J* = 8.7 Hz, 2H), 6.98 (d, *J* = 8.7 Hz, 2H), 6.86 (d, *J* = 8.7 Hz, 2H), 5.54 (s, 1H), 3.85 (s, 3H), 3.80 (s, 3H).

^{13}C NMR (126 MHz, $CDCl_3$) δ 159.2, 158.2, 144.4, 142.9, 138.8, 136.2, 133.6, 130.5, 130.0, 129.5, 128.4, 128.1, 126.7, 126.4, 114.3, 113.8, 55.8, 55.5, 55.4.

IR (KBr): 3084, 3028, 2928, 2873, 1609, 1510, 1464, 1251, 1207, 1178, 1038, 1000, 837, 698, 621 cm^{-1}.

HRMS (EI) [$C_{27}H_{24}O_2$] [M]$^+$ calculated: 380.1776, found: 380.1783.

4-(Bis(4-methoxyphenyl)methyl)-4'-methoxybiphenyl (3kc)

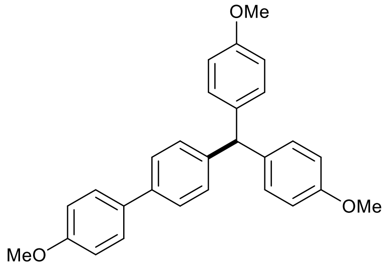

Compound **3kc** was prepared according to the general procedure **D** start from 4-fluoro-4'-methoxybiphenyl (40.5 mg, 0.2 mmol), and purified by silica gel column chromatography (*n*-hexane/DCM = 4/1) as a light-yellow oil (70.2 mg, yield: 86%).

^{1}H NMR (500 MHz, $CDCl_3$) δ 7.57 – 7.49 (m, 2H), 7.50 – 7.43 (m, 2H), 7.16 (d, *J* = 8.2 Hz, 2H), 7.10 – 7.04 (m, 4H), 7.00 – 6.93 (m, 2H), 6.89 – 6.80 (m, 4H), 5.49 (s, 1H), 3.85 (s, 3H), 3.80 (s, 6H).

^{13}C NMR (126 MHz, $CDCl_3$) δ 159.1, 158.1, 143.2, 138.7, 136.6, 133.6, 130.4, 129.8, 128.1, 126.7, 114.3, 113.8, 55.5, 55.4, 55.0.

IR (KBr): 3068, 3026, 2834, 1614, 1506, 1259, 1179, 1112, 1040, 823, 774, 737 cm^{-1}.

HRMS (EI) [$C_{28}H_{26}O_3$] $[M]^+$ calculated: 410.1882, found: 410.1897.

4-(Di-*p*-tolylmethyl)-4'-methoxybiphenyl (3kd)

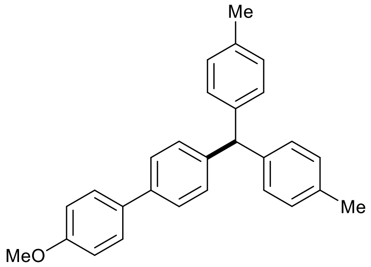

Compound **3kd** was prepared according to the general procedure **D** start from 4-fluoro-4'-methoxybiphenyl (40.5 mg, 0.2 mmol), and purified by silica gel column chromatography (*n*-hexane/DCM = 6/1) as a light-yellow oil (58.7 mg, yield: 78%).

^{1}H NMR (500 MHz, $CDCl_3$) δ 7.56 (d, *J* = 8.8 Hz, 2H), 7.52 (d, *J* = 8.4 Hz, 2H), 7.22 (d, *J* = 8.2 Hz, 2H), 7.16 (d, *J* = 7.9 Hz, 4H), 7.10 (d, *J* = 8.2 Hz, 4H), 7.01 (d, *J* = 8.8 Hz, 2H), 5.56 (s, 1H), 3.88 (s, 3H), 2.38 (s, 6H).

^{13}C NMR (126 MHz, $CDCl_3$) δ 159.1, 142.9, 141.3, 138.7, 135.9, 133.6, 129.8, 129.4, 129.1, 128.1, 126.6, 114.3, 55.9, 55.4, 21.2.

IR (KBr): 3088, 3028, 3005, 2917, 2833, 1608, 1510, 1496, 1274, 1249, 1180, 1037, 1020, 838, 814, 776, 723 cm^{-1}.

HRMS (EI) [$C_{28}H_{26}O$] $[M]^+$ calculated: 378.1984, found: 378.1991.

4-Methoxy-4'-(phenyl(*o*-tolyl)methyl)biphenyl (3ke)

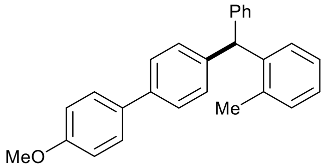

Compound **3ke** was prepared according to the general procedure **D** start from 4-fluoro-4'-methoxybiphenyl (40.5 mg, 0.2 mmol), and purified by silica gel column chromatography (*n*-hexane/DCM = 10/1) as a white solid (24.0 mg, yield: 33%).

m.p. = 103.1 – 103.8 °C.

^{1}H NMR (500 MHz, $CDCl_3$) δ 7.53 (d, *J* = 8.9 Hz, 2H), 7.50 – 7.46 (m, 2H), 7.31 (dd, *J* = 8.1, 6.7 Hz, 2H), 7.27 – 7.21 (m, 1H), 7.21 – 7.16 (m, 2H), 7.16 – 7.09 (m, 5H), 7.01 – 6.95 (m, 2H), 6.89 (dd, *J* = 7.5, 1.6 Hz, 1H), 5.72 (s, 1H), 3.85 (s, 3H), 2.26 (s, 3H).

^{13}C NMR (126 MHz, $CDCl_3$) δ 159.2, 143.5, 142.5, 143.0, 138.8, 136.8, 133.5, 130.6, 130.1, 129.7, 129.6, 128.5, 128.1, 126.7, 126.5, 126.4, 125.9, 114.3, 55.5, 53.3, 20.1.

IR (KBr): 3060, 3023, 2960, 2839, 1606, 1496, 1462, 1401, 1254, 1182, 1120, 1036, 814, 744, 700 cm^{-1}.

HRMS (EI) [$C_{27}H_{24}O$] $[M]^+$ calculated: 364.1827, found: 364.1844.

4-((4-Chlorophenyl)(phenyl)methyl)-4'-methoxybiphenyl (3kf)

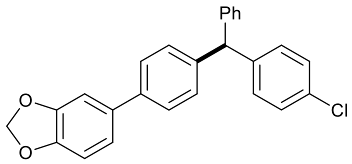

Compound **3kf** was prepared according to the general procedure **D** start from 5-(4-fluorophenyl)benzo[*d*][1,3]dioxole (43.2 mg, 0.2 mmol), and purified by silica gel column chromatography (*n*-hexane/DCM = 7/1) as a white solid (43.8 mg, yield: 57%).

m.p. = 82.6 – 83.6 °C.

^{1}H NMR (500 MHz, $CDCl_3$) δ 7.49 (d, *J* = 8.7 Hz, 2H), 7.46 (d, *J* = 8.1 Hz, 2H), 7.32 – 7.19 (m, 5H), 7.12 (d, *J* = 8.4 Hz, 4H), 7.07 (d, *J* = 8.3 Hz, 2H), 6.95 (d, *J* = 8.8 Hz, 2H), 5.52 (s, 1H), 3.81 (s, 3H).

^{13}C NMR (126 MHz, $CDCl_3$) δ 159.2, 143.5, 142.6, 141.9, 139.1, 133.4, 132.3, 130.9, 129.8, 129.5, 128.6, 128.1, 126.8, 126.7, 114.3, 56.0, 55.4.

IR (KBr): 3057, 2997, 2932, 2840, 1608, 1527, 1459, 1325, 1250, 1180, 1115, 1037, 838, 769, 701, 631 cm^{-1}.

HRMS (EI) [$C_{26}H_{21}ClO$] $[M]^+$ calculated: 384.1281, found: 384.1286.

5-(4-((4-Methoxyphenyl)(phenyl)methyl)phenyl)benzo[*d*][1,3]dioxole (3nb)

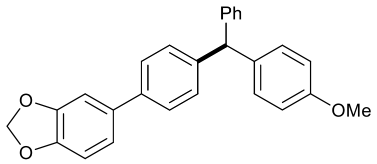

Compound **3nb** was prepared according to the general procedure **D** start from 5-(4-fluorophenyl)benzo[*d*][1,3]dioxole (43.2 mg, 0.2 mmol), and purified by silica gel column chromatography (*n*-hexane/EtOAc = 20/1) as a light-yellow solid (53.6 mg, yield: 68%).

m.p. = 96.1 – 97.4 °C.

^{1}H NMR (500 MHz, $CDCl_3$) δ 7.49 – 7.43 (m, 2H), 7.35 – 7.30 (m, 2H), 7.28 – 7.22 (m, 1H), 7.21 – 7.15 (m, 4H), 7.12 – 7.05 (m, 4H), 6.91 – 6.85 (m, 3H), 6.00 (s, 2H), 5.55 (s, 1H), 3.81 (s, 3H).

^{13}C NMR (126 MHz, $CDCl_3$) δ 158.2, 148.2, 147.1, 144.3, 143.2, 138.9, 136.1, 135.4, 130.5, 129.8, 129.5, 128.4, 126.8, 126.4, 120.6, 113.8, 108.7, 107.7, 101.2, 55.8, 55.3.

IR (KBr): 3027, 2952, 2907, 2835, 1610, 1481, 1414, 1250, 1111, 1040, 935, 842, 701, 568 cm^{-1}.

HRMS (EI) [$C_{27}H_{22}O_3$] $[M]^+$ calculated: 394.1569, found: 394.1581.

5-(4-(Bis(4-methoxyphenyl)methyl)phenyl)benzo[*d*][1,3]dioxole (3nc)

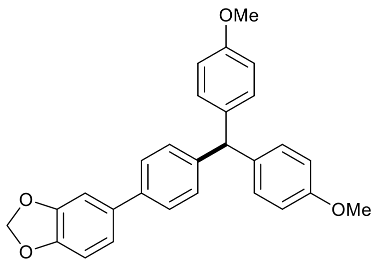

Compound **3nc** was prepared according to the general procedure **D** start from 5-(4-fluorophenyl)benzo[*d*][1,3]dioxole (43.2 mg, 0.2 mmol), and purified by silica gel column chromatography (*n*-hexane/DCM = 4/1) as a light-yellow solid (47.5 mg, yield: 56%).

m.p. = 136.1 – 136.8 °C.

^{1}**H NMR** (300 MHz, $CDCl_3$) δ 7.43 (d, *J* = 7.8 Hz, 2H), 7.15 (d, *J* = 7.8 Hz, 2H), 7.09 – 7.01 (m, 6H), 6.93 – 6.75 (m, 5H), 5.99 (s, 2H), 5.48 (s, 1H), 3.80 (s, 6H).

^{13}**C NMR** (75 MHz, $CDCl_3$) δ 158.1, 148.2, 147.0, 143.5, 138.8, 136.5, 135.4, 130.4, 129.8, 126.8, 120.6, 113.8, 108.7, 107.6, 101.2, 55.4, 54.9.

IR (KBr): 3008, 2967, 2932, 2892, 2834, 1609, 1509, 1481, 1438, 1293, 1245, 1175, 1028, 929, 802, 771 cm^{-1}.

HRMS (EI) [$C_{28}H_{24}O_4$] $[M]^+$ calculated: 424.1765, found: 424.1761.

(4-((4-Methoxyphenyl)(phenyl)methyl)phenyl)-1*H*-pyrrole (3ob)

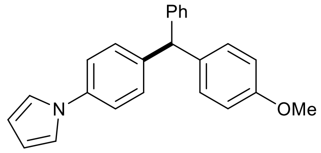

Compound **3ob** was prepared according to the general procedure **D** start from 1-(4-fluorophenyl)-1*H*-pyrrole (32.2 mg, 0.2 mmol), and purified by silica gel column chromatography (*n*-hexane/EtOAc = 40/1) as a light-yellow oil (42.0 mg, yield: 62%).

^{1}**H NMR** (500 MHz, $CDCl_3$) δ 7.38 – 7.32 (m, 4H), 7.28 (d, *J* = 8.0 Hz, 1H), 7.23 – 7.16 (m, 4H), 7.14 – 7.06 (m, 4H), 6.90 (d, *J* = 8.7 Hz, 2H), 6.38 (t, *J* = 2.2 Hz, 2H), 5.57 (s, 1H), 3.84 (s, 3H).

^{13}**C NMR** (126 MHz, $CDCl_3$) δ 158.3, 144.1, 141.9, 139.1, 135.9, 130.5, 130.4, 129.4, 128.5, 126.5, 120.5, 119.4, 113.9, 110.4, 55.5, 55.4.

IR (KBr): 3058, 2905, 2833, 1611, 1510, 1483, 1330, 1248, 1176, 1070, 1035, 923, 823, 725, 572 cm^{-1}.

HRMS (EI) [$C_{24}H_{21}NO$] $[M]^+$ calculated: 339.1623, found: 339.1635.

1-(4-(Phenyl(thiophen-2-yl)methyl)phenyl)-1*H*-pyrrole (3og)

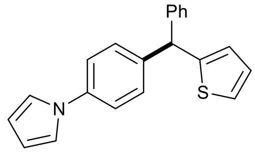

Compound **3og** was prepared according to the general procedure **D** start from 1-(4-fluorophenyl)-1*H*-pyrrole (32.2 mg, 0.2 mmol), and purified by silica gel column chromatography (*n*-hexane/DCM = 20/1) as a white solid (50.4 mg, yield: 80%).

m.p. = 90.1 – 90.9 ^{o}C.

^{1}**H NMR** (300 MHz, $CDCl_3$) δ 7.38 – 7.15 (m, 10H), 7.06 (s, 2H), 6.95 (t, *J* = 4.4 Hz, 1H), 6.71 (d, *J* = 3.4 Hz, 1H), 6.33 (s, 2H), 5.69 (s, 1H).

^{13}**C NMR** (126 MHz, $CDCl_3$) δ 147.7, 143.6, 141.4, 139.5, 130.0, 128.9, 128.6, 127.0, 126.8, 126.6, 124.8, 120.5, 119.4, 110.5, 51.6.

IR (KBr): 3061, 2908, 2830, 1611, 1519, 1474, 1427, 1325, 1227, 1120, 1066, 1019, 923, 855, 794, 702, 617 cm^{-1}.

HRMS (EI) [$C_{21}H_{17}NS$] $[M]^+$ calculated: 315.1082, found: 315.1082.

2-(4-((4-Methoxyphenyl)(phenyl)methyl)phenyl)-1-methyl-1*H*-indole (3pb)

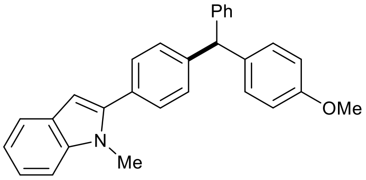

Compound **3pb** was prepared according to the general procedure **D** start from 2-(4-fluorophenyl)-1-methyl-1*H*-indole (45.0 mg, 0.2 mmol), and purified by silica gel column chromatography (*n*-hexane/DCM = 10/1) as a white solid (36.2 mg, yield: 45%).

m.p. = 157.9 – 159.2 °C.

^{1}H NMR (500 MHz, $CDCl_3$) δ 7.66 (d, *J* = 7.8 Hz, 1H), 7.46 (d, *J* = 8.3 Hz, 2H), 7.40 – 7.35 (m, 1H), 7.36 – 7.32 (m, 2H), 7.29 – 7.26 (m, 2H), 7.25 (d, *J* = 8.2 Hz, 2H), 7.22 – 7.19 (m, 2H), 7.19 – 7.15 (m, 1H), 7.12 (d, *J* = 8.7 Hz, 2H), 6.90 (d, *J* = 8.7 Hz, 2H), 6.58 (s, 1H), 5.59 (s, 1H), 3.83 (s, 3H), 3.77 (s, 3H).

^{13}C NMR (126 MHz, $CDCl_3$) δ 158.3, 144.2, 144.1, 141.5, 138.5, 135.9, 130.8, 130.5, 129.6, 129.5, 129.3, 128.5, 128.1, 126.5, 121.7, 120.5, 119.9, 113.9, 109.7, 101.7, 56.0, 55.4, 31.4.

IR (KBr): 3058, 3023, 2908, 2836, 1610, 1509, 1464, 1317, 1250, 1111, 1036, 1005, 825, 742, 700, 620 cm^{-1}.

HRMS (EI) [$C_{29}H_{25}NO$] $[M]^+$ calculated: 403.1936, found: 403.1939.

1-Methyl-2-(4-(phenyl(thiophen-2-yl)methyl)phenyl)-1*H*-indole (3pg)

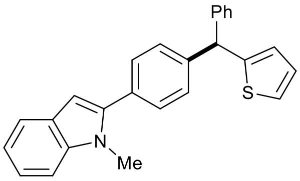

Compound **3pg** was prepared according to the general procedure **D** start from 2-(4-fluorophenyl)-1-methyl-1*H*-indole (45.0 mg, 0.2 mmol), and purified by silica gel column chromatography (*n*-hexane/DCM = 10/1) as a white solid (31.2 mg, yield: 41%).

m.p. = 138.0 – 139.4 °C.

^{1}H NMR (500 MHz, $CDCl_3$) δ 7.62 (d, *J* = 7.8 Hz, 1H), 7.47 – 7.41 (m, 2H), 7.38 – 7.28 (m, 5H), 7.30 – 7.24 (m, 3H), 7.26 – 7.20 (m, 2H), 7.17 – 7.09 (m, 1H), 6.96 (dd, *J* = 5.2, 3.5 Hz, 1H), 6.75 (dt, *J* = 3.5, 1.1 Hz, 1H), 6.55 (d, *J* = 0.9 Hz, 1H), 5.74 (s, 1H), 3.74 (s, 3H).

^{13}C NMR (126 MHz, $CDCl_3$) δ 147.7, 143.7, 141.4, 138.5, 131.3, 129.5, 129.1, 129.0, 128.7, 128.1, 127.0, 126.8, 126.6, 124.8, 121.8, 120.6, 120.0, 109.7, 101.8, 52.1, 31.4.

IR (KBr): 3058, 3025, 3026, 2867, 1600, 1545, 1491, 1467, 1315, 1131, 1100, 1006, 822, 750, 702, 639 cm^{-1}.

HRMS (EI) [$C_{26}H_{21}NS$] $[M]^+$ calculated: 379.1395, found: 379.1403.

9-(Biphenyl-4-yl)-9,10-dihydroanthracene (3ah)

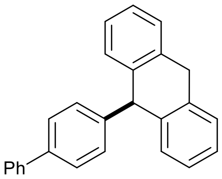

Compound **3ah** prepared according to the general procedure **D** start from 4-fluorobiphenyl (34.4 mg, 0.2 mmol), and purified by silica gel column chromatography (*n*-hexane) as a light-yellow solid (50.2 mg, yield: 76%).

m.p. = 151.0 – 151.8 °C.

^{1}H NMR (500 MHz, $CDCl_3$) δ 7.52 – 7.48 (m, 2H), 7.45 – 7.41 (m, 2H), 7.40 – 7.34 (m, 4H), 7.32 (dd, *J* = 5.2, 3.7 Hz, 2H), 7.31 – 7.26 (m, 1H), 7.25 – 7.20 (m, 4H), 7.13 (d, *J* = 8.2 Hz, 2H), 5.30 (s, 1H), 4.05 (d, *J* = 18.2 Hz, 1H), 3.92 (d, *J* = 18.2 Hz, 1H).

^{13}C NMR (126 MHz, $CDCl_3$) δ 142.8, 141.0, 39.5, 139.3, 136.6, 128.8, 128.6, 128.5, 128.0, 127.3, 127.2, 127.1, 126.6, 26.6, 51.3, 35.8.

IR (KBr): 3073, 3025, 2889, 1604, 1512, 1484, 1448, 1405, 1318, 1122, 1073, 1040, 1009, 962, 841, 780, 761, 744, 718, 697, 624 cm^{-1}.

HRMS (EI) [$C_{26}H_{20}$] $[M]^+$ calculated: 332.1565, found: 332.1576.

9-(Biphenyl-4-yl)-9*H*-xanthene (3ai)

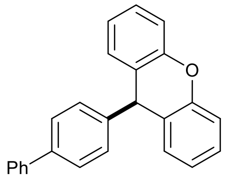

Compound **3ai** was prepared according to the general procedure **D** start from 4-fluorobiphenyl (34.4 mg, 0.2 mmol), and purified by silica gel column chromatography (*n*-hexane/DCM = 10/1) as a white solid (51.1 mg, yield: 77%).

^{1}H NMR (500 MHz, $CDCl_3$) δ 7.57 – 7.51 (m, 2H), 7.53 – 7.47 (m, 2H), 7.41 (t, *J* = 7.7 Hz, 2H), 7.36 – 7.28 (m, 1H), 7.29 – 7.26 (m, 2H), 7.25 – 7.19 (m, 2H), 7.16 (dd, *J* = 8.2, 1.3 Hz, 2H), 7.15 – 7.09 (m, 2H), 7.01 (td, *J* = 7.4, 1.4 Hz, 2H), 5.31 (s, 1H).

^{13}C NMR (126 MHz, $CDCl_3$) δ 151.2, 145.7, 140.9, 139.7, 129.9, 128.9, 128.1, 127.7, 127.3, 127.1, 124.5, 123.4, 116.8, 44.2.

MS(EI): *m/z* 334 $[M]^+$. The chemical shifts were consistent with those reported in the literature.[30]

9-(4'-Methylbiphenyl-4-yl)-9,10-dihydroanthracene (3jh)

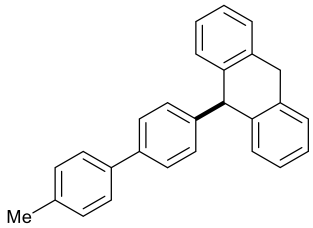

Compound **3jh** was prepared according to the general procedure **D** start from 4'-fluoro-4-methylbiphenyl (37.2 mg, 0.2 mmol), and purified by silica gel column chromatography (*n*-hexane) as a white solid (48.8 mg, yield: 71%).

m.p. = 137.3 – 138.1 ^oC.

^{1}H NMR (500 MHz, $CDCl_3$) δ 7.43 – 7.37 (m, 4H), 7.37 – 7.28 (m, 4H), 7.24 – 7.15 (m, 6H), 7.11 (d, *J* = 8.3 Hz, 2H), 5.28 (s, 1H), 4.04 (d, *J* = 18.2 Hz, 1H), 3.91 (d, *J* = 18.2 Hz, 1H), 2.34 (s, 3H).

^{13}C NMR (126 MHz, $CDCl_3$) δ 142.5, 139.6, 139.2, 138.1, 137.0, 136.6, 129.6, 128.6, 128.5, 128.0, 127.1, 126.9, 126.6, 126.6, 51.3, 35.8, 21.2.

IR (KBr): 3022, 2963, 1606, 1497, 1452, 1373, 1265, 1148, 1004, 798, 748, 685, 599 cm^{-1}.

HRMS (EI) [$C_{27}H_{22}$] $[M]^+$ calculated: 346.1722, found: 346.1725.

9-(4'-Methyl-biphenyl-4-yl)-9*H*-xanthene (3ji)

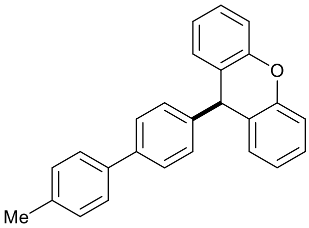

Compound **3ji** was prepared according to the general procedure **D** start from 4'-fluoro-4-methylbiphenyl (37.2 mg, 0.2 mmol), and purified by silica gel column chromatography (*n*-hexane) as a white solid (59.0 mg, yield: 85%).

m.p. = 170.6 – 171.8 °C.

^{1}H NMR (500 MHz, $CDCl_3$) δ 7.48 – 7.43 (m, 2H), 7.41 (d, *J* = 8.2 Hz, 2H), 7.25 – 7.19 (m, 2H), 7.19 (dd, *J* = 7.3, 1.6 Hz, 4H), 7.13 (dd, *J* = 8.2, 1.3 Hz, 2H), 7.09 (dd, *J* = 7.5, 1.6 Hz, 2H), 6.97 (td, *J* = 7.4, 1.4 Hz, 2H), 5.27 (s, 1H), 2.35 (s, 3H).

^{13}C NMR (126 MHz, $CDCl_3$) δ 151.2, 145.3, 139.6, 138.0, 137.1, 129.9, 129.6, 128.8, 128.0, 127.4, 127.0, 124.5, 123.4, 116.7, 44.2, 21.2.

IR (KBr): 3060, 3029, 2916, 2854, 1600, 1573, 1481, 1450, 1397, 1321, 1257, 1119, 1096, 1036, 1006, 801, 751, 619 cm^{-1}.

HRMS (EI) [$C_{26}H_{20}O$] $[M]^+$ calculated: 348.1514, found: 348.1509.

9-(4'-Methoxybiphenyl-4-yl)-9,10-dihydroanthracene (3kh)

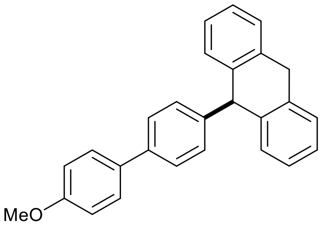

Compound **3kh** was prepared according to the general procedure **D** start from 4-fluoro-4'-methoxybiphenyl (40.5 mg, 0.2 mmol), and purified by silica gel column chromatography (*n*-hexane/DCM = 6/1) as a white solid (54.2 mg, yield: 75%).

m.p. = 177.1 – 178.7 °C.

^{1}H NMR (500 MHz, $CDCl_3$) δ 7.49 (d, *J* = 8.8 Hz, 2H), 7.44 (d, *J* = 8.3 Hz, 2H), 7.43 – 7.35 (m, 4H), 7.31 – 7.25 (m, 4H), 7.17 (d, *J* = 8.1 Hz, 2H), 6.97 (d, *J* = 8.7 Hz, 2H), 5.34 (s, 1H), 4.10 (d, *J* = 18.2 Hz, 1H), 3.97 (d, *J* = 18.3 Hz, 1H), 3.85 (s, 3H).

^{13}C NMR (126 MHz, $CDCl_3$) δ 159.1, 142.1, 139.6, 138.9, 136.6, 133.5, 128.6, 128.5, 128.1, 128.0, 126.9, 126.6, 126.6, 114.3, 55.4, 51.3, 35.8.

IR (KBr): 3075, 3021, 2994, 2886, 1605, 1525, 1498, 1288, 1205, 1116, 1038, 1014, 800, 749, 686, 612 cm^{-1}.

HRMS (EI) [$C_{27}H_{22}O$] $[M]^+$ calculated: 362.1671, found: 362.1685.

9-(4'-Methoxy-biphenyl-4-yl)-9*H*-xanthene (3ki)

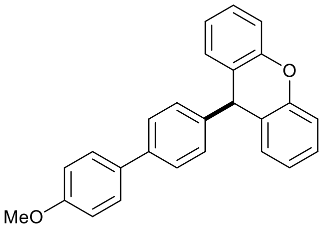

Compound **3ki** was prepared according to the general procedure **D** start from 4-fluoro-4'-methoxybiphenyl (40.5 mg, 0.2 mmol), and purified by silica gel column chromatography (*n*-hexane/DCM = 5/1) as a white solid (58.4 mg, yield: 80%).

m.p. = 161.7 – 163.2 ^{o}C.

^{1}H NMR (500 MHz, $CDCl_3$) δ 7.43 (t, *J* = 8.0 Hz, 4H), 7.20 (dd, *J* = 14.1, 6.9 Hz, 4H), 7.13 (d, *J* = 8.2 Hz, 2H), 7.08 (d, *J* = 8.1 Hz, 2H), 6.97 (t, *J* = 7.4 Hz, 2H), 6.91 (d, *J* = 8.4 Hz, 2H), 5.25 (s, 1H), 3.78 (s, 3H).

^{13}C NMR (126 MHz, $CDCl_3$) δ 159.2, 151.2, 145.0, 139.2, 133.4, 129.8, 128.8, 128.1, 128.0, 127.2, 124.5, 123.4, 116.7, 114.3, 55.4, 44.2.

IR (KBr): 3038, 2963, 2908, 2839, 1606, 1477, 1400, 1256, 1182, 1119, 1036, 936, 904, 835, 753, 684, 617 cm^{-1}.

HRMS (EI) [$C_{26}H_{20}O_2$] [M]$^+$ calculated: 364.1463, found: 364.1462.

9-(4'-(Benzyloxy)-[1,1'-biphenyl]-4-yl)-9,10-dihydroanthracene (3lh)

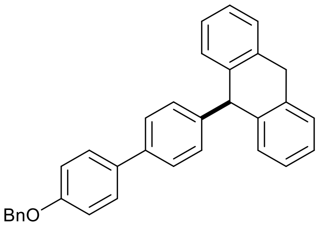

Compound **3lh** was prepared according to the general procedure **D** start from 4-(benzyloxy)-4'-fluorobiphenyl (55.6 mg, 0.2 mmol), and purified by silica gel column chromatography (*n*-hexane/DCM = 6/1) as a white solid (39.4 mg, yield: 45%).

m.p. = 168.7 – 169.8 ^{o}C.

^{1}H NMR (300 MHz, $CDCl_3$) δ 7.50 – 7.29 (m, 13H), 7.25 – 7.18 (m, 4H), 7.11 (d, *J* = 8.1 Hz, 2H), 6.99 (d, *J* = 8.7 Hz, 2H), 5.29 (s, 1H), 5.07 (s, 2H), 4.05 (d, *J* = 18.2 Hz, 1H), 3.91 (d, *J* = 18.3 Hz, 1H).

^{13}C NMR (176 MHz, $CDCl_3$) δ 158.4, 142.2, 139.6, 138.8, 137.1, 136.6, 133.8, 128.7, 128.6, 128.5, 128.1, 128.0, 127.6, 126.9, 126.6, 126.57, 115.2, 70.2, 51.3, 35.8.

IR (KBr): 3063, 3031, 2918, 2894, 1607, 1579, 1498, 1451, 1381, 1253, 1199, 1177, 1041, 1000, 799, 738, 694 cm^{-1}.

HRMS (EI) [$C_{33}H_{26}O$] [M]$^+$ calculated: 438.1984, found: 438.1989.

5-(4-(9,10-Dihydroanthracen-9-yl)phenyl)benzo[*d*][1,3]dioxole (3nh)

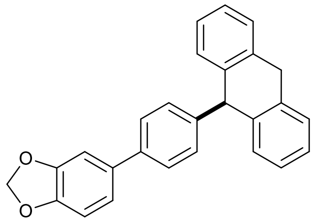

Compound **3nh** was prepared according to the general procedure **D** start from 5-(4-fluorophenyl)benzo[*d*][1,3]dioxole (43.2 mg, 0.2 mmol), and purified by silica gel column chromatography (*n*-hexane/DCM = 5/1) as a white solid (53.4 mg, yield: 71%).

m.p. = 127.8 – 129.2 ^{o}C.

^{1}H NMR (500 MHz, $CDCl_3$) δ 7.44 – 7.35 (m, 6H), 7.30 – 7.26 (m, 4H), 7.15 (d, *J* = 8.3 Hz, 2H), 7.05 – 6.99 (m, 2H), 6.87 (d, *J* = 8.0 Hz, 1H), 5.99 (s, 2H), 5.33 (s, 1H), 4.10 (d, *J* = 18.2 Hz, 1H), 3.97 (d, *J* = 18.2 Hz, 1H).

^{13}C NMR (126 MHz, $CDCl_3$) δ 148.1, 147.0, 142.5, 139.5, 138.9, 136.5, 135.3, 128.6, 128.5, 128.0, 127.0, 126.6, 126.5, 120.5, 108.6, 107.6, 101.2, 51.2, 35.8.

IR (KBr): 3079, 3011, 2909, 2822, 1611, 1460, 1345, 1253, 1153, 1121, 1036, 962, 938, 892, 837, 799, 756, 625 cm^{-1}.

HRMS (EI) [$C_{27}H_{20}O_2$] $[M]^+$ calculated: 376.1463, found: 376.1459.

9-(4-(Benzo[*d*][1,3]dioxol-5-yl)phenyl)-9*H*-xanthene (3ni)

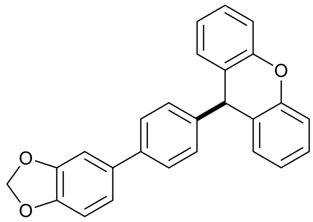

Compound **3ni** was prepared according to the general procedure **D** start from 5-(4-fluorophenyl)benzo[*d*][1,3]dioxole (43.2 mg, 0.2 mmol), and purified by silica gel column chromatography (*n*-hexane/DCM = 5/1) as a white solid (55.6 mg, yield: 74%).

m.p. = 190.6 – 191.3 ^{o}C.

^{1}H NMR (500 MHz, $CDCl_3$) δ 7.43 (d, *J* = 8.3 Hz, 2H), 7.26 – 7.22 (m, 4H), 7.17 (dd, *J* = 8.2, 1.4 Hz, 2H), 7.12 (dd, *J* = 7.8, 1.6 Hz, 2H), 7.04 – 6.99 (m, 4H), 6.86 (d, *J* = 7.9 Hz, 1H), 5.98 (s, 2H), 5.30 (s, 1H).

^{13}C NMR (126 MHz, $CDCl_3$) δ 151.2, 148.2, 147.1, 145.3, 139.4, 135.3, 129.8, 128.8, 128.1, 127.3, 124.5, 123.4, 120.6, 116.7, 108.7, 107.6, 101.2, 44.2.

IR (KBr): 3071, 3046, 1603, 1480, 1451, 1326, 1233, 1219, 1107, 1038, 935, 805, 748, 693 cm^{-1}.

HRMS (EI) [$C_{26}H_{18}O_3$] $[M]^+$ calculated: 378.1256, found: 378.1267.

2-(4-(9*H*-Xanthen-9-yl)phenyl)-1-methyl-1*H*-indole (3pi)

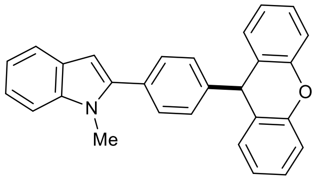

Compound **3pi** was prepared according to the general procedure **D** start from 2-(4-fluorophenyl)-1-methyl-1*H*-indole (45.0 mg, 0.2 mmol), and purified by silica gel column chromatography (*n*-hexane/EtOAc = 40/1) as a white solid (57.0 mg, yield: 74%).

m.p. = 130.4 – 131.8 ^{o}C.

^{1}H NMR (500 MHz, $CDCl_3$) δ 7.67 (dt, *J* = 7.8, 1.0 Hz, 1H), 7.49 – 7.42 (m, 2H), 7.41 – 7.35 (m, 1H), 7.36 – 7.30 (m, 2H), 7.33 – 7.25 (m, 3H), 7.23 (dd, *J* = 8.3, 1.3 Hz, 2H), 7.22 – 7.15 (m, 3H), 7.07 (td, *J* = 7.3, 1.4 Hz, 2H), 6.56 (d, *J* = 0.8 Hz, 1H), 5.36 (s, 1H), 3.73 (s, 3H).

^{13}C NMR (126 MHz, $CDCl_3$) δ 151.3, 146.3, 141.3, 138.5, 131.3, 129.82, 129.79, 128.4, 128.2, 128.0, 124.3, 123.5, 121.8, 120.5, 120.0, 116.8, 109.7, 101.7, 44.4, 31.3.

IR (KBr): 3060, 3034, 2946, 1600, 1573, 1480, 1448, 1413, 1316, 1256, 1163, 1095, 1007, 905, 860, 753, 736 cm^{-1}.

HRMS (EI) [$C_{28}H_{21}NO$] $[M]^+$ calculated: 387.1623, found: 387.1617.

4-(1,1-Diphenylethyl)-biphenyl (3aj)

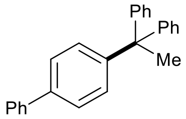

Compound **3aj** was prepared according to the general procedure **D** start from 4-fluorobiphenyl (34.4 mg, 0.2 mmol), and purified by silica gel column chromatography (*n*-hexane) as a white solid (53.0 mg, yield: 79%).

m.p. = 109.9 – 110.7 ^{o}C.

^{1}H NMR (300 MHz, $CDCl_3$) δ 7.59 (d, *J* = 6.6 Hz, 2H), 7.56 – 7.45 (m, 2H), 7.42 (t, *J* = 7.4 Hz, 2H), 7.38 – 7.16 (m, 7H), 7.22 – 7.09 (m, 6H), 2.22 (s, 3H).

^{13}C NMR (75 MHz, $CDCl_3$) δ 149.1, 148.3, 140.8, 138.8, 129.3, 128.9, 128.0, 127.3, 127.1, 126.6, 126.2, 52.5, 30.6.

IR (KBr): 3056, 3027, 2977, 1596, 1490, 1443, 1402, 1213, 1157, 1028, 1004, 860, 766, 733, 692, 624, 573 cm^{-1}.

HRMS (EI) [$C_{26}H_{22}$] $[M]^+$ calculated: 334.1722, found: 334.1725.

4-(1,1-Diphenylpropyl)-biphenyl (3ak)

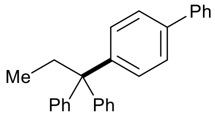

Compound **3ak** was prepared according to the general procedure **D** start from 4-fluorobiphenyl (34.4 mg, 0.2 mmol), and purified by silica gel column chromatography (*n*-hexane/DCM = 10/1) as an orange oil (44.7 mg, yield: 64%).

^{1}H NMR (700 MHz, $CDCl_3$) δ 7.64 – 7.61 (m, 2H), 7.55 – 7.53 (m, 2H), 7.46 – 7.43 (m, 2H), 7.40 – 7.38 (m, 2H), 7.37 – 7.34 (m, 5H), 7.32 (t, *J* = 7.6 Hz, 4H), 7.24 – 7.20 (m, 2H), 2.71 (q, *J* = 7.3 Hz, 2H), 0.85 (t, *J* = 7.3 Hz, 3H).

^{13}C NMR (176 MHz, $CDCl_3$) δ 147.4, 146.6, 140.9, 138.5, 129.8, 129.5, 128.8, 127.9, 127.2, 127.1, 126.5, 125.9, 56.9, 32.9, 10.6.

IR (KBr): 3086, 3056, 3027, 2975, 2935, 2879, 1599, 1487, 1445, 1008, 831, 761, 733, 700, 634 cm^{-1}.

HRMS (EI) [$C_{27}H_{24}$] $[M]^+$ calculated: 348.1878, found: 348.1883.

4-(1,1-Diphenylpentyl)-biphenyl (3al)

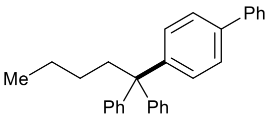

Compound **3al** was prepared according to the general procedure **D** start from 4-fluorobiphenyl (34.4 mg, 0.2 mmol), and purified by silica gel column chromatography (*n*-hexane/DCM = 10/1) as an orange oil (38.0 mg, yield: 51%).

^{1}H NMR (700 MHz, $CDCl_3$) δ 7.60 (d, *J* = 8.4 Hz, 2H), 7.51 (d, *J* = 7.9 Hz, 2H), 7.45 – 7.41 (m, 2H), 7.37 – 7.31 (m, 7H), 7.31 – 7.27 (m, 4H), 7.23 – 7.18 (m, 2H), 2.66 – 2.57 (m, 2H), 1.38 (h, *J* = 7.4 Hz, 2H), 1.15 – 1.08 (m, 2H), 0.88 (t, *J* = 7.3 Hz, 3H).

^{13}C NMR (176 MHz, $CDCl_3$) δ 147.7, 146.9, 140.9, 138.5, 129.8, 129.4, 128.8, 127.9, 127.2, 127.1, 126.5, 125.9, 56.5, 40.4, 28.0, 23.6, 14.2.

IR (KBr): 3085, 3057, 3030, 2955, 2871, 1599, 1487, 1469, 1444, 1007, 763, 731, 701, 635 cm^{-1}.

HRMS (EI) [$C_{29}H_{28}$] $[M]^+$ calculated: 376.2191, found: 376.2202.

4-(2-Phenylpropan-2-yl)biphenyl (3am)

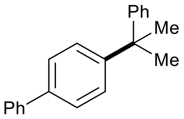

Compound **3am** prepared according to the general procedure **D** start from 4-fluorobiphenyl (34.4 mg, 0.2 mmol), and purified by silica gel column chromatography (*n*-hexane) as a clear oil (12.5 mg, yield: 23%).

^{1}H NMR (300 MHz, $CDCl_3$) δ 7.59 (dd, *J* = 8.3, 1.2 Hz, 2H), 7.52 (d, *J* = 8.5 Hz, 2H), 7.43 (t, *J* = 7.4 Hz, 2H), 7.37 – 7.28 (m, 7H), 7.24 – 7.17 (m, 1H), 1.74 (s, 6H).

MS(EI): *m/z* 272 $[M]^+$.

The chemical shifts were consistent with those reported in the literature.[31]

4-(1-Phenylethyl)-biphenyl (3an)

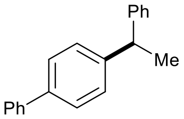

Compound **3an** prepared according to the general procedure **D** start from 4-fluorobiphenyl (34.4 mg, 0.2 mmol), and purified by silica gel column chromatography (*n*-hexane) as a white solid (13.0 mg, yield: 25%).

^{1}H NMR (300 MHz, $CDCl_3$) δ 7.58 (d, *J* = 7.2 Hz, 2H), 7.53 (d, *J* = 7.9 Hz, 2H), 7.43 (t, *J* = 7.5 Hz, 2H), 7.39 – 7.18 (m, 8H), 4.21 (q, *J* = 7.2 Hz, 1H), 1.69 (d, *J* = 7.2 Hz, 3H).

MS(EI): *m/z* 258 $[M]^+$.

The chemical shifts were consistent with those reported in the literature.[32]

4-(1-Phenylbutyl)biphenyl (3ao)

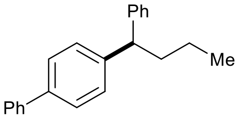

Compound **3ao** was prepared according to the general procedure **D** start from 4-fluorobiphenyl (34.4 mg, 0.2 mmol), and purified by silica gel column chromatography (*n*-hexane) as a light-yellow oil (12.6 mg, yield: 22%).

^{1}H NMR (300 MHz, $CDCl_3$) δ 7.59 – 7.54 (m, 2H), 7.51 (d, *J* = 8.3 Hz, 2H), 7.46 – 7.38 (m, 2H), 7.36 – 7.28 (m, 7H), 7.23 – 7.16 (m, 1H), 3.96 (t, *J* = 7.7 Hz, 1H), 2.06 (q, *J* = 7.7 Hz, 2H), 1.38 – 1.27 (m, 2H), 0.95 (t, *J* = 7.3 Hz, 3H).

MS(EI): *m/z* 286 $[M]^+$.

The chemical shifts were consistent with those reported in the literature.[33]

4-(1-Phenylbut-3-en-1-yl)biphenyl (3ap)

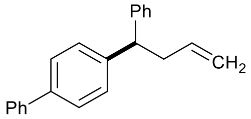

Compound **3ap** was prepared according to the general procedure **D** start from 4-fluorobiphenyl (34.4 mg, 0.2 mmol), and purified by silica gel column chromatography (*n*-hexane) as a light-yellow oil (11.4 mg, yield: 20%).

^{1}H NMR (300 MHz, $CDCl_3$) δ 7.57 (d, *J* = 7.0 Hz, 2H), 7.52 (d, *J* = 8.2 Hz, 2H), 7.42 (t, *J* = 7.4 Hz, 2H), 7.37 – 7.26 (m, 7H), 7.26 – 7.14 (m, 1H), 5.76 (ddt, *J* = 17.0, 10.2, 6.8 Hz, 1H), 5.07 (dd, *J* = 17.1, 1.8 Hz, 1H), 4.98 (dd, *J* = 10.3, 1.9 Hz, 1H), 4.06 (t, *J* = 7.9 Hz, 1H), 2.86 (t, *J* = 7.9 Hz, 2H).

MS(EI): *m/z* 284 $[M]^+$.

The chemical shifts were consistent with those reported in the literature.[34]

1-(4'-Biphenyl)-1-phenyl propene (3aq)

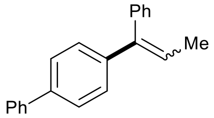

Compound **3aq** was prepared according to the general procedure **D** start from 4-fluorobiphenyl (34.4 mg, 0.2 mmol), and purified by silica gel column chromatography (*n*-hexane) as a white solid (18.3 mg, yield: 34%). ^{1}H NMR spectral copied below consist of two isomers ((*E*)-**3aq**/(*Z*)-**3aq** = 1.3/1), the ^{1}H NMR characterization data of the major isomer (*E*)-**3aq** is:

^{1}H NMR (300 MHz, $CDCl_3$) δ 7.61 – 7.17 (m, 14H), 6.25 (q, *J* = 6.9 Hz, 1H), 1.78 (d, *J* = 7.1 Hz, 3H).

MS(EI): *m/z* 270 $[M]^+$.

The chemical shifts were consistent with those reported in the literature.[35]

Di(biphenyl-4-yl)methane (3ar)

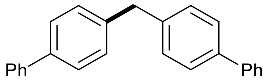

Compound **3ar** was prepared according to the general procedure **D** start from 4-fluorobiphenyl (34.4 mg, 0.2 mmol), and purified by silica gel column chromatography (*n*-hexane) as a colorless solid (9.7 mg, yield: 15%).

^{1}H NMR (300 MHz, $CDCl_3$) δ 7.68 – 7.49 (m, 8H), 7.48 – 7.38 (m, 4H), 7.37 – 7.26 (m, 6H), 4.07 (s, 2H).

MS(EI): *m/z* 320 $[M]^+$.

The chemical shifts were consistent with those reported in the literature.[36]

(8*R*,9*S*,13*S*,14*S*)-3-Benzhydryl-17-methoxy-13-methyl-7,8,9,11,12,13,14,15,16,17-decahydro-6*H*-cyclopenta[*a*]phenanthrene (3ra)

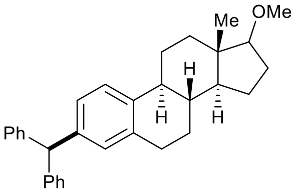

Compound **3ra** was prepared according to the general procedure **D** start from fluoro-estron derivative (57.6 mg, 0.2 mmol), and purified by silica gel column chromatography (*n*-hexane/DCM = 10/1) as a colorless oil (52.5 mg, yield: 60%).

^{1}H NMR (700 MHz, $CDCl_3$) δ 7.34 – 7.25 (m, 4H), 7.26 – 7.17 (m, 3H), 7.19 – 7.10 (m, 4H), 6.92 – 6.84 (m, 1H), 6.85 (s, 1H), 5.49 (s, 1H), 3.39 (s, 3H), 3.33 (t, *J* = 8.4 Hz, 1H), 2.87 – 2.72 (m, 2H), 2.32 – 2.27 (m, 1H), 2.26 – 2.21 (m, 1H), 2.13 – 2.02 (m, 2H), 1.92 – 1.82 (m, 1H), 1.76 – 1.64 (m, 1H), 1.58 – 1.49 (m, 1H), 1.49 – 1.43 (m, 1H), 1.46 – 1.36 (m, 1H), 1.41 – 1.28 (m, 2H), 1.24 – 1.18 (m, 1H), 0.90 (t, *J* = 7.1 Hz, 1H), 0.81 (s, 3H).

^{13}C NMR (176 MHz, $CDCl_3$) δ 144.3, 144.2, 141.1, 138.4, 136.7, 130.0, 129.6, 128.4, 126.8, 126.3, 125.3, 90.9, 58.0, 56.6, 50.6, 44.4, 43.3, 38.4, 29.7, 27.9, 27.4, 26.3, 23.2, 11.7.

IR (KBr): 3060, 3024, 2927, 2866, 1599, 1494, 1449, 1248, 1133, 1108, 1077, 1031, 748, 700 cm^{-1}.

HRMS (ESI) [$C_{32}H_{36}ONa$] $[M+Na]^+$ calculated: 459.2658, found: 459.2620.

(6*S*,8*R*,9*S*,13*S*,14*S*)-6-([1,1'-Biphenyl]-4-yl)-3,17-dimethoxy-13-methyl-7,8,9,11,12,13,14,15,16,17-decahydro-6*H*-cyclopenta[*a*]phenanthrene (3as)

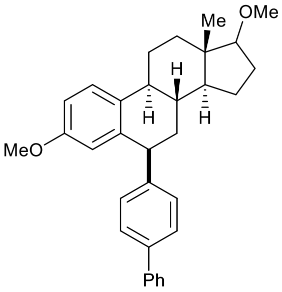

Compound **3as** was was prepared according to the general procedure **D** start from 4-fluorobiphenyl (34.4 mg, 0.2 mmol), and purified by silica gel column chromatography (toluene) as a colorless oil (24.0 mg, yield: 27%).

^{1}H NMR (700 MHz, $CDCl_3$) δ 7.62 – 7.58 (m, 2H), 7.51 – 7.47 (m, 2H), 7.45 – 7.40 (m, 2H), 7.34 – 7.30 (m, 2H), 7.11 – 7.08 (m, 2H), 6.80 (dd, *J* = 8.7, 2.8 Hz, 1H), 6.50 (dd, *J* = 2.7, 0.7 Hz, 1H), δ 4.27 (dd, *J* = 6.6, 2.6 Hz, 1H), 3.70 (s, 3H), 3.36 (s, 3H), 3.30 (t, *J* = 8.3 Hz, 1H), 2.39 – 2.34 (m, 1H), 2.28 – 2.21 (m, 1H), 2.10 – 2.05 (m, 1H), 2.04 – 1.96 (m, 1H), 1.93 – 1.88 (m, 1H), 1.88 – 1.81 (m, 1H), 1.69 – 1.58 (m, 2H), 1.57 – 1.51 (m, 1H), 1.47 – 1.38 (m, 2H), 1.25 – 1.17 (m, 1H), 1.12 – 1.04 (m, 1H), 0.72 (s, 3H).

^{13}C NMR (176 MHz, $CDCl_3$) δ 157.7, 147.3, 141.1, 139.6, 138.6, 133.7, 129.3, 128.8, 127.2, 127.1, 126.9, 126.2, 115.4, 112.5, 90.9, 58.0, 55.3, 50.1, 44.3, 44.2, 43.6, 38.2, 36.0, 33.2, 27.8, 26.6, 23.0, 11.8.

IR (KBr): 3050, 3031, 2929, 2847, 1609, 1572, 1499, 1486, 1450, 1282, 1233, 1133, 1040, 847, 738, 697, cm^{-1}.

HRMS (ESI) [$C_{32}H_{36}O_2Na$] $[M+Na]^+$ calculated: 475.2608, found: 475.2613.

2-(4-((4-Methoxyphenyl)(phenanthren-9-yl)methyl)phenoxy)-*N*,*N*-dimethylethan-1-amine (3st)

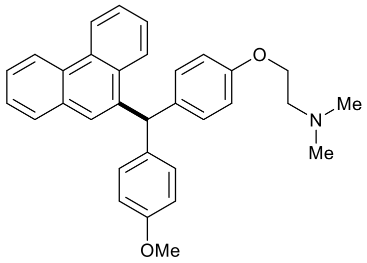

Compound **3st** was prepared according to the general procedure **D** start from 9-fluorophenanthrene (39.3 mg, 0.2 mmol), and purified by silica gel column chromatography (EtOAc with 3% Et_3N) as a colorless oil (63.9 mg, yield: 68%).

^{1}H NMR (700 MHz, $CDCl_3$) δ 8.72 (d, *J* = 8.2 Hz, 1H), 8.65 (d, *J* = 8.2 Hz, 1H), 8.04 (d, *J* = 8.0 Hz, 1H), 7.68 (d, *J* = 7.9 Hz, 1H), 7.62 – 7.58 (m, 2H), 7.54 – 7.51 (m, 1H), 7.50 – 7.47 (m, 1H), 7.15 (s, 1H), 7.06 (t, *J* = 8.7 Hz, 4H), 6.91 – 6.76 (m, 4H), 6.15 (s, 1H), 4.04 (t, *J* = 5.7 Hz, 2H), 3.78 (s, 3H), 2.72 (d, *J* = 5.6 Hz, 2H), 2.33 (s, 6H).

^{13}C NMR (176 MHz, $CDCl_3$) δ 158.2, 157.5, 139.0, 136.1, 136.0, 131.6 131.3, 130.9, 130.7, 130.6, 129.9, 128.8, 128.5, 126.7, 126.6, 126.5, 126.2, 125.4, 123.1, 122.5, 114.5, 113.9, 66.0, 58.4, 55.3, 51.9, 46.0.

IR (KBr): 3063, 3029, 2941, 1608, 1507, 1463, 1254, 1176, 1119, 1037, 839, 771, 748 cm^{-1}.

HRMS (ESI) [$C_{32}H_{31}NNaO_2$] $[M+Na]^+$ calculated: 484.2247, found: 484.2254.

4-Benzhydryl-4'-chlorobiphenyl (3ta)

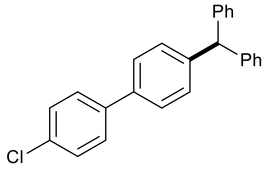

Compound **3ta** was prepared according to the general procedure **D** start from 4-chloro-4'-fluorobiphenyl (41.2 mg, 0.2 mmol), and purified by silica gel column chromatography (*n*-hexane/DCM = 10/1) as a white solid (16.4 mg, yield: 23%).

m.p. = 108.5 – 111.0 ^oC.

^{1}H NMR (500 MHz, $CDCl_3$) δ 7.60 – 7.56 (m, 1H), 7.54 – 7.50 (m, 2H), 7.48 – 7.39 (m, 2H), 7.35 – 7.27 (m, 5H), 7.23 (t, *J* = 7.3 Hz, 1H), 7.20 – 7.07 (m, 7H), 5.59 (s, 1H).

^{13}C NMR (126 MHz, $CDCl_3$) δ 144.0, 143.2, 141.0, 139.3, 130.0, 129.6, 128.9, 128.5, 127.3, 127.2, 126.5, 115.7, 56.7.

IR (KBr): 3055, 3029, 1598, 1494, 1448, 1222, 1159, 1078, 1030, 1008, 832, 801, 760, 747, 735, 606 cm^{-1}.

HRMS (EI) [$C_{25}H_{19}Cl$] $[M]^+$ calculated:354.1175, found: 354.1189.

4-Benzhydryl-3-(but-3-en-1-yl)biphenyl (3ua)

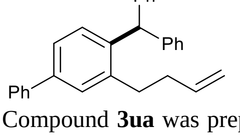

Compound **3ua** was prepared according to the general procedure **D** start from 3-(but-3-en-1-yl)-4-fluorobiphenyl (45.3 mg, 0.2 mmol), and purified by silica gel column chromatography (*n*-hexane/DCM = 10/1) as a white solid (51.0 mg, yield: 68%).

m.p. = 93.5 – 94.1 ^oC.

^{1}H NMR (300 MHz, $CDCl_3$) δ 7.58 (d, *J* = 7.0 Hz, 2H), 7.45 – 7.38 (m, 3H), 7.36 – 7.19 (m, 8H), 7.10 (d, *J* = 7.4 Hz, 4H), 6.92 (d, *J* = 8.0 Hz, 1H), 5.91 – 5.78 (m, 1H), 5.80 (s, 1H), 4.97 (d, *J* = 11.8 Hz, 2H), 2.73 (dd, *J* = 9.5, 6.5 Hz, 2H), 2.24 (q, *J* = 7.5 Hz, 2H).
^{13}C NMR (126 MHz, $CDCl_3$) δ 143.8, 141.1, 140.6, 139.3, 138.2, 130.6, 129.7, 128.8, 128.9, 128.5, 127.2, 127.1, 126.5, 124.7, 115.2, 52.7, 35.3, 32.6.
IR (KBr): 3060, 3026, 1640, 1599, 1483, 1449, 1300, 1231, 1077, 1030, 916, 748, 697, 607 cm^{-1}.
HRMS (EI) [$C_{29}H_{26}$] $[M]^+$ calculated: 374.2035, found: 374.2025.

1,1-Diphenyl-2,3-dihydro-1*H*-indene (3v)

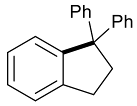

Compound **3v** was prepared according to the general procedure **D** start from (3-(2-Fluorophenyl)propane-1,1-diyl)dibenzene (58.0 mg, 0.2 mmol), and purified by silica gel column chromatography (*n*-hexane/DCM = 10/1) as a colorless oil (48.0 mg, yield: 89%).
^{1}H NMR (300 MHz, $CDCl_3$) δ 7.29 – 7.20 (m, 5H), 7.19 – 7.12 (m, 8H), 7.10 – 7.00 (m, 1H), 2.98 – 2.70 (m, 4H).
^{13}C NMR (75 MHz, $CDCl_3$) δ 149.4, 147.4, 143.9, 128.6, 128.0, 127.0, 126.4, 126.2, 126.1, 124.8, 62.0, 43.6, 30.8.
IR (KBr): 3059, 3021, 2948, 2905, 2845, 1596, 1492, 1473, 1443, 1025, 751, 700 cm^{-1}.
HRMS (EI) [$C_{21}H_{18}$] $[M]^+$ calculated: 270.1409, found: 270.1411.

2-(4'-Fluoro-biphenyl-4-yl)-4,4,5,5-tetramethyl-1,3,2-dioxaborolane (5)

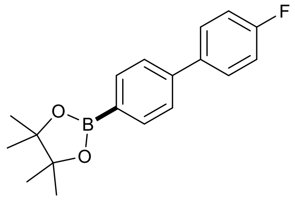

Compound **5** was prepared according to the general procedure **D** start from 4-chloro-4'-fluorobiphenyl (41.2 mg, 0.2 mmol), and purified by silica gel column chromatography (*n*-hexane/DCM = 5/1) as a white solid (34 mg, yield: 57%).
^{1}H NMR (500 MHz, $CDCl_3$) δ 7.89 (d, *J* = 8.2 Hz, 2H), 7.64 – 7.49 (m, 4H), 7.13 (t, *J* = 8.7 Hz, 2H), 1.37 (s, 12H).
^{13}C NMR (126 MHz, $CDCl_3$) δ 162.78 (d, *J* = 246.6 Hz), 143.0, 137.3, 135.5, 134.8, 128.9, 126.4, 115.8 (d, *J* = 21.4 Hz), 84.0, 25.0.
^{19}F NMR (282 MHz, $CDCl_3$) δ -115.19 – -116.27 (m, 1F).
HRMS (EI) [$C_{18}H_{20}BFO_2$] $[M]^+$ calculated: 298.1540, found: 298.1546.
The chemical shifts were consistent with those reported in the literature.[37]

Triethyl((4-methoxyphenyl)(phenyl)methyl)silane (10)

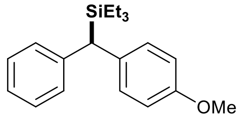

Compound **14** was purified by silica gel column chromatography (*n*-hexane/toluene = 1/1) as a colorless oil (6.2 mg, yield: 10%).
^{1}H NMR (700 MHz, $CDCl_3$) δ 7.28 – 7.18 (m, 4H), 7.22 – 7.14 (m, 2H), 7.15 – 7.06 (m, 1H), 6.91 – 6.71 (m, 2H), 3.77 (s, 3H), 3.59 (s, 1H), 0.85 (t, *J* = 7.9 Hz, 9H), 0.60 (q, *J* = 7.8 Hz, 6H).
MS(EI): *m/z* 312 $[M]^+$.

The chemical shifts were consistent with those reported in the literature.[38]

1-(Benzhydryloxy)-2,2,6,6-tetramethylpiperidine (Int-TEMPO)

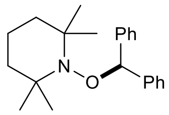

Compound **Int-TEMPO** was purified by silica gel column chromatography (*n*-hexane/DCM = 10/1) as a white solid (37.9 mg, yield: 58%).

^{1}H NMR (300 MHz, $CDCl_3$) δ 7.42 – 7.32 (m, 4H), 7.32 – 7.19 (m, 4H), 7.21 – 7.09 (m, 2H), 5.64 (s, 1H), 1.47 – 1.35 (m, 4H), 1.28 (d, J = 12.8 Hz, 2H), 1.15 (s, 6H), 0.74 (s, 6H).

MS(EI): *m/z* 323 $[M]^+$.

The chemical shifts were consistent with those reported in the literature.[39]

6. Preliminary Mechanistic Investigations

6.1 The NMR spectroscopic studies

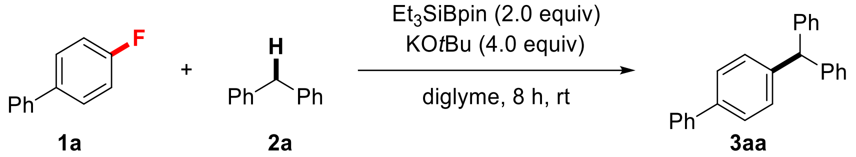

Following the **General Procedure D**, charging **1a** (17.2 mg, 0.1 mmol), **2a** (33.6 mg, 0.2 mmol), silyl boronate $Et_3SiBpin$ (48.4 mg, 0.2 mmol, 2.0 equiv), KO*t*Bu (45 mg, 0.4 mmol, 4.0 equiv), and then anhydrous diglyme (0.75 mL) sequentially. And then stirred in glovebox at room temperature for 8 h. The reaction mixture was then subjected to ^{11}B NMR and ^{19}F NMR analysis using THF-d^8 as a solvent to show the details of the reaction. After that the reaction mixture was quenched by adding D_2O (2.0 mL) while stirring for 5 min, then the ^{19}F NMR analysis of the water system were conducted to show the details of the reaction. The organic system was extracted with Et_2O, washed by water, dried over Na_2SO_4, and concentrated under vacuum, followed by 3-fluoropyridine (8.6 μL, 0.1 mmol) as an internal standard. Then the ^{1}H NMR analysis and ^{19}F NMR analysis of the crude mixture were conducted to show the details of the model reaction.

^{11}**B NMR** (225 MHz, THF-d_8)

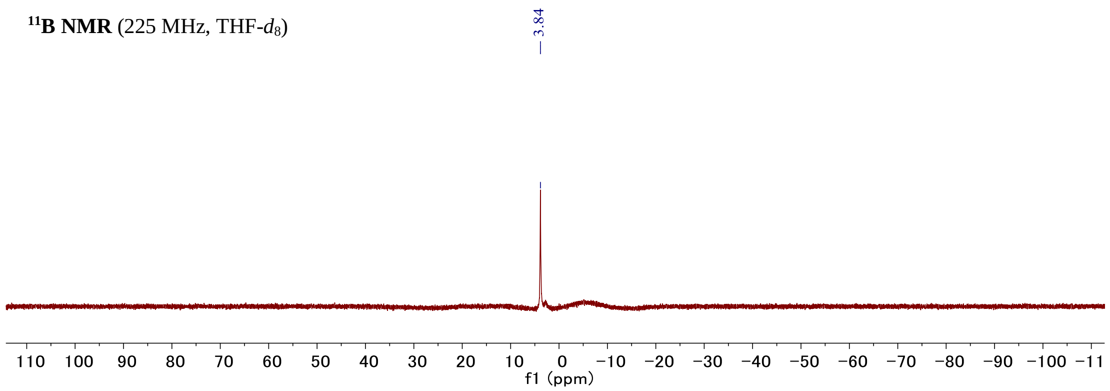

^{19}**F NMR** (282 MHz, THF-d_8)

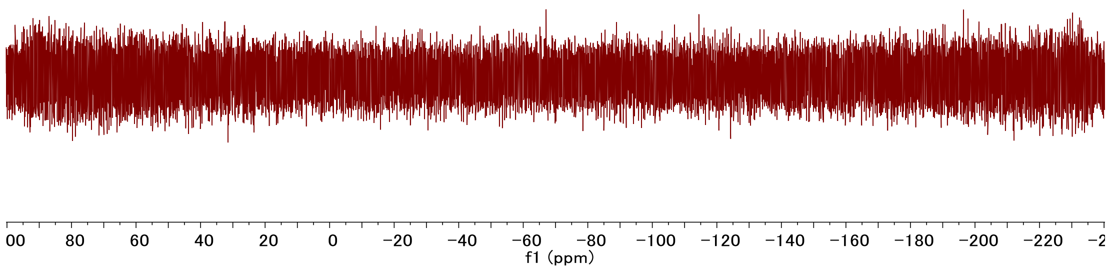

Figure S1.^{11}B NMR and ^{19}F NMR observation of the crude model reaction

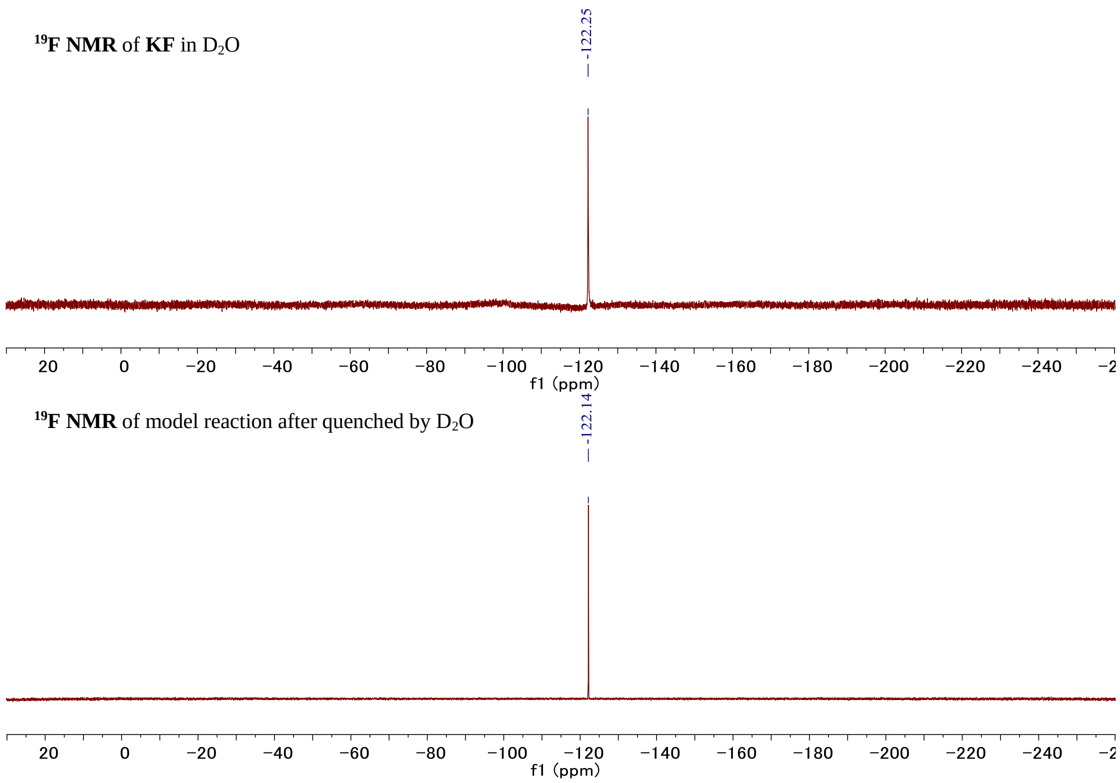

Figure S2. ^{19}F NMR observation of KF in D_2O and KF released in the model reaction

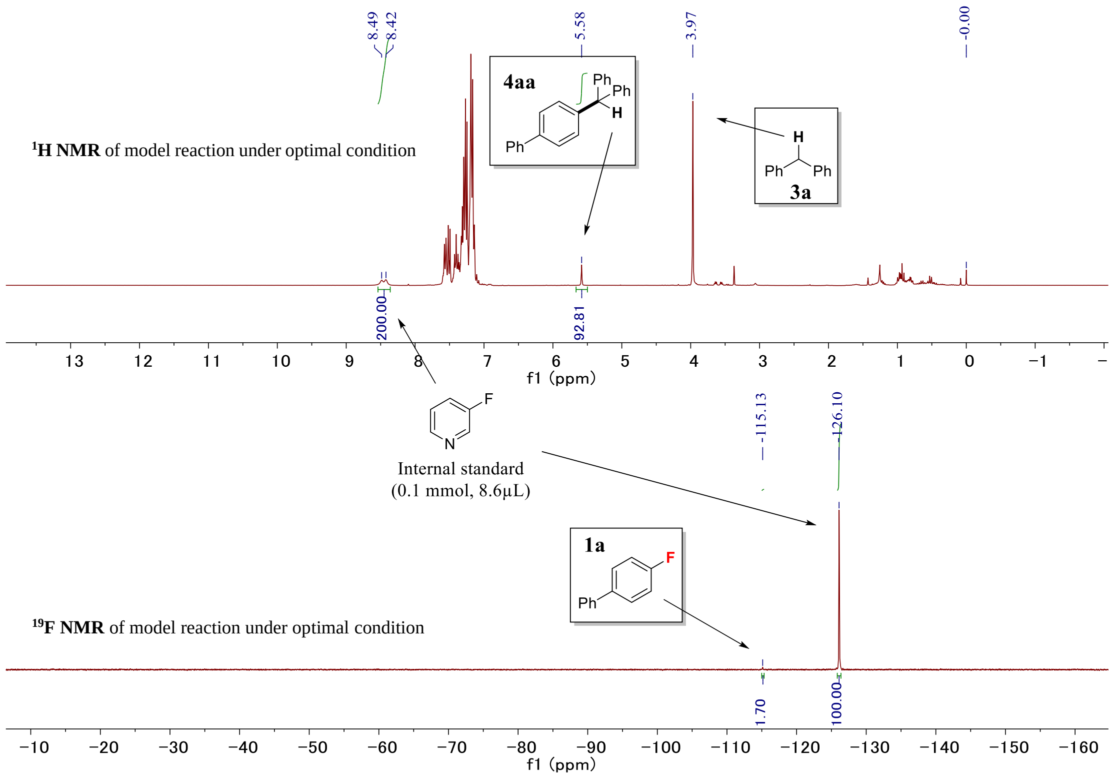

Figure S3. ^{1}H NMR and ^{19}F NMR observation of model reaction details.

6.2 Reaction with radical scavenger

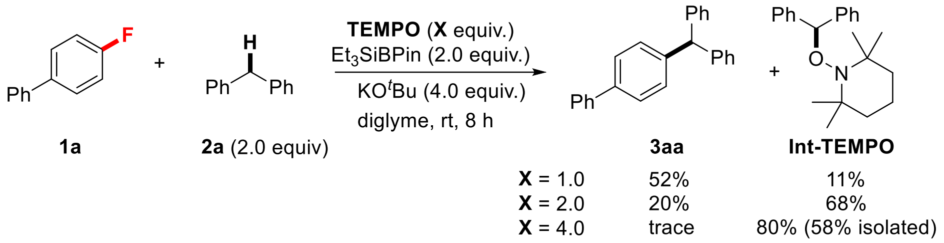

Figure S4. Effect of TEMPO to the silylboronate-mediated coupling reaction.

Following the **General Procedure D**, charging **1a** (17.2 mg, 0.1 mmol), **2a** (33.6 mg, 0.2 mmol), silyl boronate $Et_3SiBpin$ (48.4 mg, 0.2 mmol, 2.0 equiv), KO*t*Bu (45 mg, 0.4 mmol, 4.0 equiv), TEMPO, and then anhydrous diglyme (0.75 mL) sequentially. And then move out from glovebox and stirred at room temperature for 8 h. The reaction tube was diluted with Et_2O (5 mL), then extracted with Et_2O, washed with brine, dried over Na_2SO_4, then concentrated under vacuum, followed by adding 3-fluoropyridine (8.6 µL, 0.1 mmol) as an internal standard, then ^{1}H NMR analysis was taken to show the reaction details.

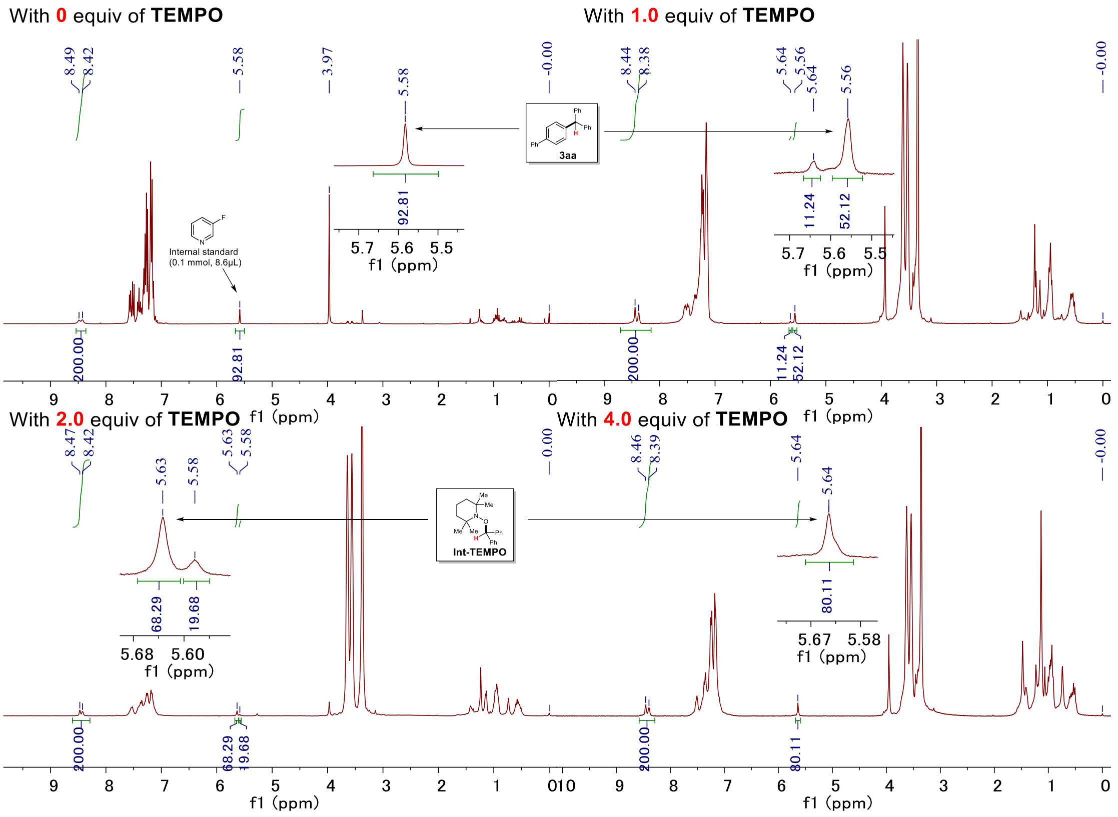

Figure S5. ^{1}H NMR observation of the Effect of TEMPO to the radical coupling reaction.

6.3 Control experiments involve radical-clock reactions

Ring-opening reaction attempt of 1a and 2u

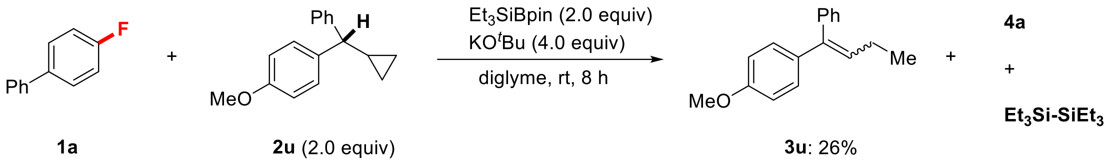

Following the general procedure **D**, charging **1a** (0.1 mmol), **2u** (47.5 mg, 0.2 mmol), silyl boronate (48.4 mg, 0.2 mmol), KO*t*Bu (45.0 mg, 0.4 mmol), and then anhydrous diglyme (1.0 mL) sequentially into a flame-dried screw-capped test tube. And then move out from glovebox and stirred at room temperature for 8 h. The reaction tube was diluted with Et_2O (5 mL), then extracted with Et_2O and water, washed with brine, dried over Na_2SO_4, then concentrated under vacuum, followed by adding 3-fluoropyridine (8.6 μL, 0.1 mmol) as an internal standard, then ^{19}F NMR analysis of the reaction crude and ^{1}H NMR analysis of isolated products were taken to show the reaction details.

The reaction using **1a** and **2u** failed to afford the corresponding cross-coupling product, however, the ring-opening product **3u** was isolated instead (26% yield), and we also detected the concomitant by-products, such as **4a** and hexaethyldisilane. The ^{1}H NMR of isolated products of **3u** are copied below.

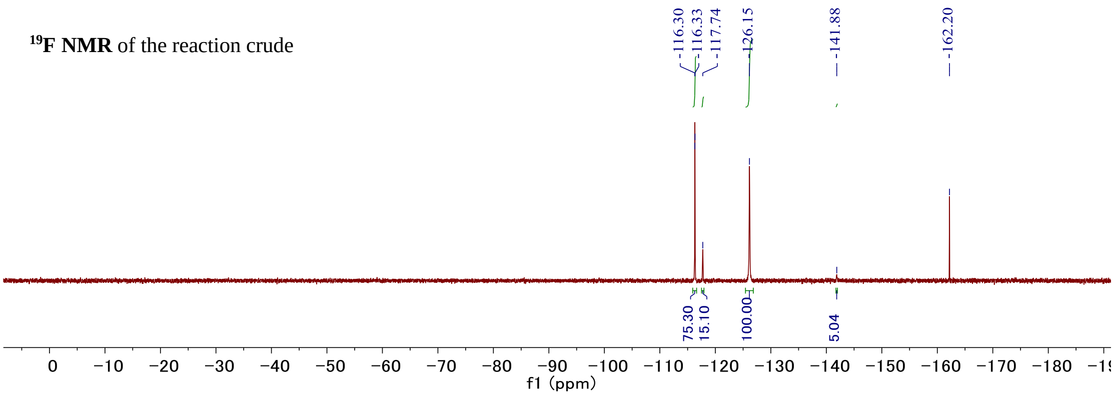

Figure S6. ^{19}F NMR of the reaction crude.

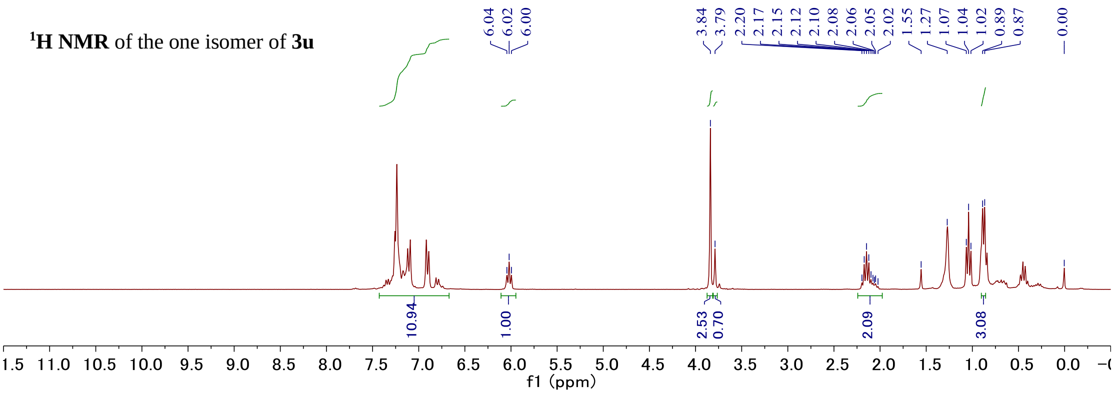

Figure S7. ^{1}H NMR of the one isomer of **3u**.

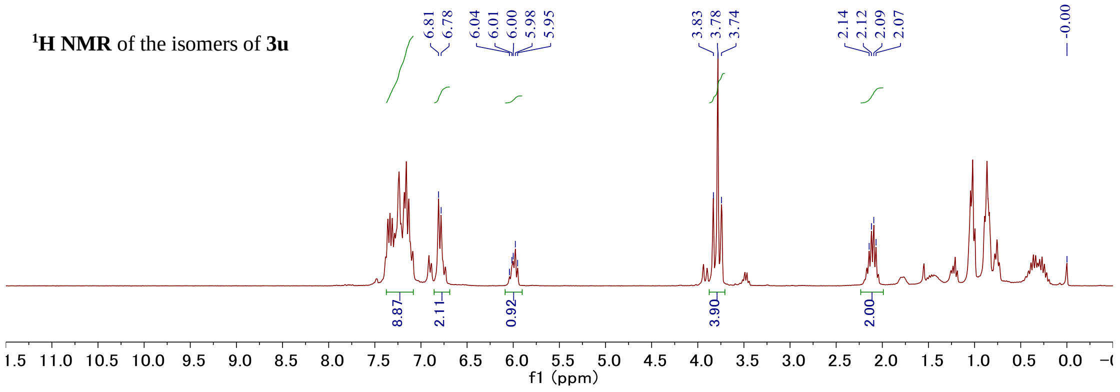

Figure S8. ^{1}H NMR of the isomers of **3u**.

Ring-cyclization attempt of 1v

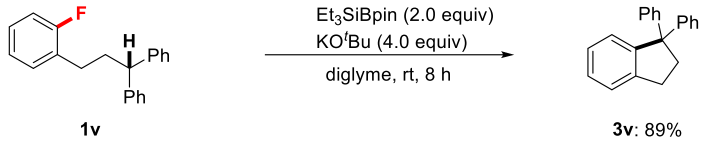

Following the general procedure **D**, charging **1v** (0.2 mmol), silyl boronate (96.8 mg, 0.4 mmol), KO*t*Bu (90.0 mg, 0.8 mmol), and then anhydrous diglyme (2.0 mL) sequentially into a flame-dried screw-capped test tube. And then move out from glovebox and stirred at room temperature for 8 h. The reaction tube was diluted with Et_2O (5 mL), then extracted with Et_2O and water, washed with brine, dried over Na_2SO_4, then concentrated under vacuum, followed by ^{19}F NMR and ^{1}H NMR analysis of the reaction crude to show the reaction details. Therefore, the intramolecular cross-coupling reaction was achieved by furnishing cyclization product **3v** in 89% yield, and trace of starting material **1v** (^{19}F NMR: –119.07 ppm) remains.

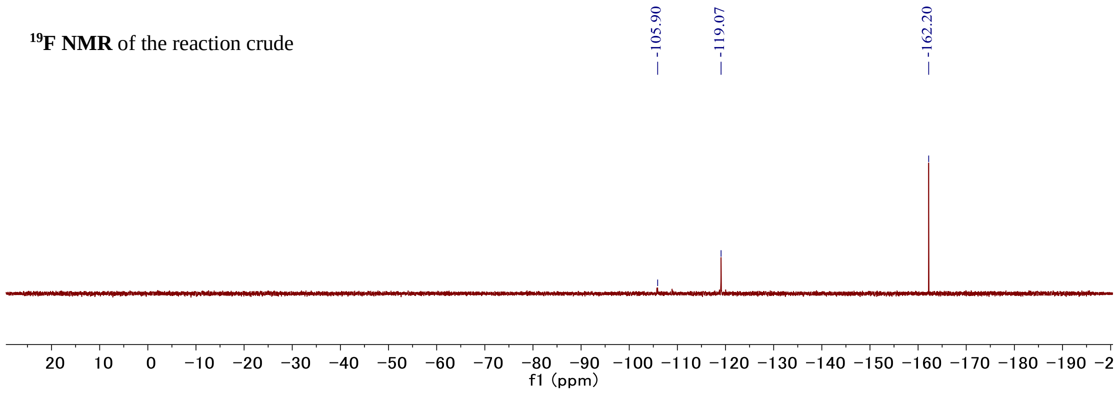

Figure S9. ^{19}F NMR observation of the ring-cyclization reaction crude.

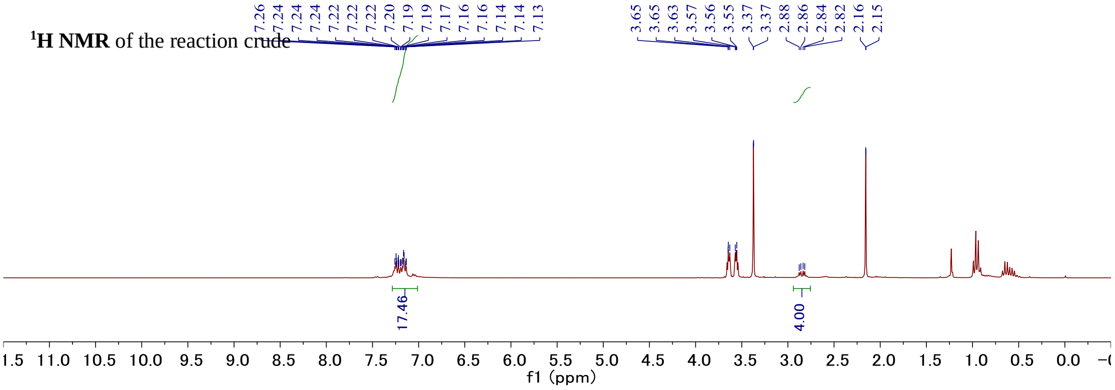

Figure S10. ^{1}H NMR observation of the ring-cyclization reaction crude.

6.4 Radical process regarding to the formation of 3aq

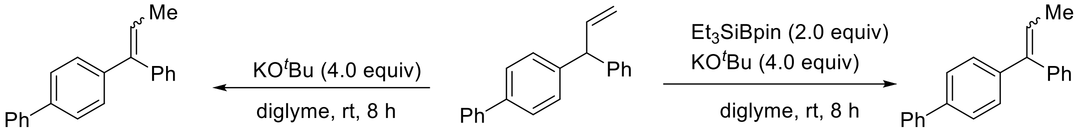

3aq: 83% yield (*Z*/*E* = 1:1) | **3aq'** | **3aq**: 28% yield (*Z*/*E* = 1:1.3)

Left reaction: In a N_2-filled glovebox, charging **3aq'** (54.0 mg, 0.2 mmol), anhydrous diglyme (1.0 mL), and then KO*t*Bu (45.0 mg, 0.4 mmol), sequentially into a flame-dried screw-capped test tube. And then move out from glovebox and stirred at room temperature for 8 h. The reaction tube was diluted with Et_2O (5 mL), then extracted with Et_2O and water, washed with brine, dried over Na_2SO_4, then concentrated under vacuum, followed by ^{1}H NMR analysis of the reaction crude to show the reaction details.

Right reaction: In a N_2-filled glovebox, charging **3aq'** (54.0 mg, 0.2 mmol), Et_3SiBpin (48.4 mg, 0.2 mmol), anhydrous diglyme (1.0 mL), and then KO*t*Bu (45.0 mg, 0.4 mmol), sequentially into a flame-dried screw-capped test tube. And then move out from glovebox and stirred at room temperature for 8 h. The reaction tube was diluted with Et_2O (5 mL), then extracted with Et_2O and water, washed with brine, dried over Na_2SO_4, then concentrated under vacuum, followed by ^{1}H NMR analysis of the reaction crude to show the reaction details.

According to above two control experiments, further insight into this reaction process was revealed. When the new synthesized **3aq'** was only treated with KO^{t}Bu in diglyme for 8 h at room temperature, **3aq'** was transformed into **3aq** in 83% ^{1}H NMR yield with *Z*/*E* = 1:1 ratio. However, when treated **3aq'** under standard conditions, **3aq** was detected in 28% ^{1}H NMR yield with *Z*/*E* = 1:1.3 ratio. Therefore, optimal reaction system (combination of Et_3SiBpin and KO^{t}Bu) didn't proceed through the deprotonation process.

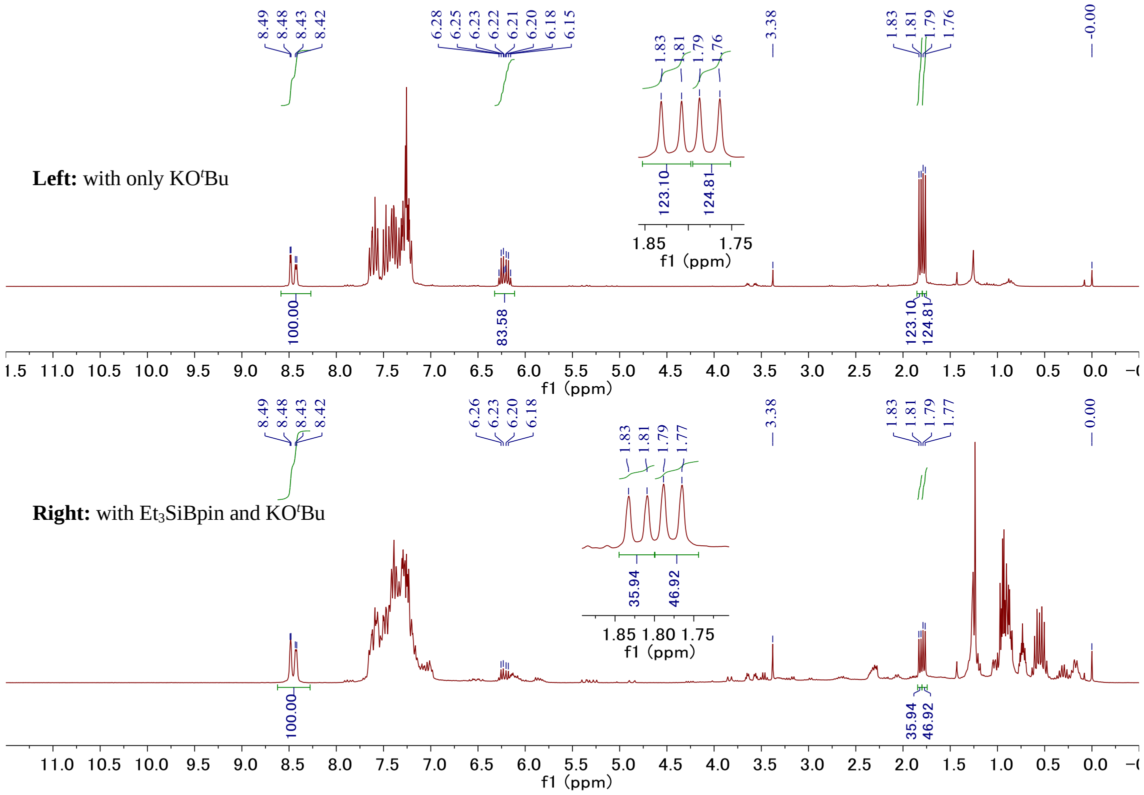

Figure S11. ^{1}H NMR observation of the crudes of the comparison reactions.

6.5 Control experiment involve nucleophilic aromatic substitution (S_NAr) process

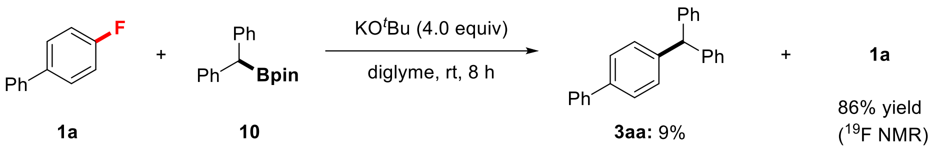

In a N_2-filled glovebox, charging **1a** (34.4 mg, 0.2 mmol), 2-benzhydryl-4,4,5,5-tetramethyl-1,3,2-dioxaborolane **10** (118 mg, 0.4 mmol), KO*t*Bu (90.0 mg, 0.8 mmol), and then anhydrous diglyme (2.0 mL) sequentially into a flame-dried screw-capped test tube. And then move out from glovebox and stirred at room temperature for 8 h. The reaction tube was diluted with Et_2O (5 mL), then extracted with Et_2O and water, washed with brine, dried over Na_2SO_4, then concentrated under vacuum, followed by ^{19}F NMR and ^{1}H NMR analysis of the reaction crude to show the reaction details.

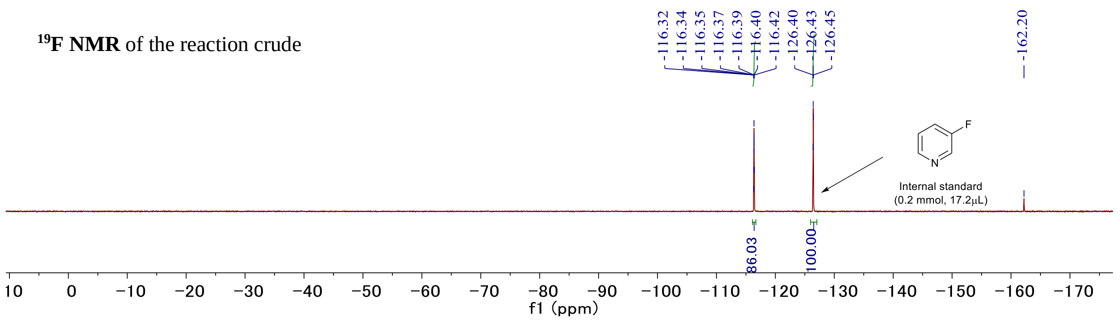

Figure S12. ^{19}F NMR observation of the reaction crude.

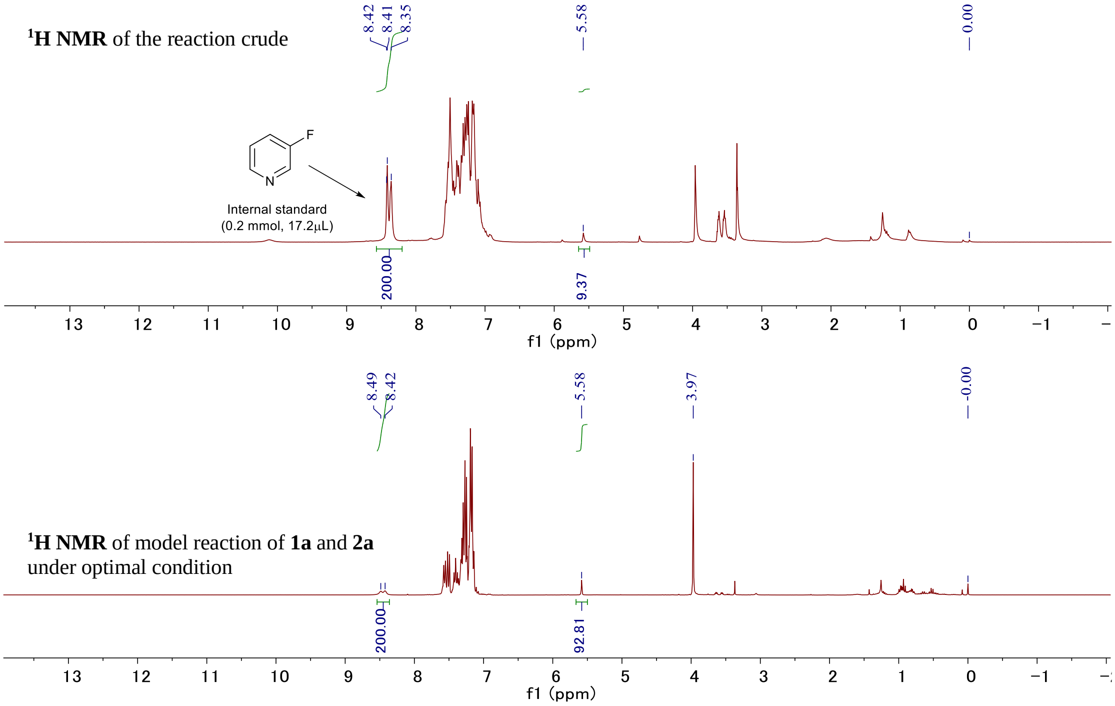

Figure S13. ^{1}H NMR spectrum comparison of the reaction crude.

6.6 Electron spin resonance (ESR) studies

Electron spin resonance (ESR) spectra, also referred to as electron paramagnetic resonance (EPR) spectra, were performed on a JEOL FA200 ESR spectrometer. ESR spectra were obtained at a microwave power level of 0.0997 ~ 0.998 mW and 100kHz filed modulation at room temperature (~ 288 K). The magnetic field was calibrated with the well-known splitting constants of Mn^{2+} in MgO. The *g*-values were determined by comparison with the spectrum of Mn^{2+} in MgO. Tri-*tert*-butyl nitrosobenzene (TTBNB, Aldrich) was used as spin-trapping reagent. TTBNB was purified by sublimation under reduced pressure.

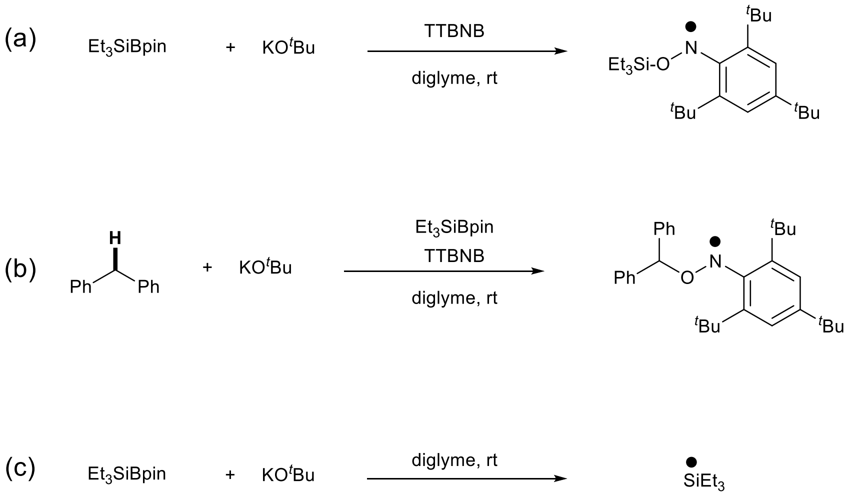

Figure S14. ESR experiments

Sample (a): In a N_2 filled glovebox, to a flame-dried screw-capped test tube was added KO*t*Bu (11.2 mg, 0.1 mmol, 1.0 equiv), silyl boronates $Et_3SiBpin$ (0.1 mmol, 1.0 equiv), dry diglyme (0.5 mL) sequentially. Stir the mixture till it turns to pale yellow, then 20 μL of TTBNB (0.01 M in diglyme) solution was added. Transfer 30 μL of mixture into a capillary tube that furtherly be put in the ESR test tube, the tube then was sealed and removed from the glovebox for conducting ESR measurement quickly.

Sample (b): In a N_2 filled glovebox, to a flame-dried screw-capped test tube was added KO*t*Bu (2.3 mg, 0.02 mmol, 2.0 equiv), silyl boronates $Et_3SiBpin$ (0.01 mmol, 1.0 equiv), diphenylmethane (0.02 mmol, 2.0 equiv), dry diglyme (0.2 mL) sequentially. Stir the mixture till it turns to pale yellow. then 20 μL of TTBNB (0.01 M in diglyme) solution was added, dilute the mixture by adding 1.5 mL diglyme. Transfer 30 μL of the mixture into a capillary tube that furtherly be put in the ESR test tube, the tube then was sealed and removed from the glovebox for conducting ESR measurement.

Sample (c): In a N_2 filled glovebox, to a flame-dried screw-capped test tube was added KO*t*Bu (11.2 mg, 0.1 mmol, 1.0 equiv), silyl boronates $Et_3SiBpin$ (0.1 mmol, 1.0 equiv), dry diglyme (0.5 mL) sequentially. Stir mixture till it turns to pale yellow, transfer 30 μL of the mixture into a capillary tube that furtherly be put in the ESR test tube, the tube then was sealed and removed from the glovebox for conducting ESR measurement quickly.

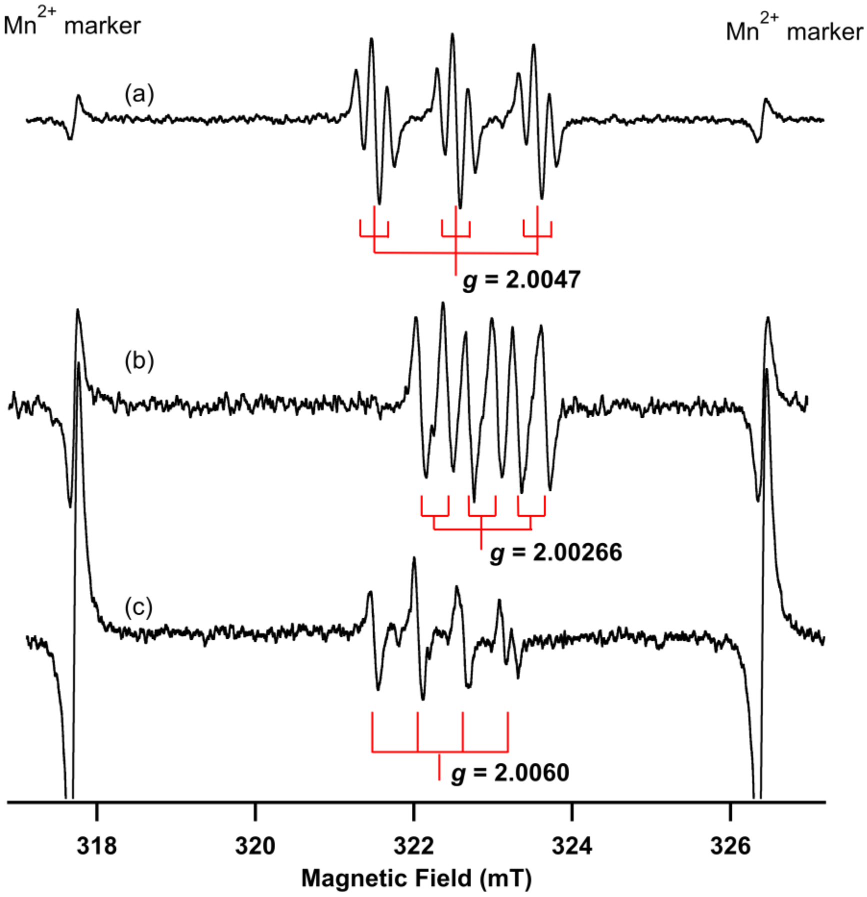

Figure S15. ESR spectra of spin-adducts: (a) the triethyl silyl radicals (silyl radicals) and (b) diphenyl methyl radicals (benzyl radicals) trapped with TTBNB in diglyme solvent. Spectrum (c) can be assigned to triethyl silyl radicals.

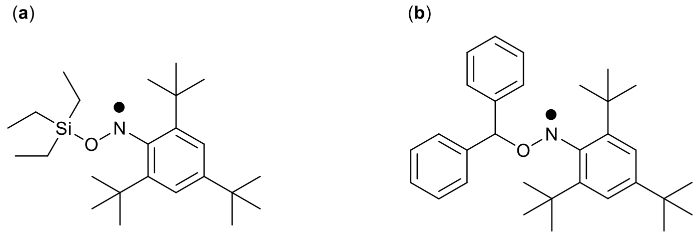

Figure S16. Chemical structure of spin-adducts; (a) triethyl silyl radical (anilino-type) and (b) diphenyl methyl radical trapped with TTBNB (anilino-type).

Figure S15(a) indicates the ESR spectrum (triple-triplet) from the spin-adduct of the silyl radical trapped with TTBNB. The hyperfine splitting (*hfs*) constant A_N due to nitrogen (the spin quantum number I = 1) was 1.03 mT, and the small splitting A_{Hm} due to two hydrogens (I = 1/2) at *meta* position of TTBNB benzene ring was 0.175 mT. The *g*-value of 2.0047 was assigned to the anilino-type radical[40], as shown in Figure S16(a). The spectrum Figure S15(b) (double-triplet; sextet line) was assigned to the benzyl type radicals trapped with TTBNB. The *hfs* constant A_N and $A_{H\alpha}$ due to an alpha proton were 0.62 and 0.34 mT, respectively. The splitting due to the meta hydrogens was too small to be resolved (less than 0.06 mT). The *g*-value of 2.00266 was assigned to the anilino-type radical, as shown in Figure S16(b).[41]

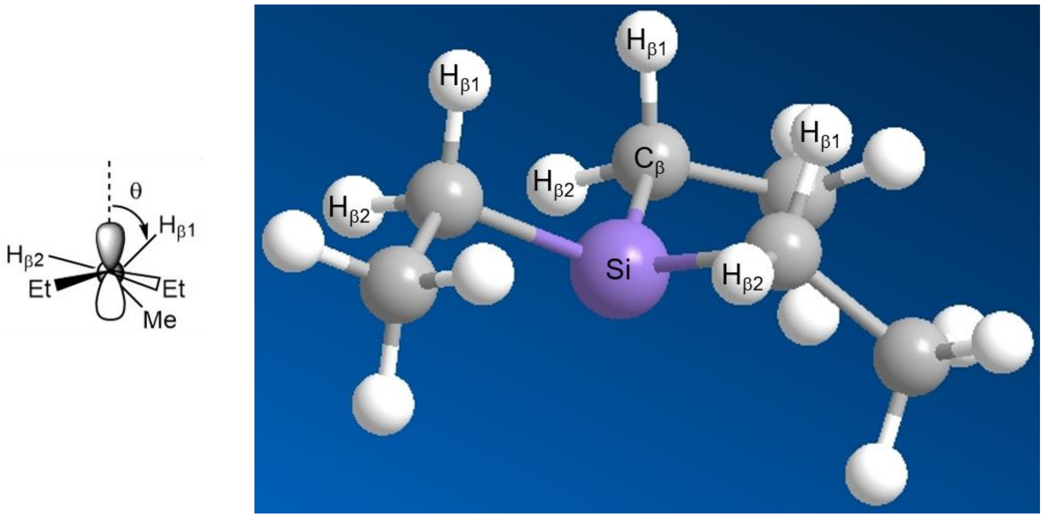

Figure S17. Illustration of the Newman projection of the Siα-C$_\beta$ bond (left) and the steric structure of triethylsilyl radical after energy minimization (right).

Figure S15(c) indicates the quartet line with the splitting constant of 0.57 mT. The relative intensity ratio of 1 : 3 : 3 : 1 means the radical has three equivalent nuclei. The ^{28}Si (I = 0) and ^{29}Si (I = 1/2) radicals essentially give singlet and doublet peaks, respectively. Since the natural abundance of ^{29}Si was ~ 4%, the signal due to ^{29}Si was buried in noise level in this experiment. We suggested that this signal can be assigned to the $Si(Et)_3$ radicals. The *hfs* constant due to hydrogens H_β on the carbon (β-position) atoms connected to the silicon atom depends on the torsional angle between the axis of the *p*-orbital of the unpaired electron on the silyl radical and the C_β-H_β bond axis in the Newman projection of the Si_α-C_β bond as shown in Figure S17. The dihedral angle is θ formed by the projection of the Si_α-C_β bond on the axis of the *p*-orbital of the unpaired electron. The *hfs* constants were assumed to be given by the following equation. This form is the well-established empirical relation carbon-center radical species.[42]

$$A_{H_\beta} = B_0 + B_2 cos^2\theta \sim B_2 cos^2\theta$$

The values of B_0 are typically small to be ignored for simplicity (still unknown). The *hfs* constant of the A_{H_β} was reported to be 0.642 mT. In the report, the ESR spectrum of trimethylsilyl radicals was obtained at 203K by hydrogen abstraction of $SiH(CH_3)_3$ by the initially produced *tert*-butoxy radicals. The presence of nine equivalent protons gave the 10-lines spectrum, which means all protons of the methyl groups indicate the same *hfs* value of 0.642 mT where the free rotation of the methyl groups is permitted (faster than measurement time scale). In such a situation, the term of $cos^2\theta$ is averaged to be 0.5, $\langle cos^2\theta \rangle = 0.5$. Thus, the value of B_2 is approximately 1.28 mT. From our experiment, since the value of the *hfs* constant was 0.57 mT, the dihedral angle θ is calculated to be 48° ($H_{\beta 1}$). For one other proton ($H_{\beta 2}$), the dihedral angle is predicted to be -72° (120° apart from $H_{\beta 1}$), giving the *hfs* of 0.12 mT. In this case, the quadra quartet lines would be observed if highly resolved. Experimentally, however, the spectrum was a simple quartet line (a small undefined peak was observed, though). That means the values of the dihedral angles and constant B_2 are inappropriate, and/or different environmental situations surrounded the radicals affected on the constants, including g-value, which is a higher value (2.006) than the reported value of trimethylsilyl radicals (2.003)[43]. Diglyme solution (polar solvent) was used in our case. Here, we will consider the effect of dihedral angle on the ESR spectrum, although we are not sure about the influence of the solvent on the inherent values so far. We carried out the calculation of the energy minimization of the steric structure of triethylsilyl radical using Chem3D, the obtained structure was shown in Figure S17. From the resulted structure, the dihedral angle of $H_{\beta 1}$ was small (C_β-$H_{\beta 1}$ bond axis is nearly parallel to the *p*-orbital). On the other hand, the dihedral angle of $H_{\beta 2}$ is around 90°, resulting in the *hfs* of zero. Therefore, three $H_{\beta 1}$ do not contribute to the splitting. We concluded that the quartet line spectrum could be attributed to three equivalent protons $H_{\beta 1}$ in our case.

7. Unsuccessful Substrates for the Cross-Coupling Reaction

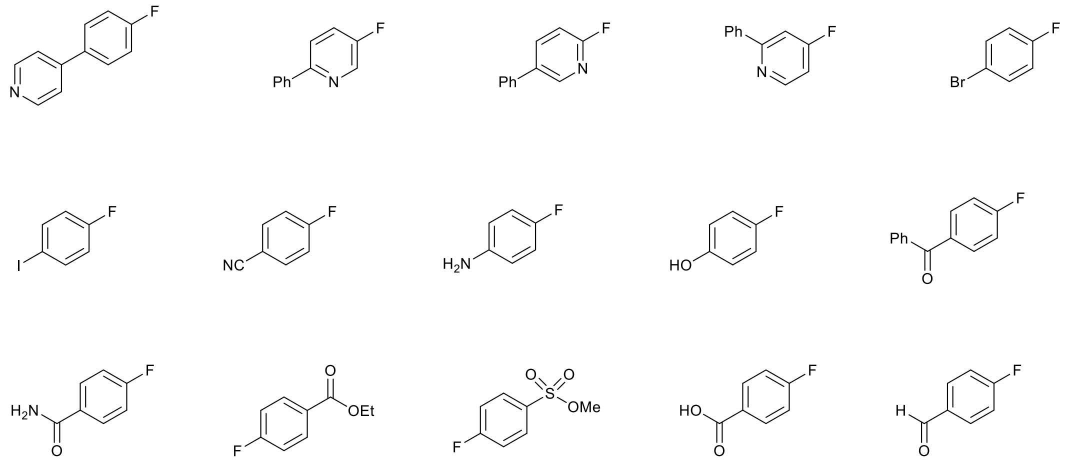

Figure S18. Unsuccessful organic fluorides.

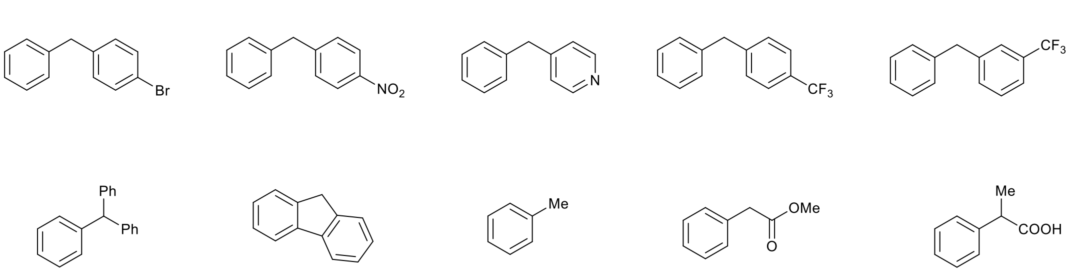

Figure S19. Unsuccessful benzylic C–H bond containing compounds.

8. NMR Spectra (^{1}H NMR, ^{13}C NMR and ^{19}F NMR)

2-(4-Fluorophenyl)benzofuran (1q)

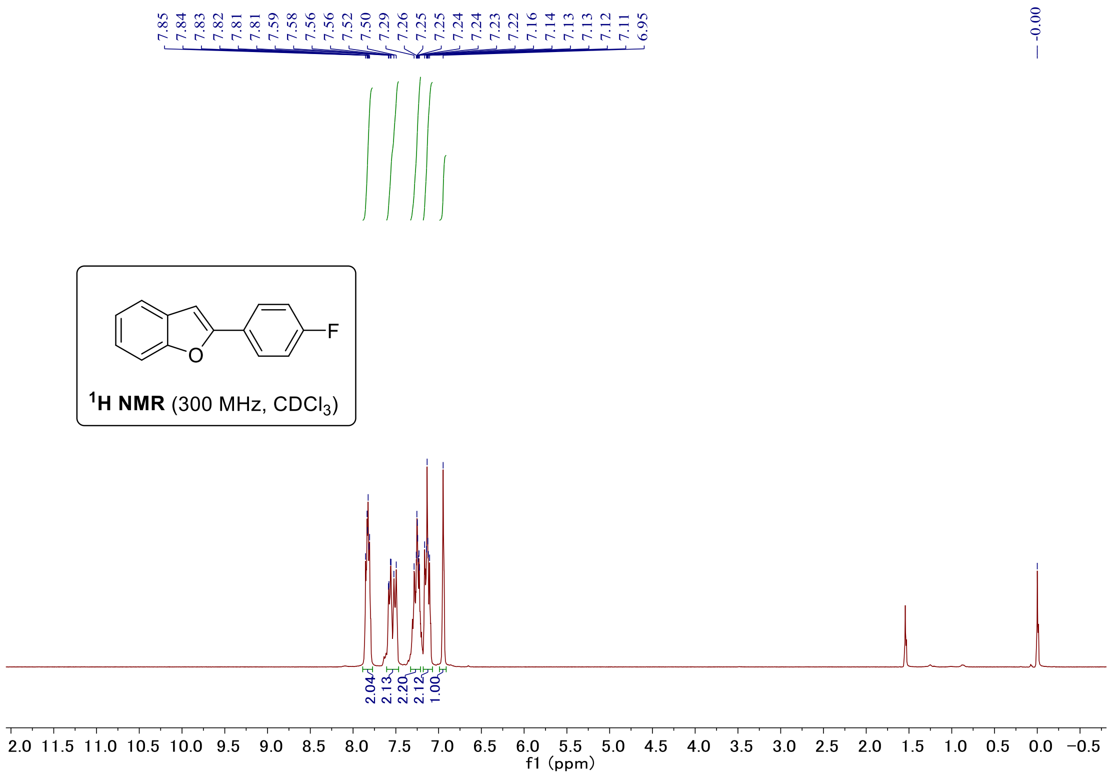

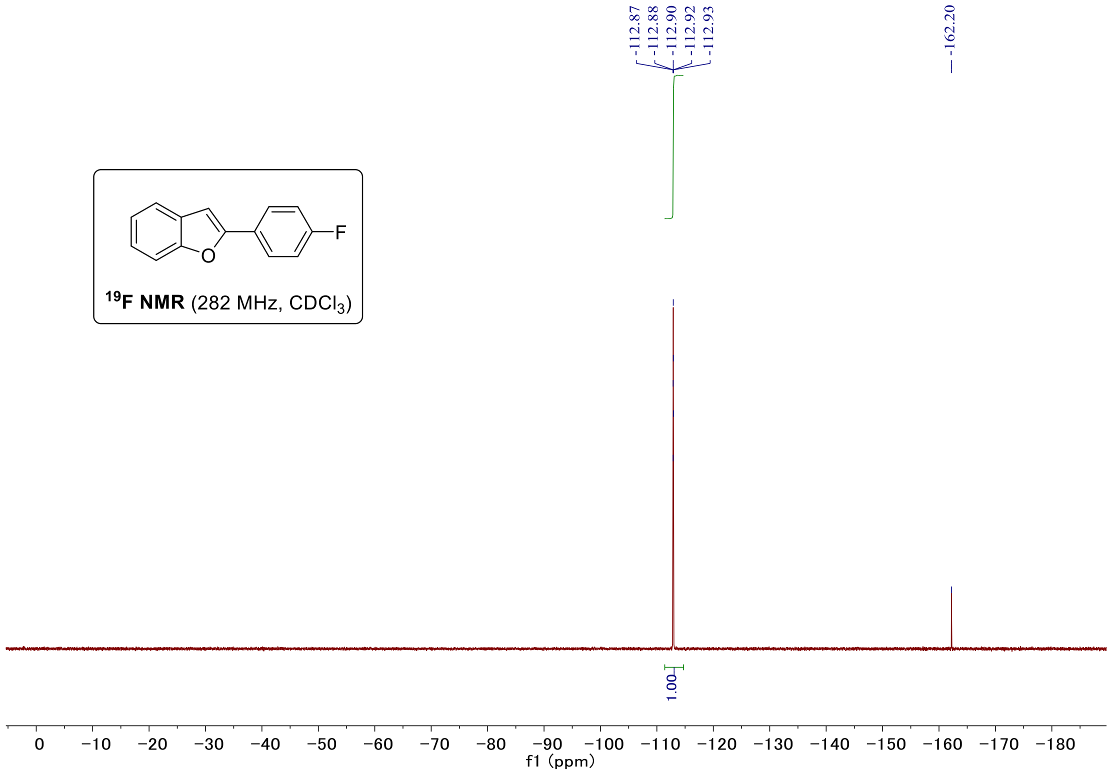

9-fluorophenanthrene (1s)

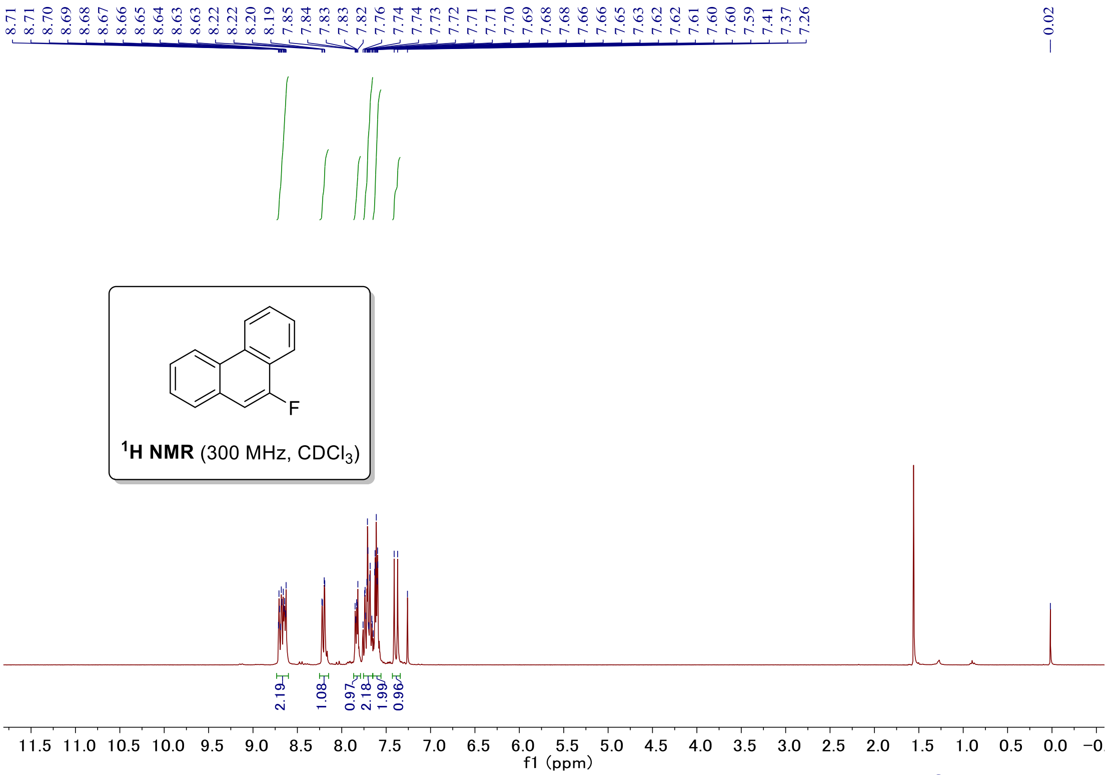

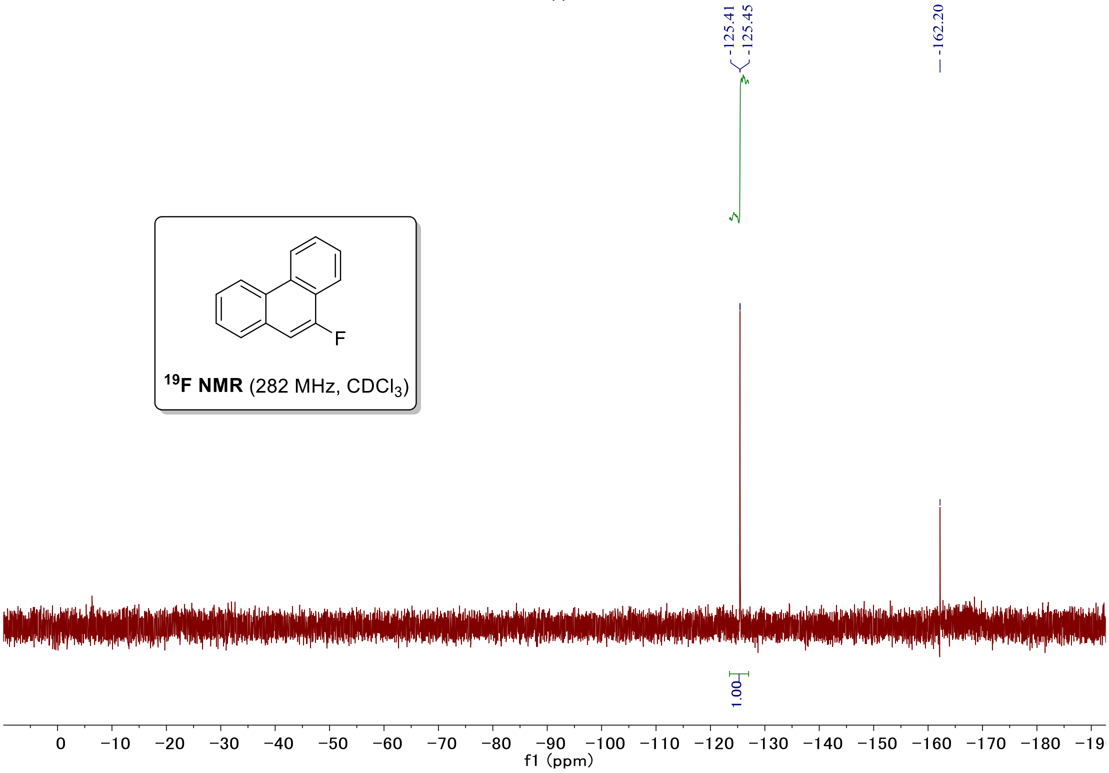

3-(But-3-en-1-yl)-4-fluorobiphenyl (1u)

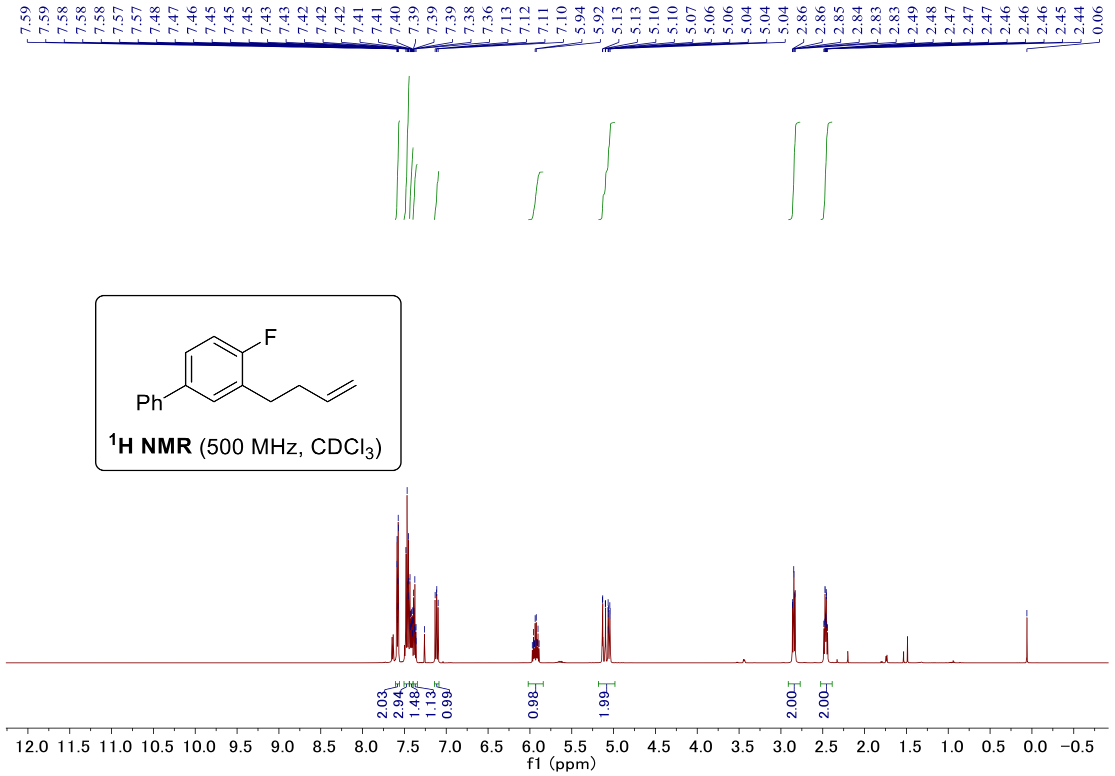

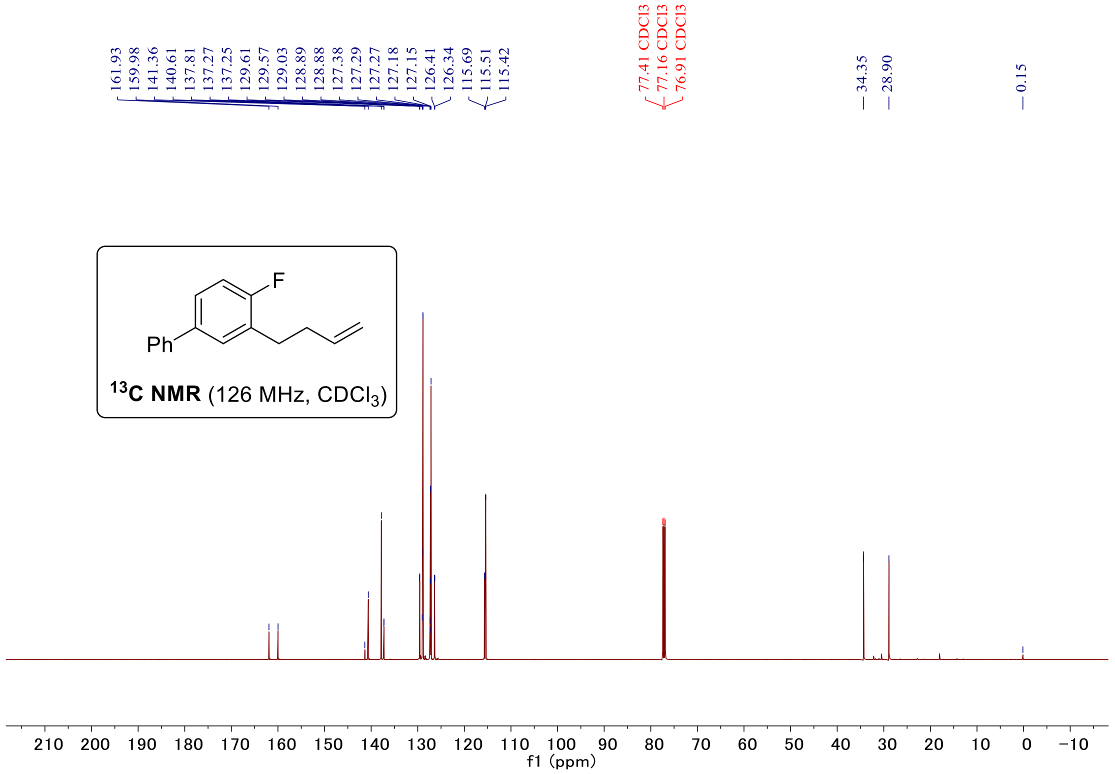

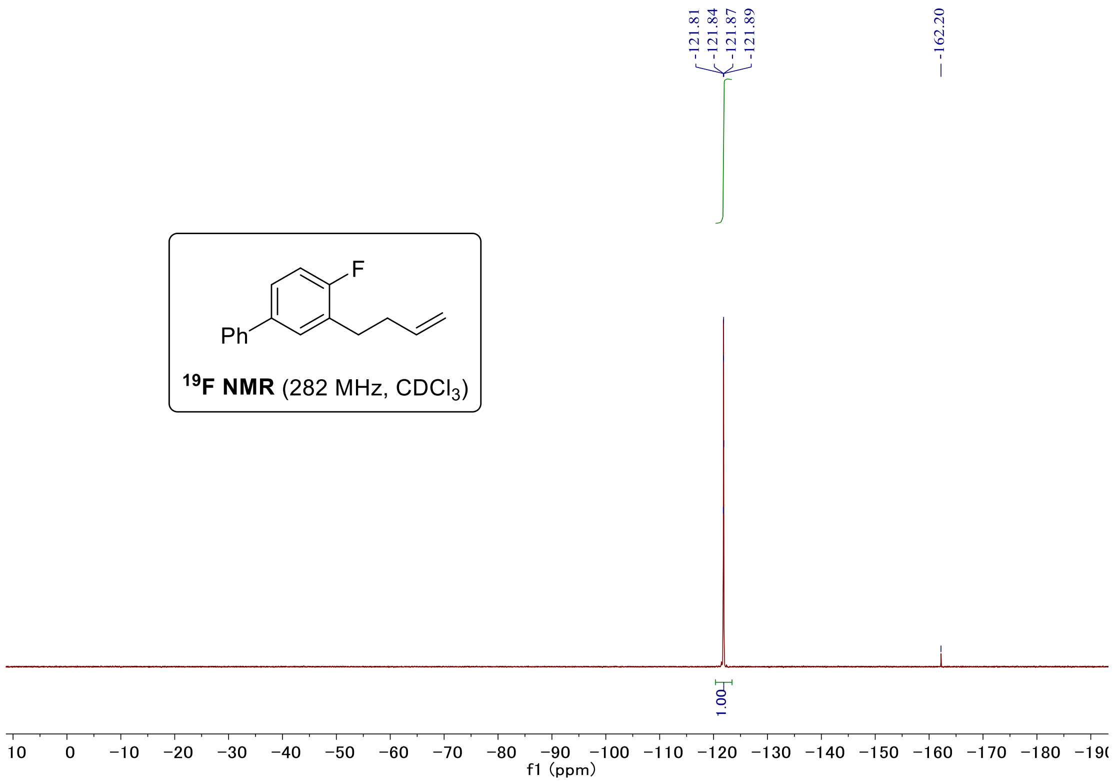

(3-(2-Fluorophenyl)propane-1,1-diyl)dibenzene (1v)

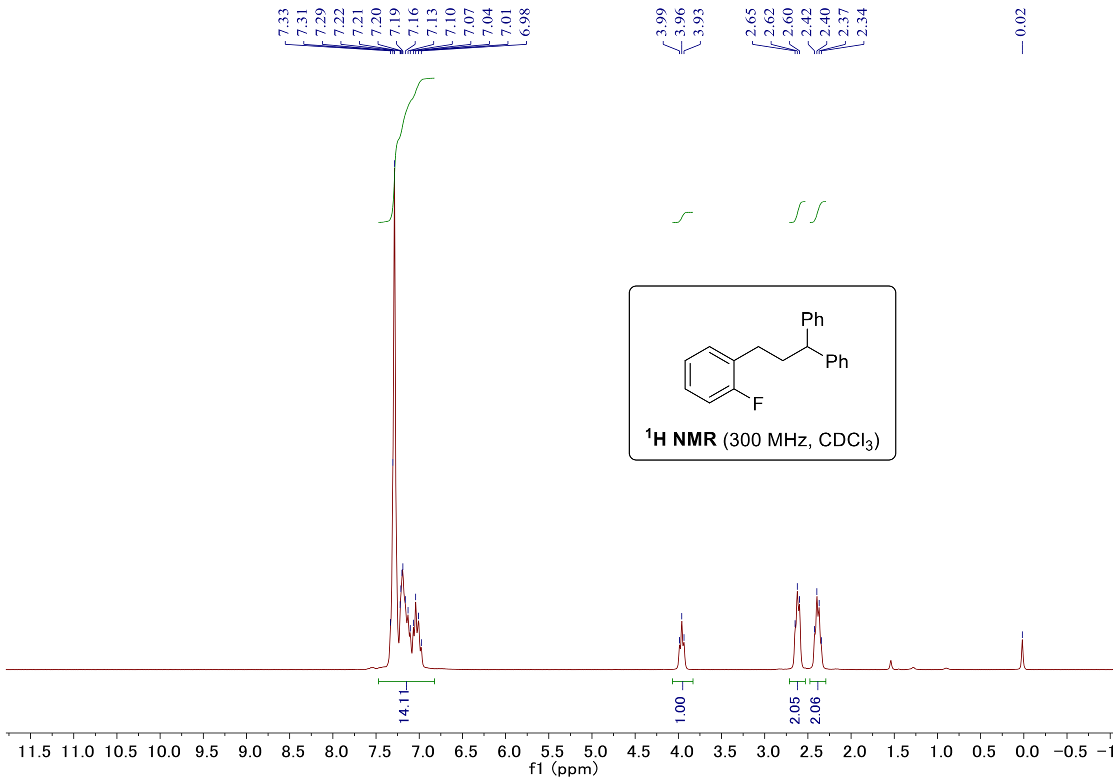

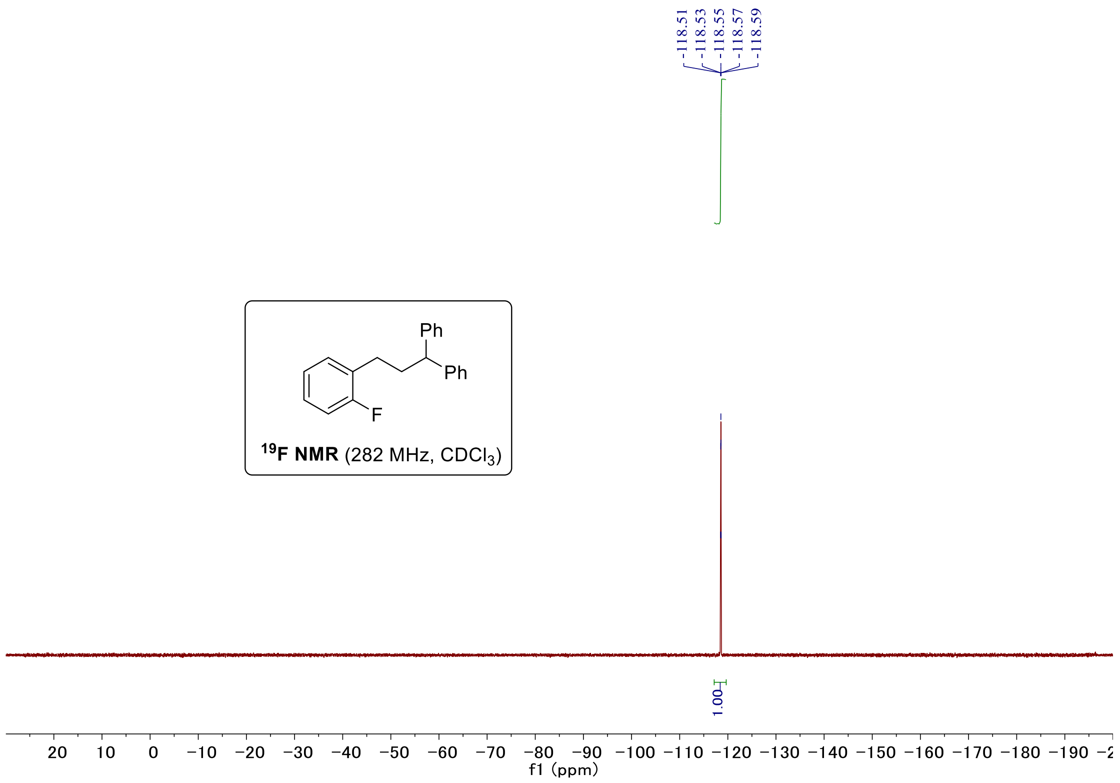

Diphenylmethane (2a)

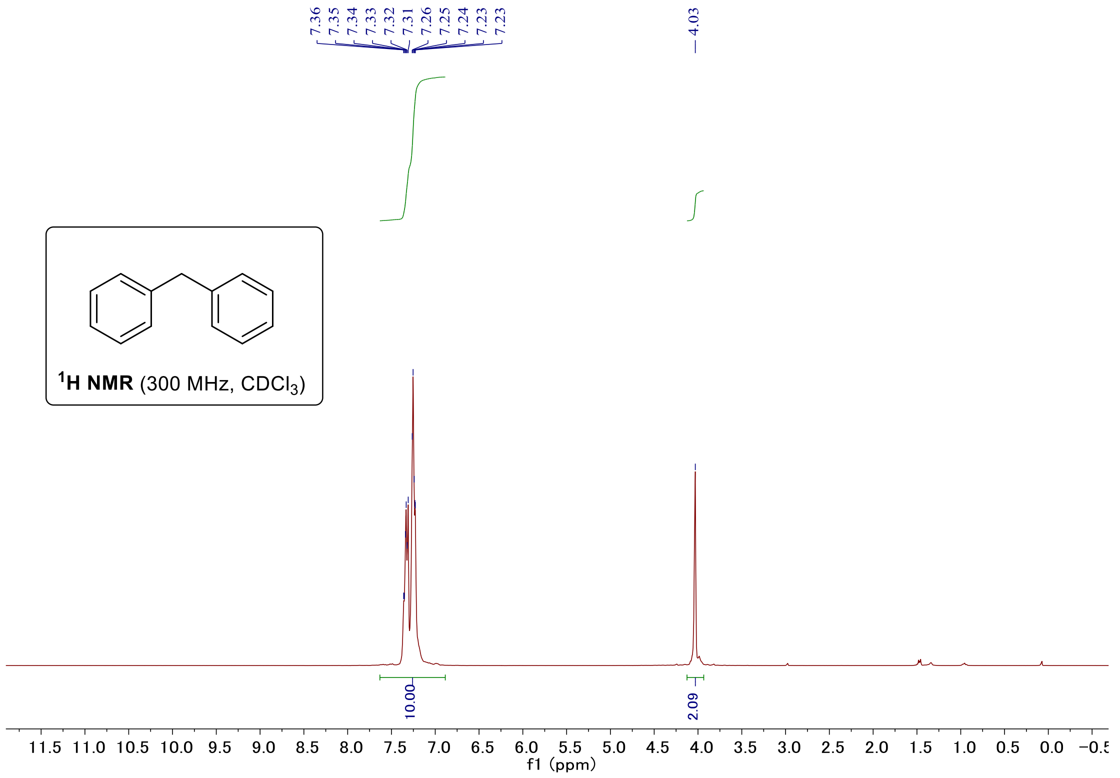

1-Benzyl-4-methoxybenzene (2b)

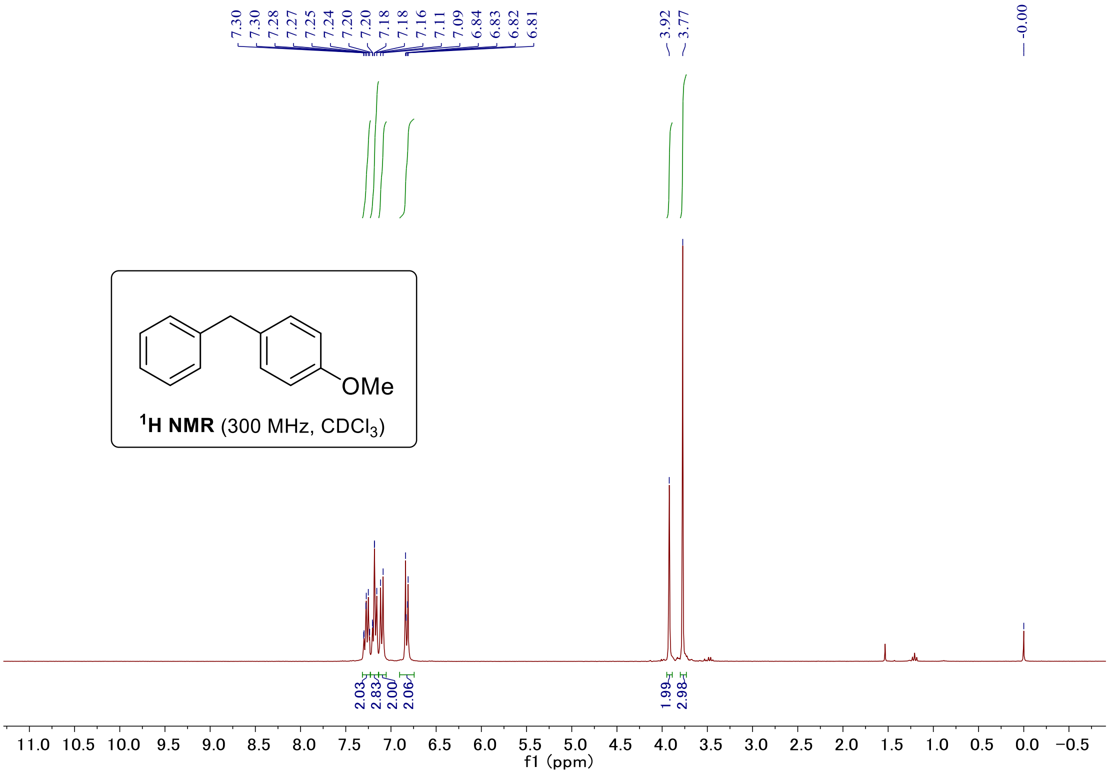

Dianisylmethan (2c)

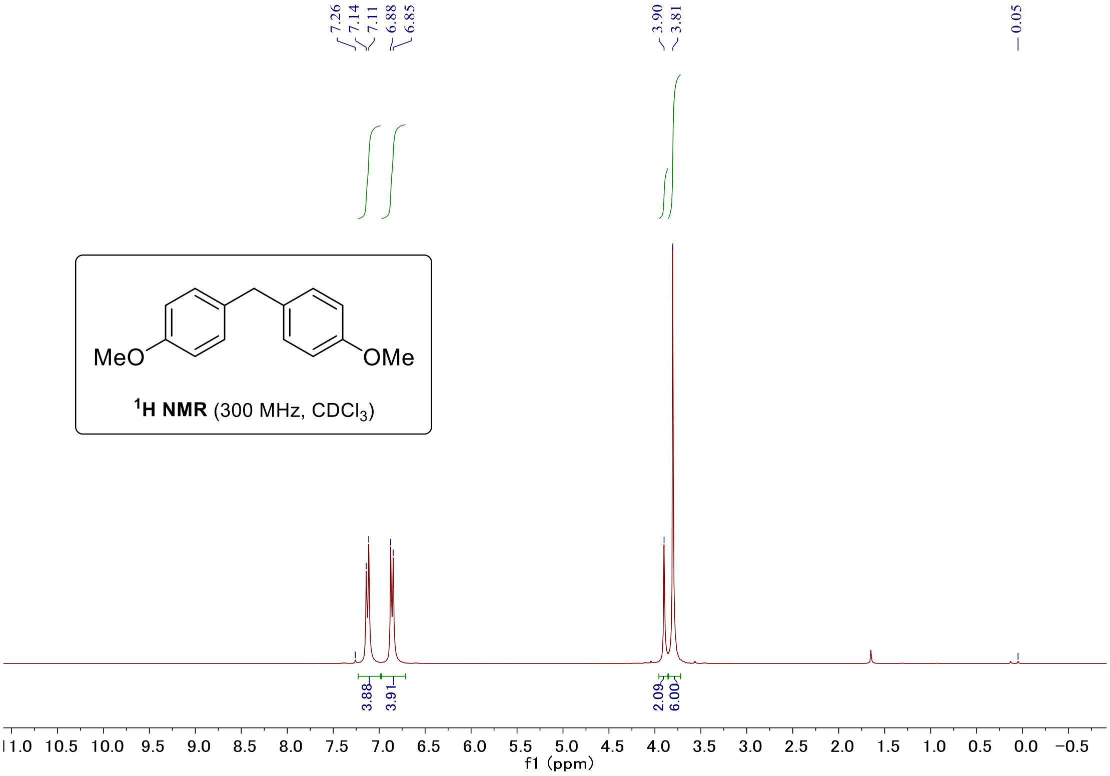

Di-*p*-tolylmethane (2d)

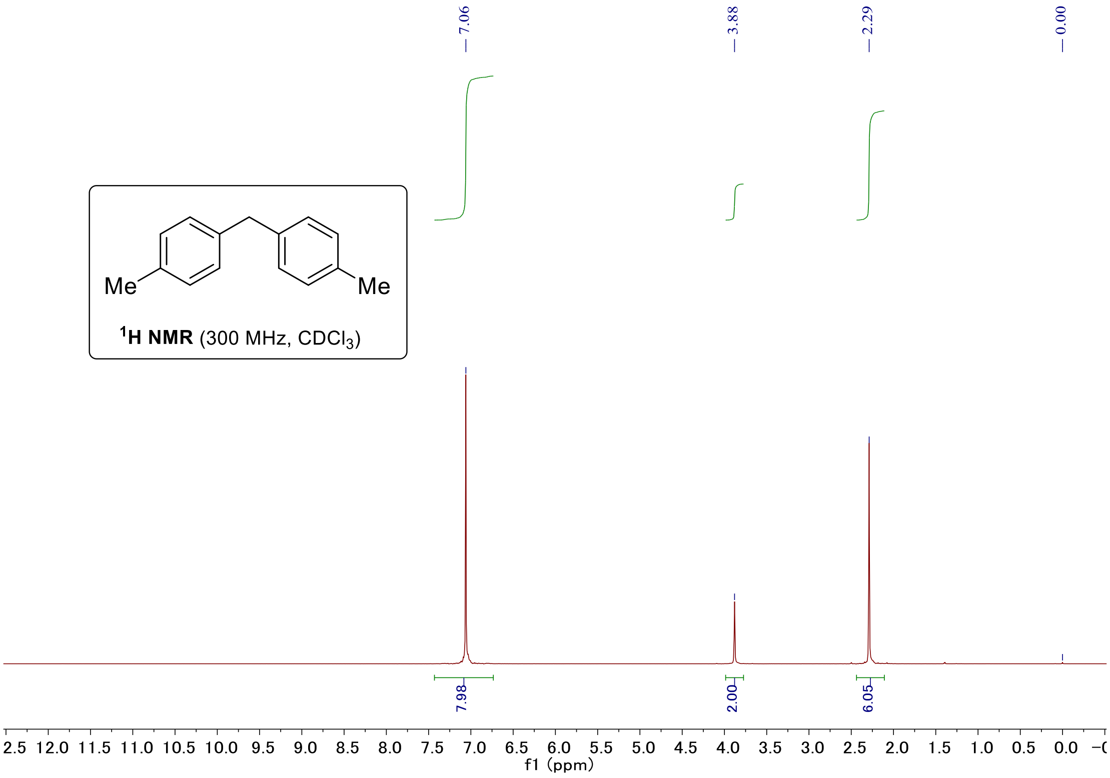

1-Benzyl-2-methylbenzene (2e)

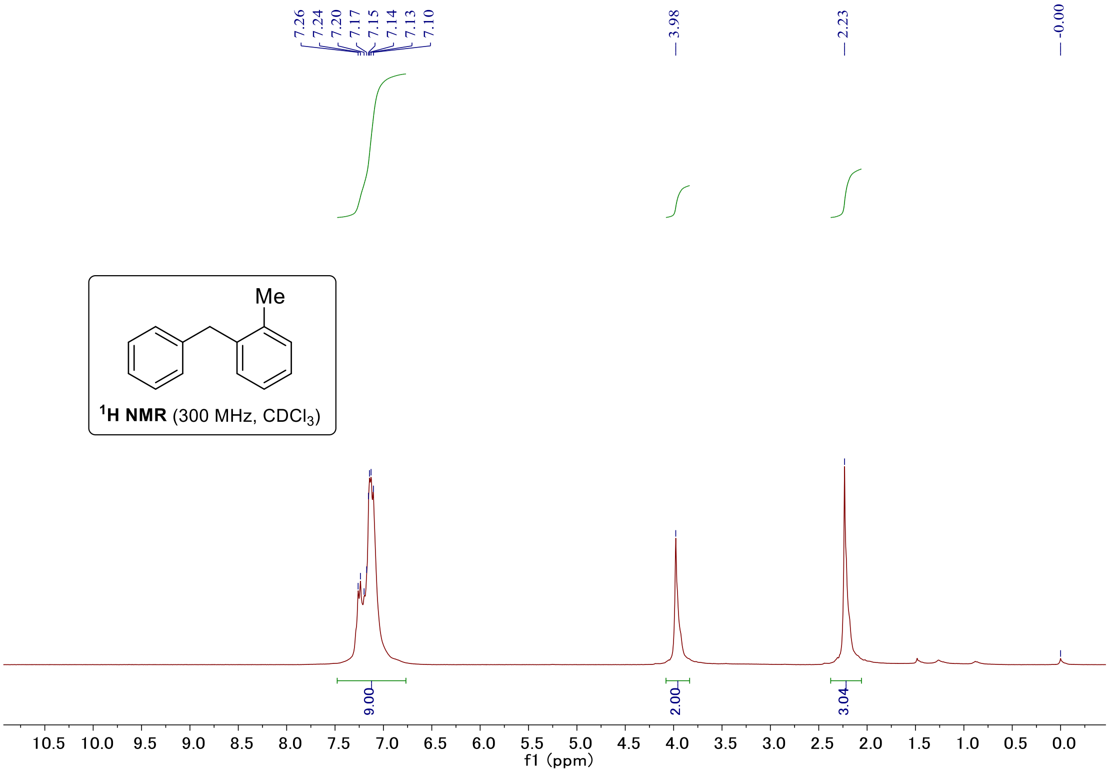

1-Benzyl-4-chlorobenzene (2f)

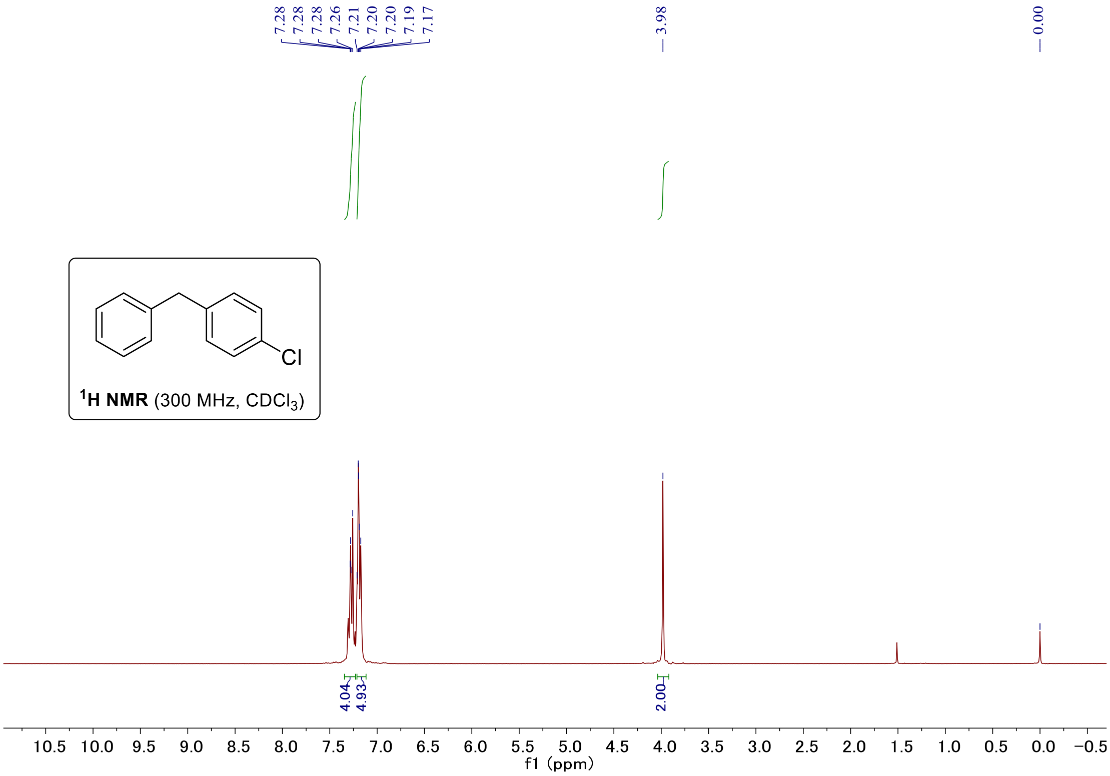

2-Benzylthiophene (2g)

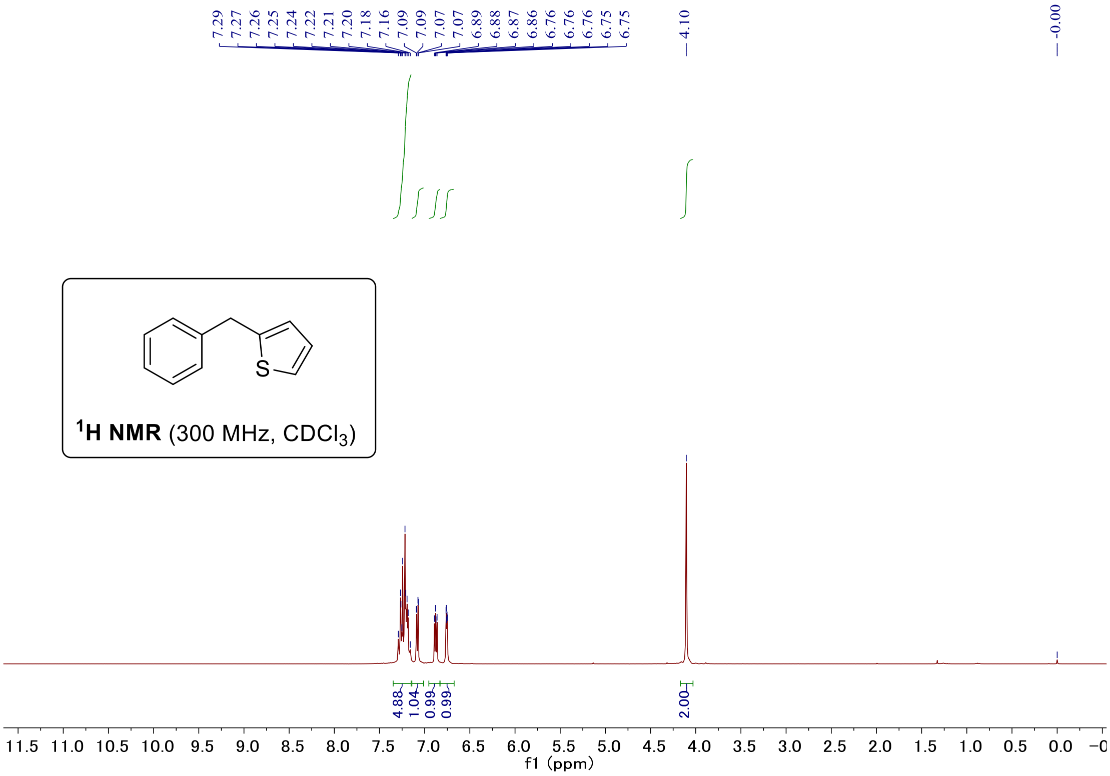

9*H*-Fluorene

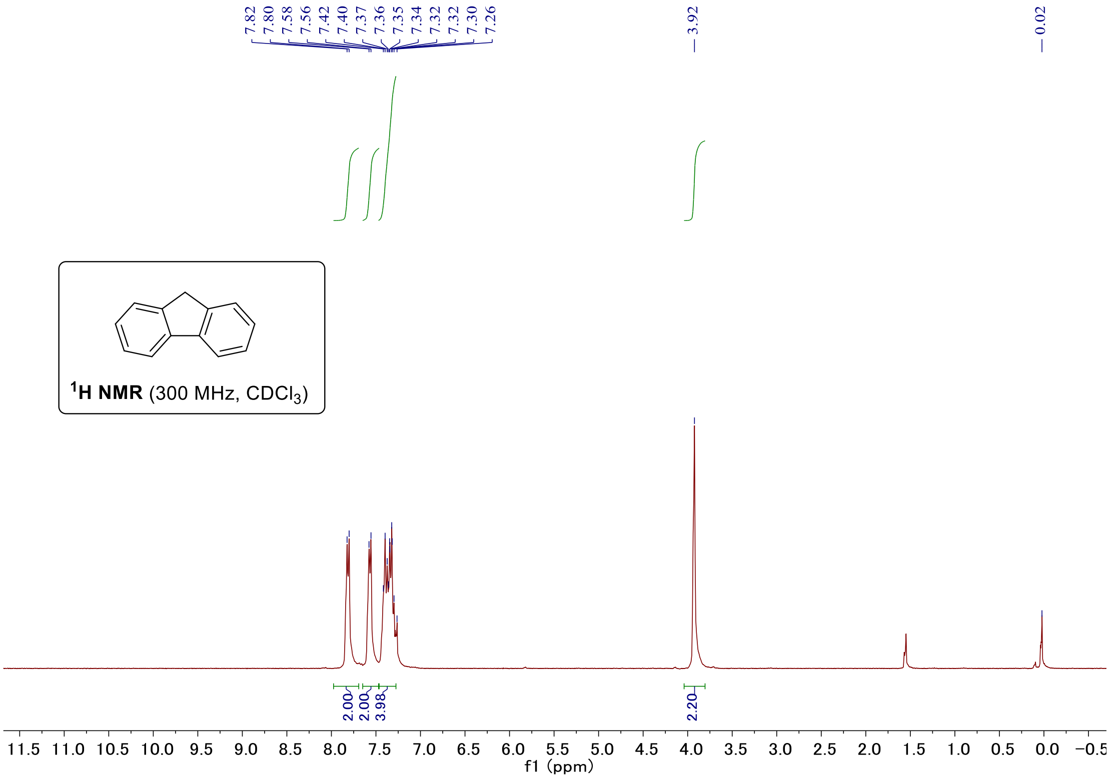

Propane-1,1-diyldibenzene (2k)

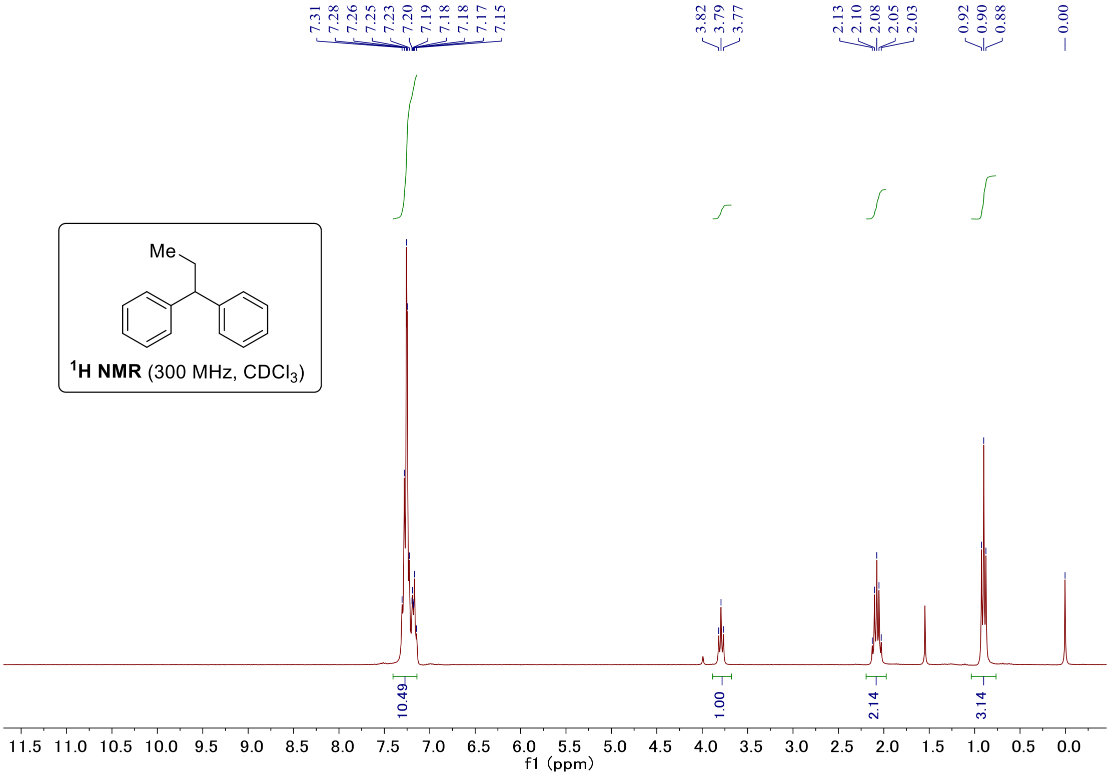

Pentane-1,1-diyldibenzene (2l)

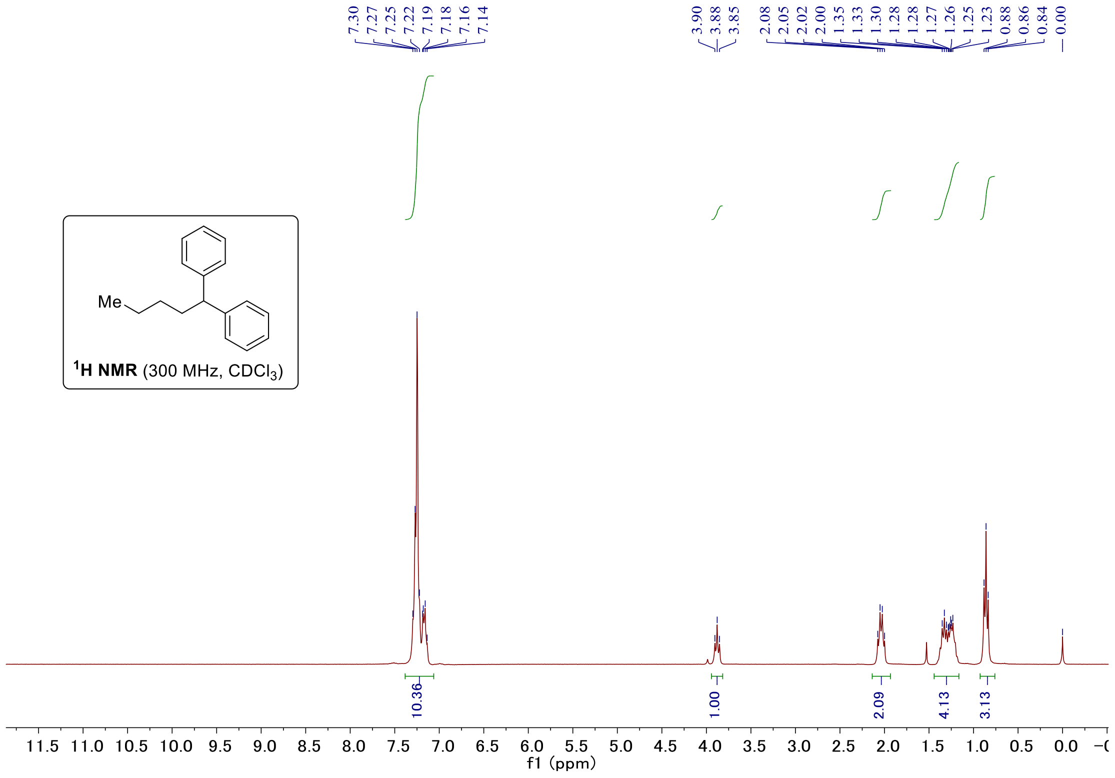

(8*R*,9*S*,13*S*,14*S*)-3,17-dimethoxy-13-methyl-7,8,9,11,12,13,14,15,16,17-decahydro-6*H*-cyclopenta[*a*]phenanthrene (2s)

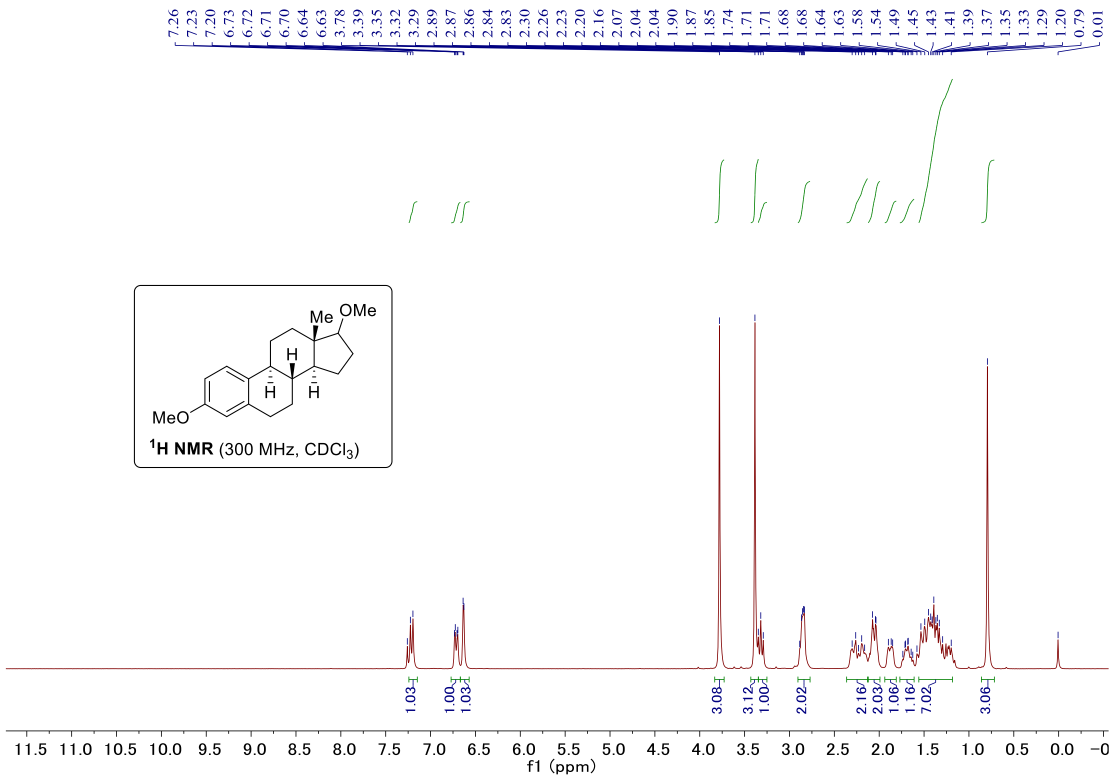

2-(4-(4-Methoxybenzyl)phenoxy)-*N*,*N*-dimethylethan-1-amine (2t)

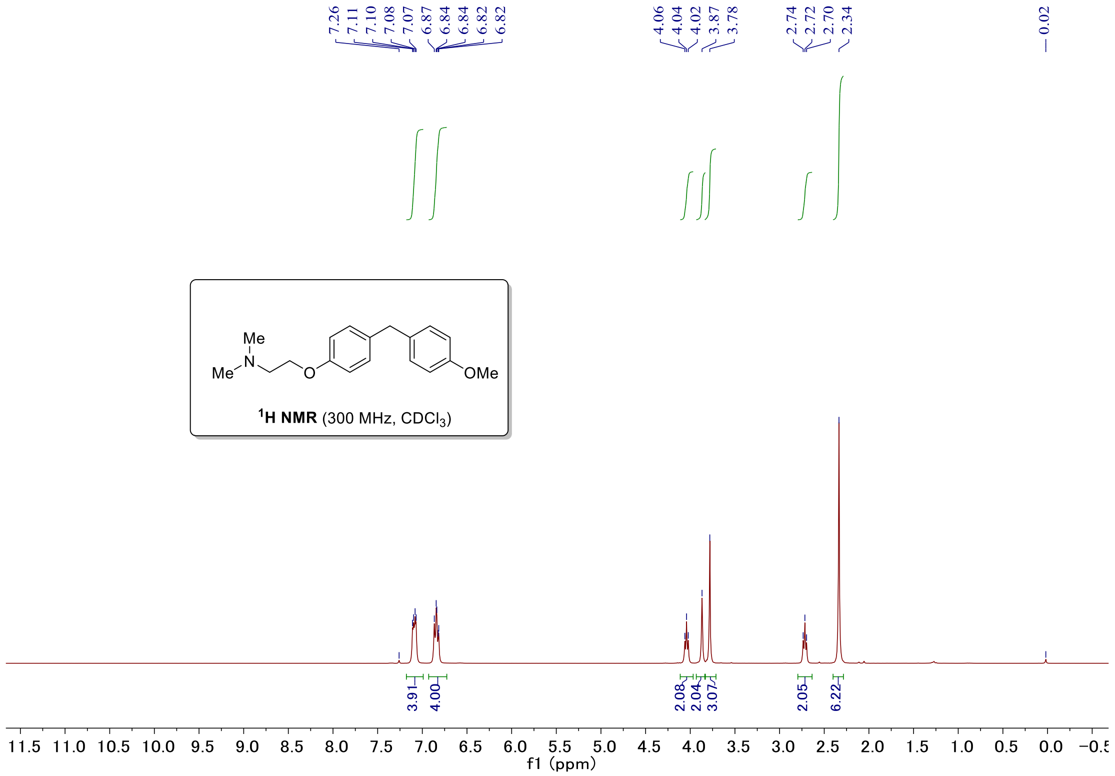

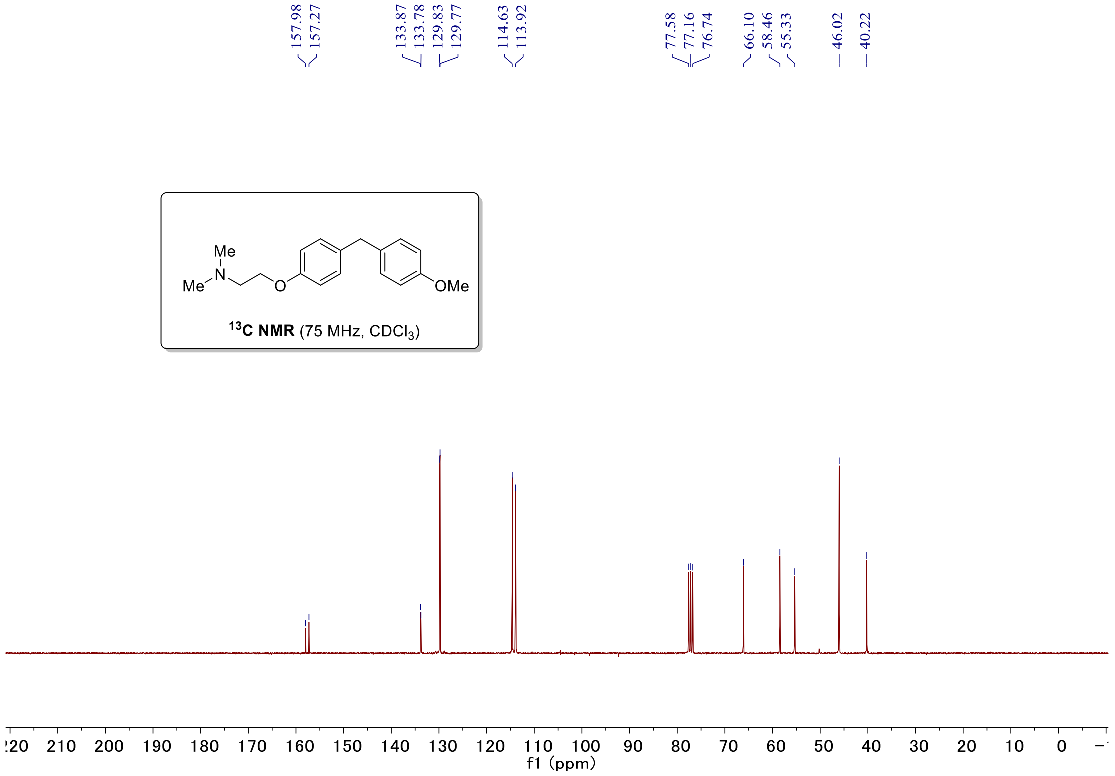

1-(Cyclopropyl(phenyl)methyl)-4-methoxybenzene (2u)

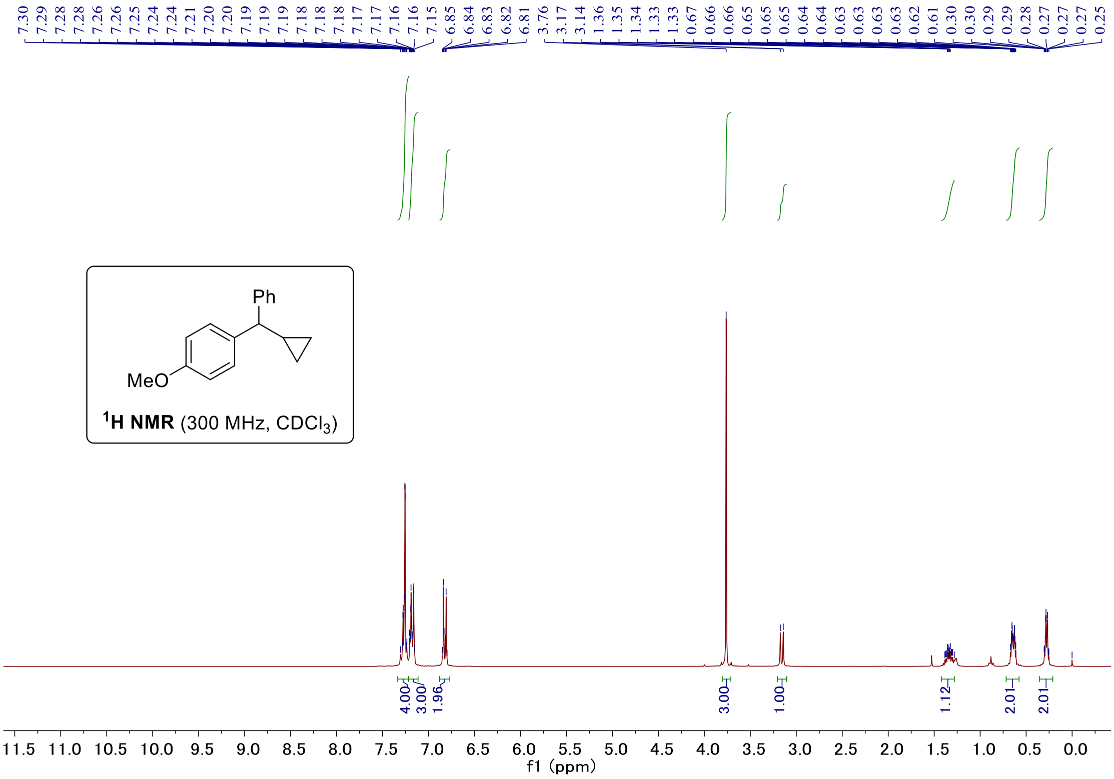

4-Benzhydrylbiphenyl (3aa)

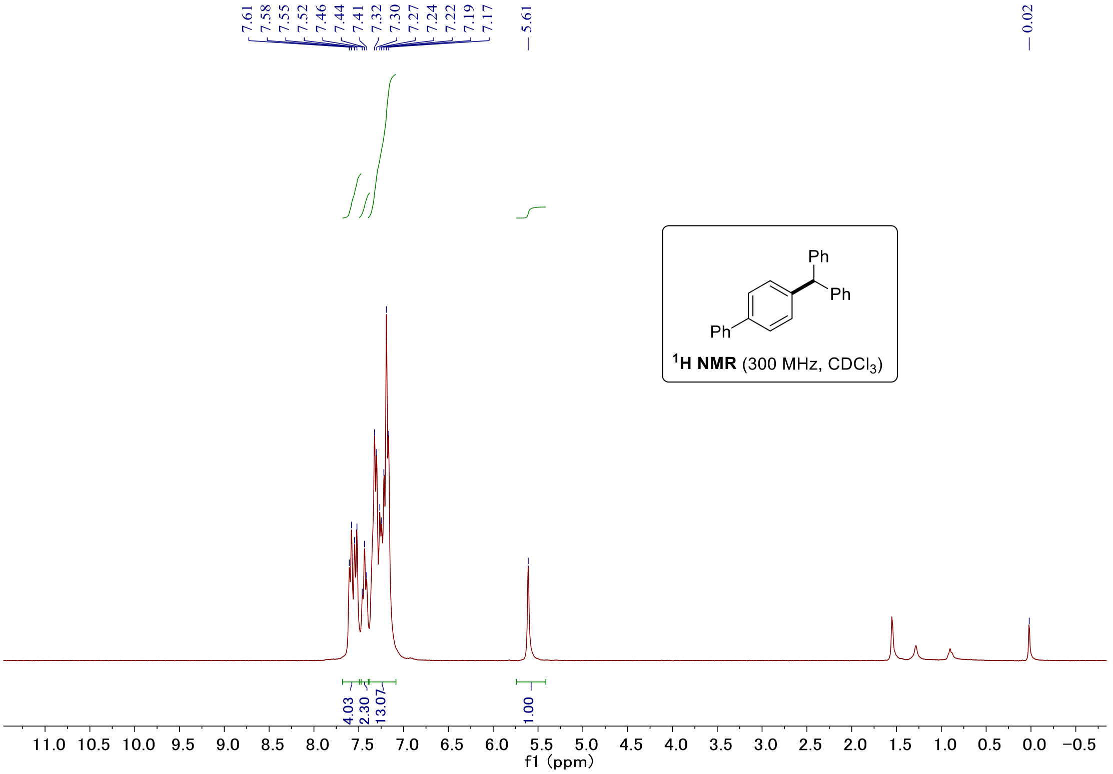

3-Benzhydrylbiphenyl (3ba)

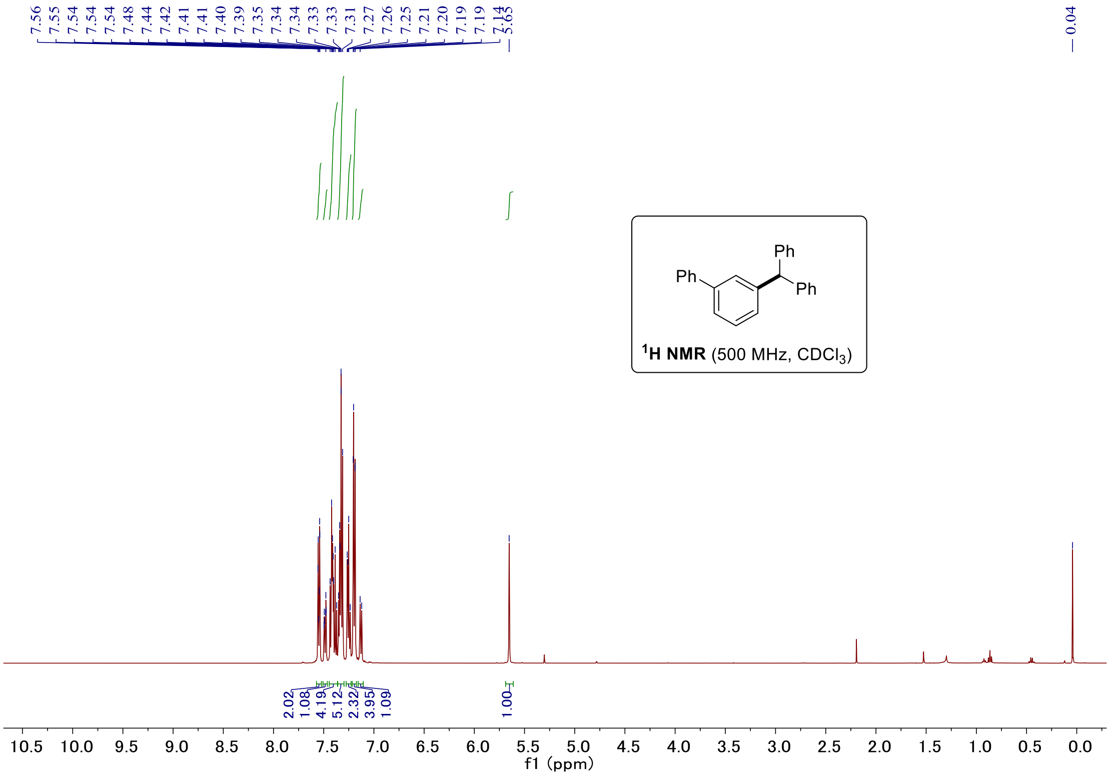

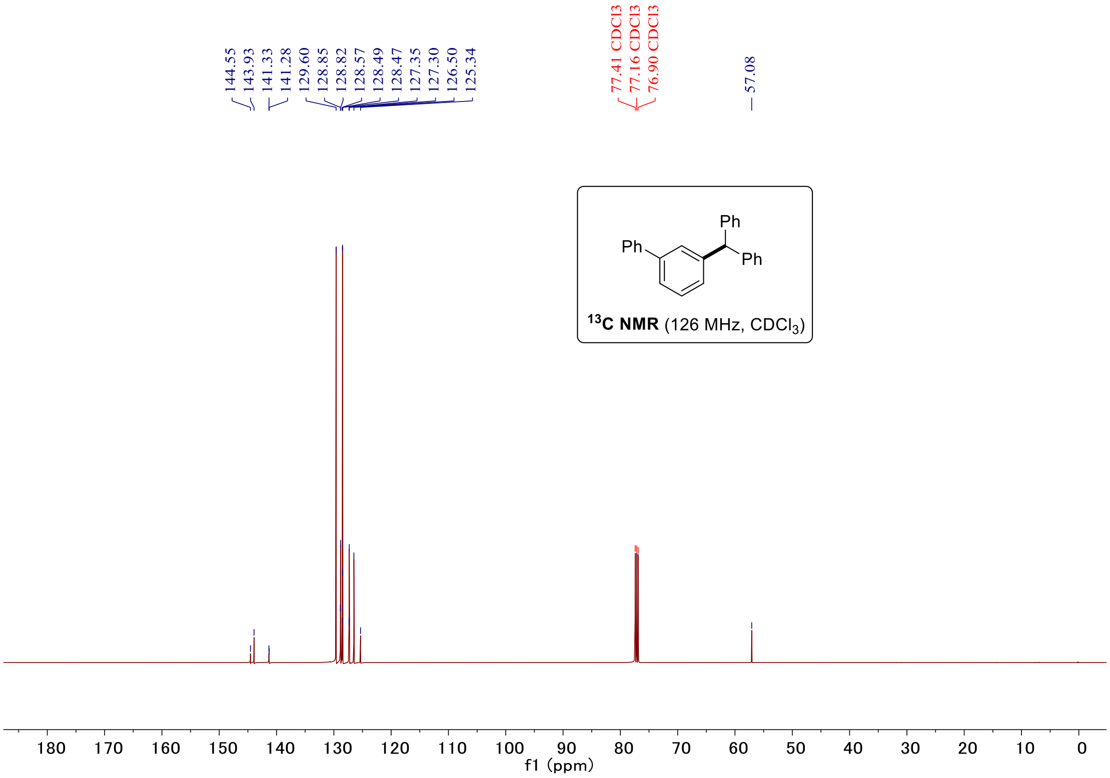

2-Benzhydrylbiphenyl (3ca)

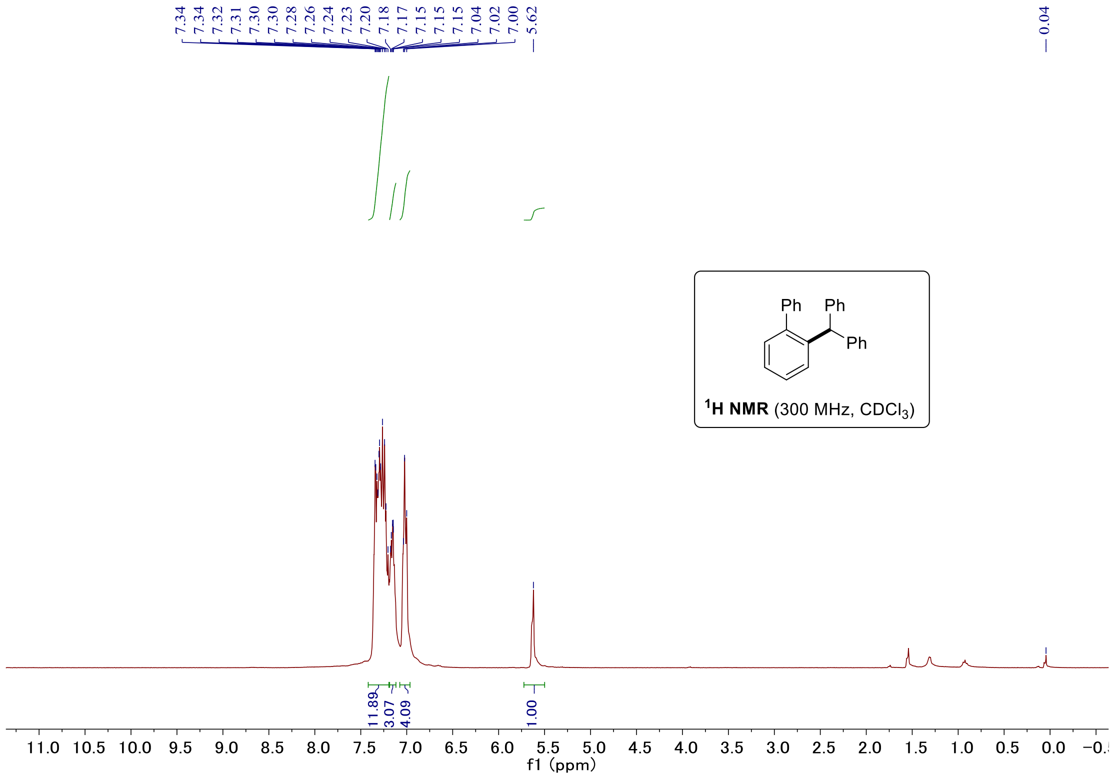

1-Benzhydrylnaphthalene (3da)

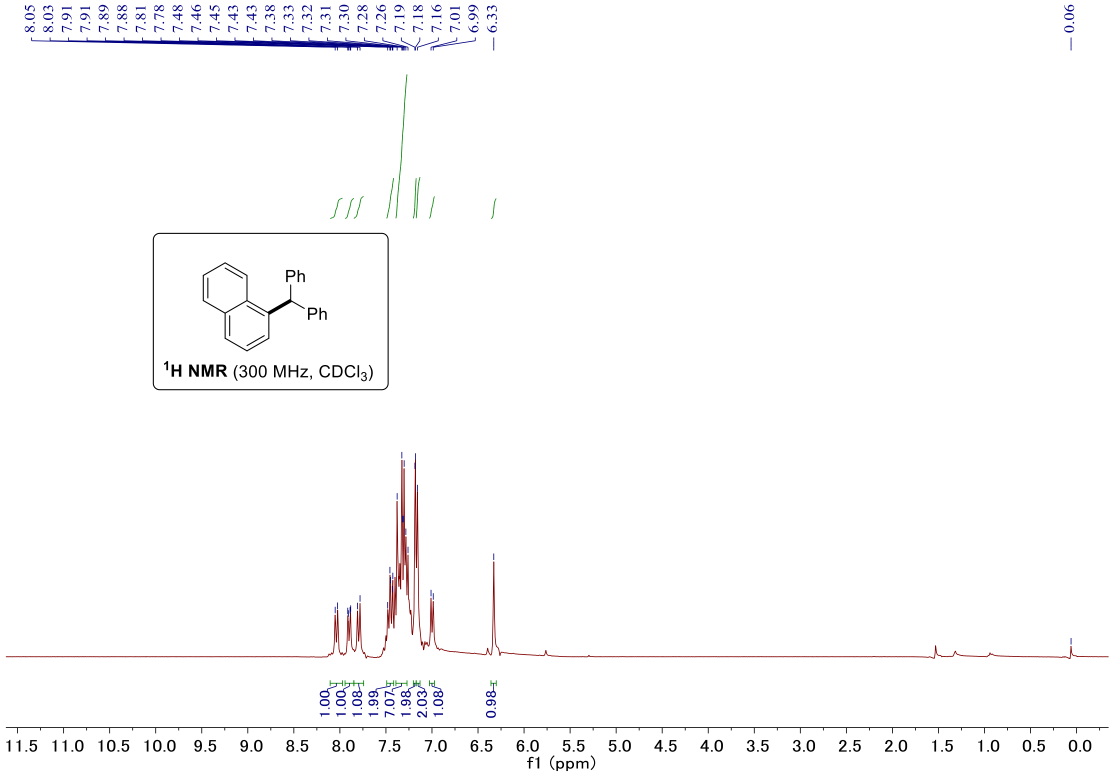

Triphenylmethane (3ea)

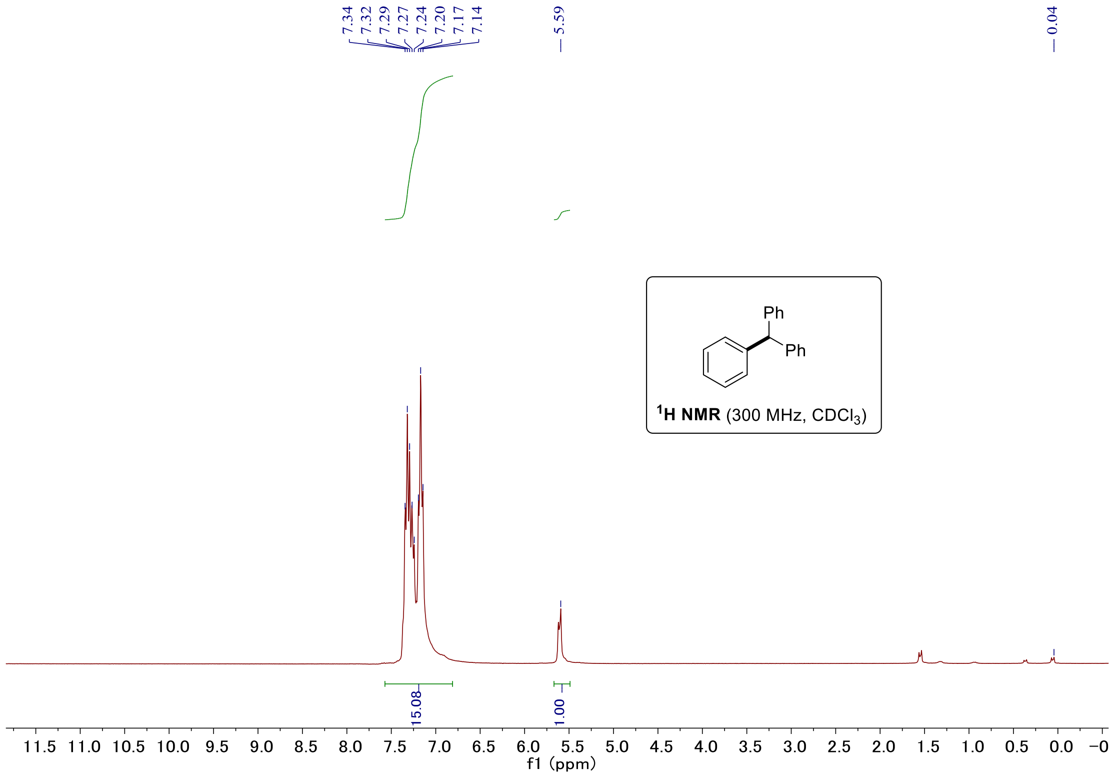

1-Benzhydryl-4-methylbenzene (3fa)

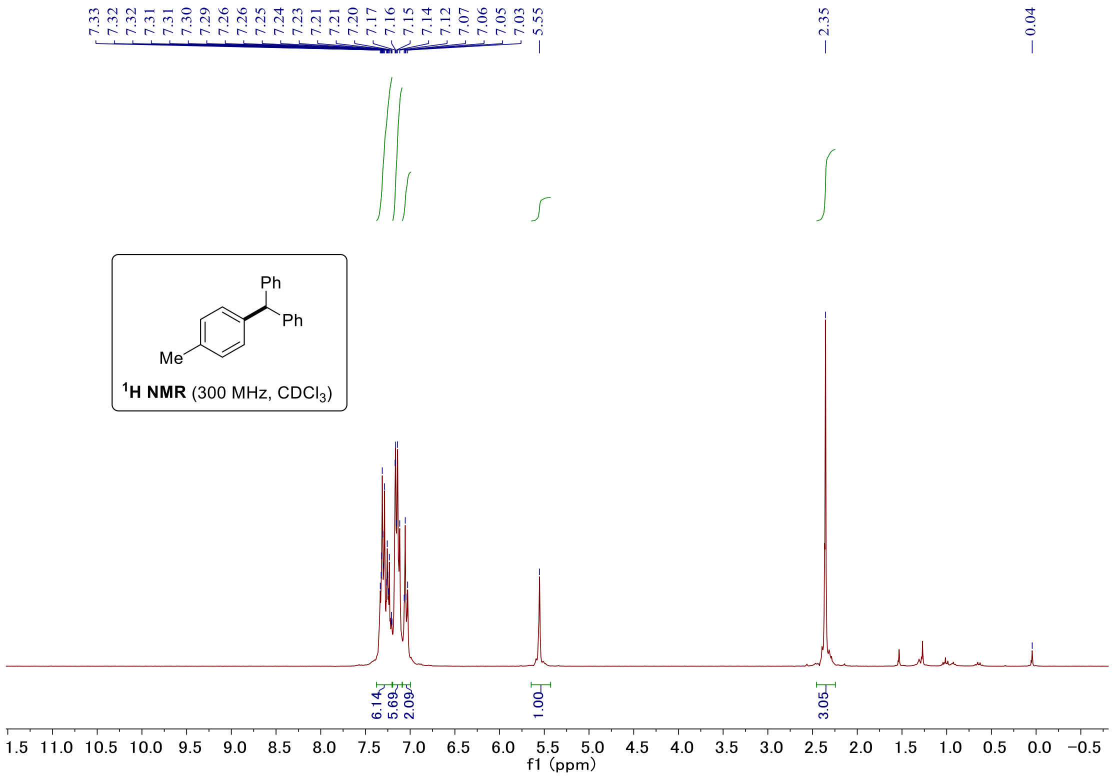

4-Benzhydrylanisole (3ga)

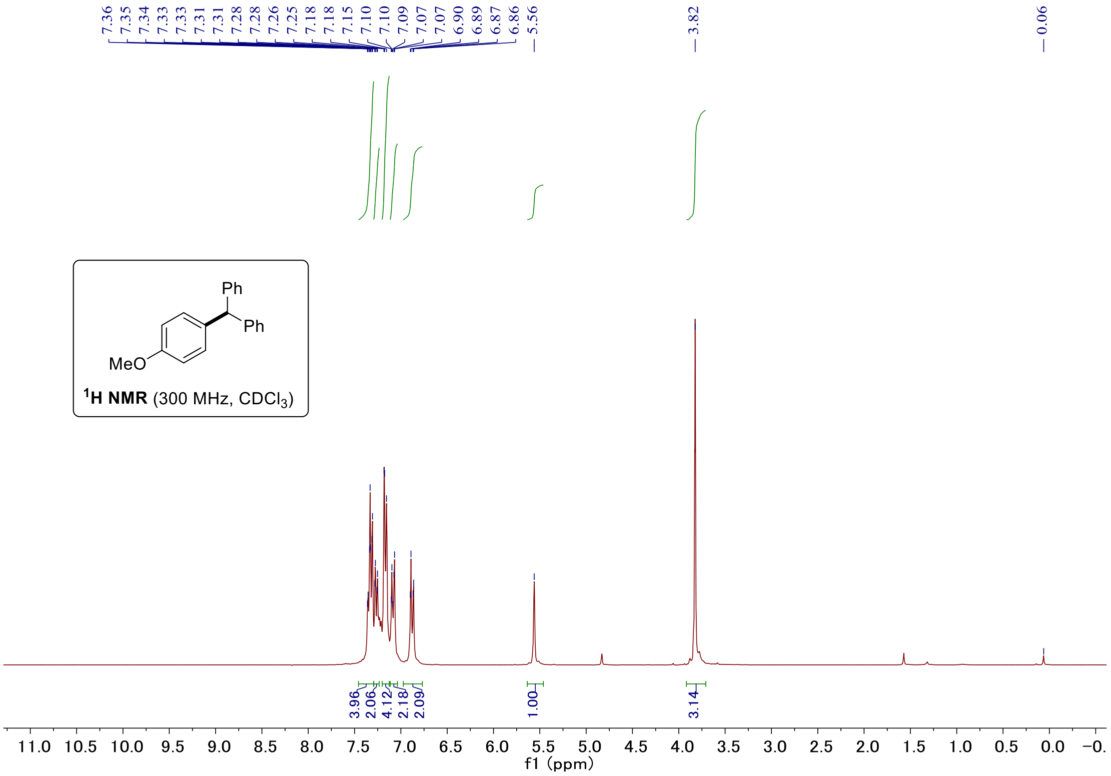

3-Benzhydrylanisole (3ha)

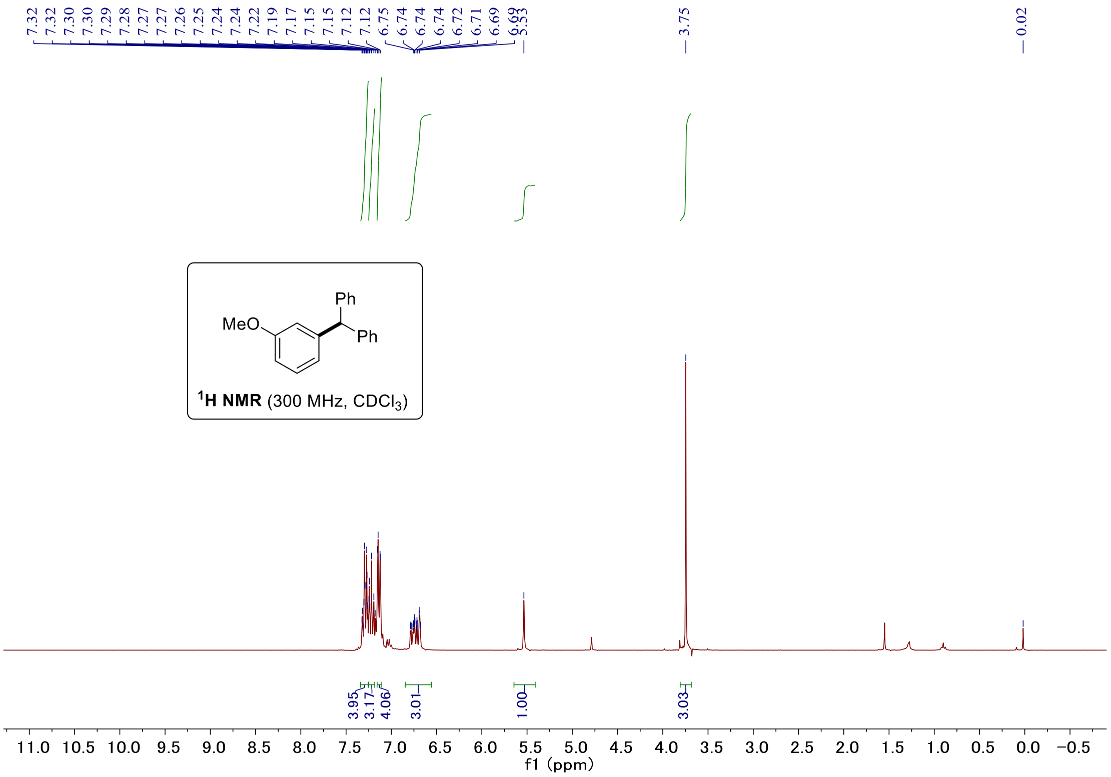

1-(4-Benzhydrylphenyl)naphthalene (3ia)

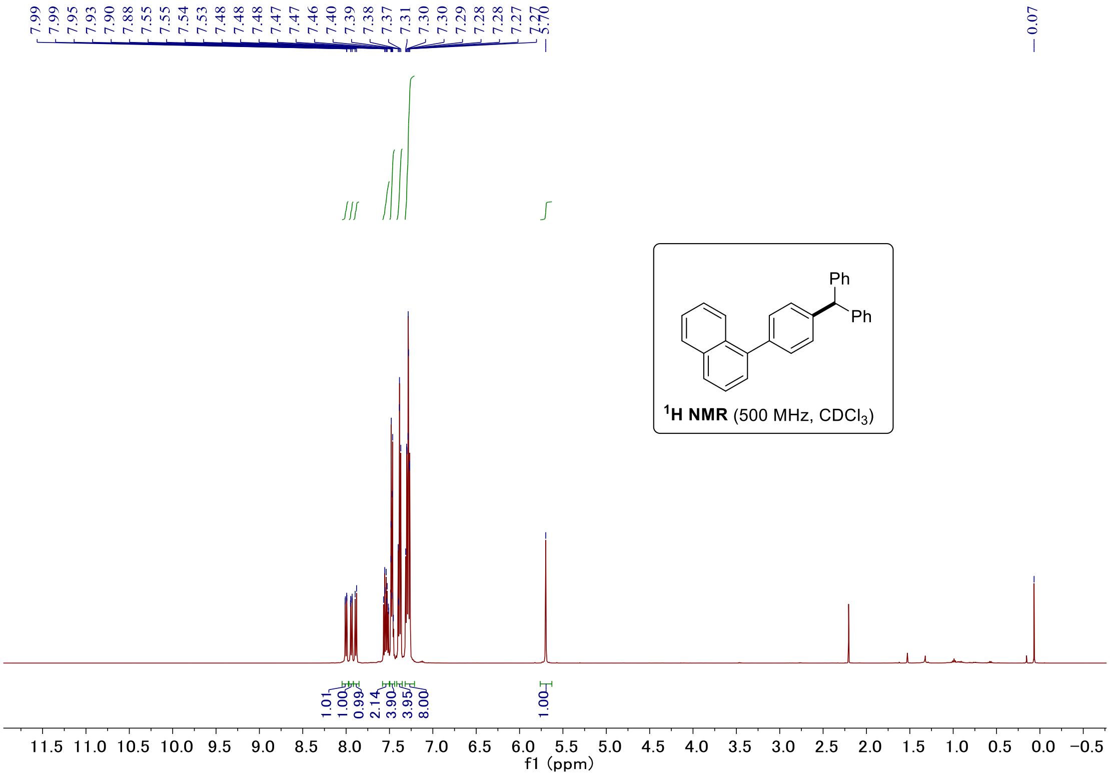

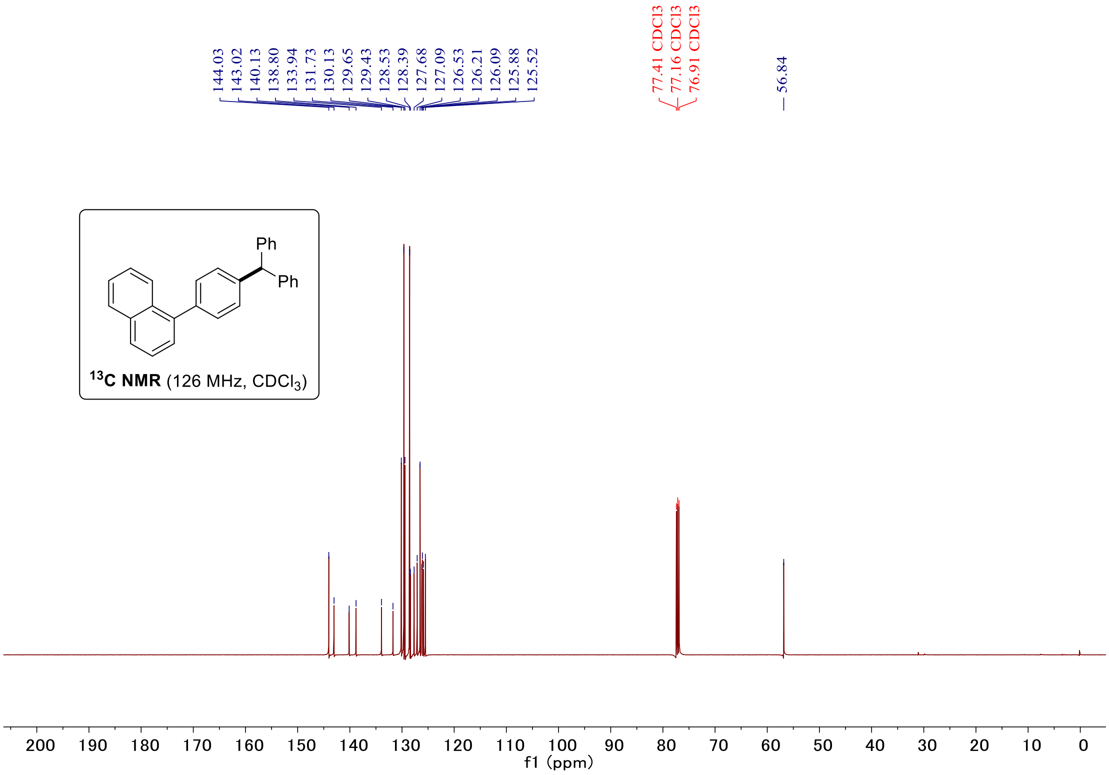

4-Benzhydryl-4'-methylbiphenyl (3ja)

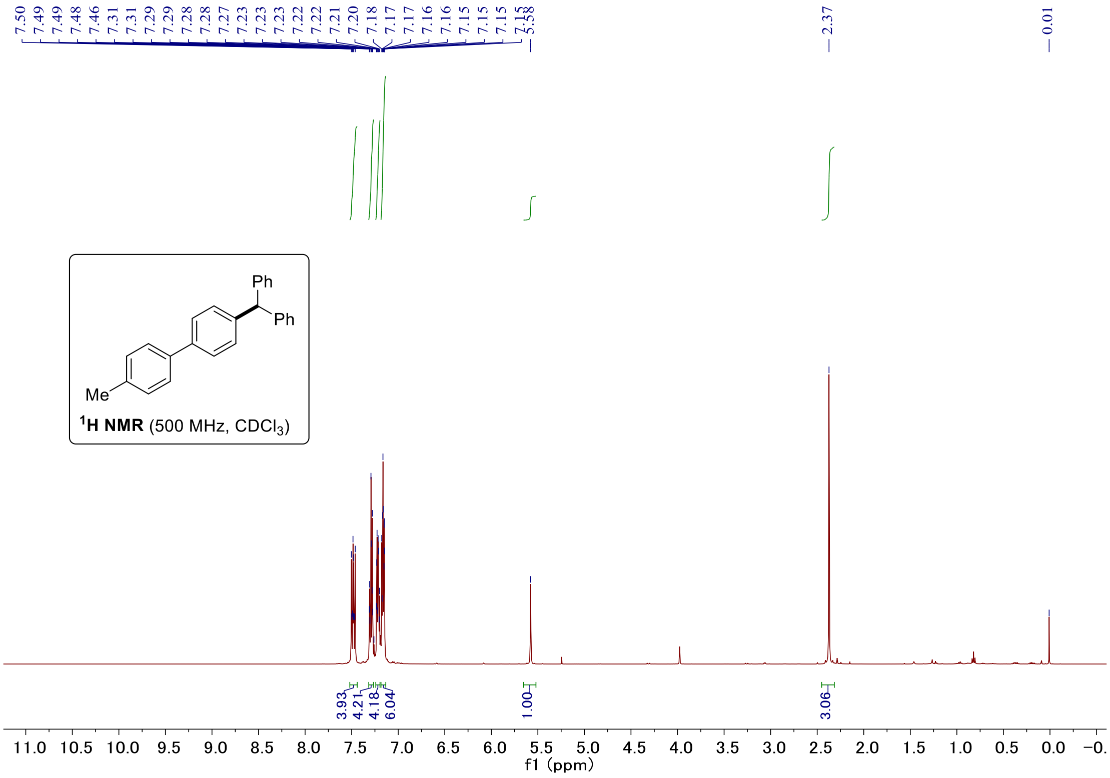

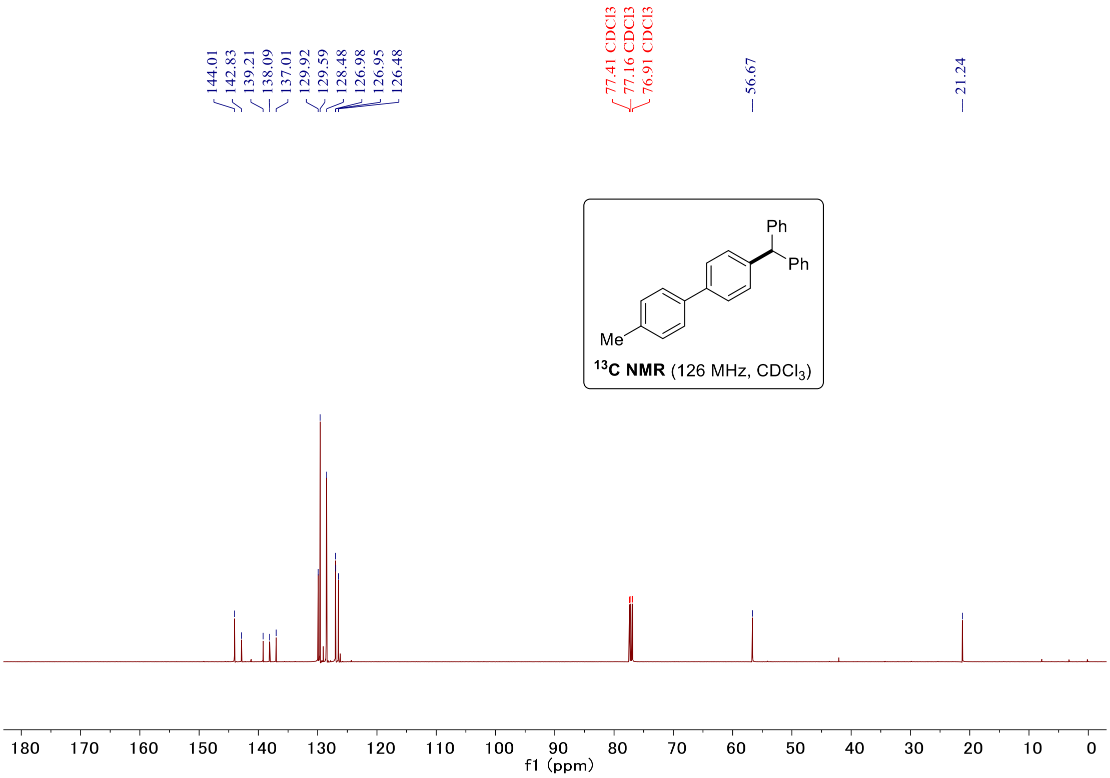

4-Benzhydryl-4'-methoxybiphenyl (3ka)

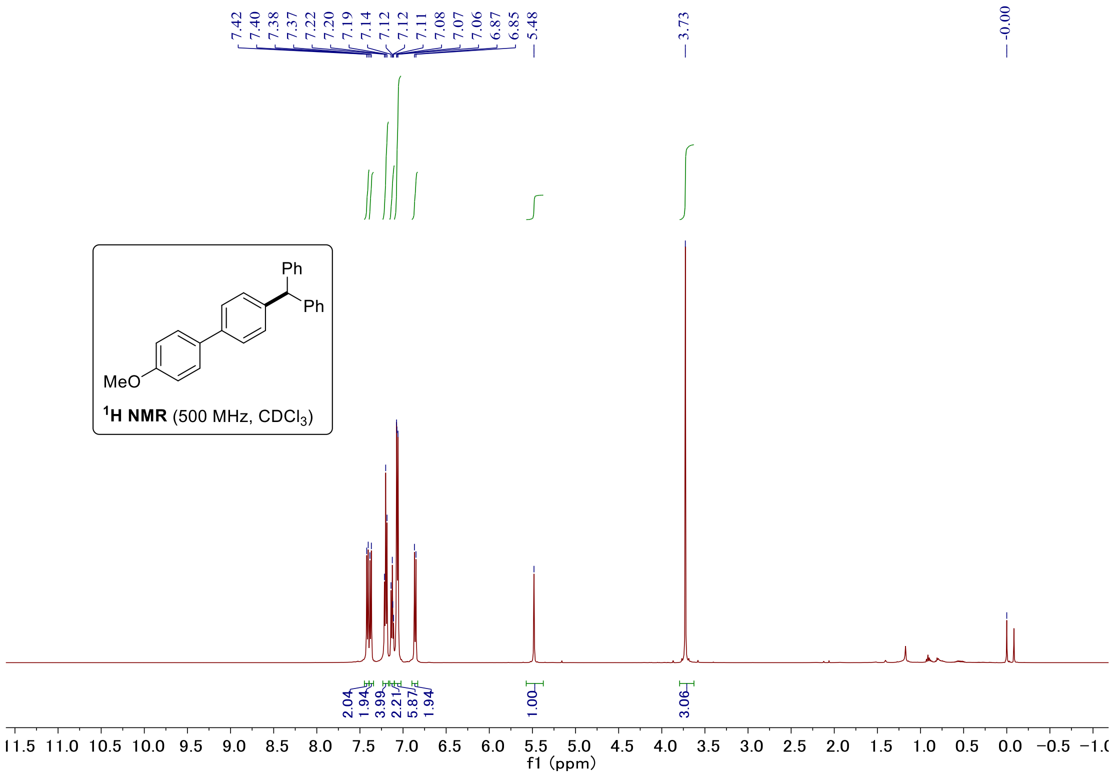

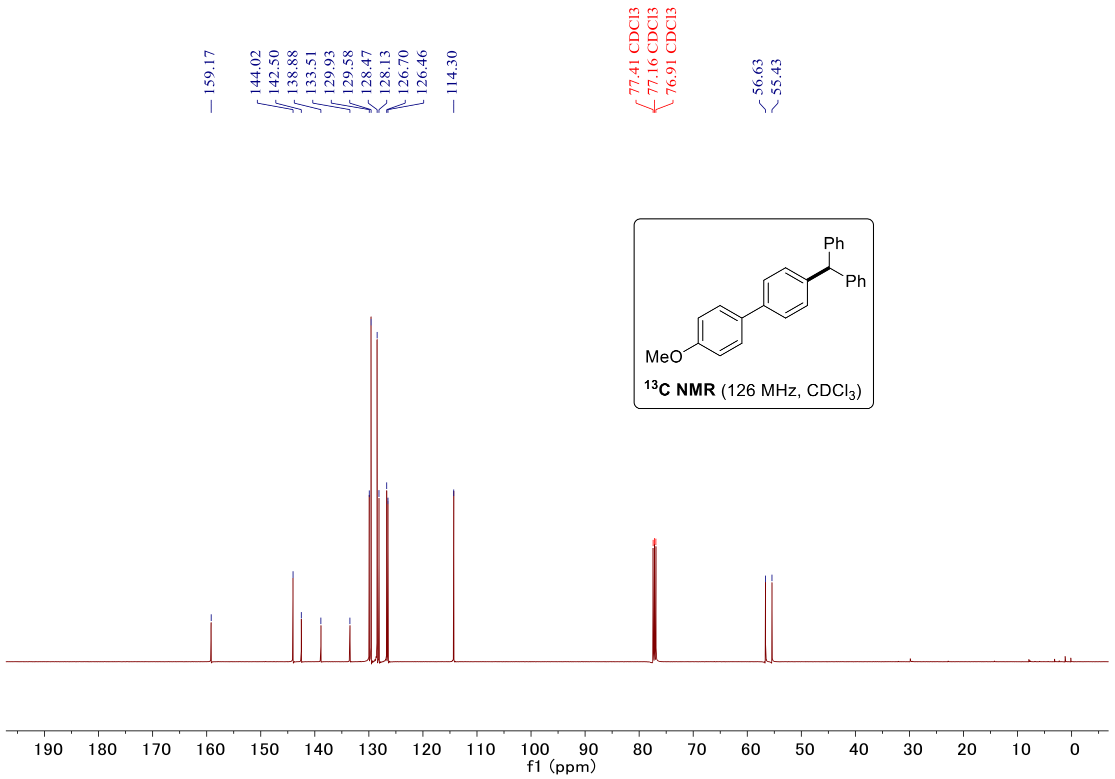

4-Benzhydryl-4'-(benzyloxy)-biphenyl (3la)

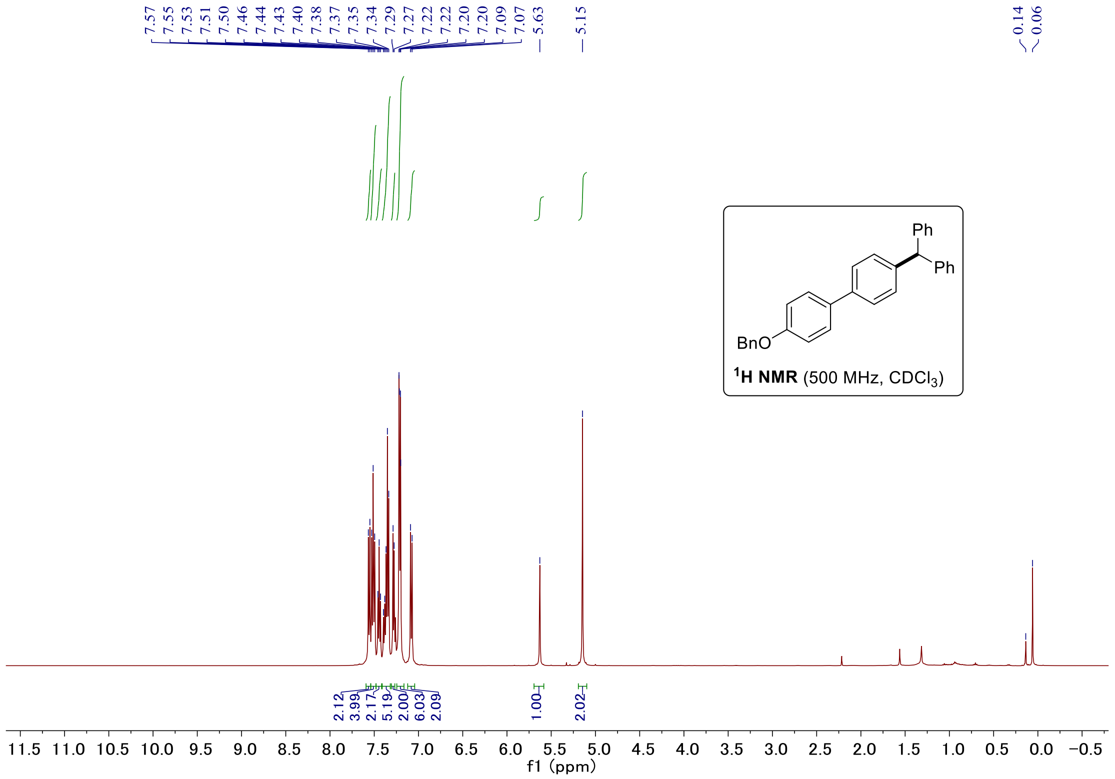

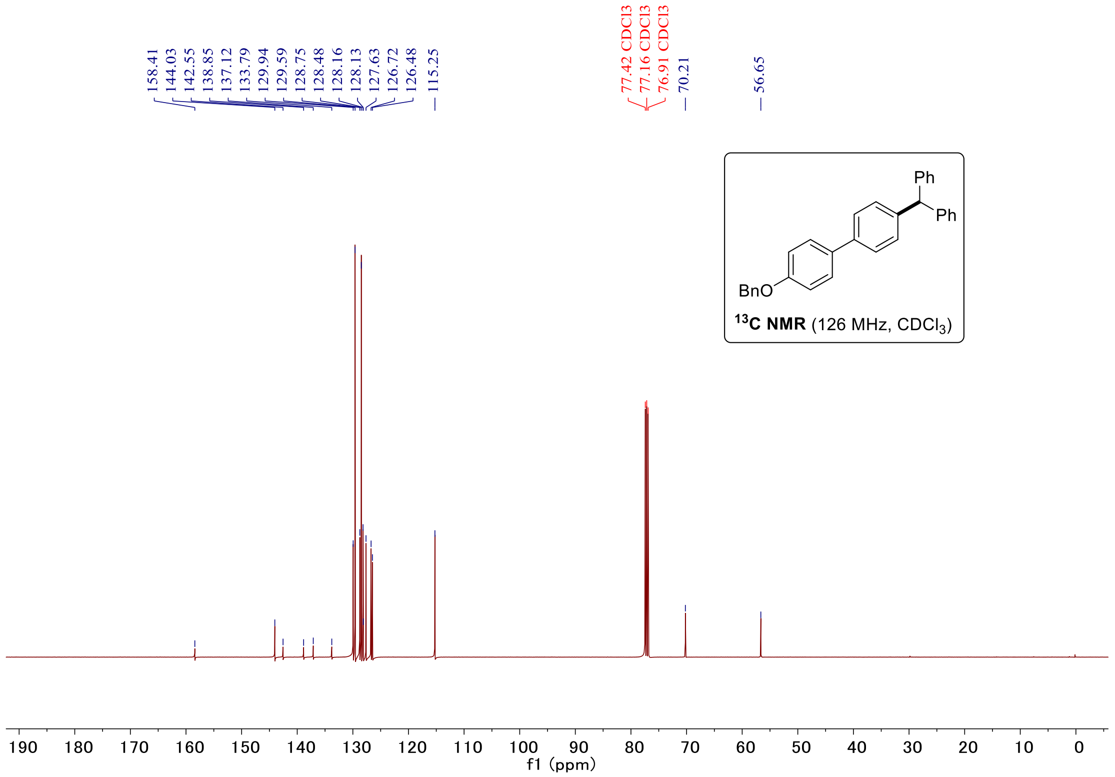

4-Benzhydryl-4'-(trifluoromethyl)biphenyl (3ma)

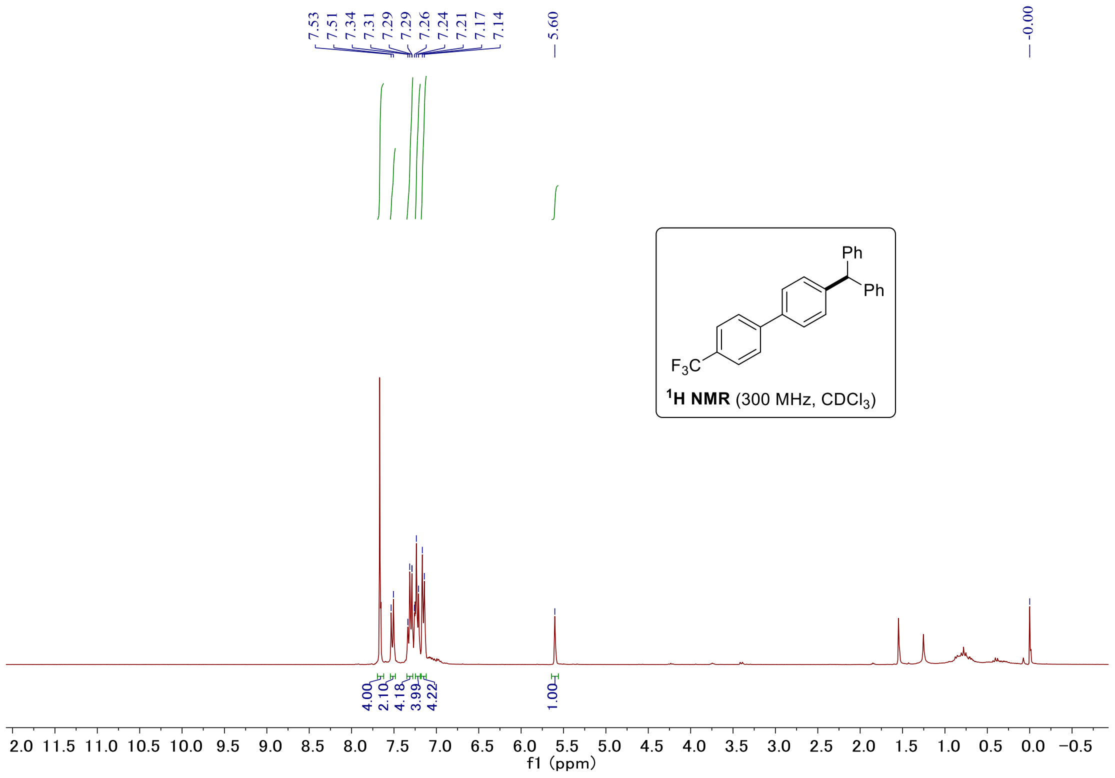

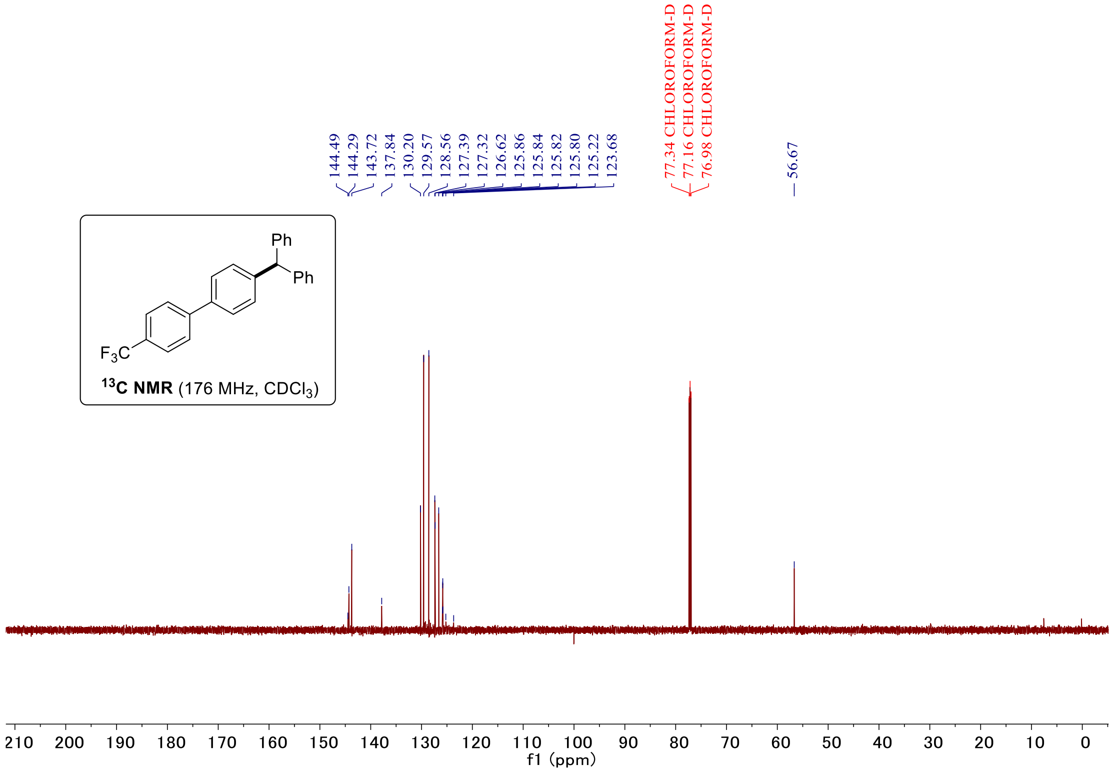

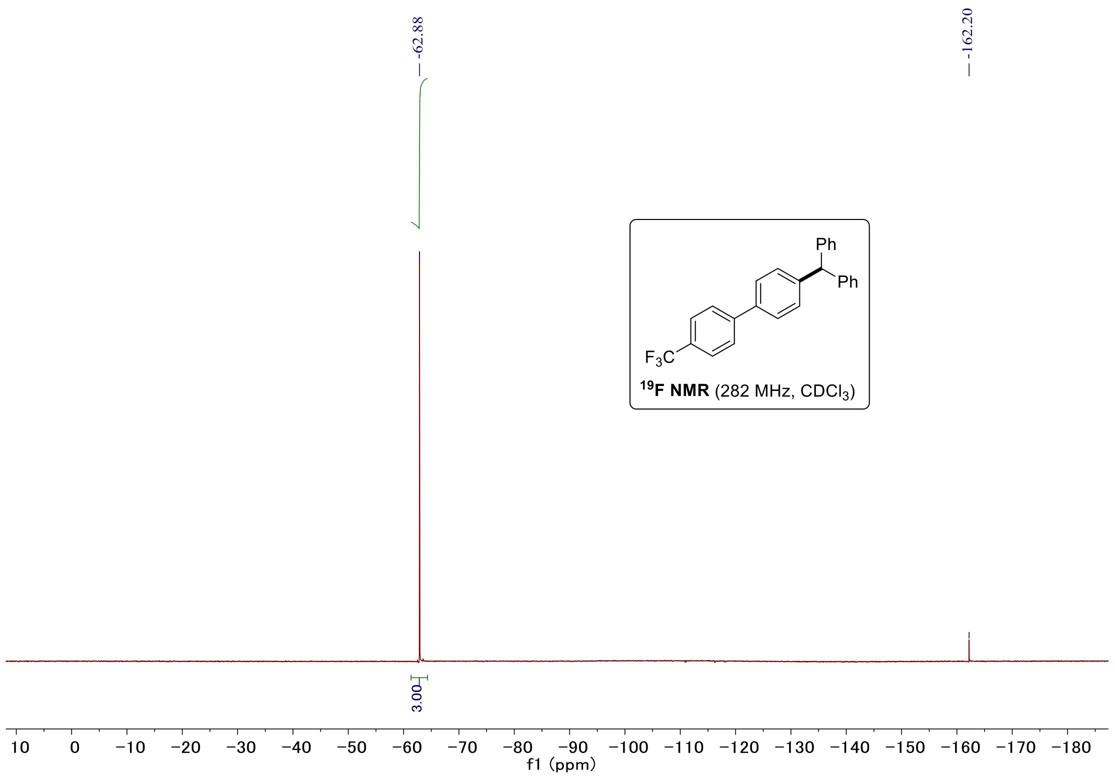

5-(4-Benzhydrylphenyl)benzo[*d*][1,3]dioxole (3na)

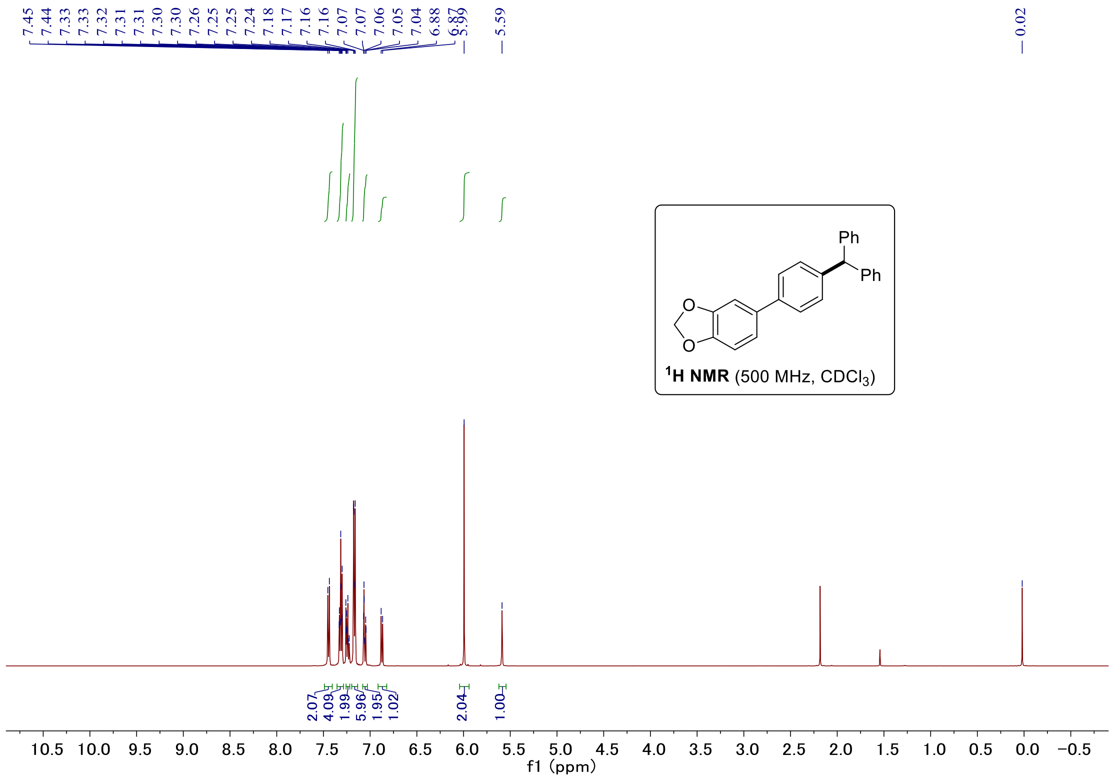

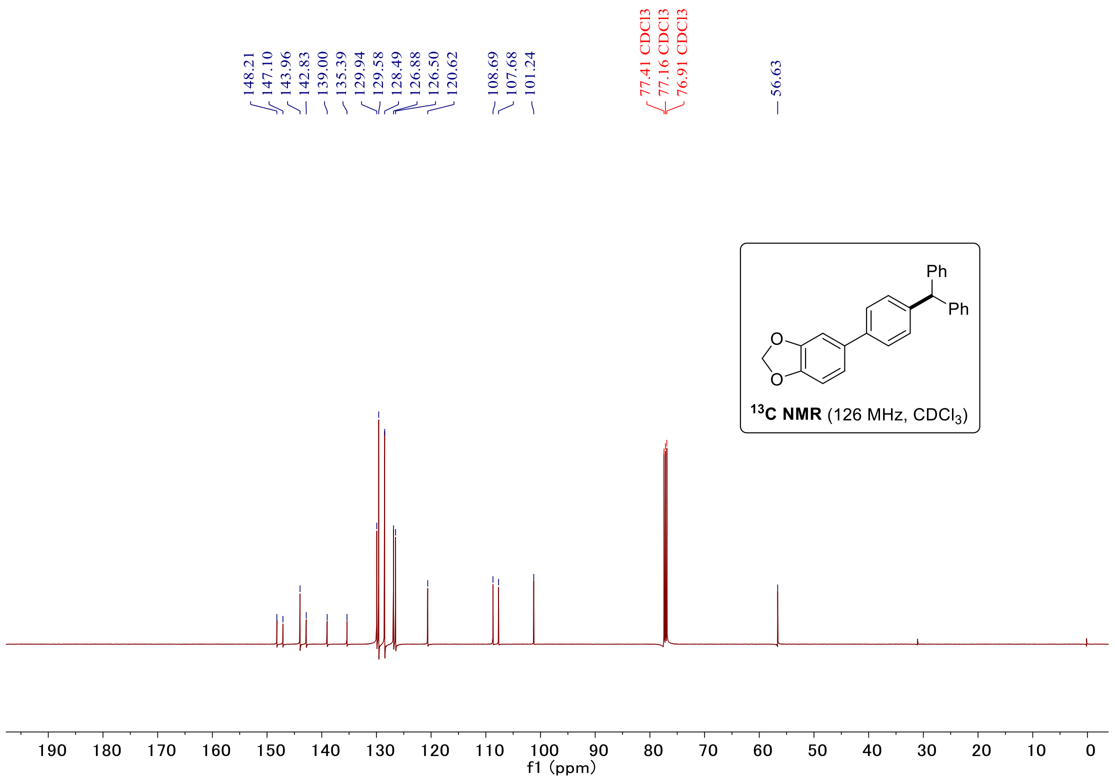

1-(4-Benzhydrylphenyl)-1*H*-pyrrole (3oa)

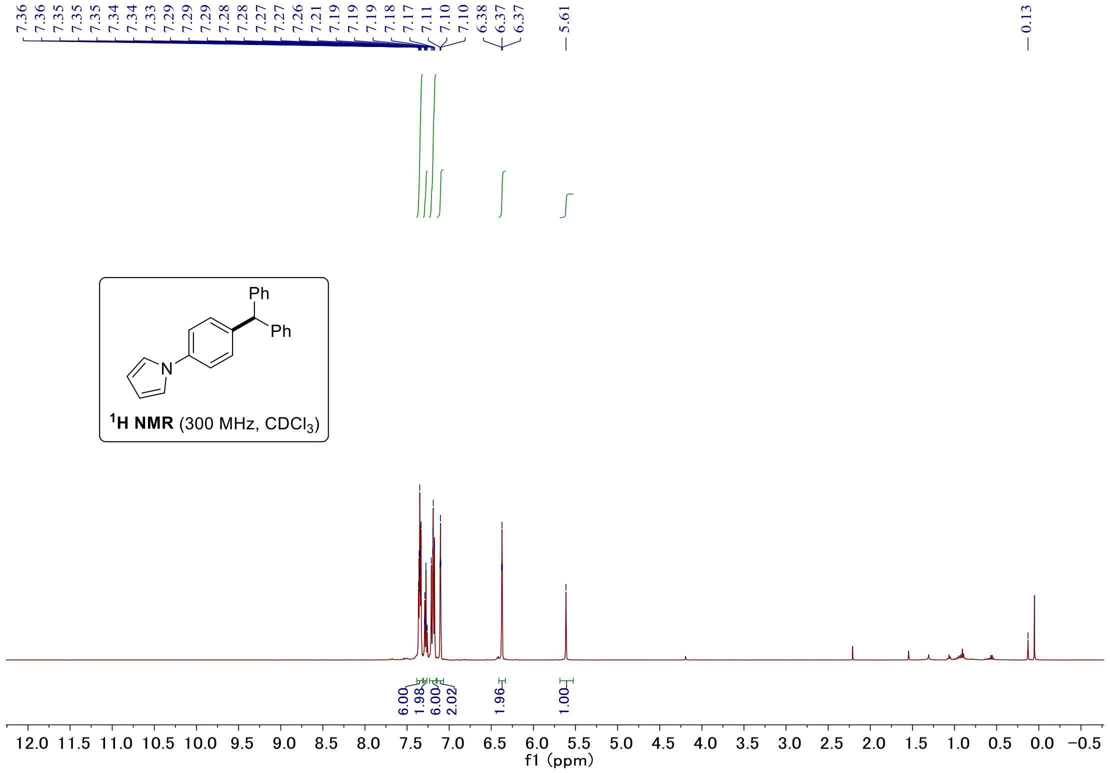

2-(4-Benzhydrylphenyl)-1-methyl-1*H*-indole (3pa)

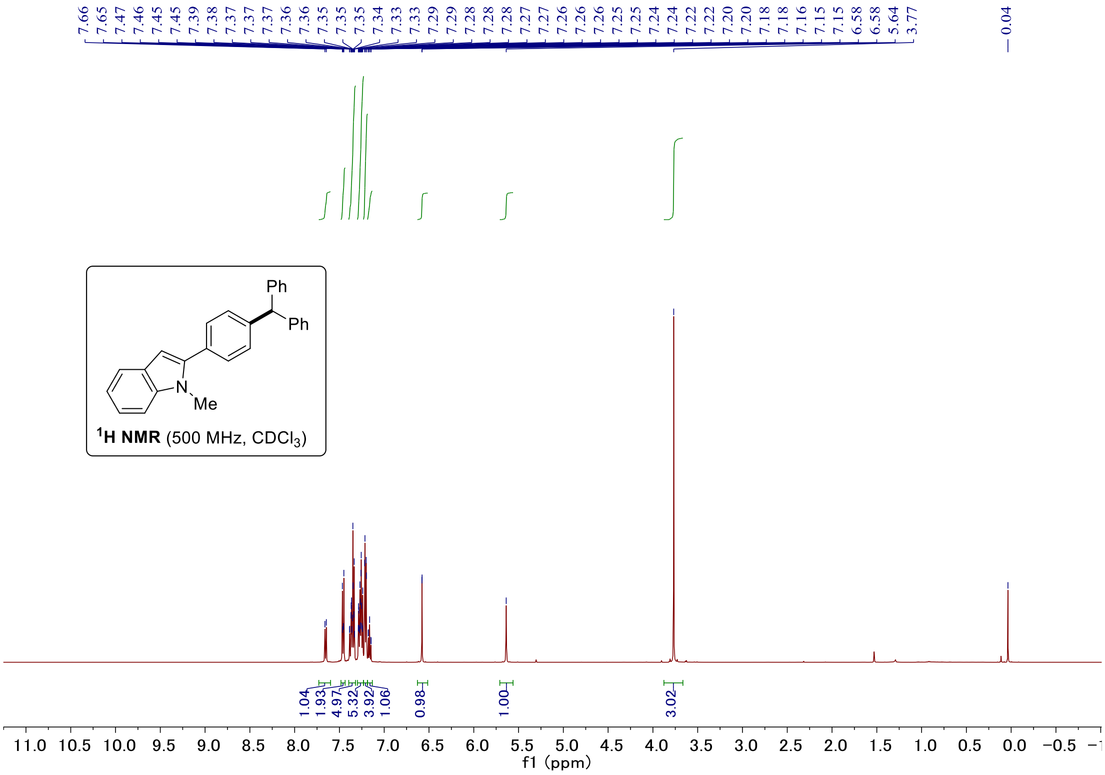

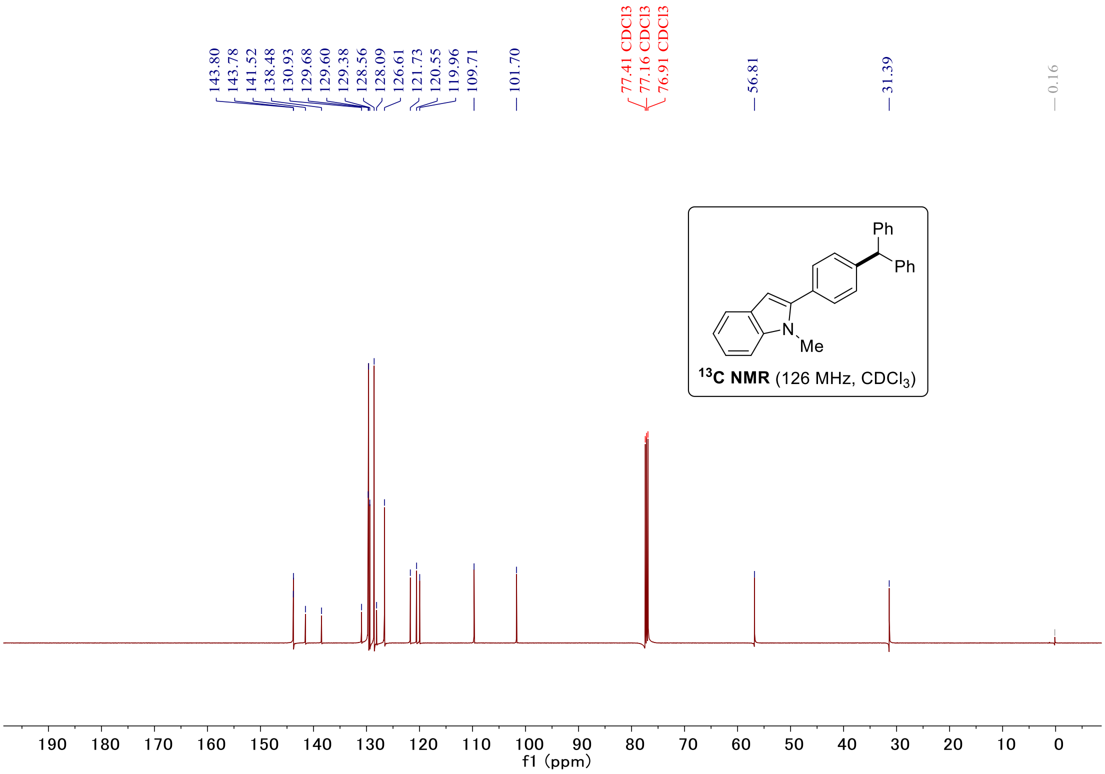

2-(4-Benzhydrylphenyl)benzofuran (3qa)

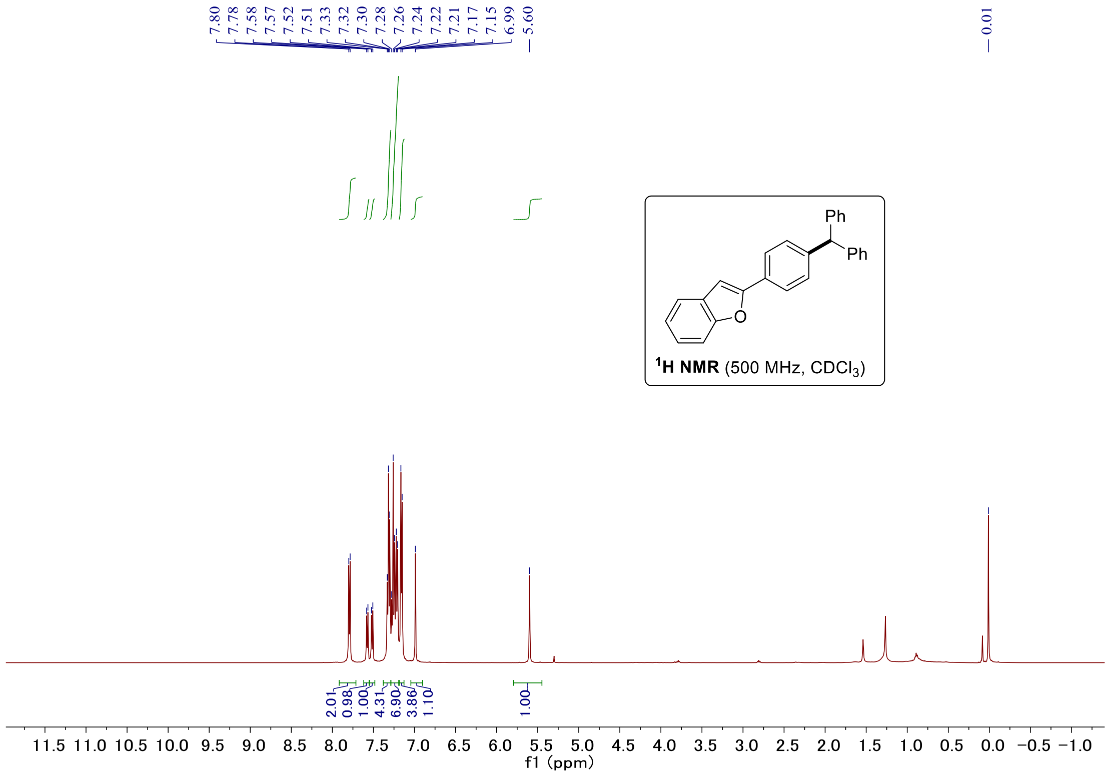

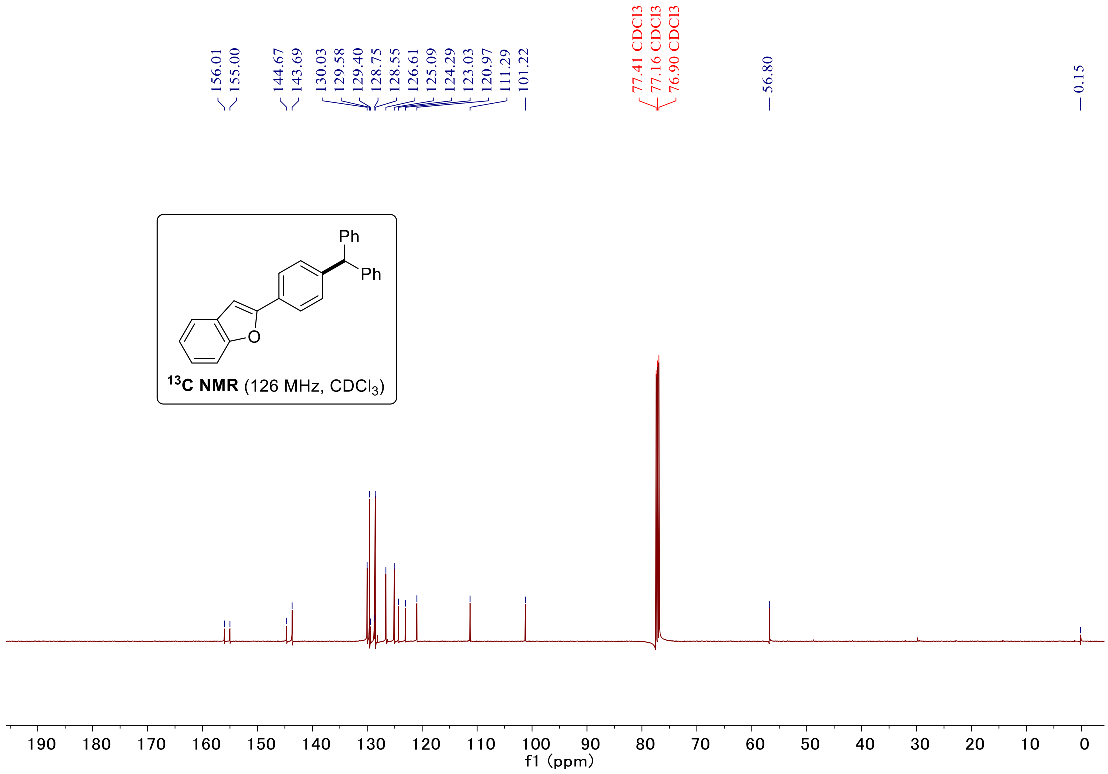

4-((4-Methoxyphenyl)(phenyl)methyl)biphenyl (3ab)

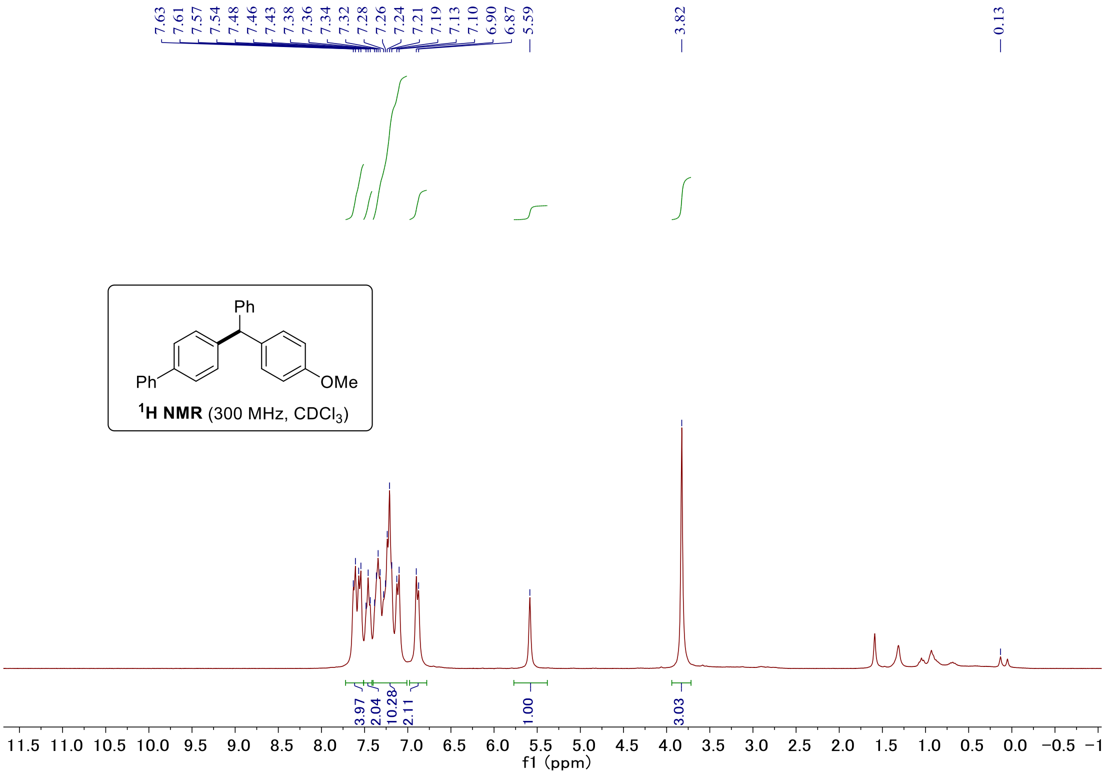

4-(Bis(4-methoxyphenyl)methyl)biphenyl (3ac)

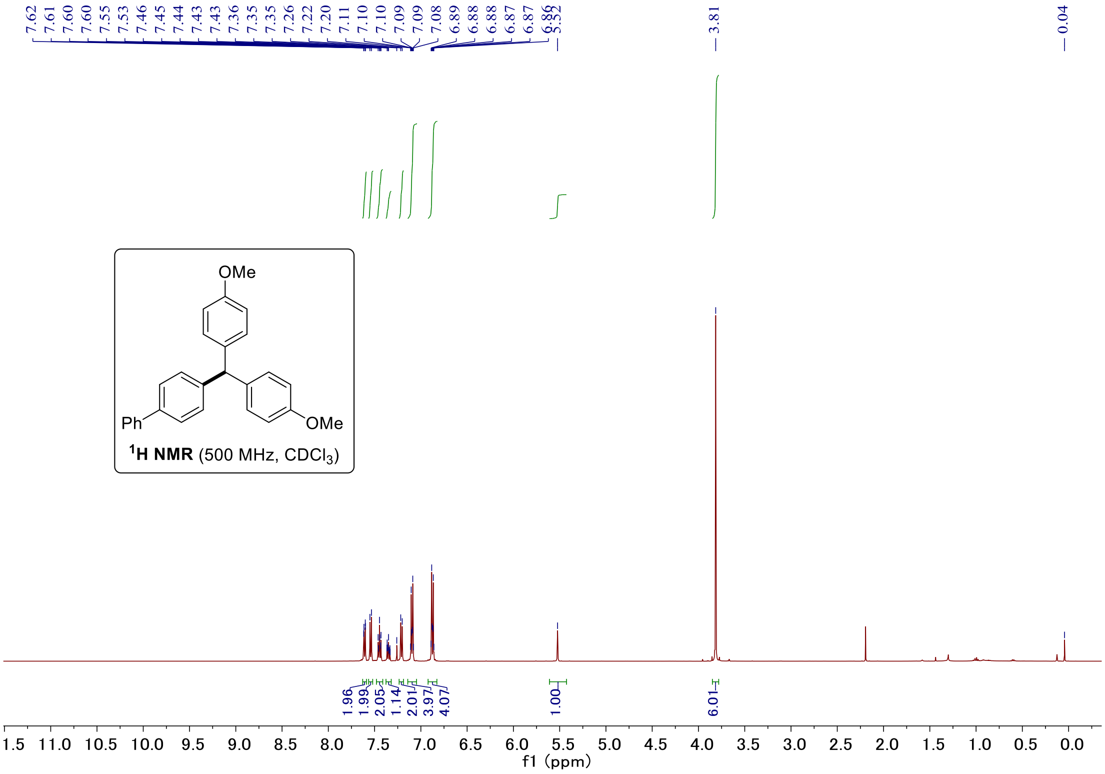

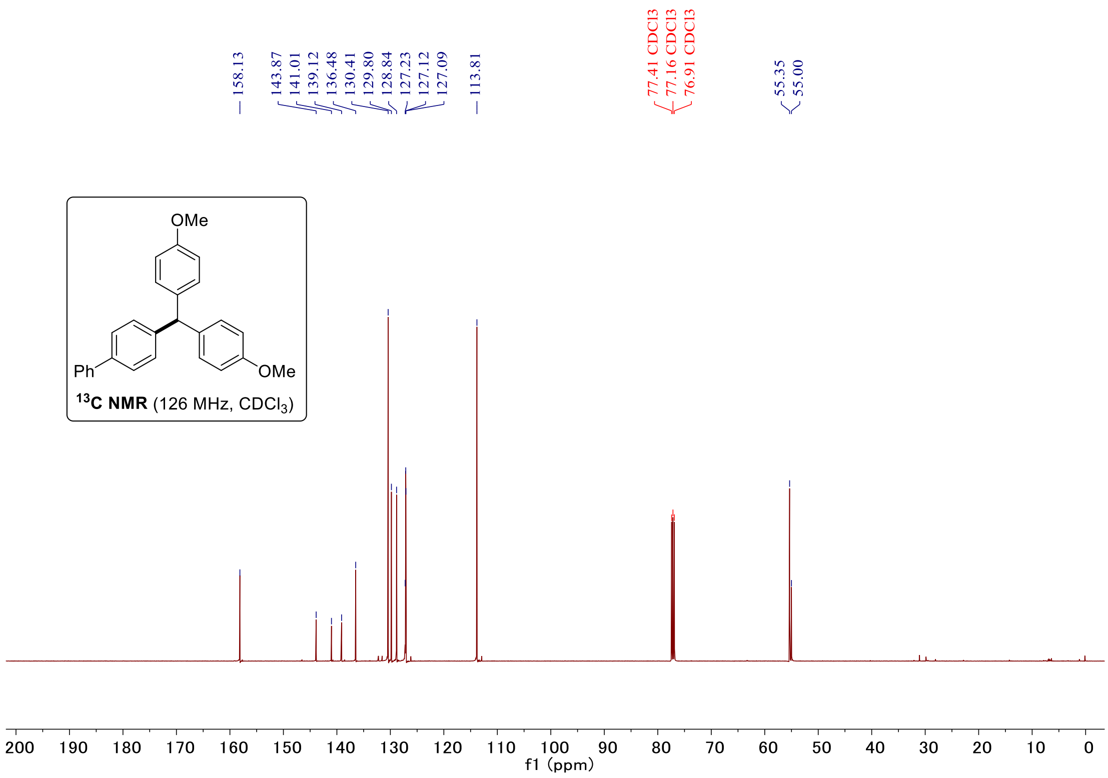

4-(Di-*p*-tolylmethyl)biphenyl (3ad)

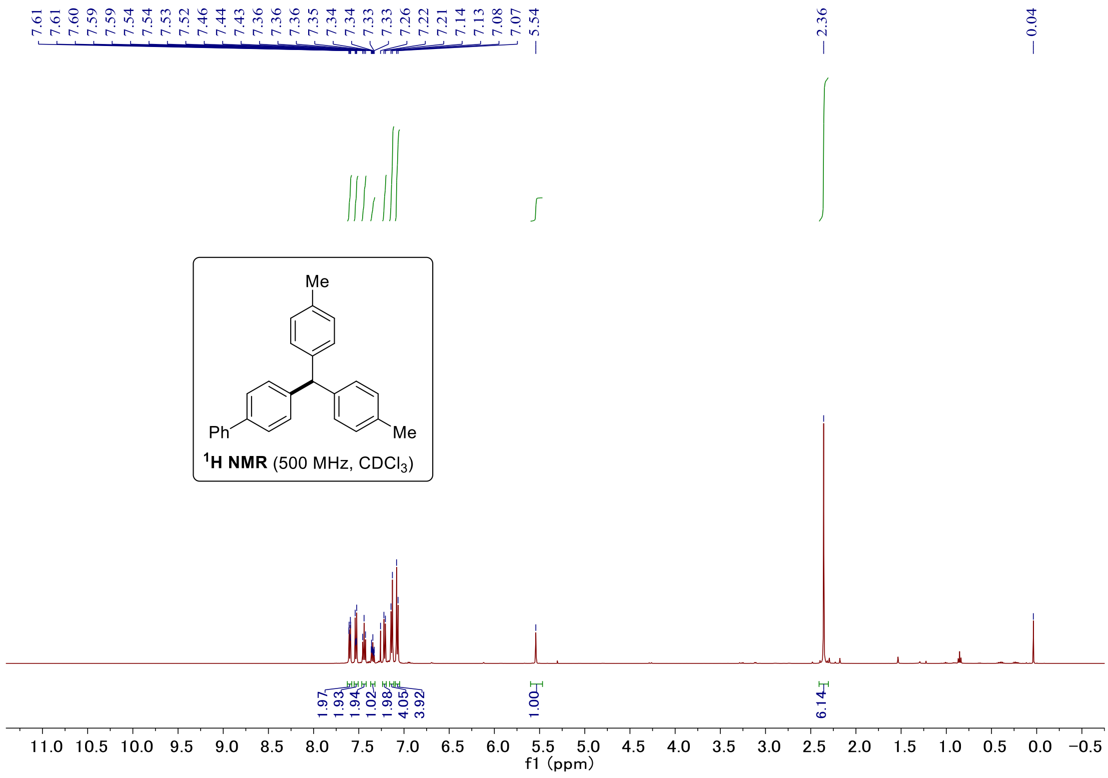

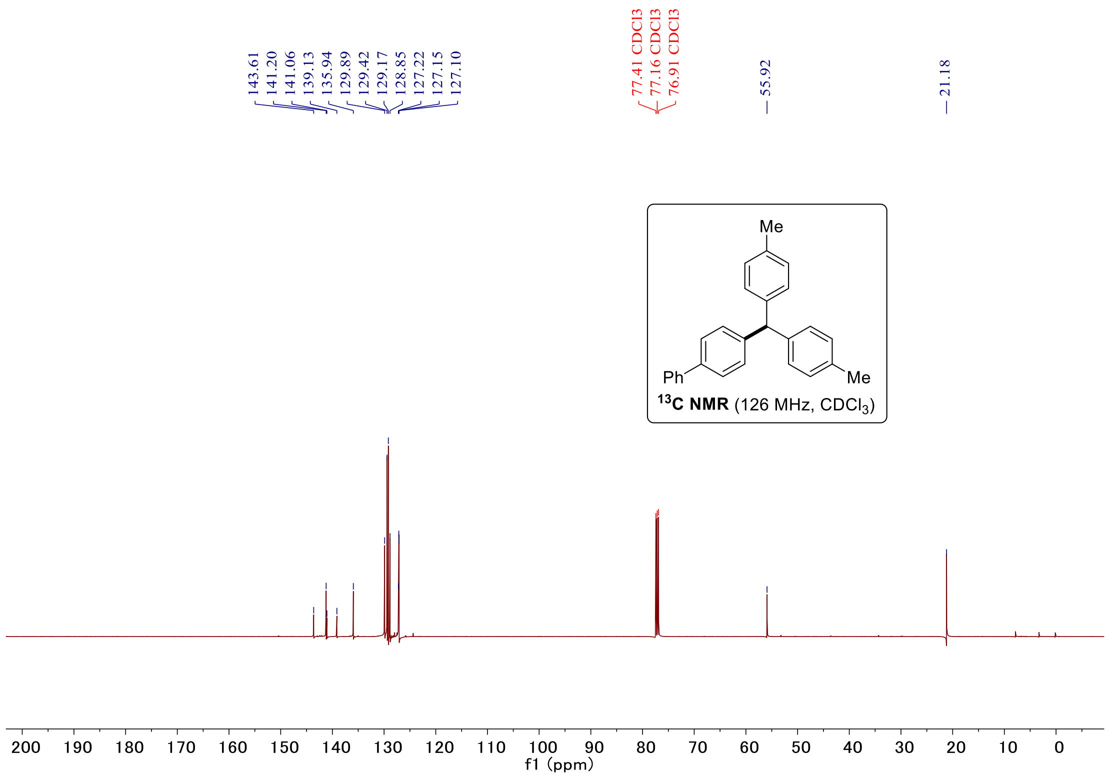

4-(Phenyl(*o*-tolyl)methyl)biphenyl (3ae)

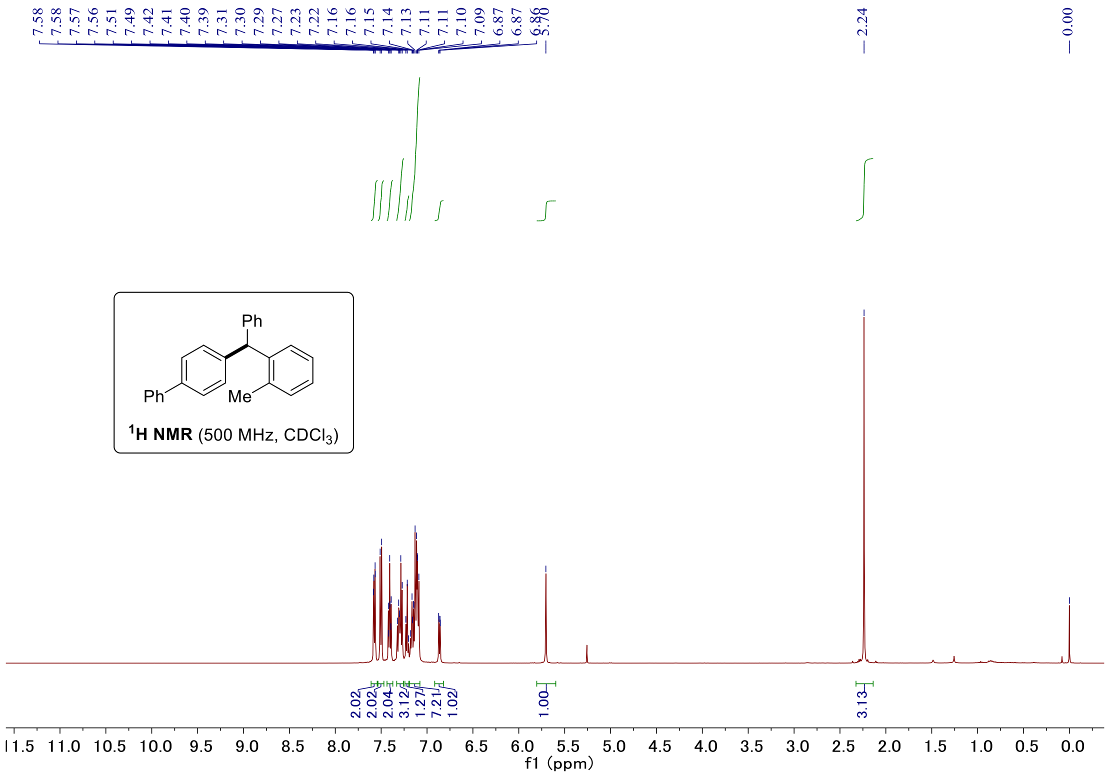

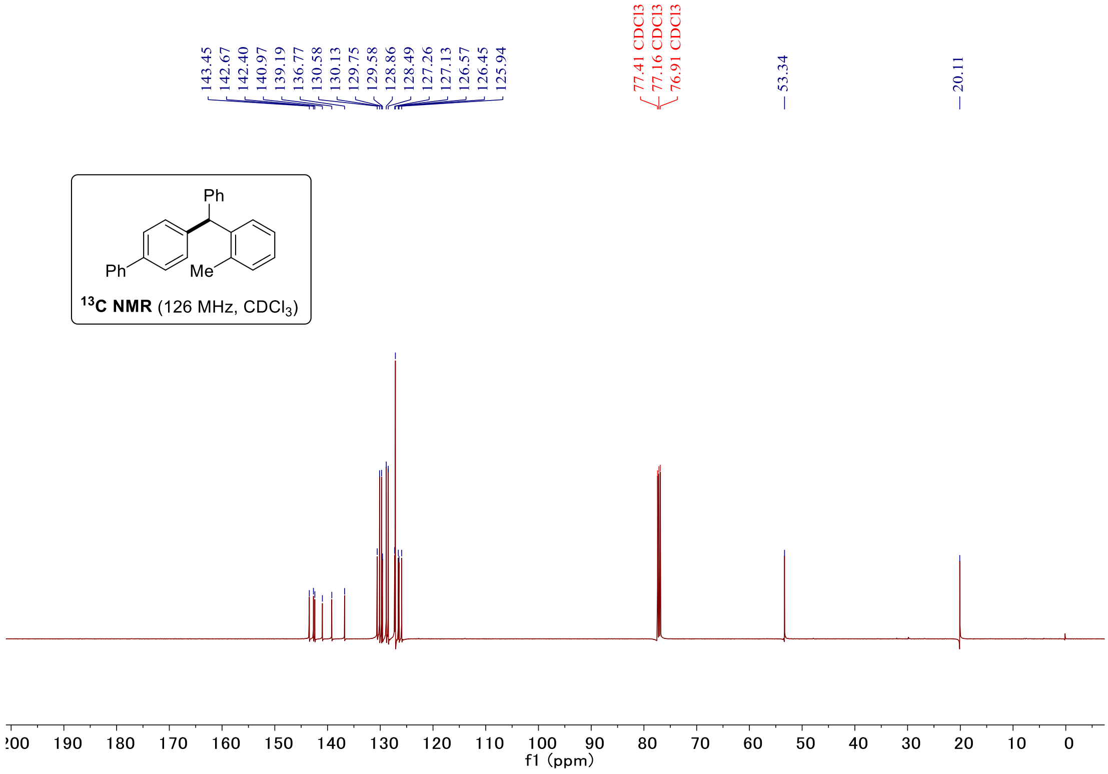

4-((4-Chlorophenyl)(phenyl)methyl)biphenyl (3af)

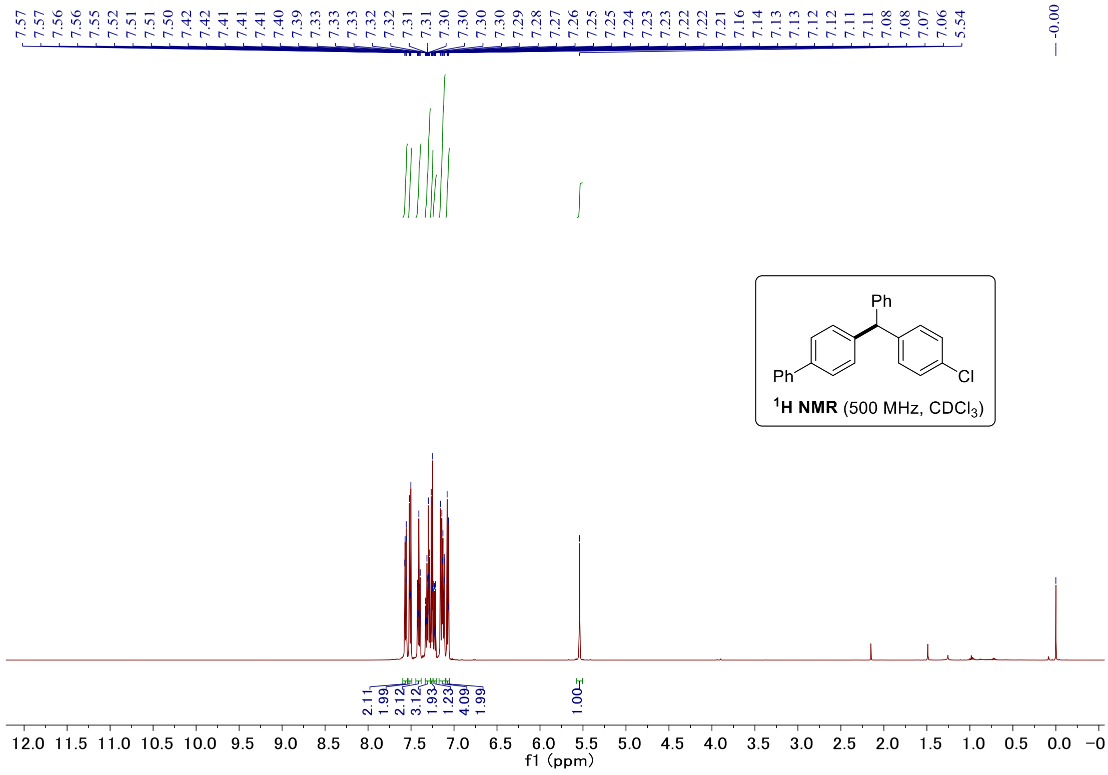

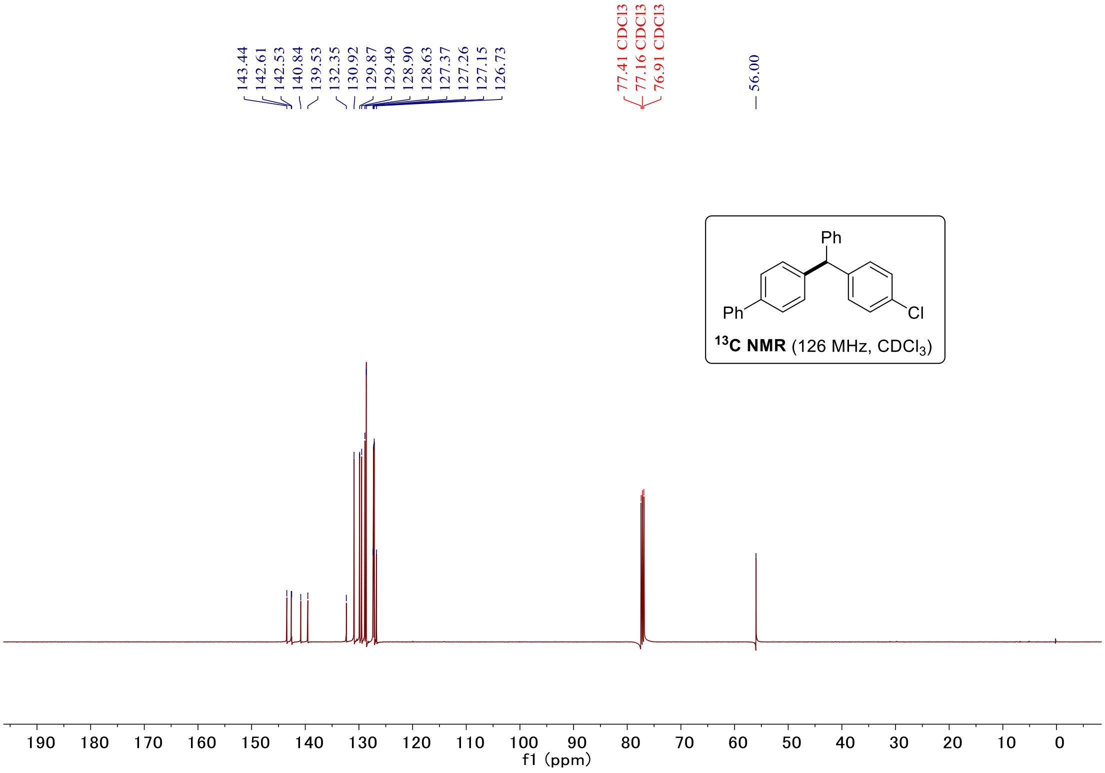

2-(Biphenyl-4-yl(phenyl)methyl)thiophene (3ag)

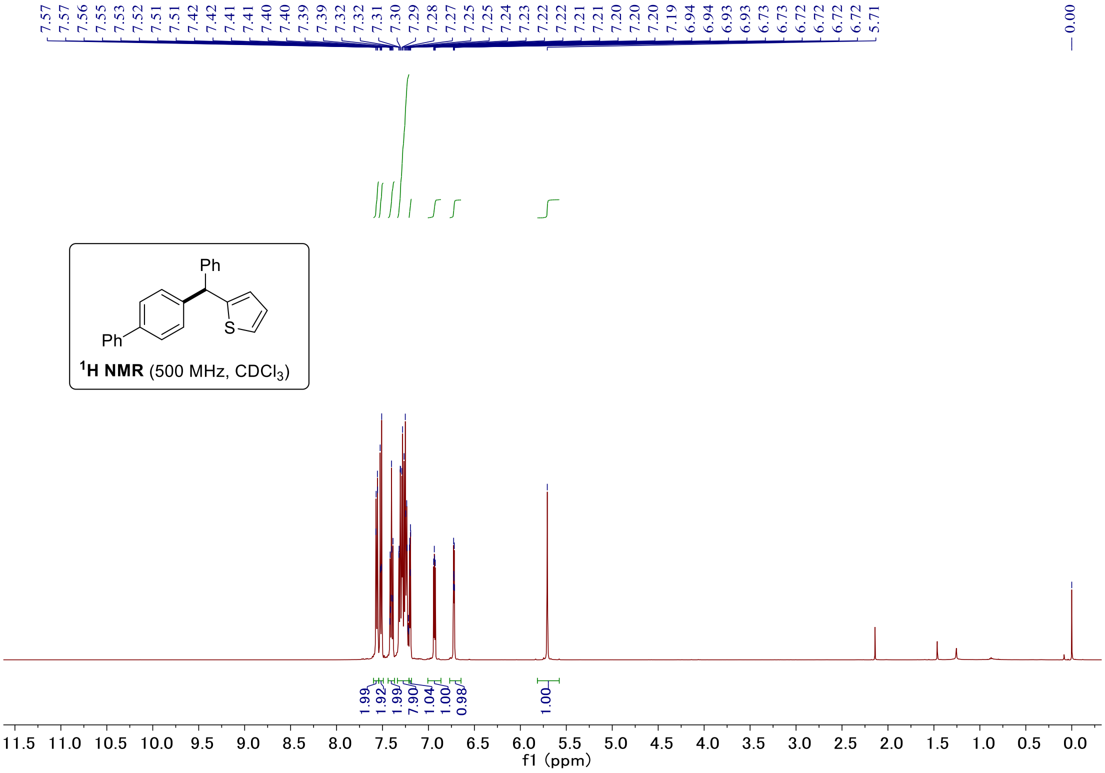

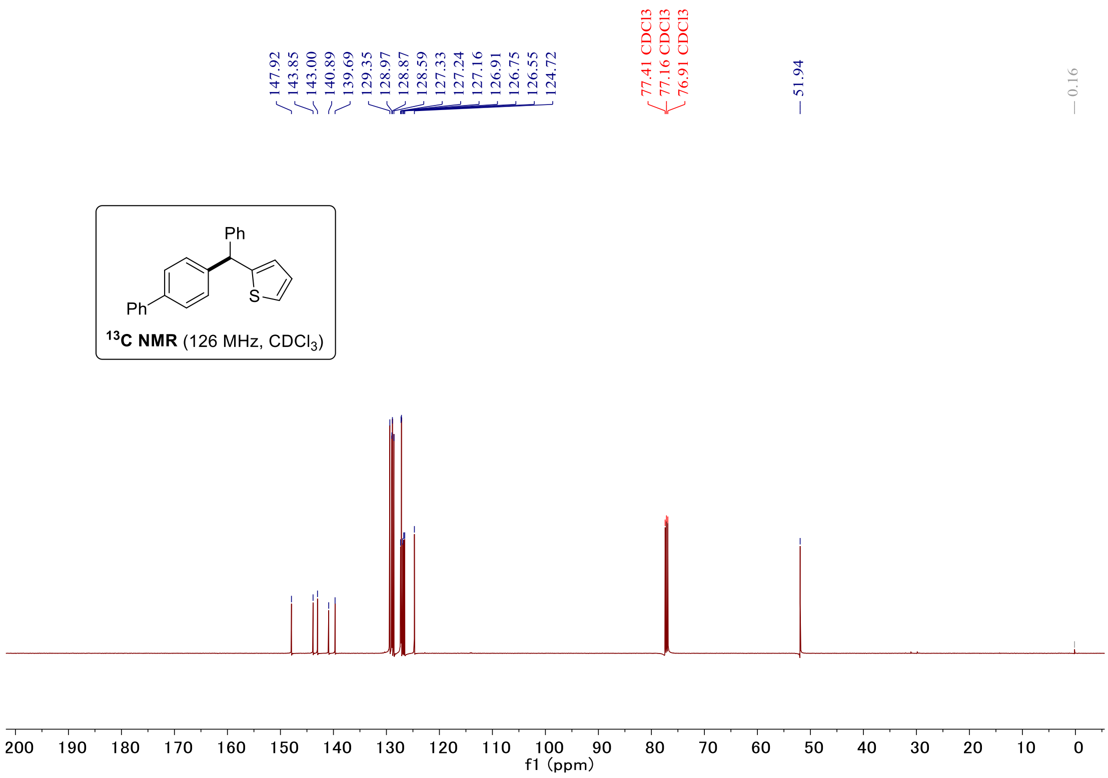

4-((4-Methoxyphenyl)(phenyl)methyl)-4'-methylbiphenyl (3jb)

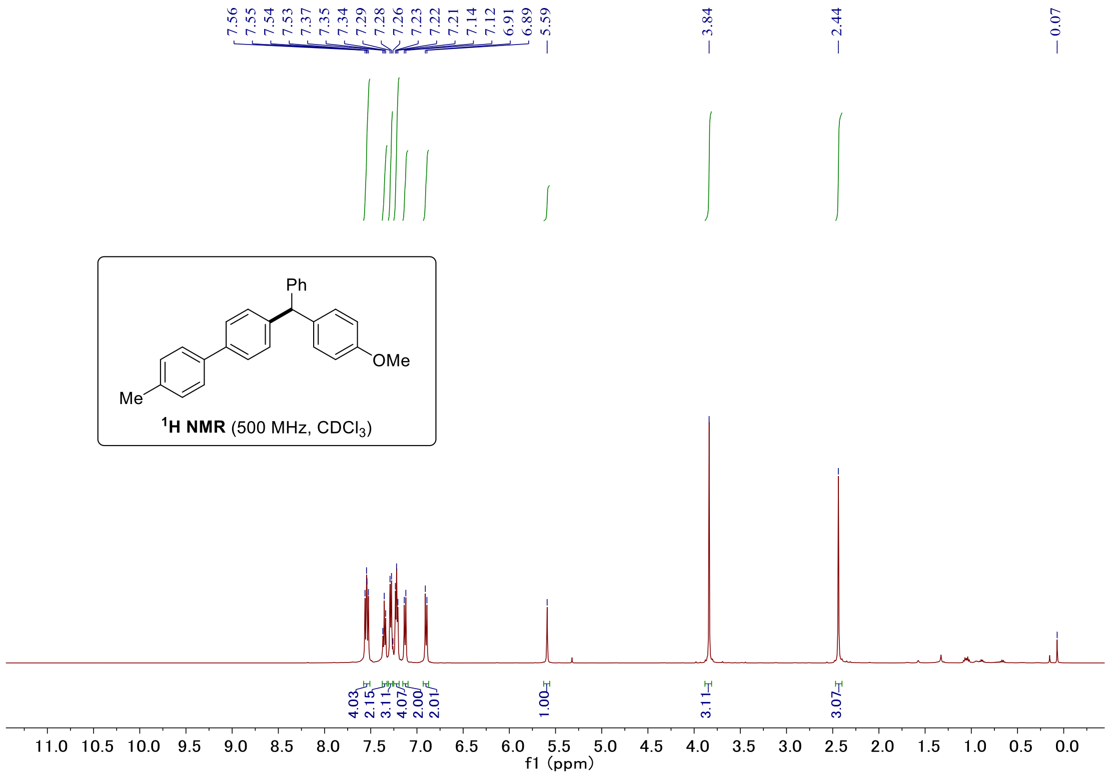

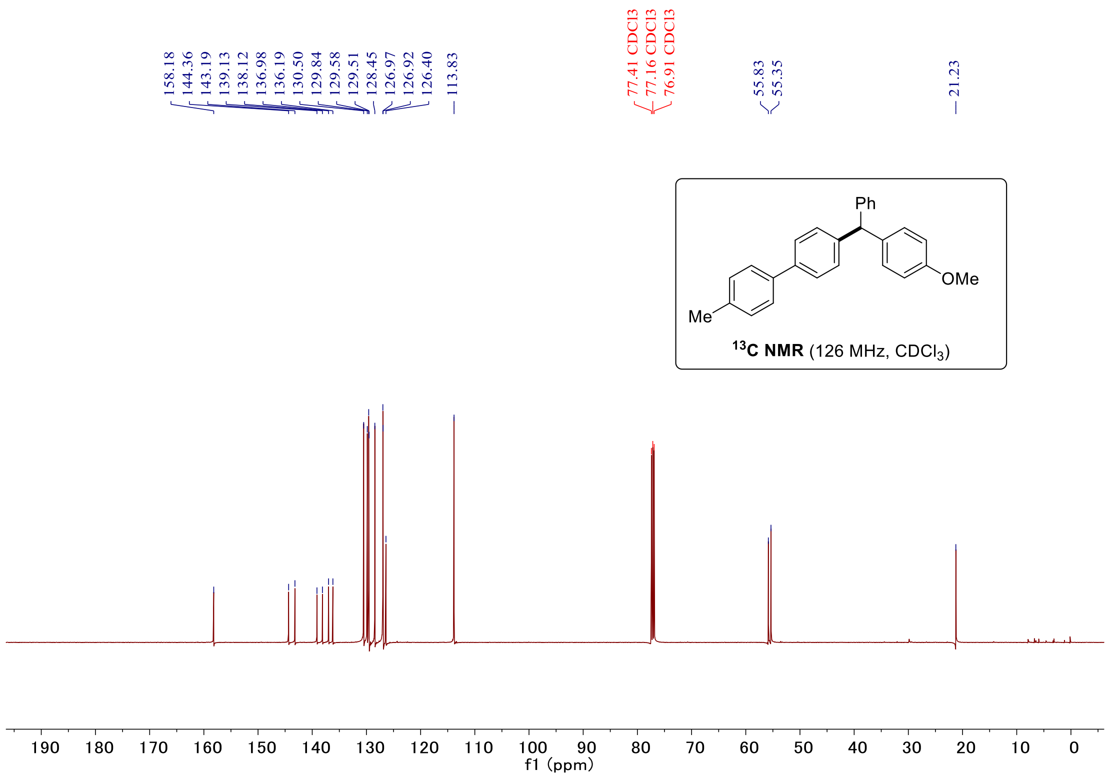

4-(Bis(4-methoxyphenyl)methyl)-4'-methylbiphenyl (3jc)

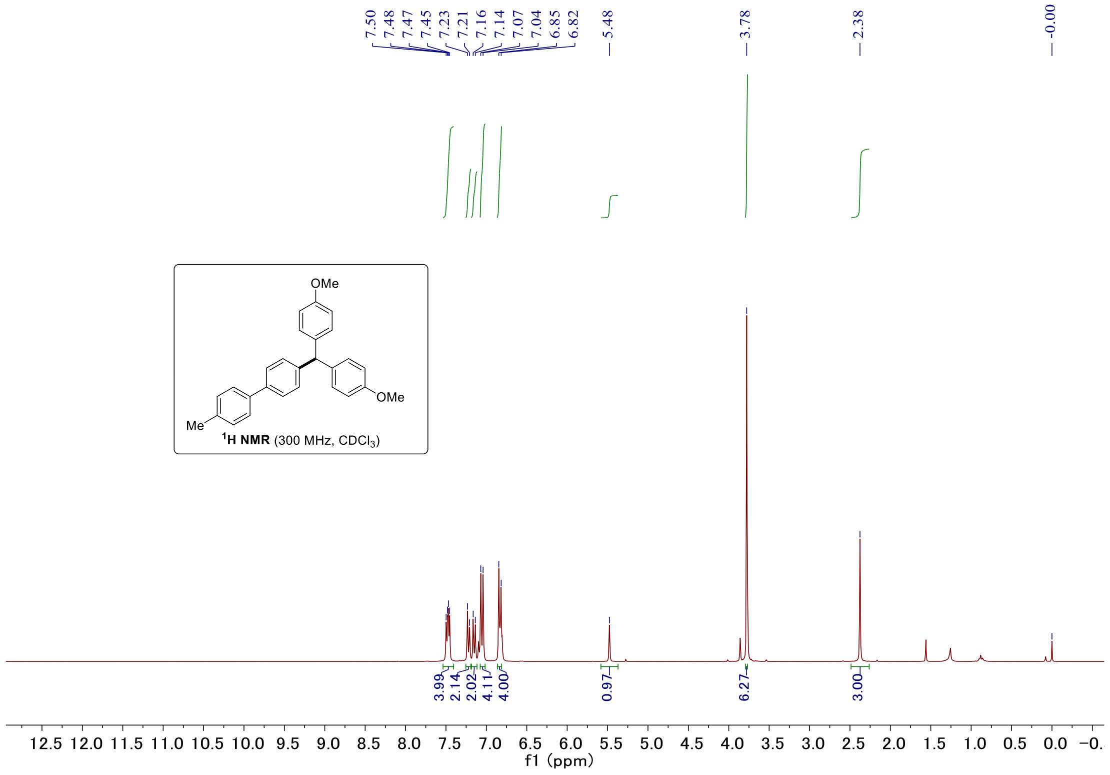

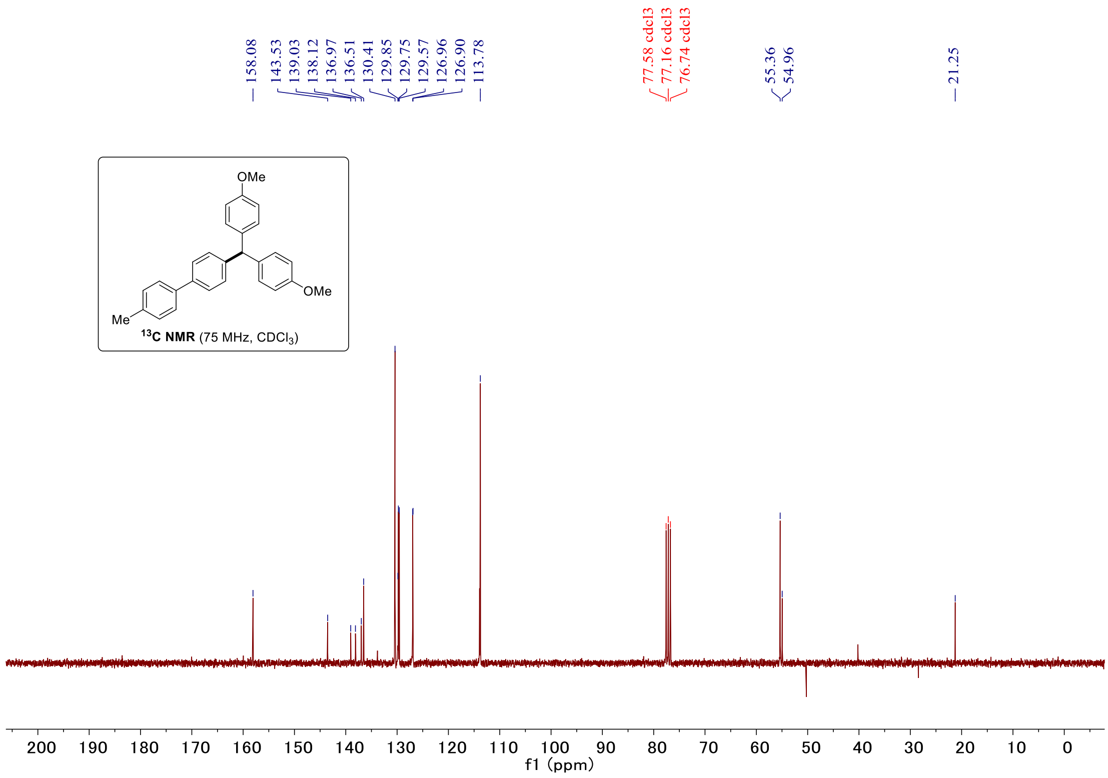

2-((4'-Methyl-biphenyl]-4-yl)(phenyl)methyl)thiophene (3jg)

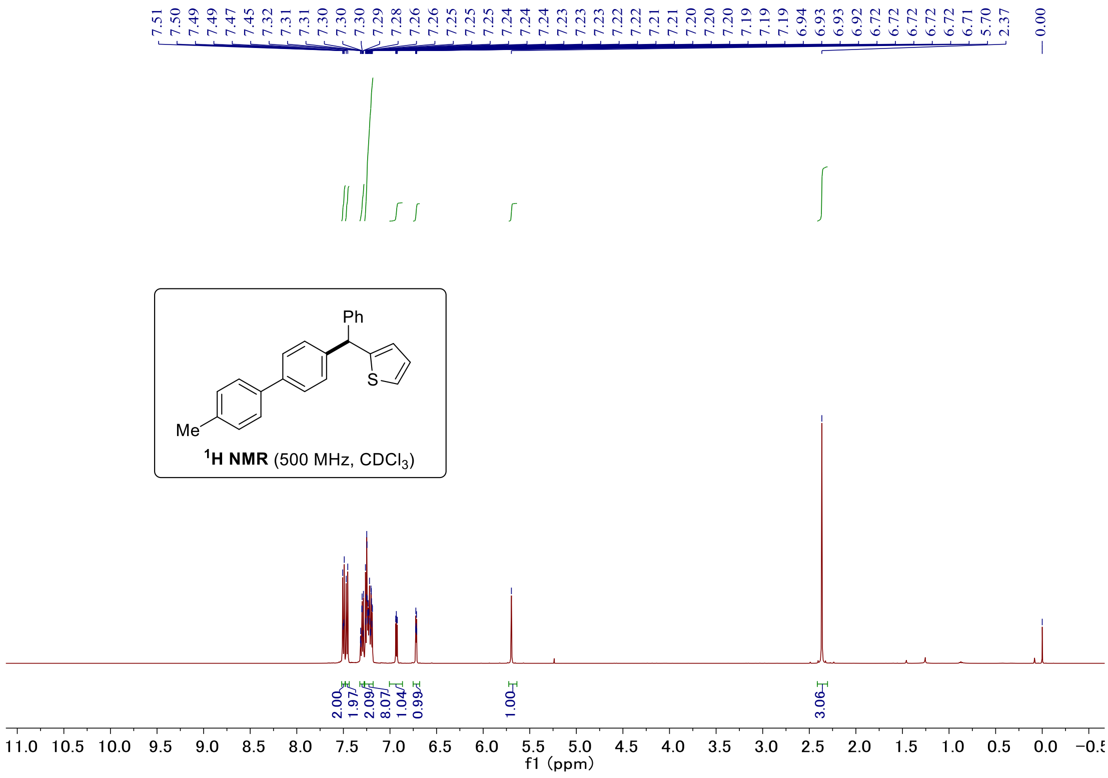

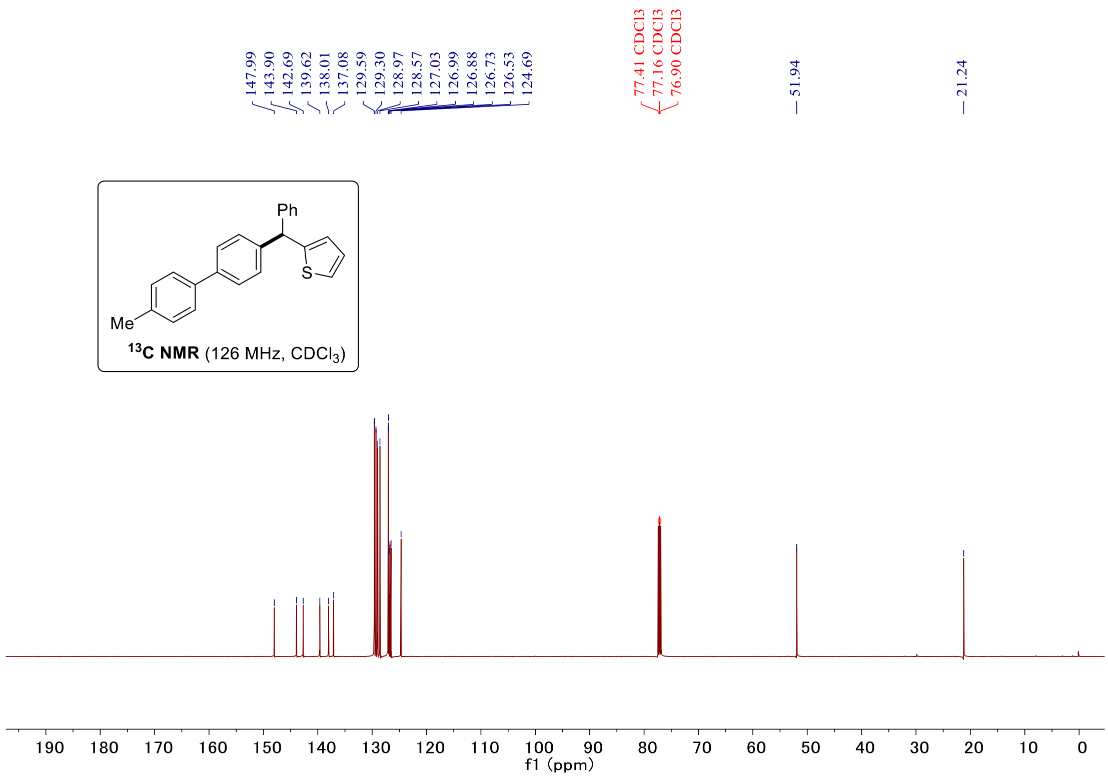

4-Methoxy-4'-((4-methoxyphenyl)(phenyl)methyl)biphenyl (3kb)

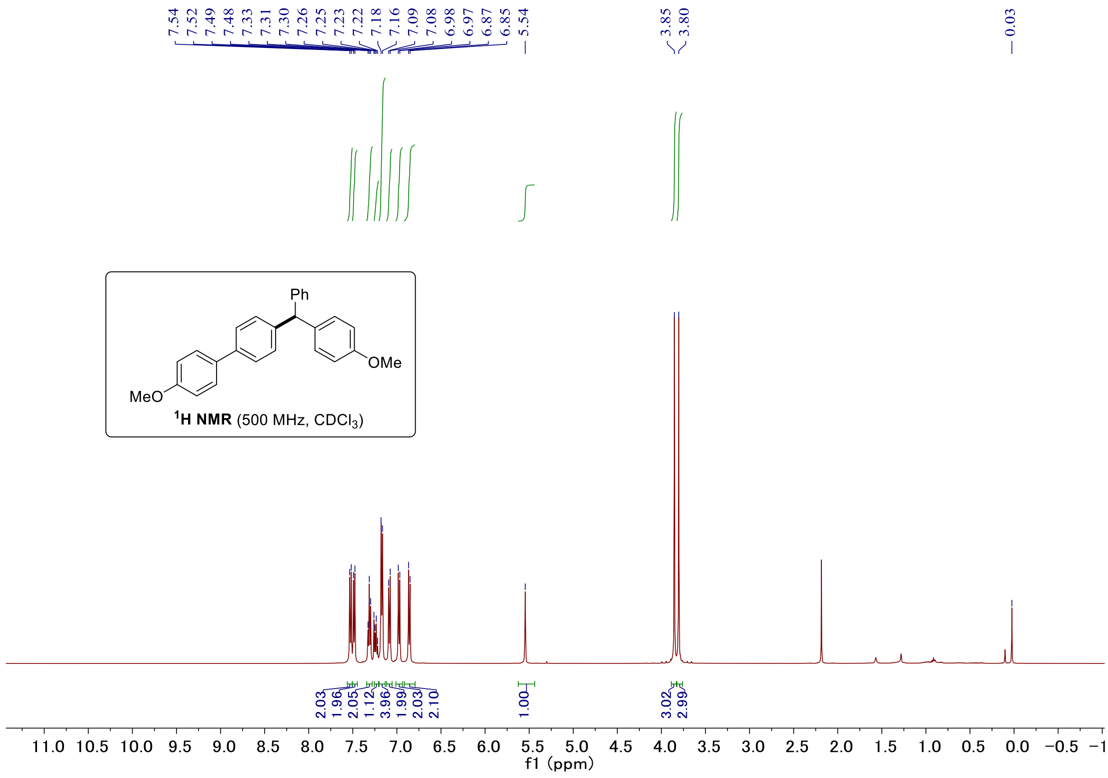

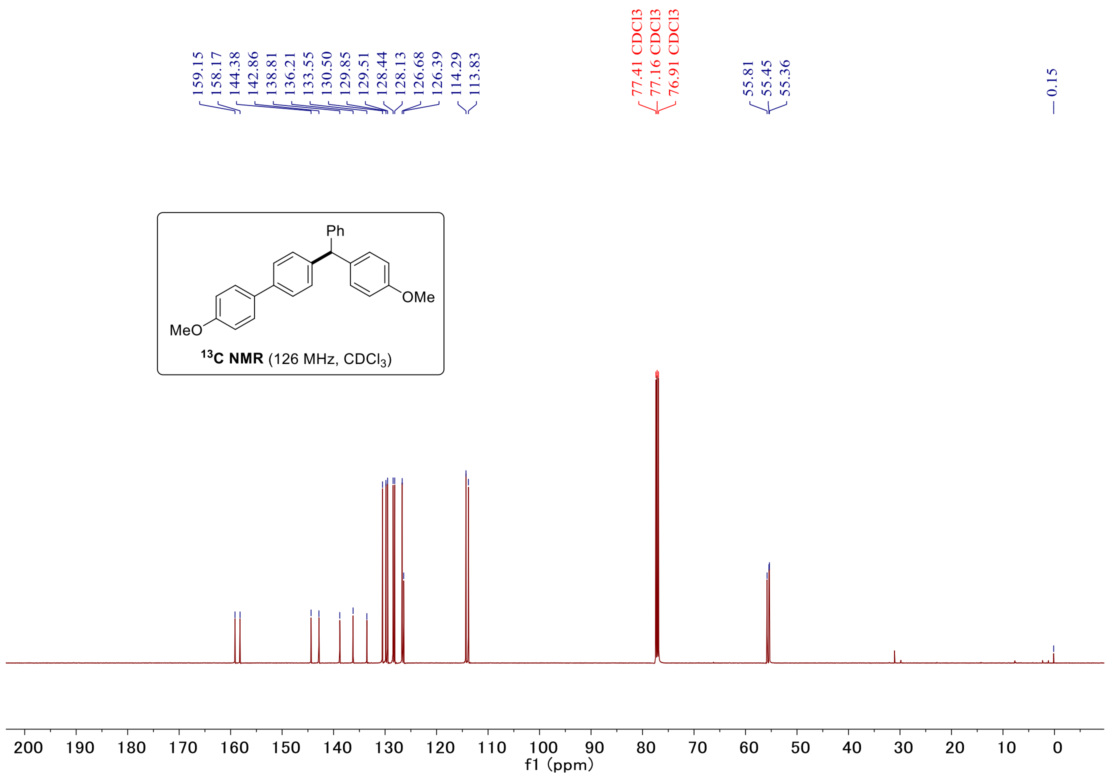

4-(Bis(4-methoxyphenyl)methyl)-4'-methoxybiphenyl (3kc)

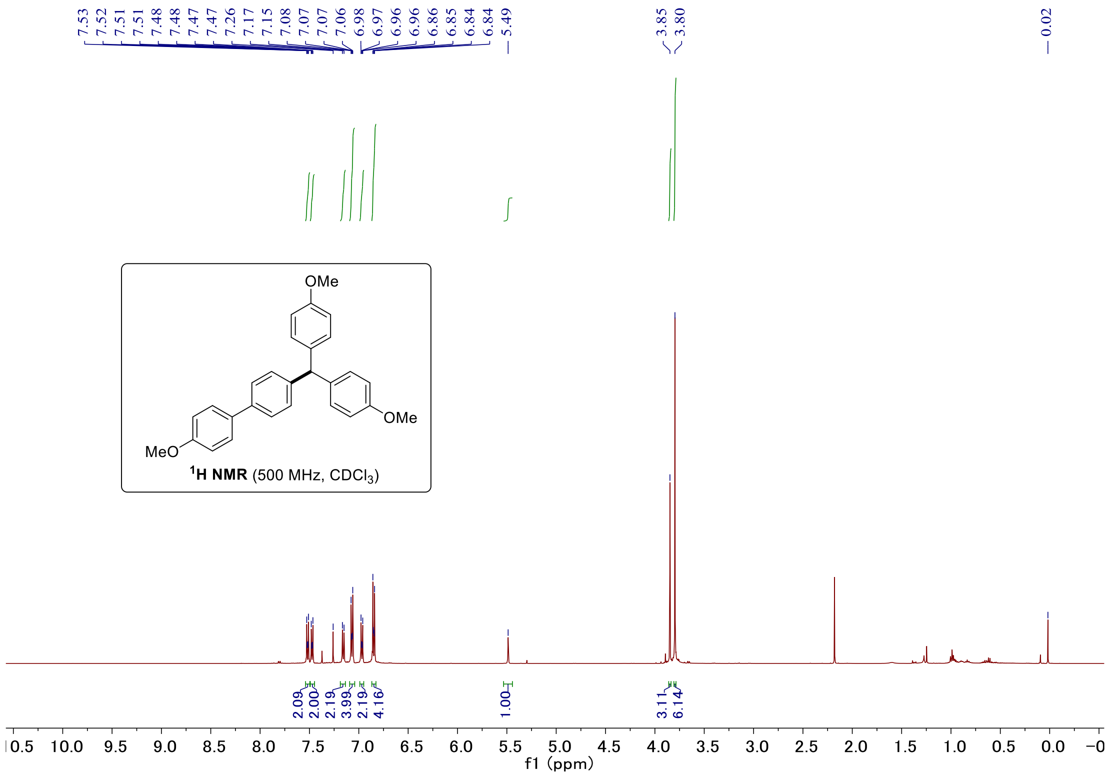

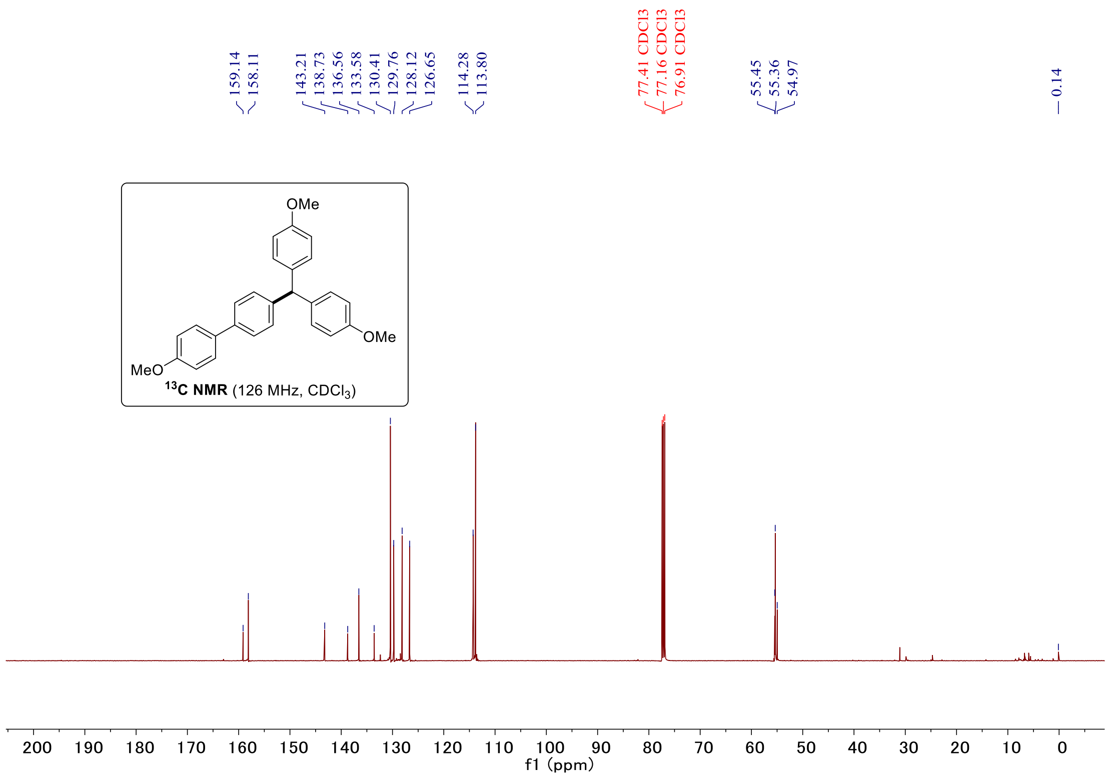

4-(Di-*p*-tolylmethyl)-4'-methoxybiphenyl (3kd)

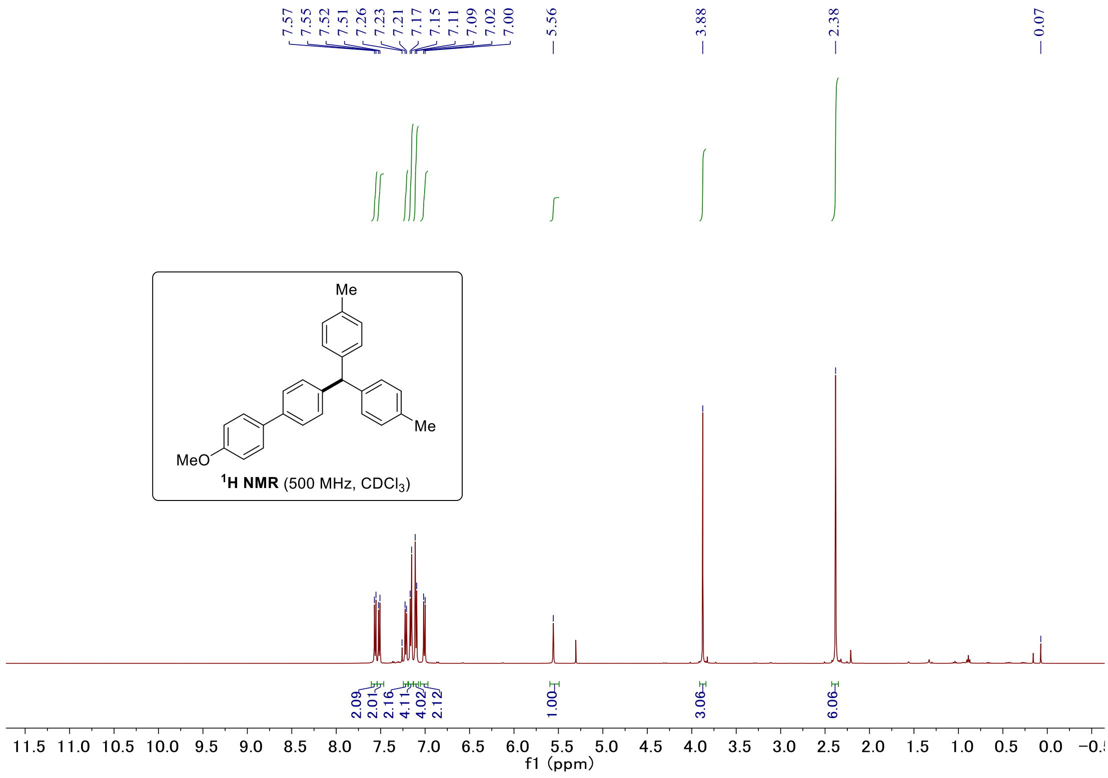

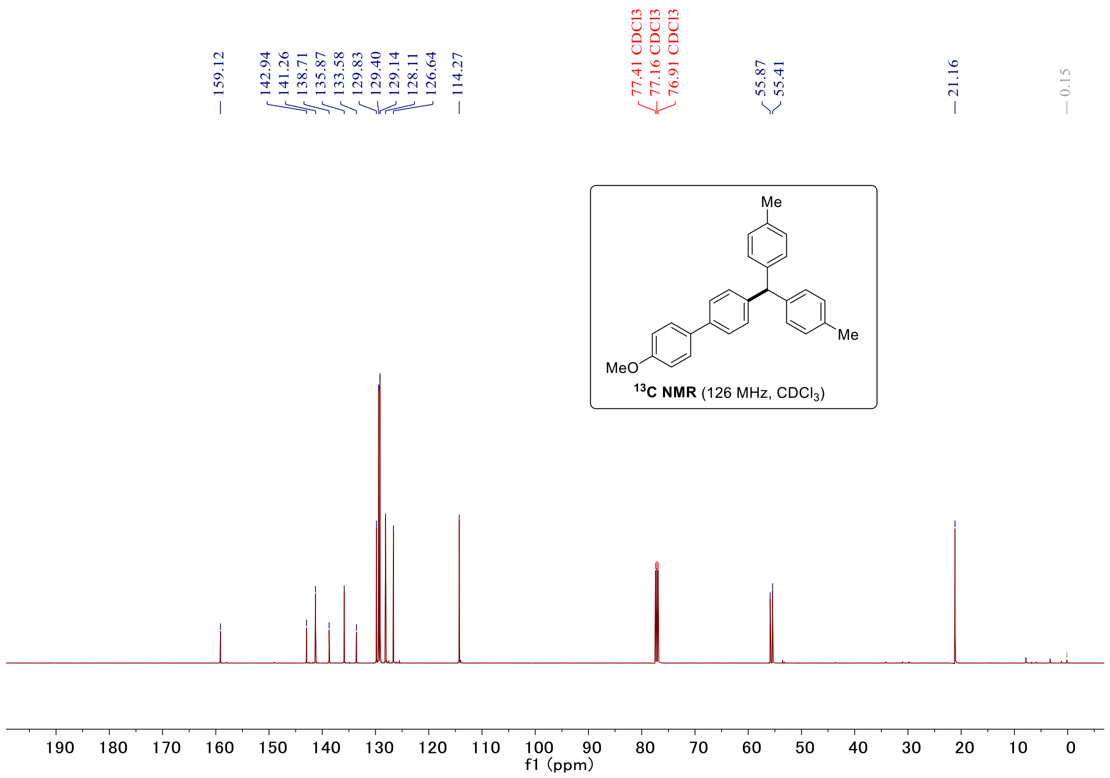

4-Methoxy-4'-(phenyl(*o*-tolyl)methyl)biphenyl (3ke)

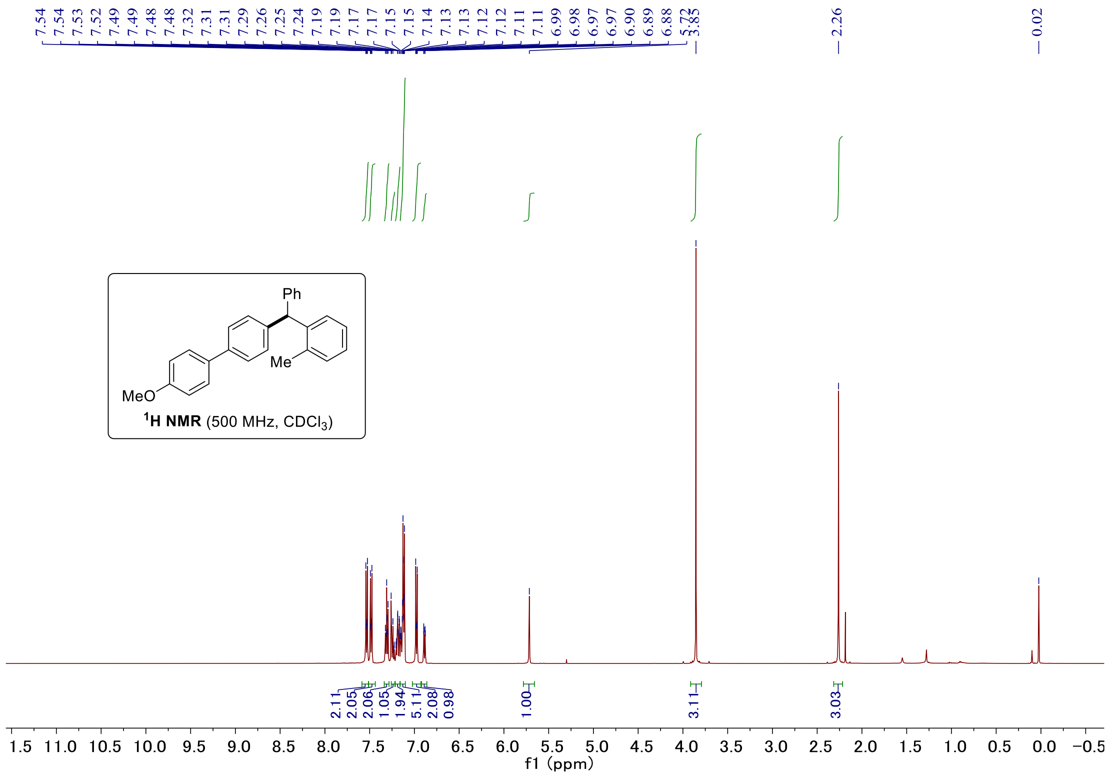

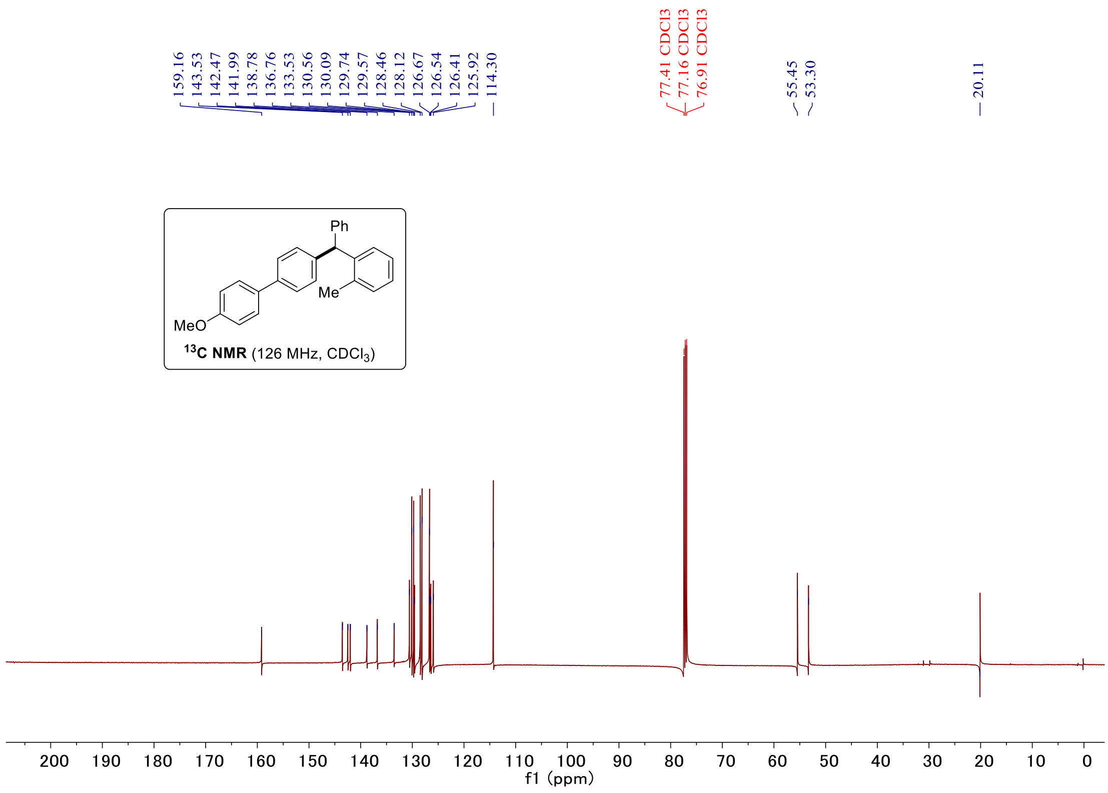

4-((4-Chlorophenyl)(phenyl)methyl)-4'-methoxybiphenyl (3kf)

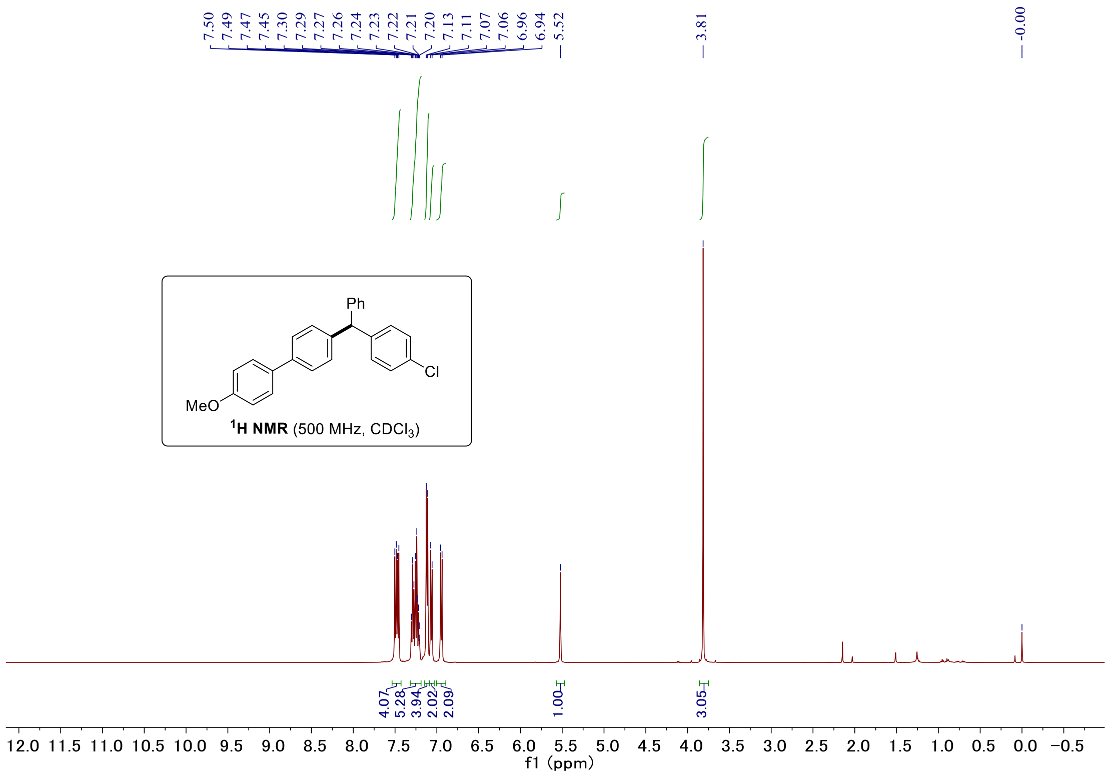

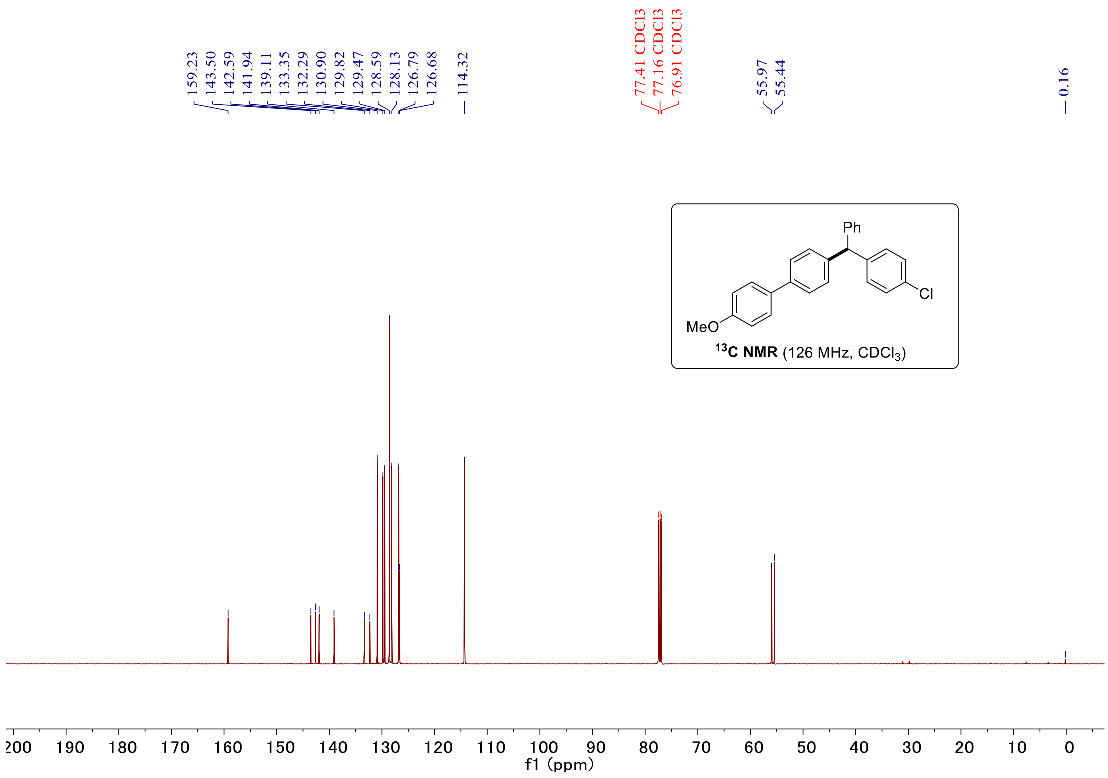

5-(4-((4-Methoxyphenyl)(phenyl)methyl)phenyl)benzo[*d*][1,3]dioxole (3nb)

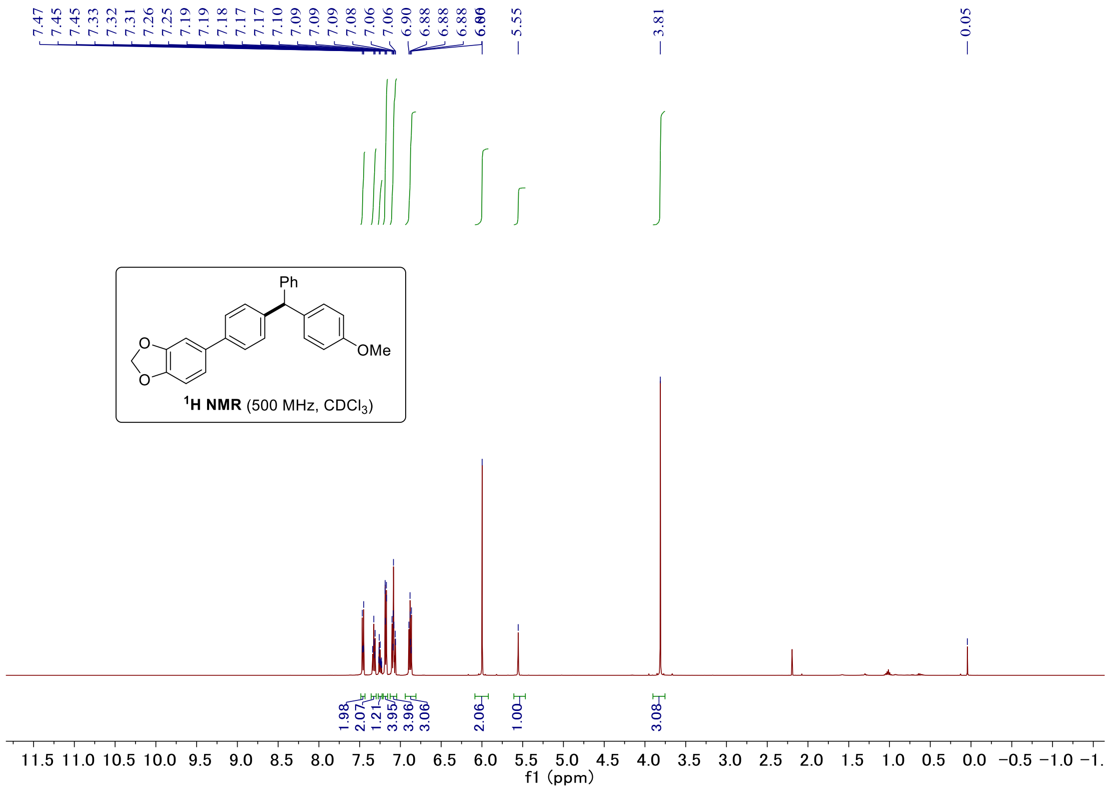

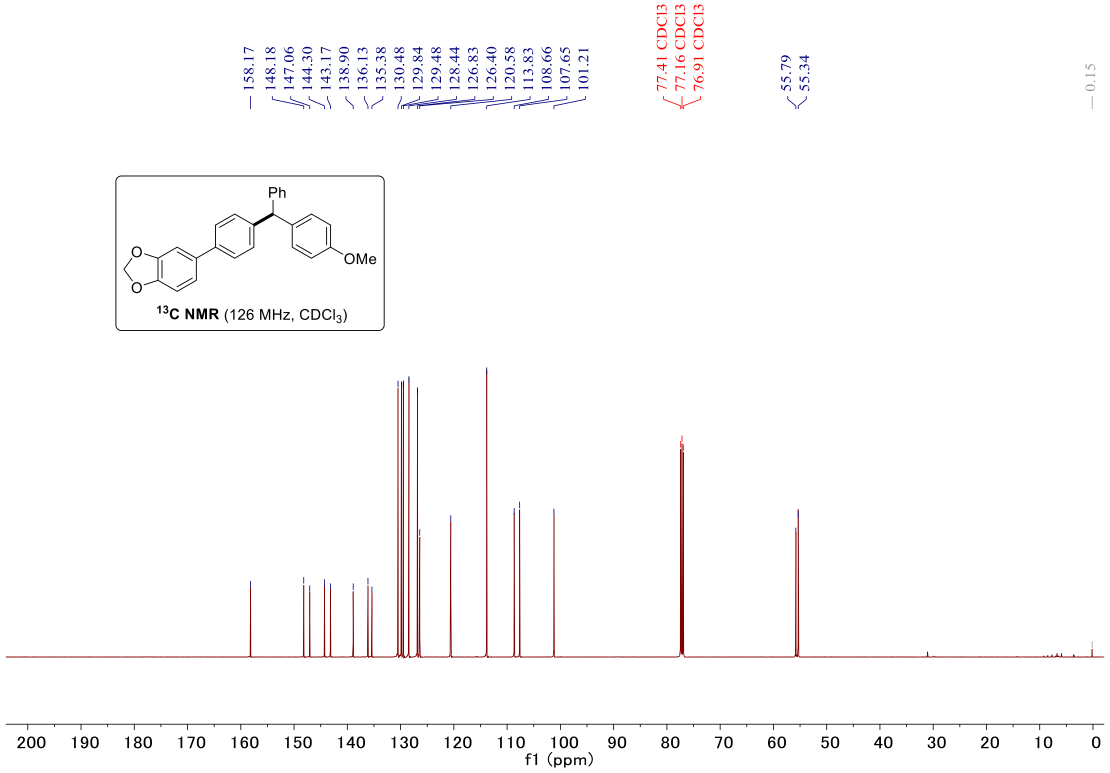

5-(4-(Bis(4-methoxyphenyl)methyl)phenyl)benzo[*d*][1,3]dioxole (3nc)

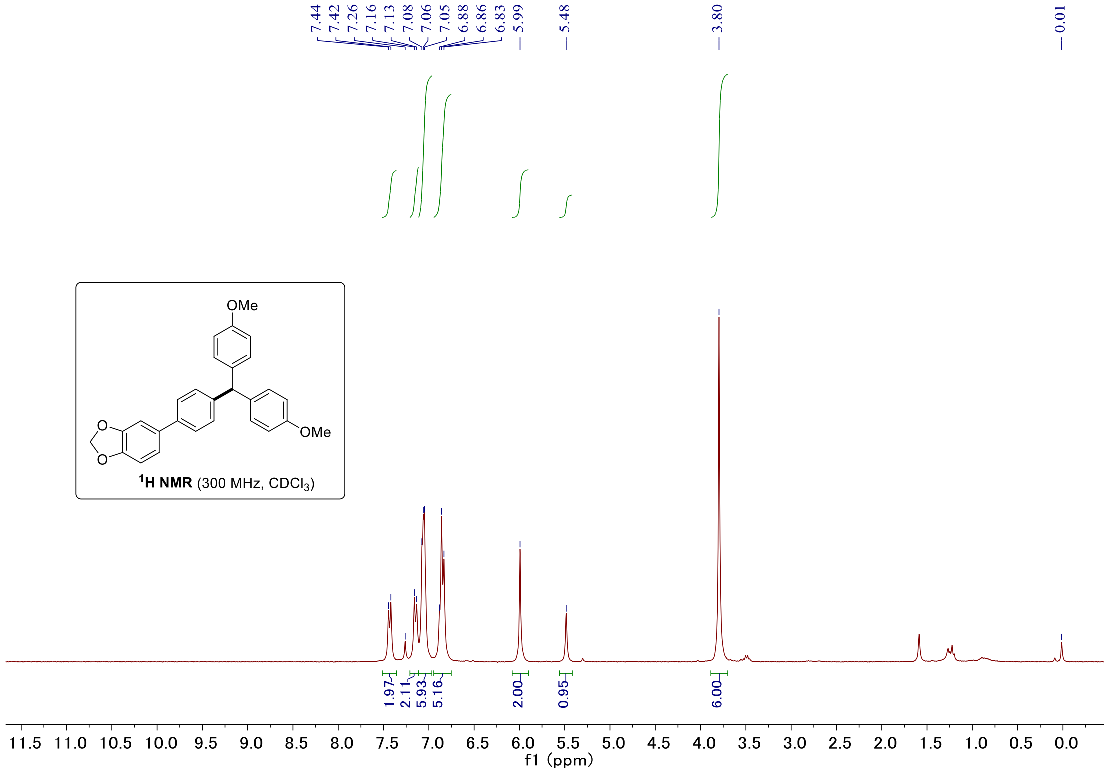

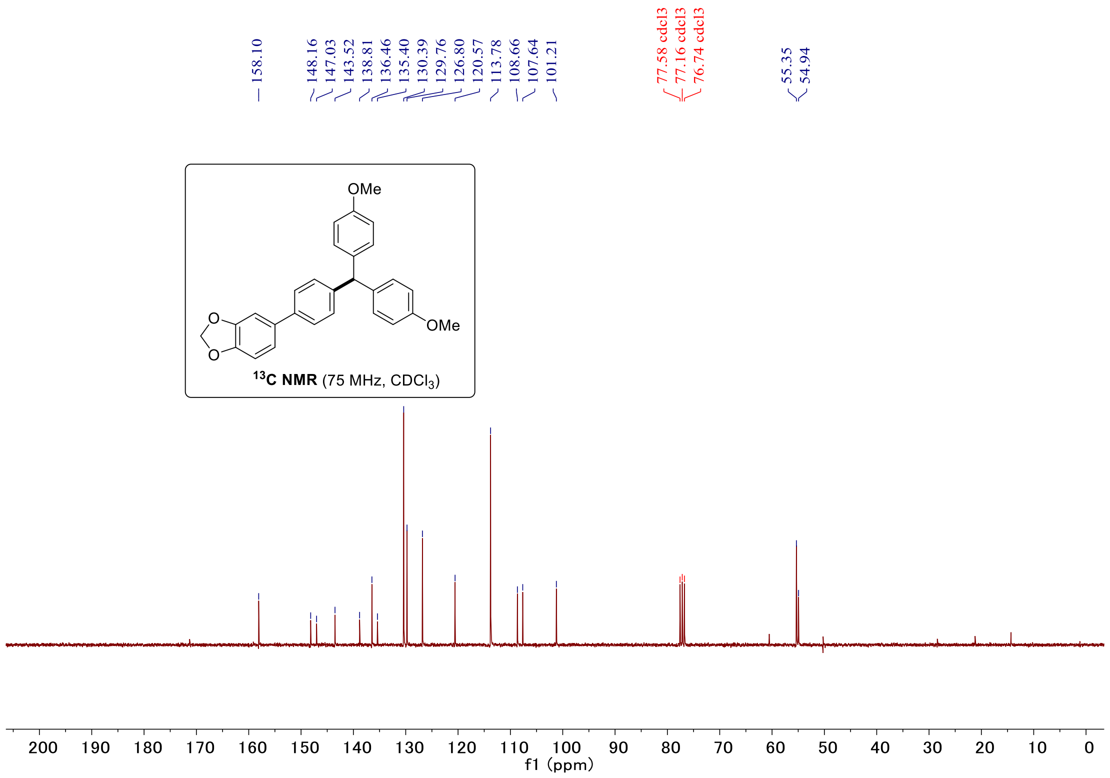

(4-((4-Methoxyphenyl)(phenyl)methyl)phenyl)-1*H*-pyrrole (3ob)

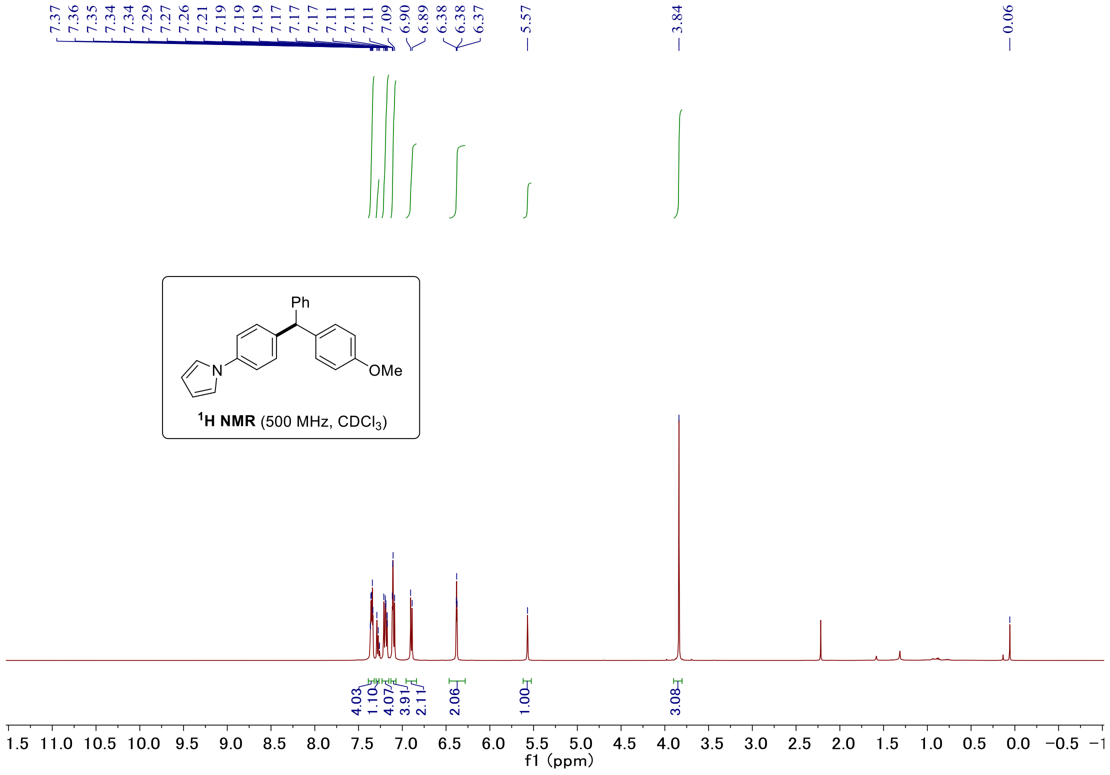

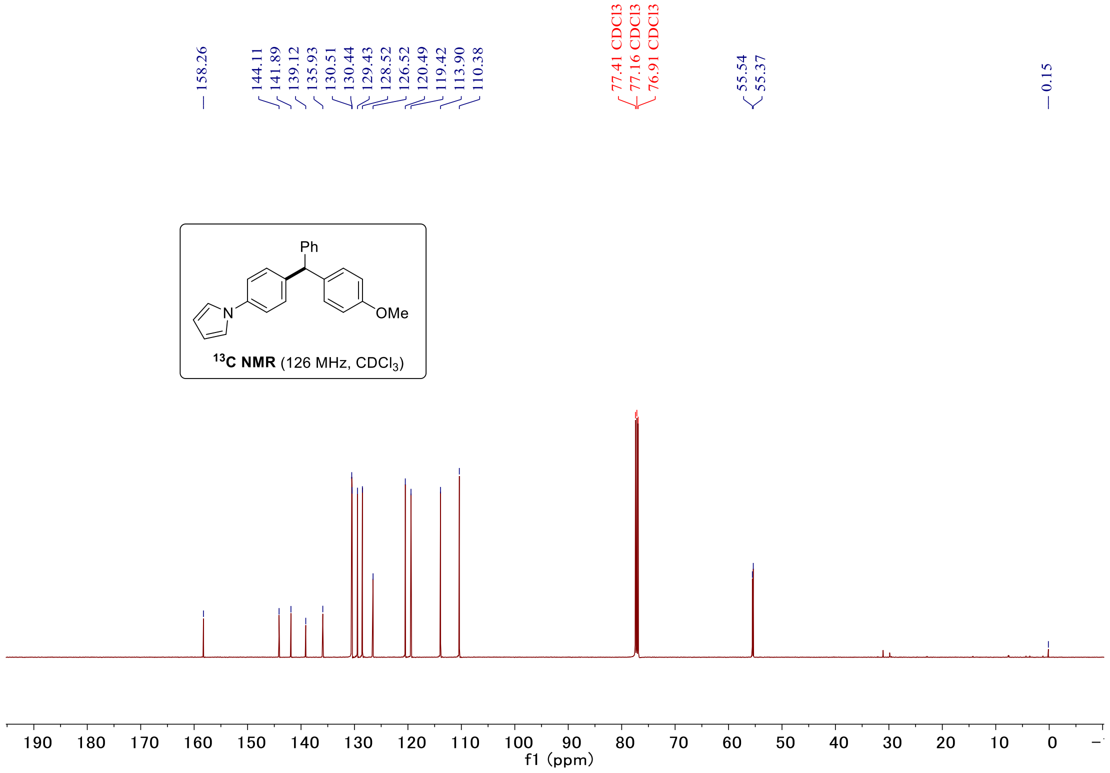

1-(4-(Phenyl(thiophen-2-yl)methyl)phenyl)-1*H*-pyrrole (3og)

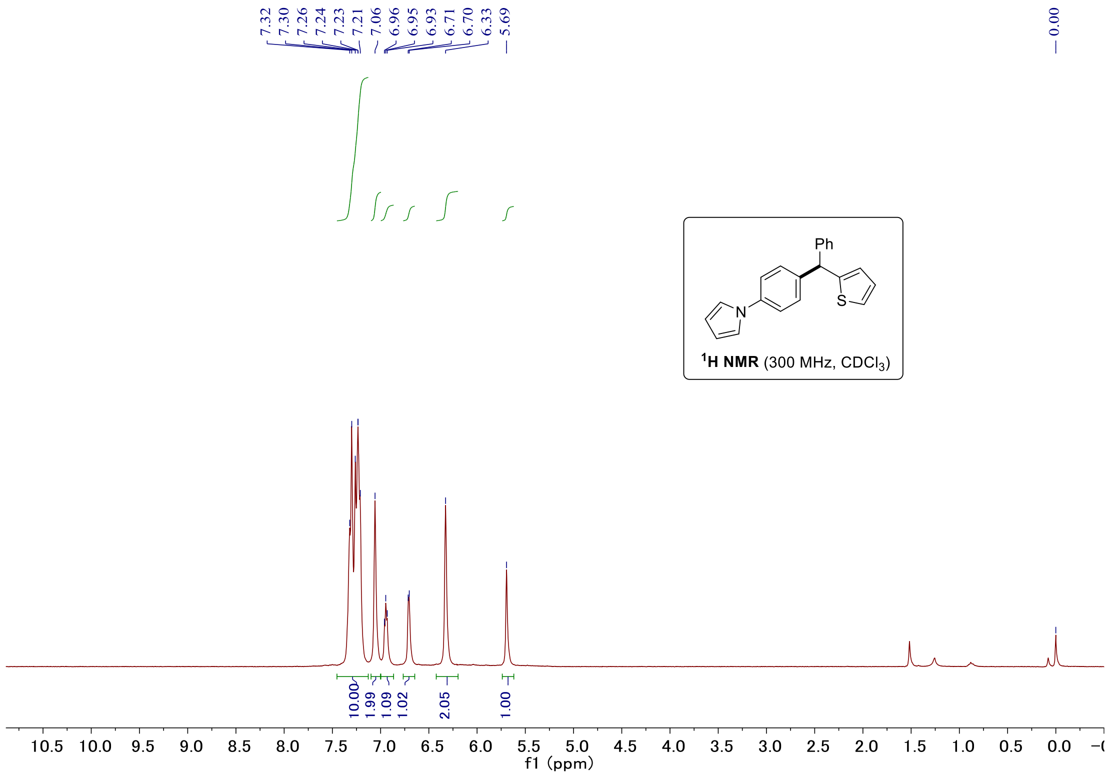

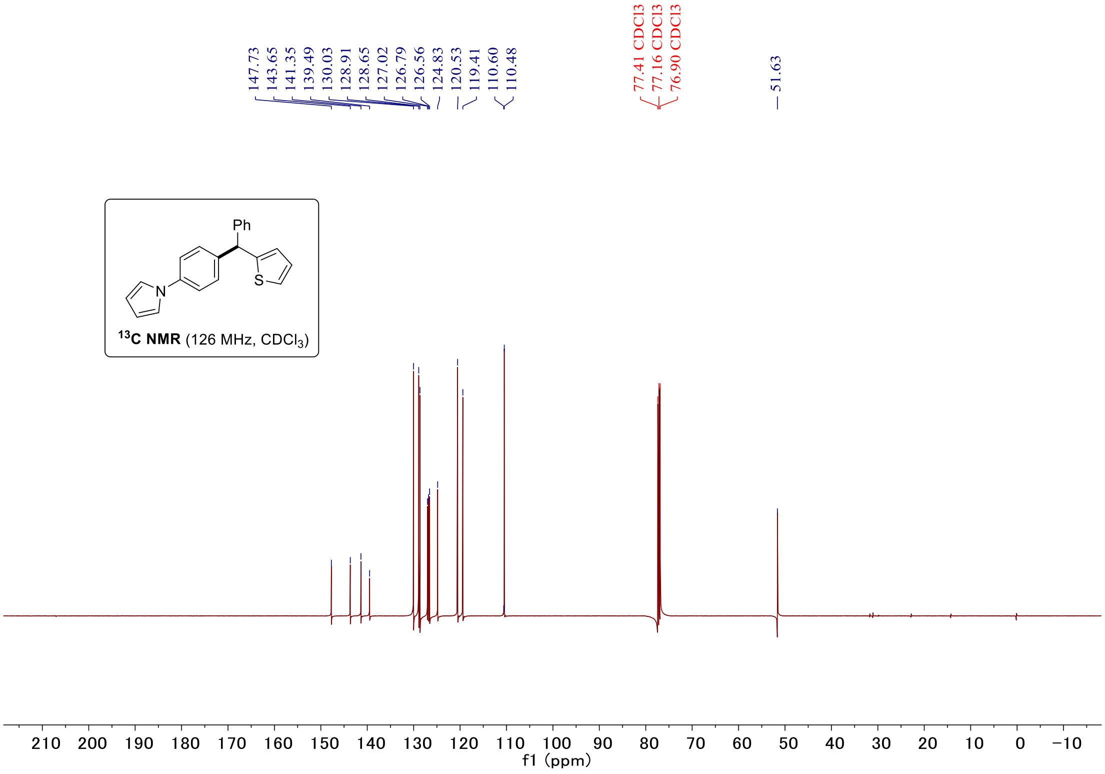

2-(4-((4-Methoxyphenyl)(phenyl)methyl)phenyl)-1-methyl-1*H*-indole (3pb)

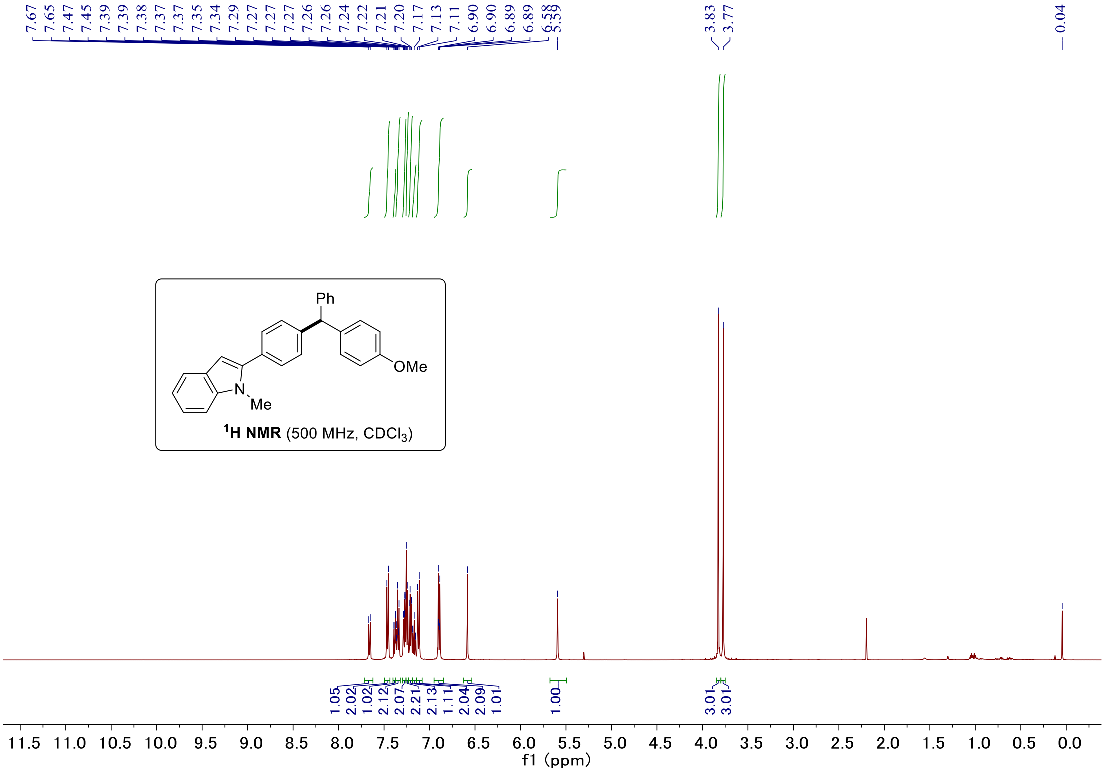

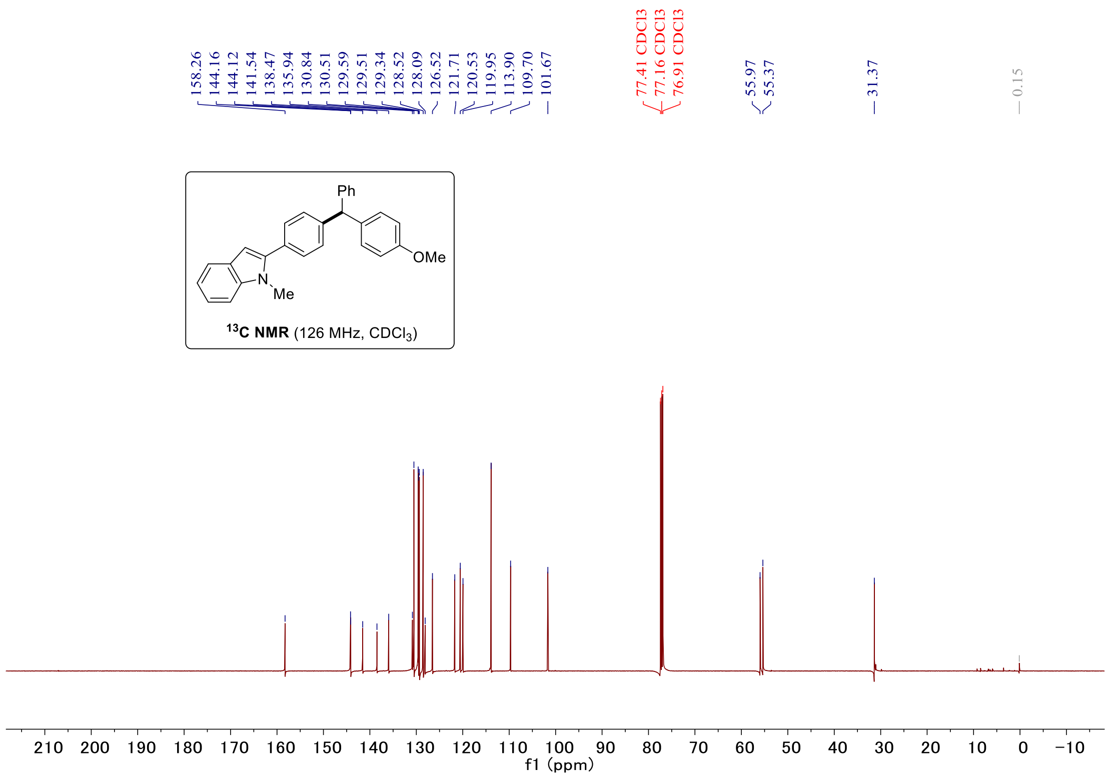

1-Methyl-2-(4-(phenyl(thiophen-2-yl)methyl)phenyl)-1*H*-indole (3pg)

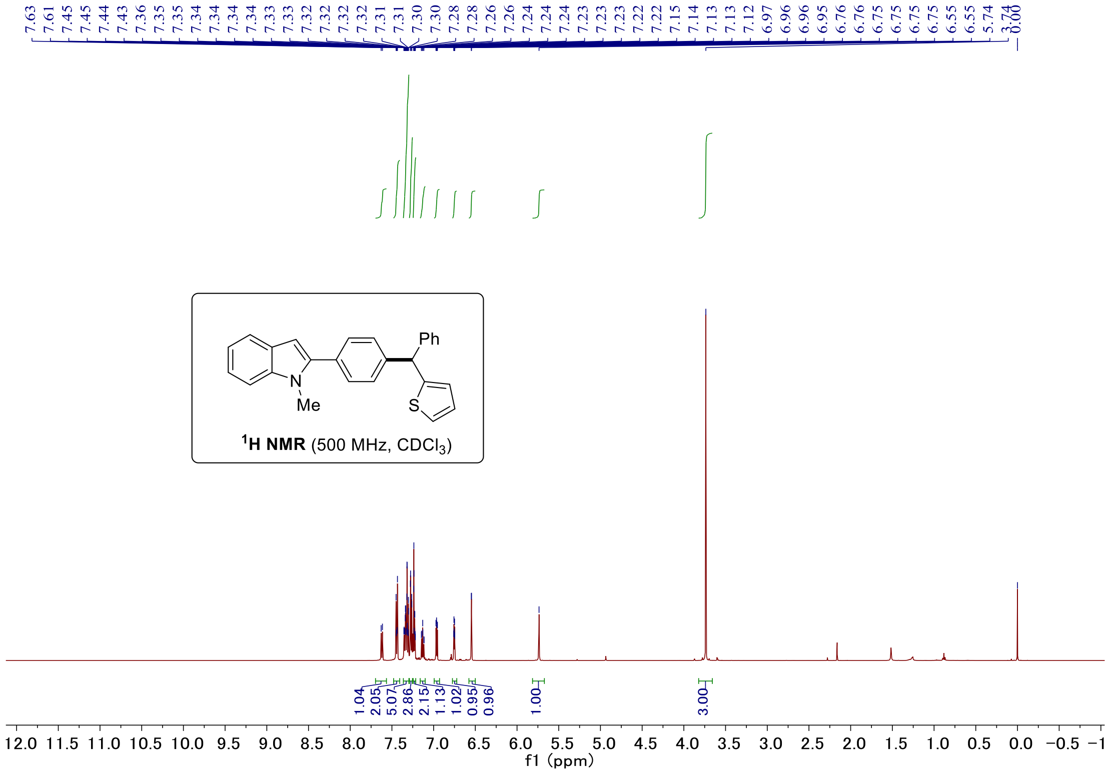

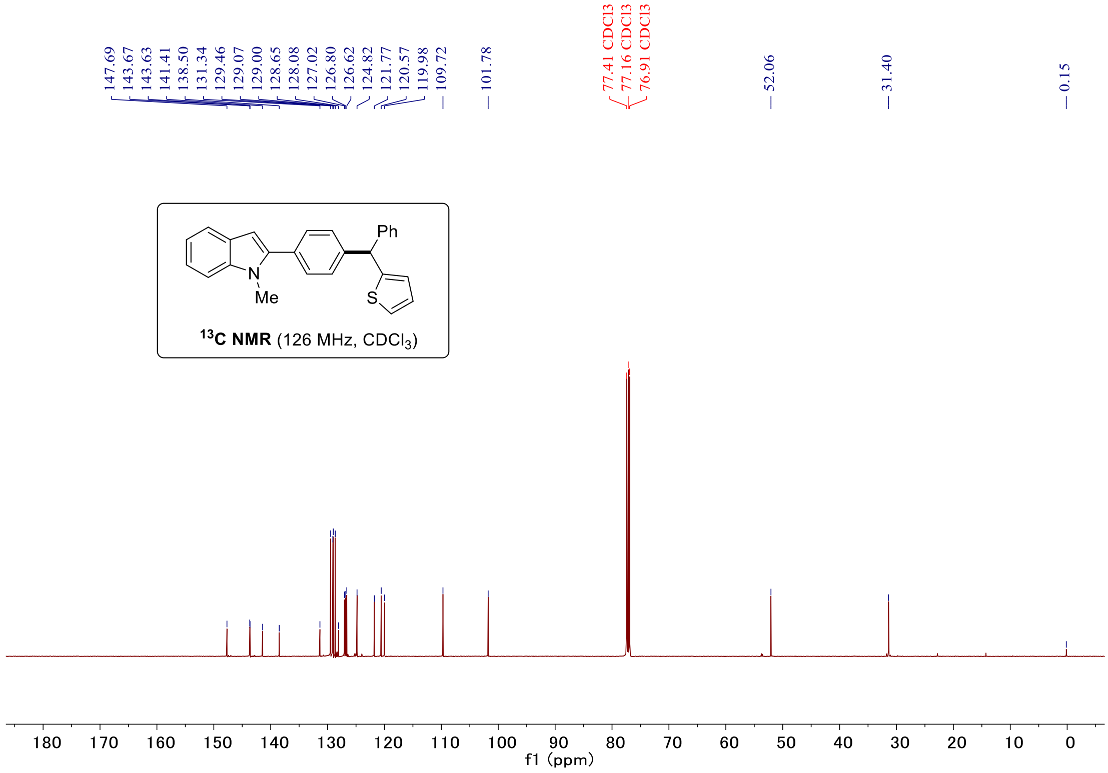

9-(Biphenyl]-4-yl)-9,10-dihydroanthracene (3ah)

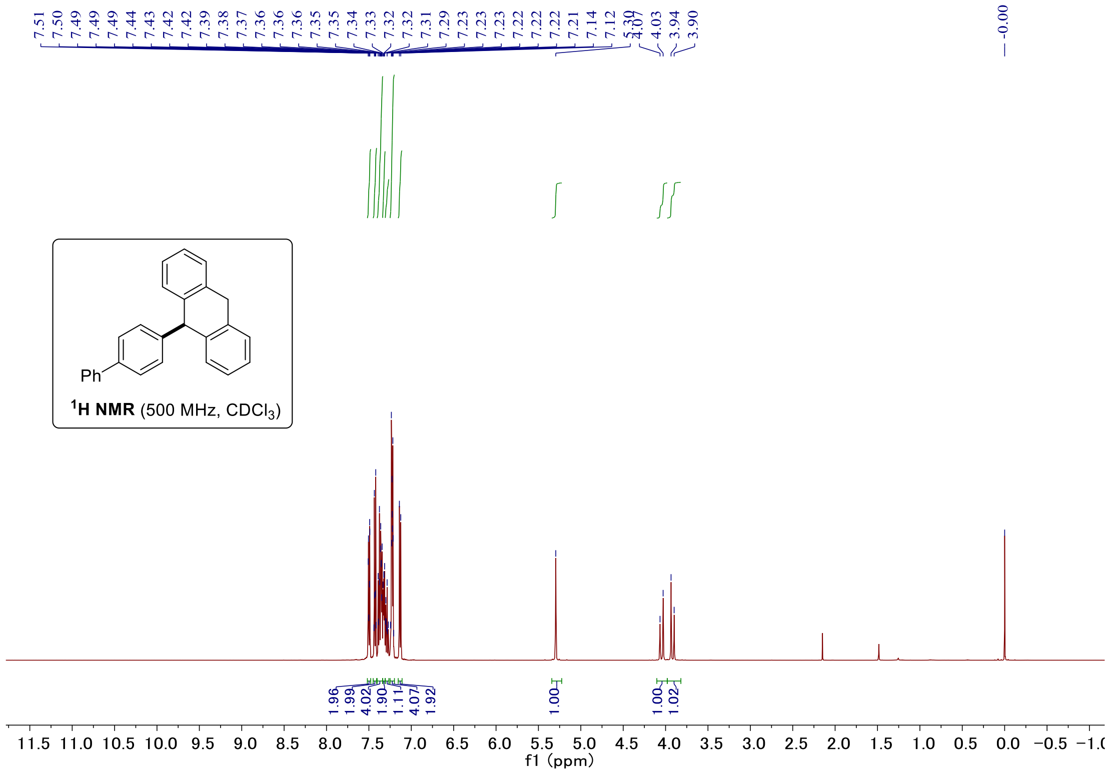

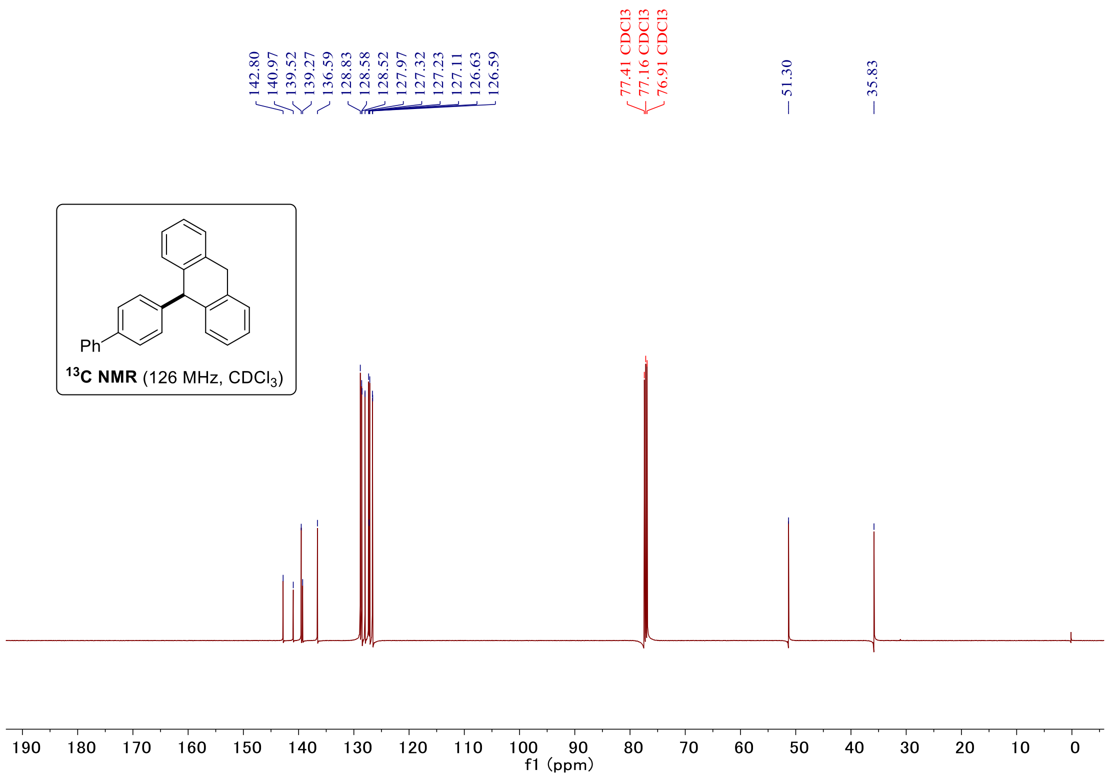

9-(Biphenyl-4-yl)-9*H*-xanthene (3ai)

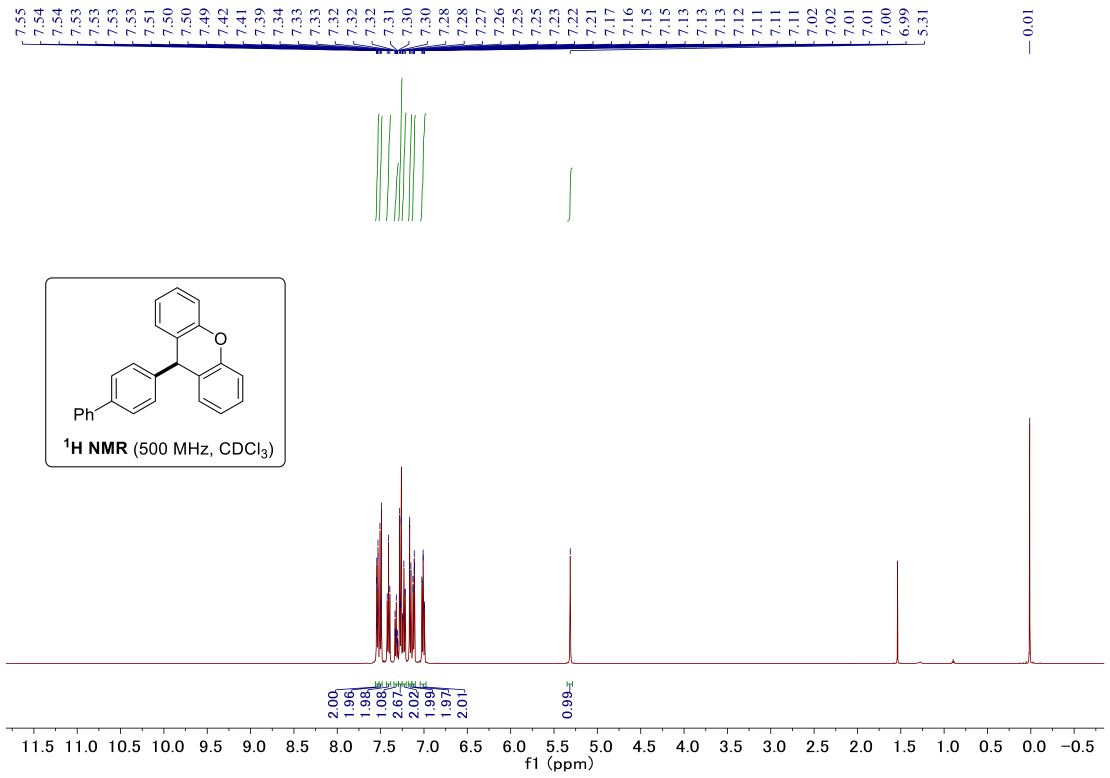

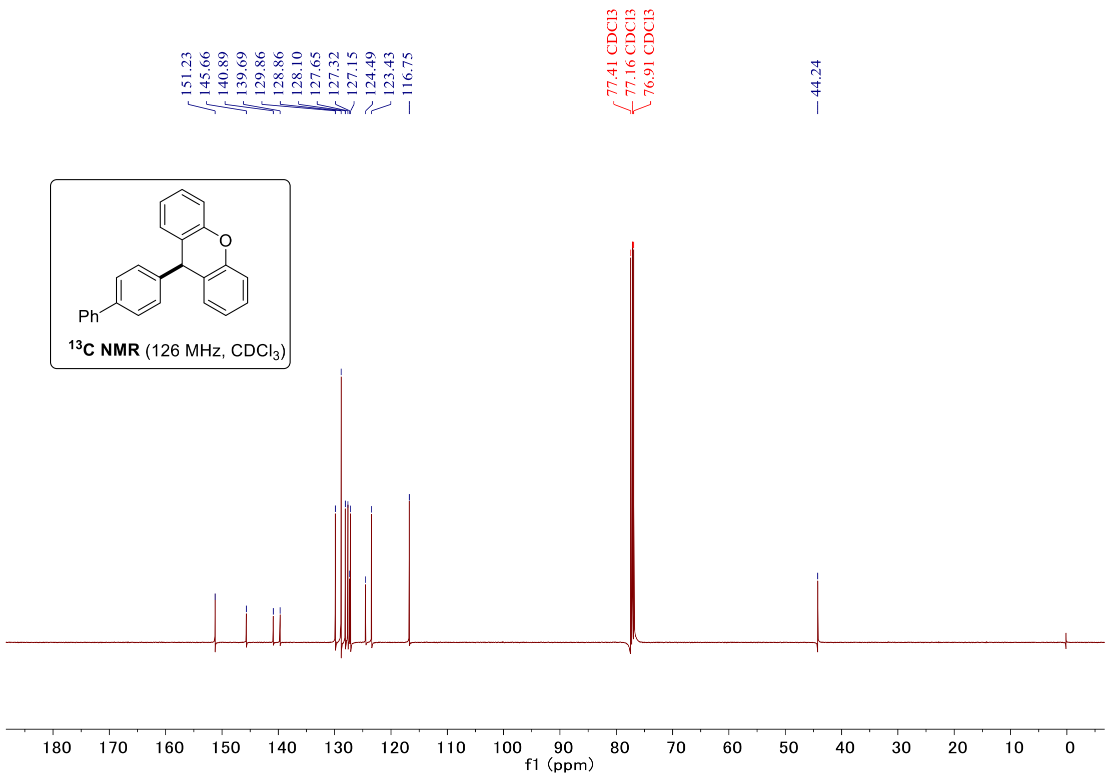

9-(4'-Methylbiphenyl-4-yl)-9,10-dihydroanthracene (3jh)

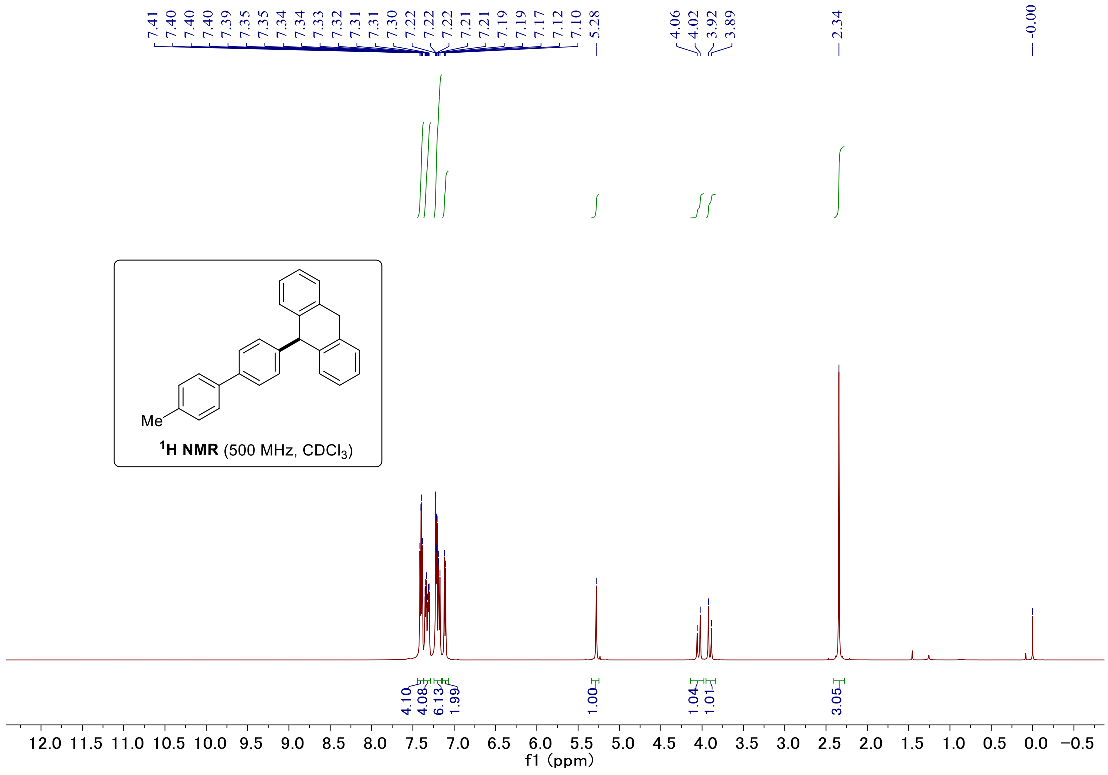

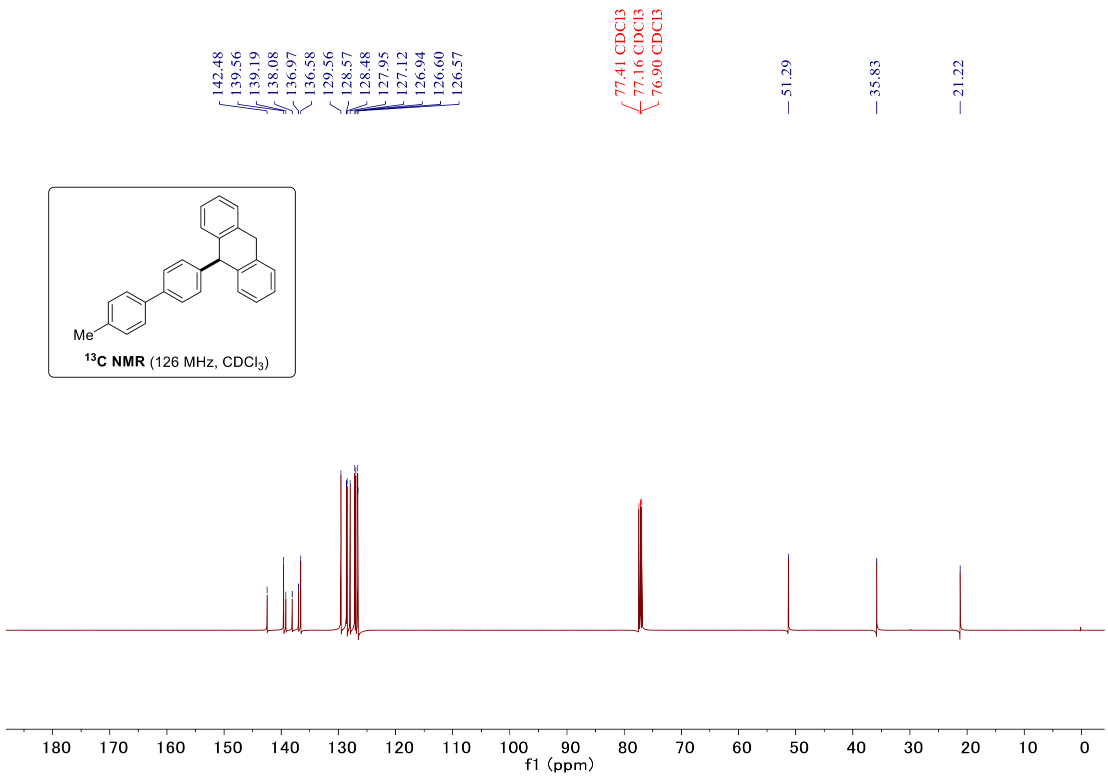

9-(4'-Methyl-biphenyl-4-yl)-9*H*-xanthene (3ji)

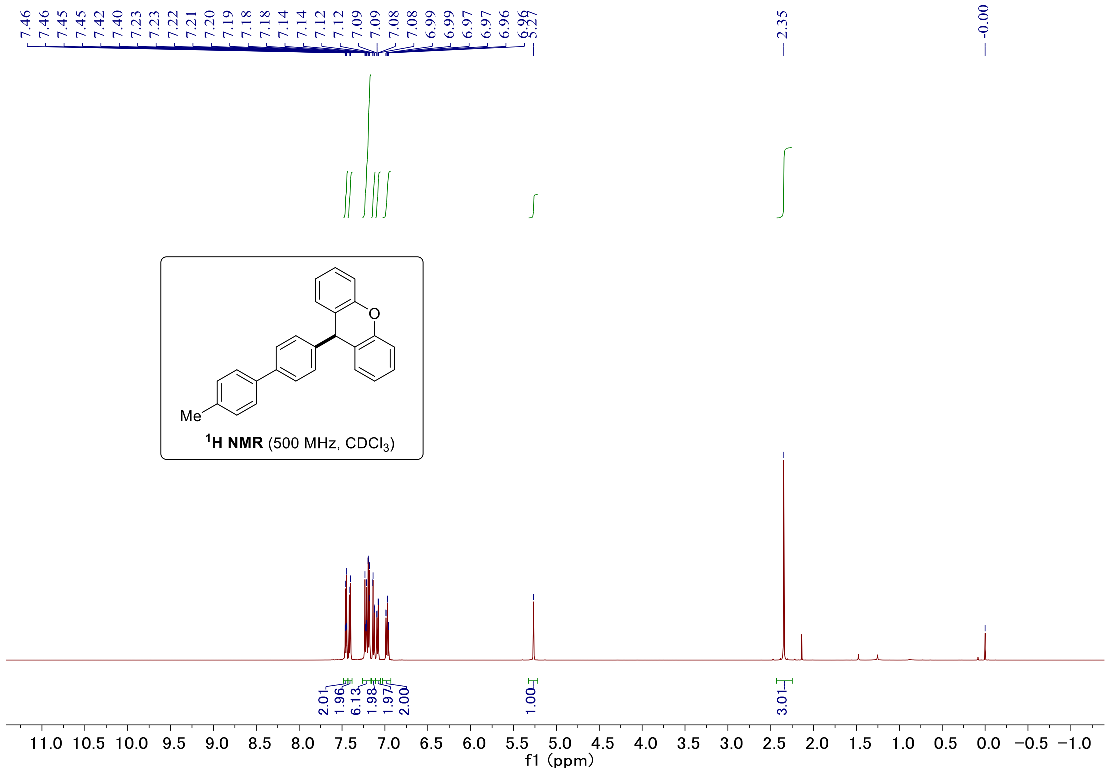

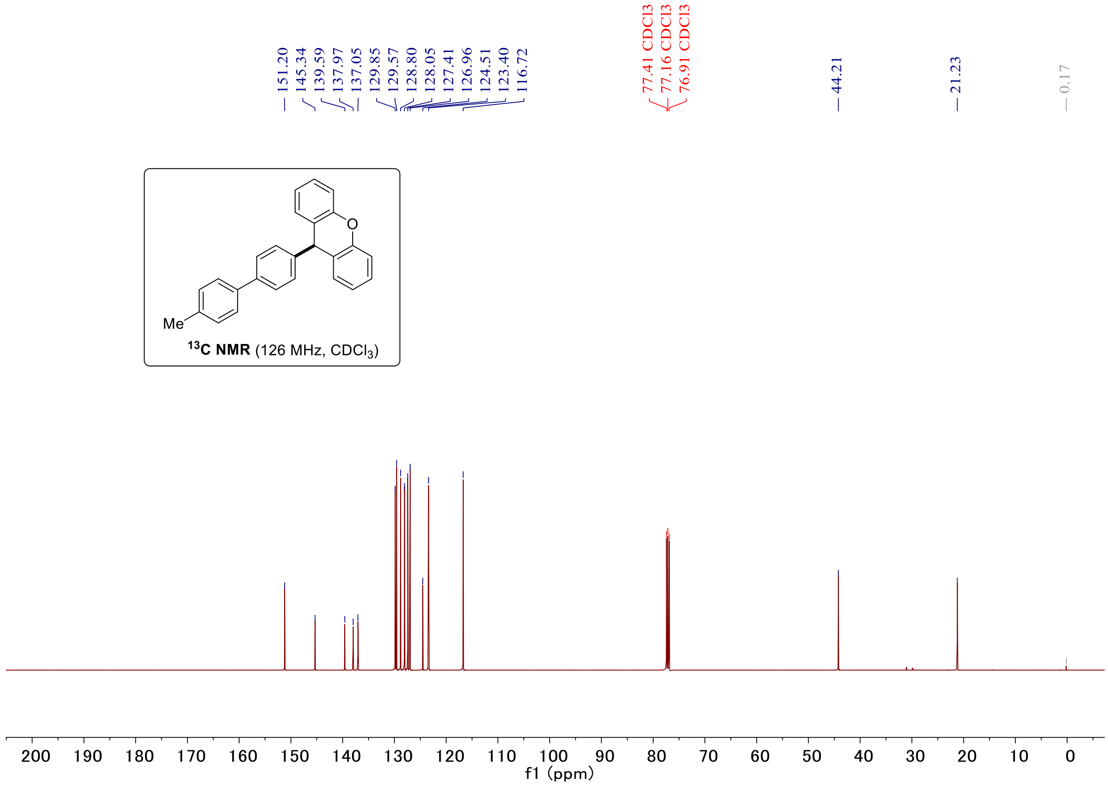

9-(4'-Methoxybiphenyl-4-yl)-9,10-dihydroanthracene (3kh)

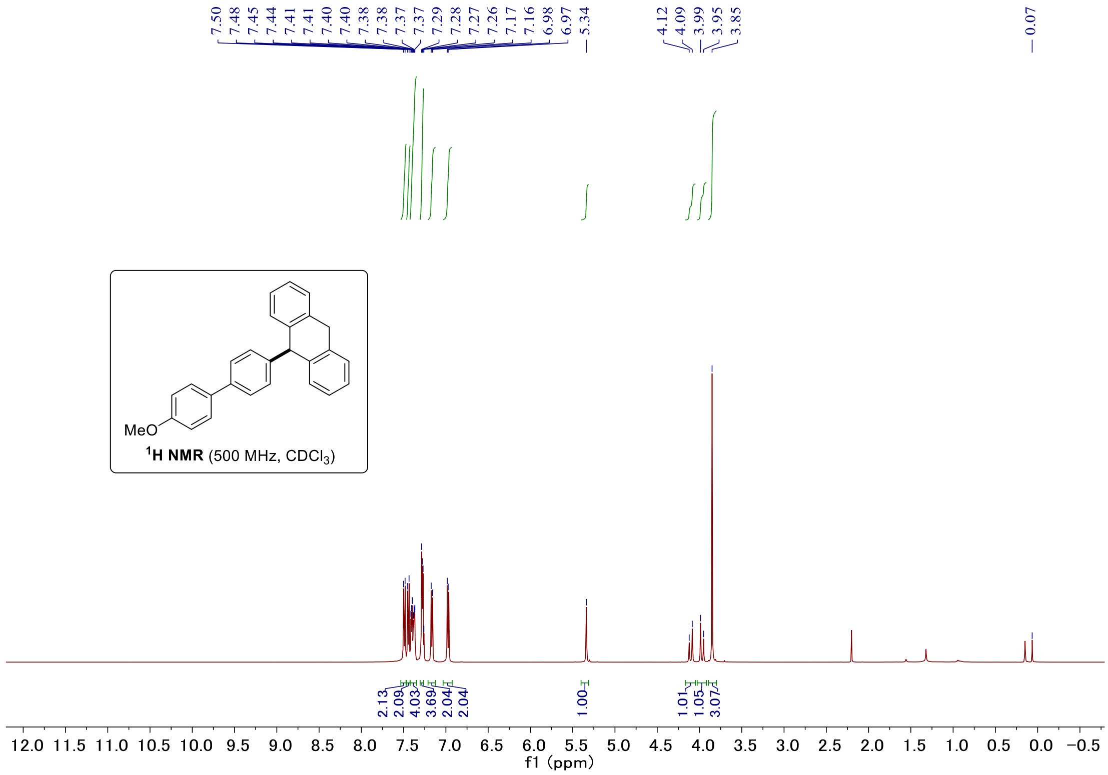

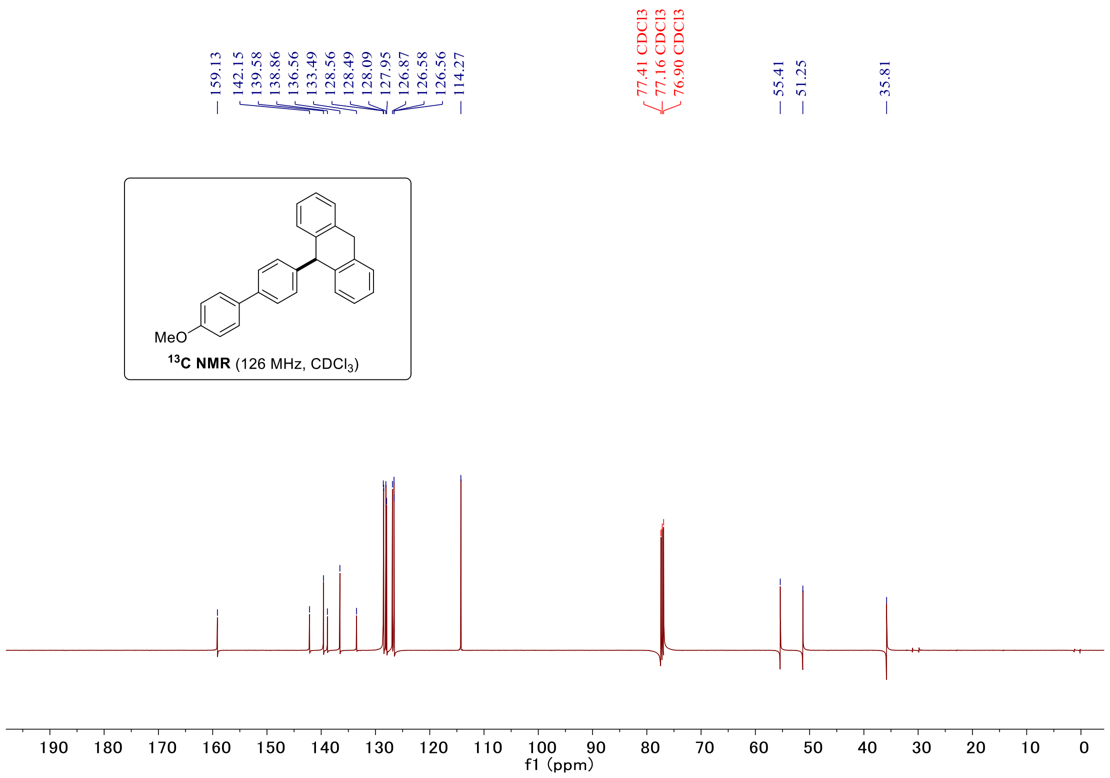

9-(4'-Methoxy-biphenyl-4-yl)-9*H*-xanthene (3ki)

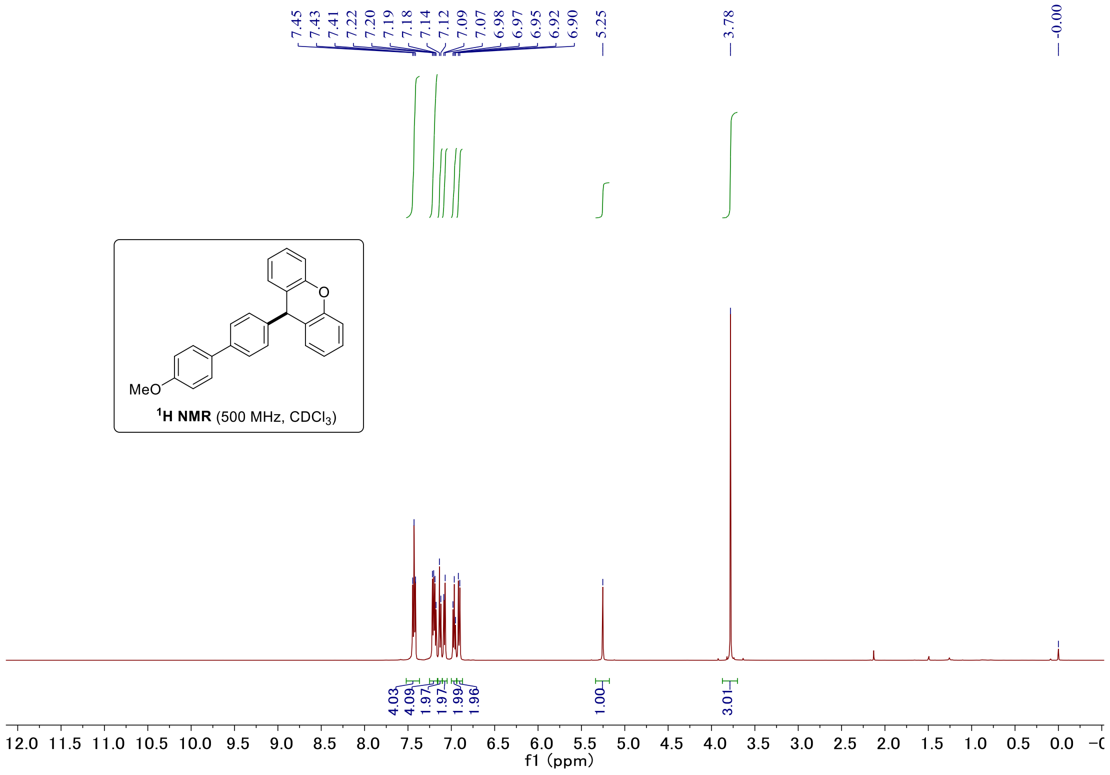

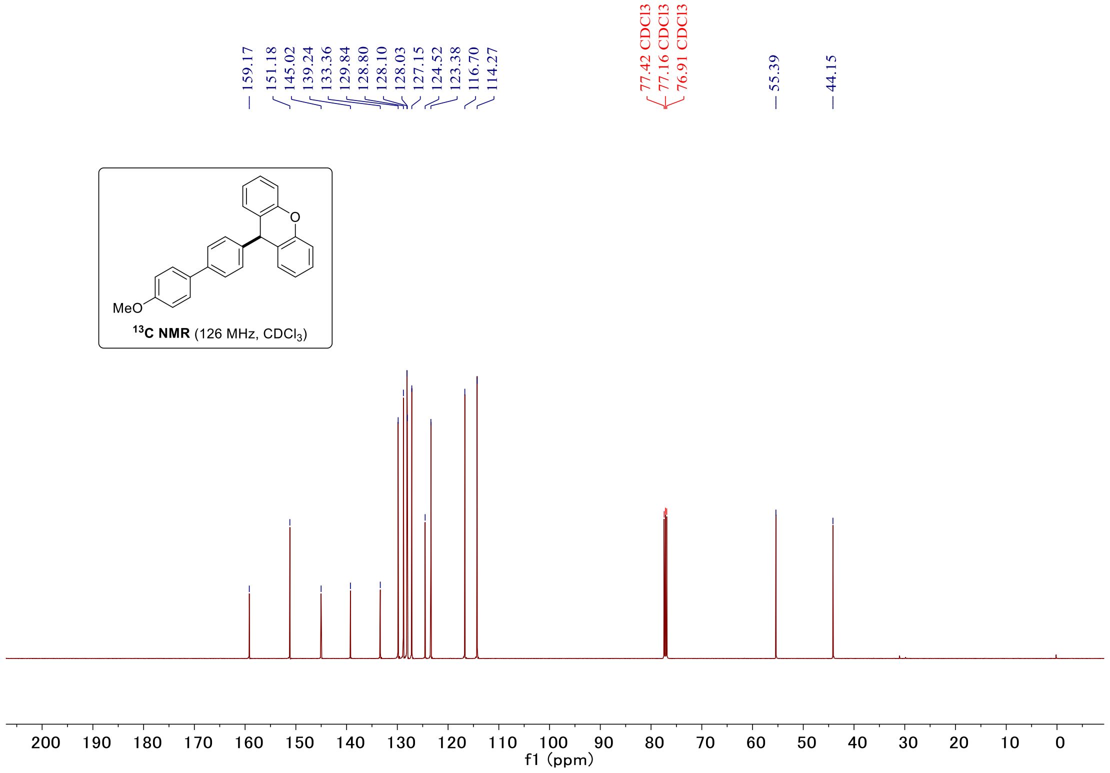

9-(4'-(Benzyloxy)-[1,1'-biphenyl]-4-yl)-9,10-dihydroanthracene (3lh)

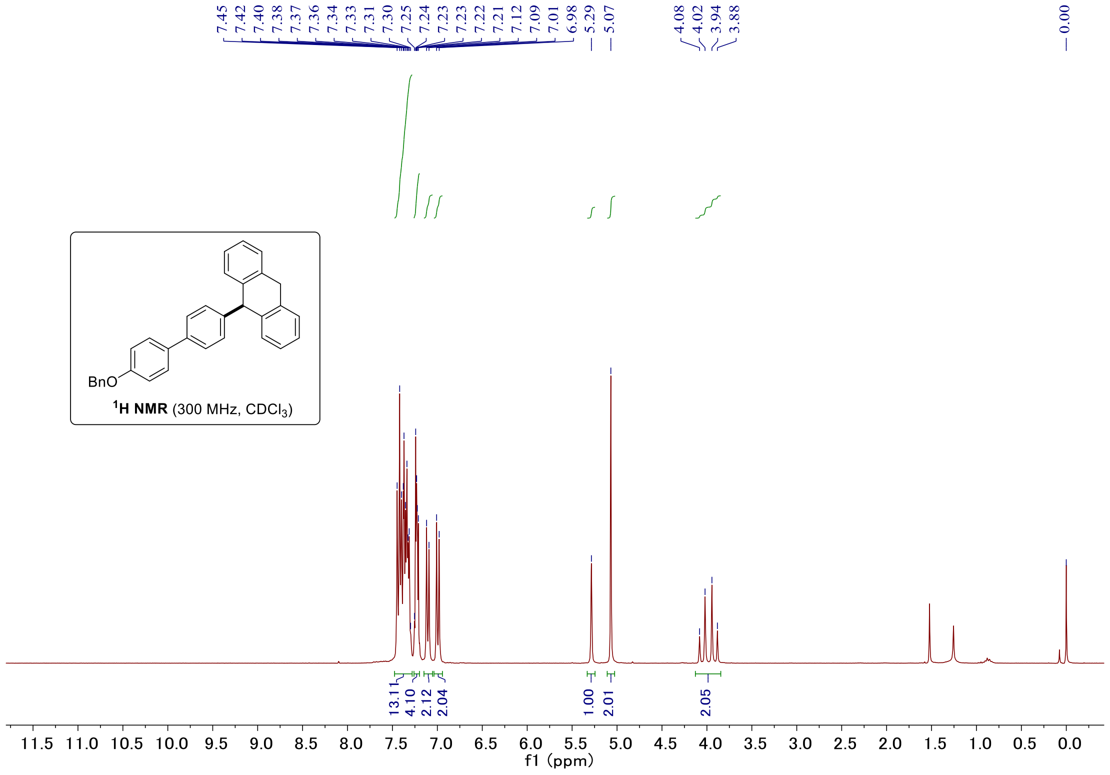

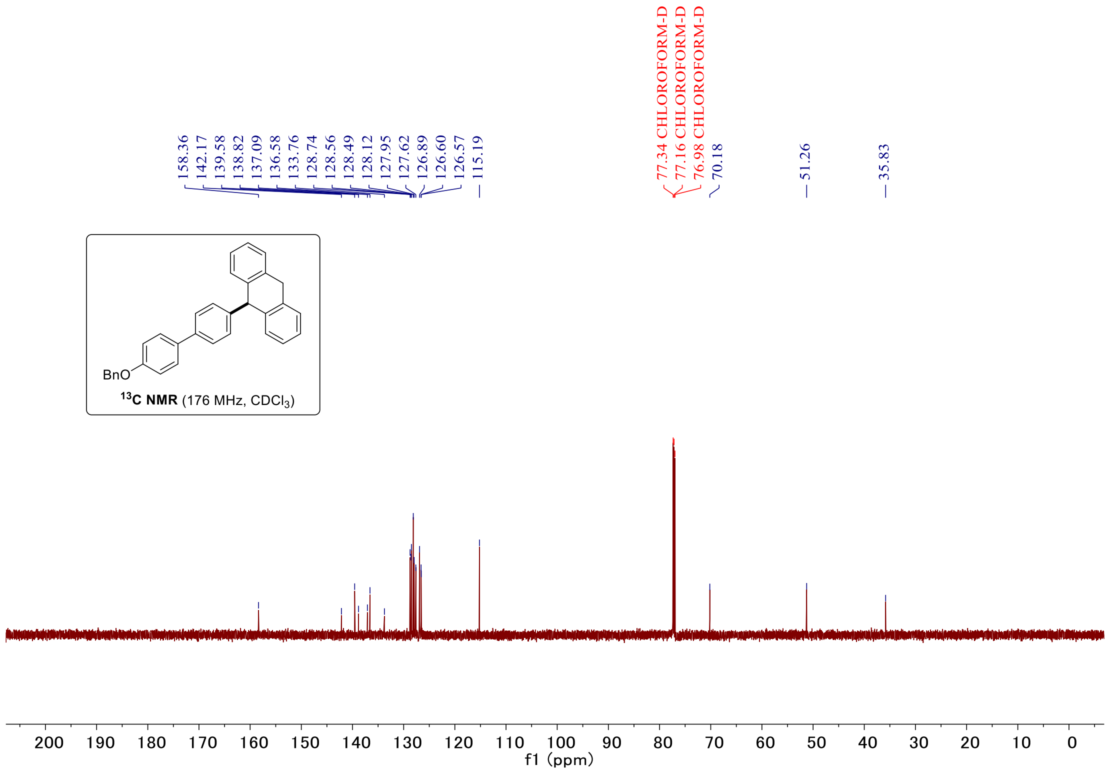

5-(4-(9,10-Dihydroanthracen-9-yl)phenyl)benzo[*d*][1,3]dioxole (3nh)

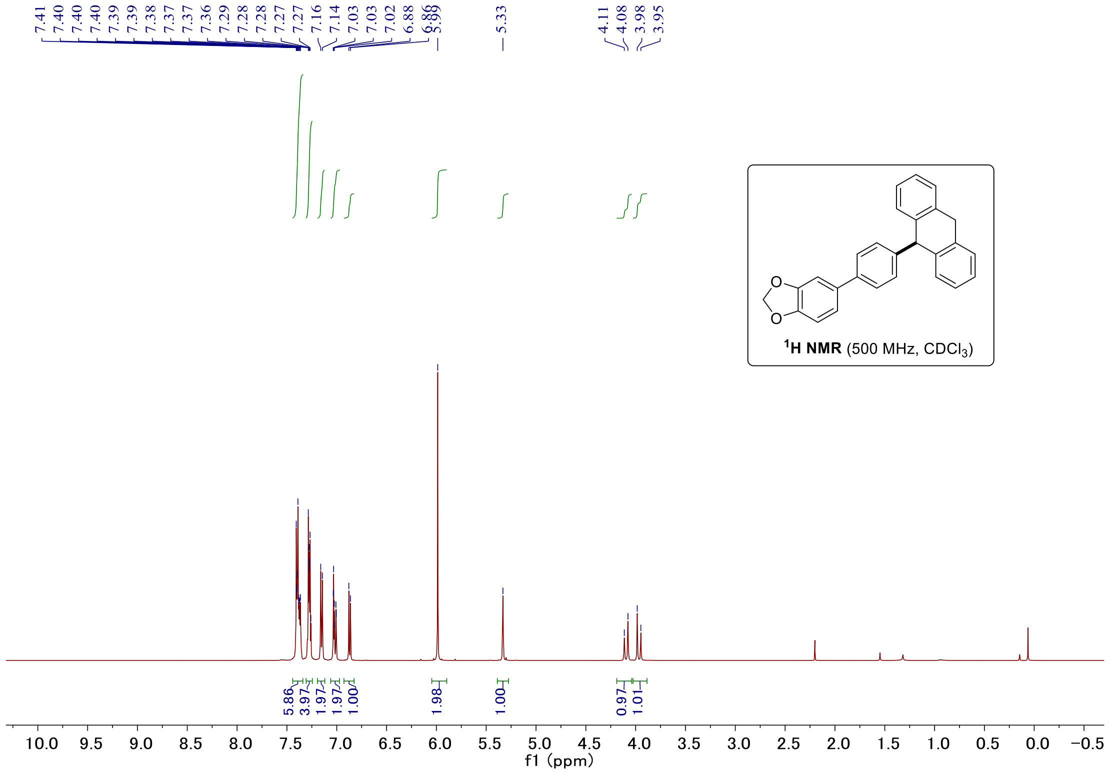

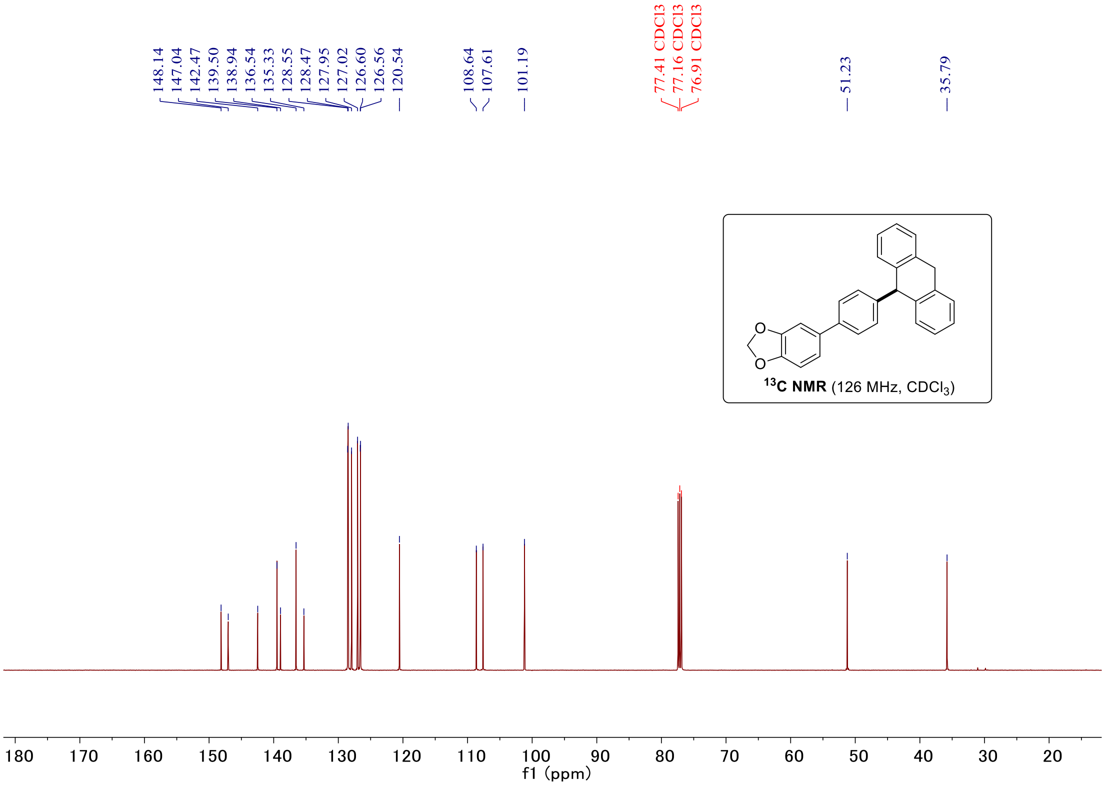

9-(4-(Benzo[*d*][1,3]dioxol-5-yl)phenyl)-9*H*-xanthene (3ni)

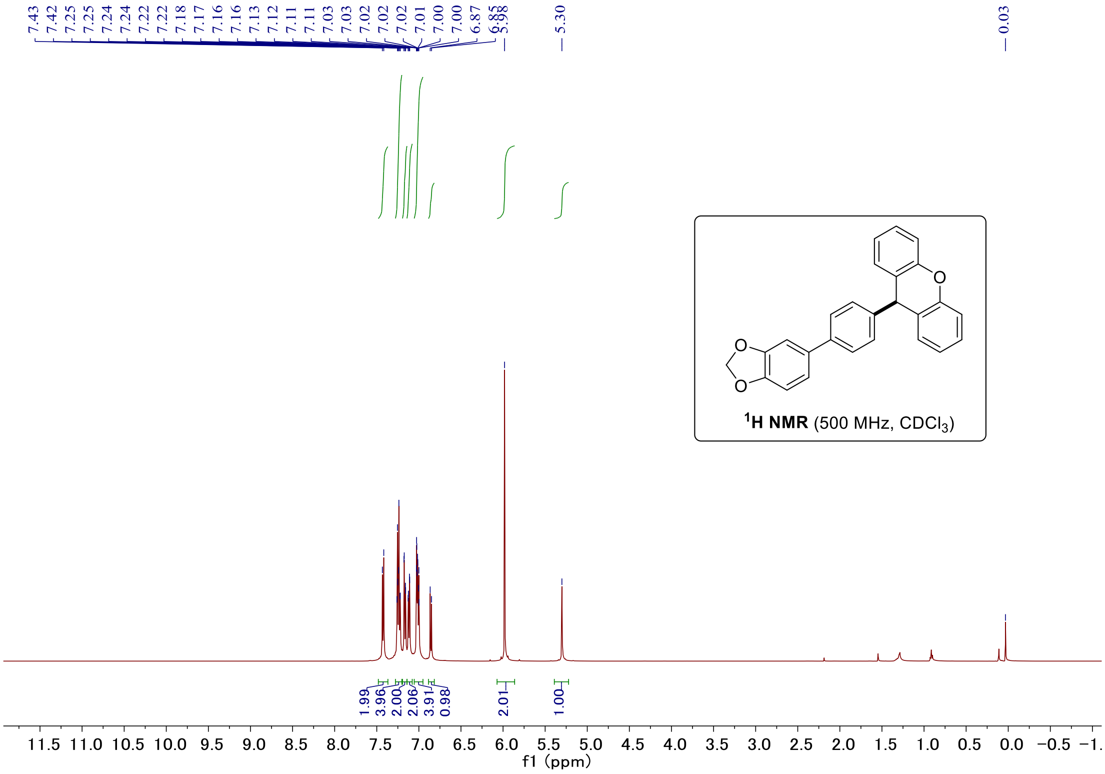

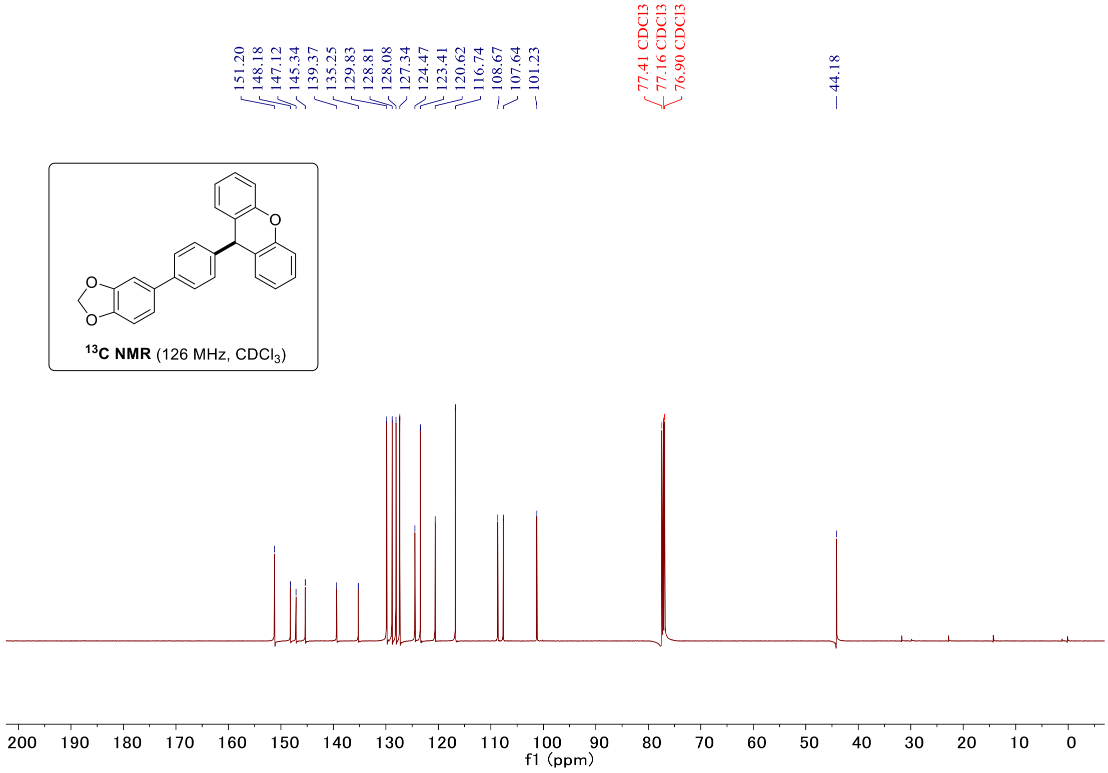

2-(4-(9*H*-Xanthen-9-yl)phenyl)-1-methyl-1*H*-indole (3pi)

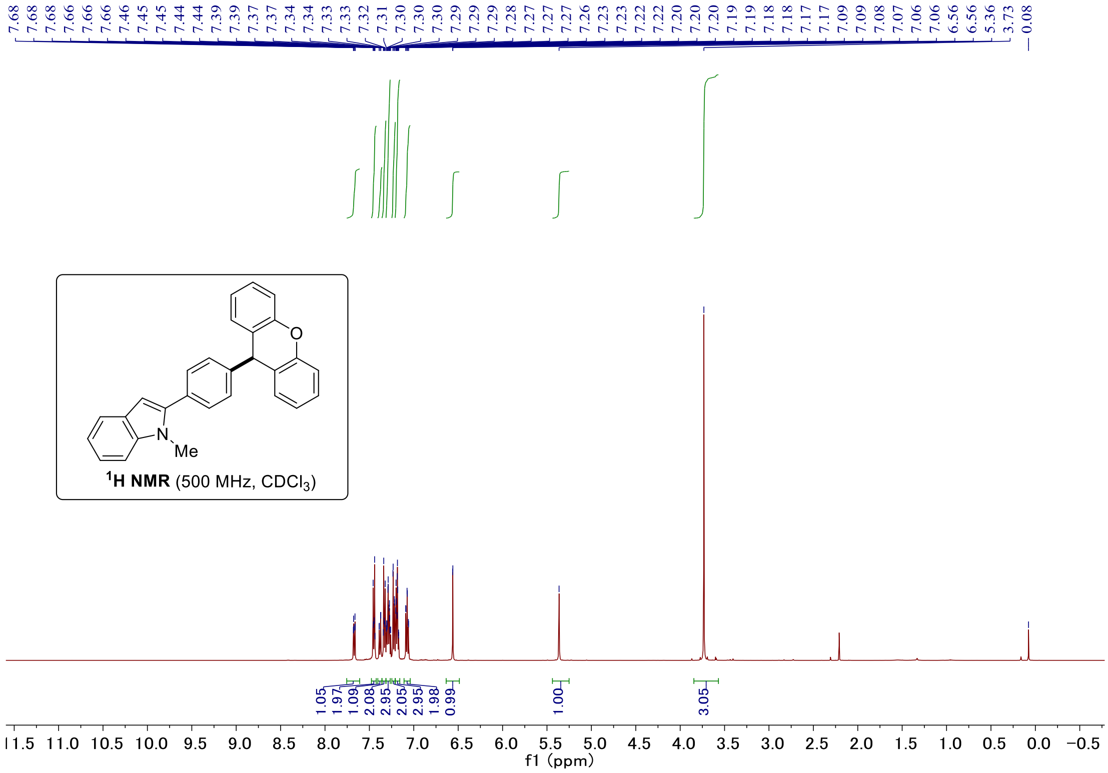

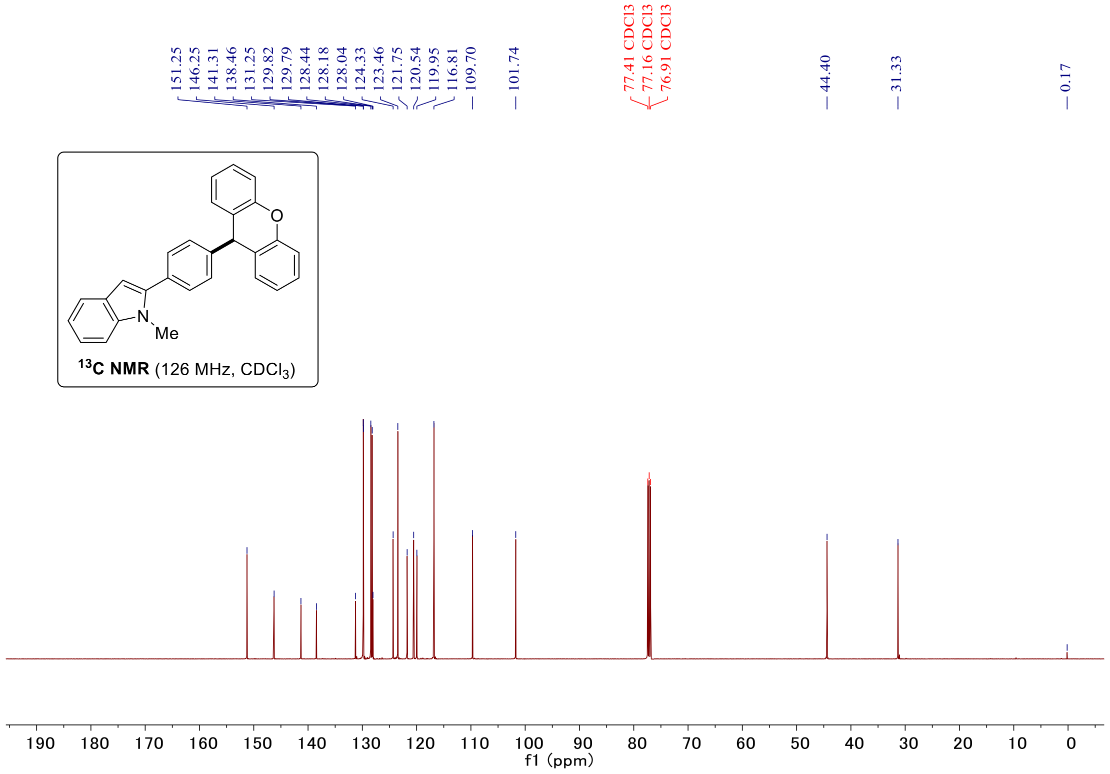

4-(1,1-Diphenylethyl)-biphenyl (3aj)

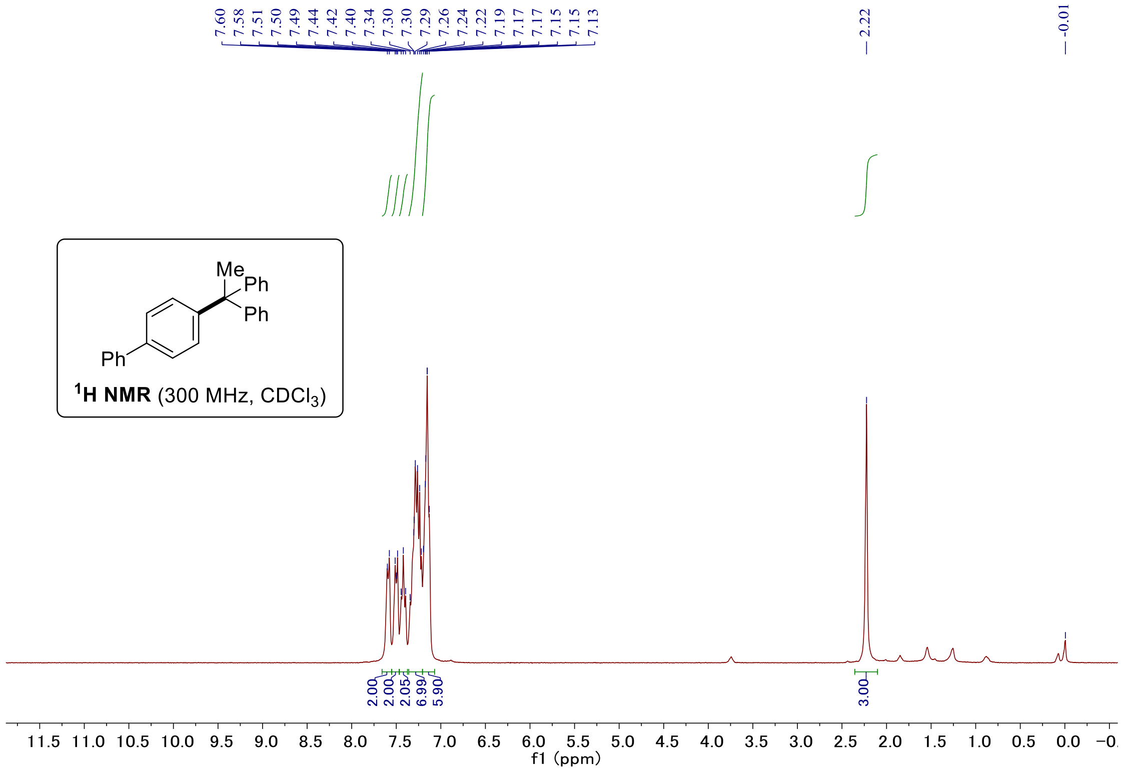

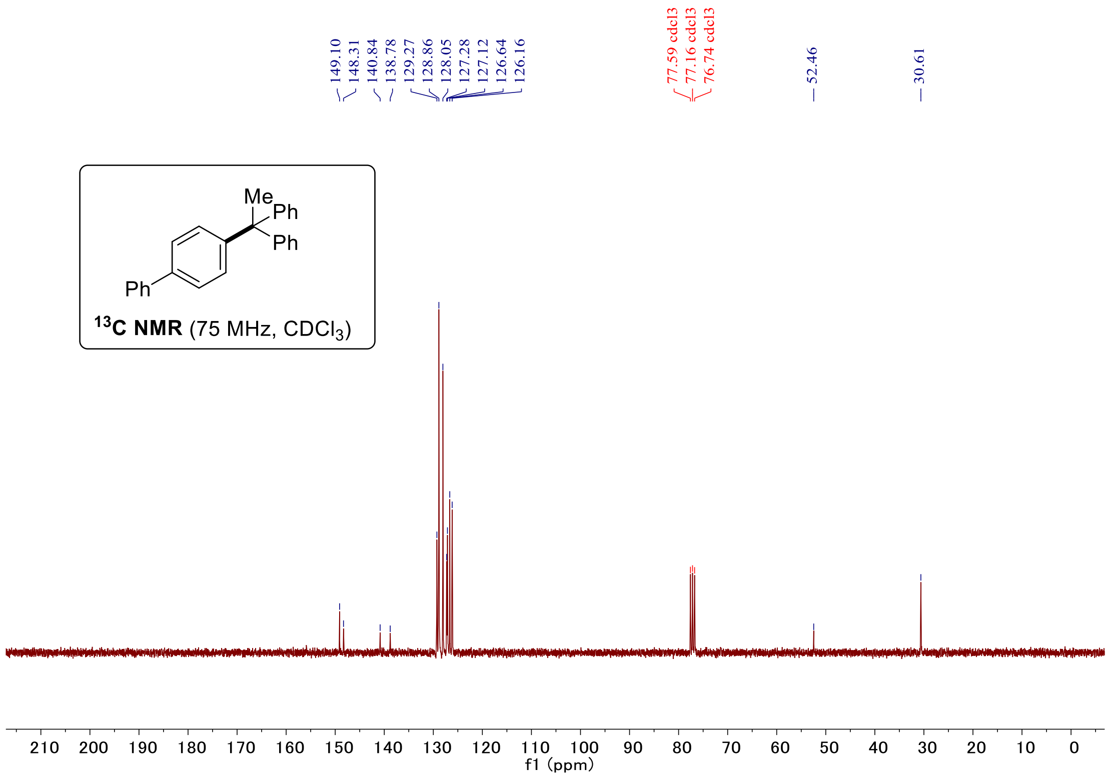

4-(1,1-Diphenylpropyl)-biphenyl (3ak)

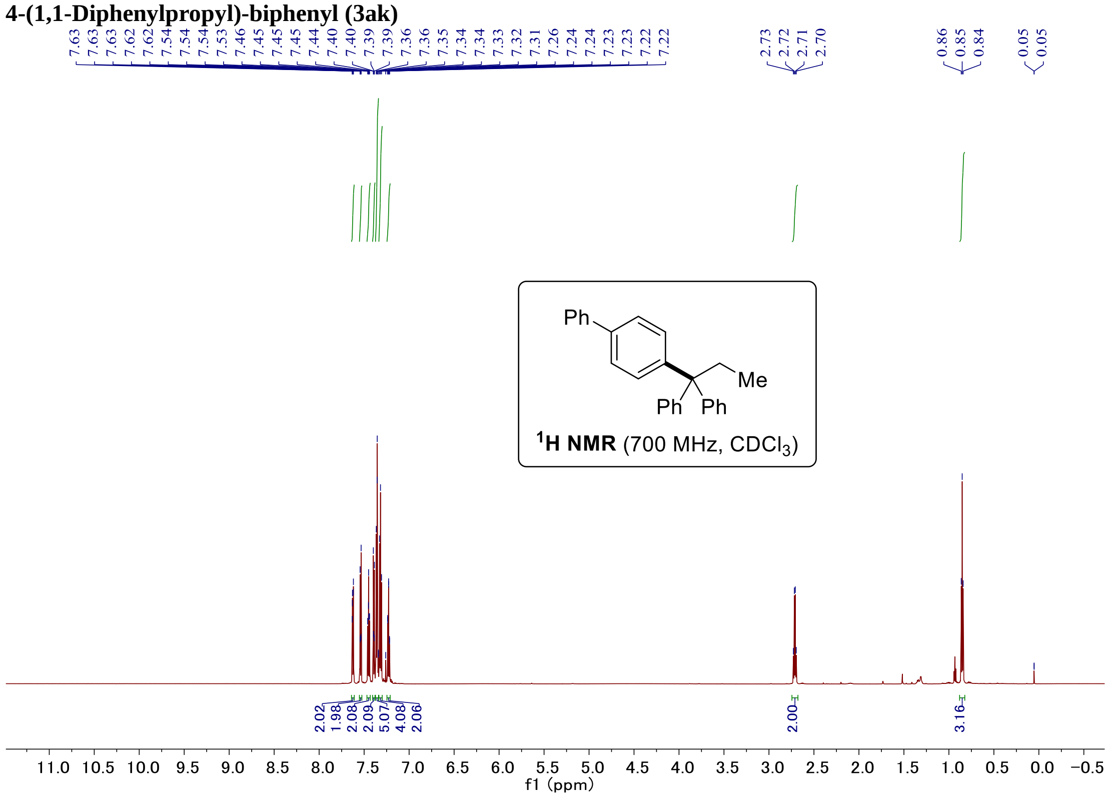

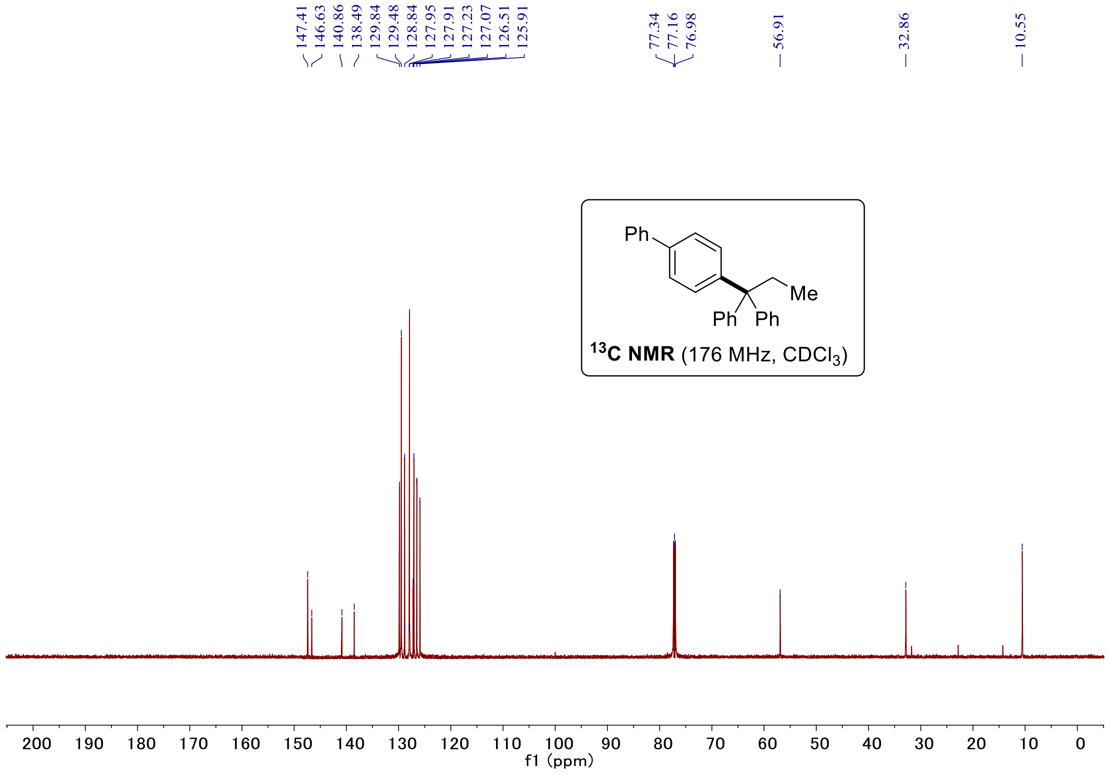

4-(1,1-Diphenylpentyl)-biphenyl (3al)

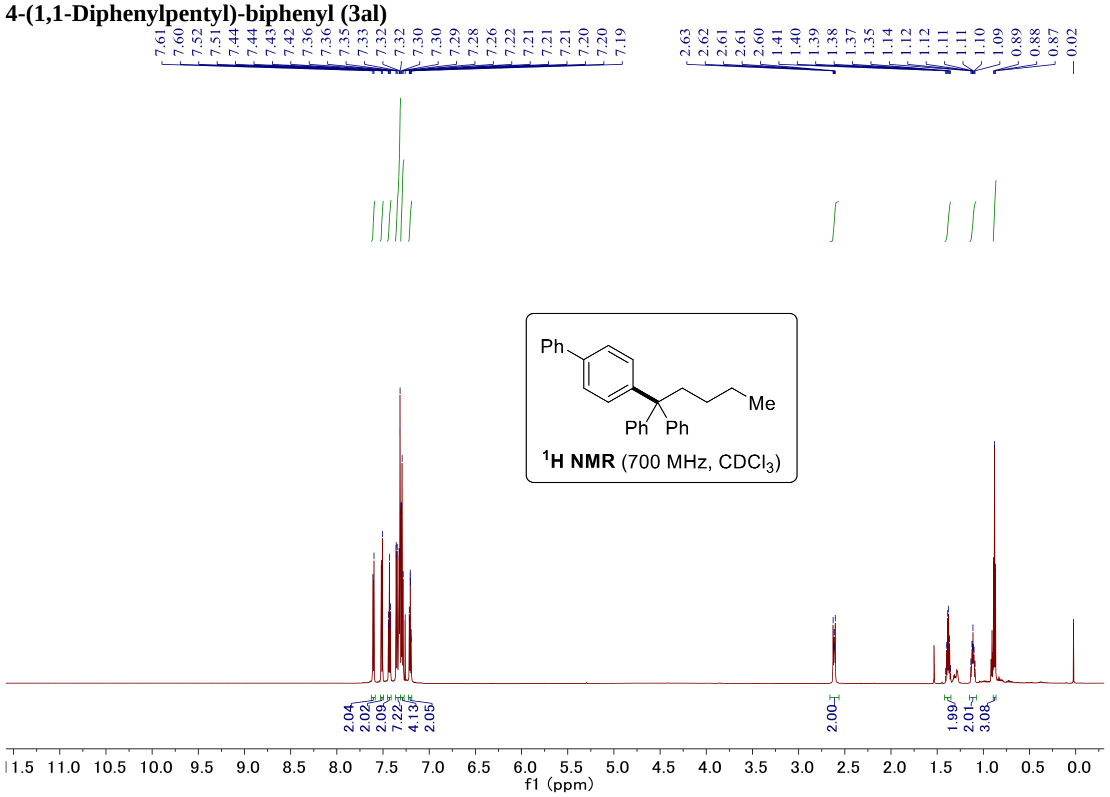

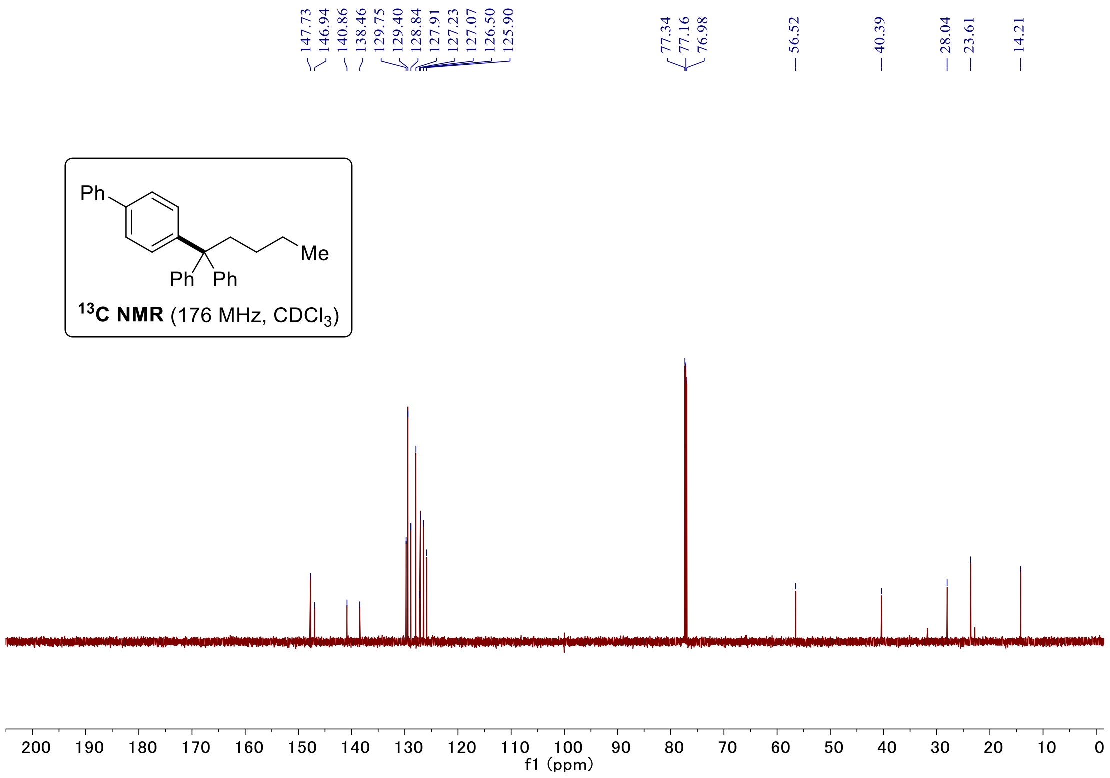

4-(2-Phenylpropan-2-yl)biphenyl (3am)

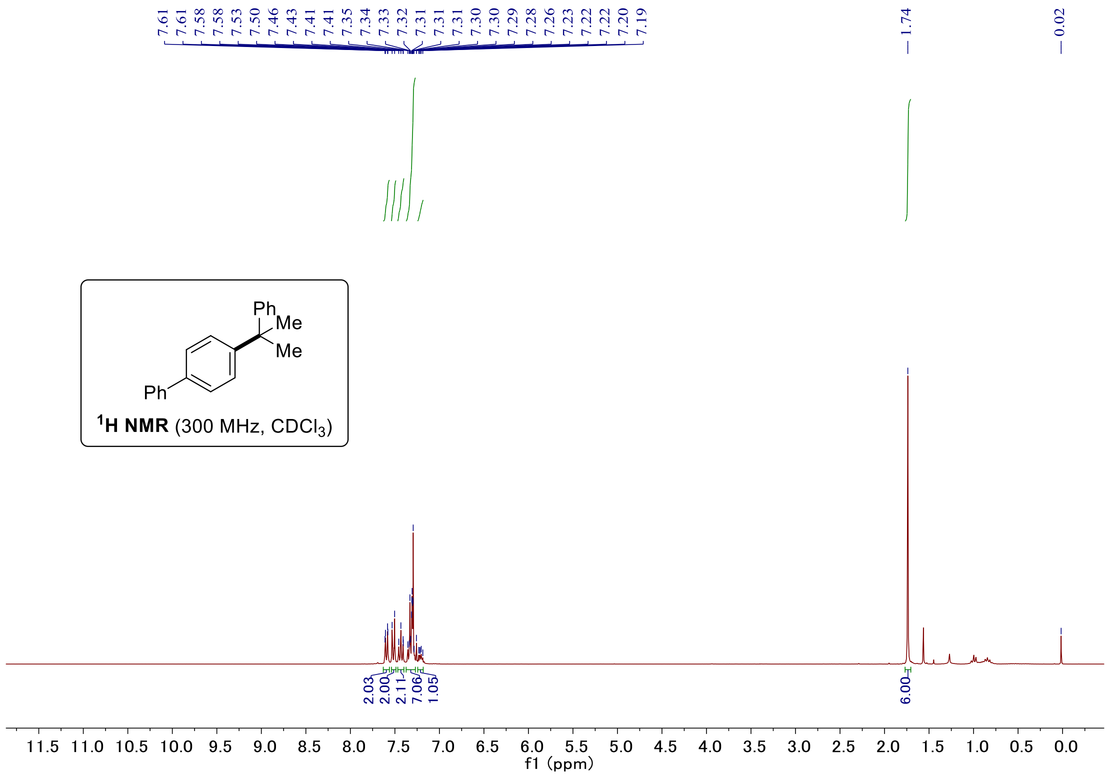

4-(1-Phenylethyl)-biphenyl (3an)

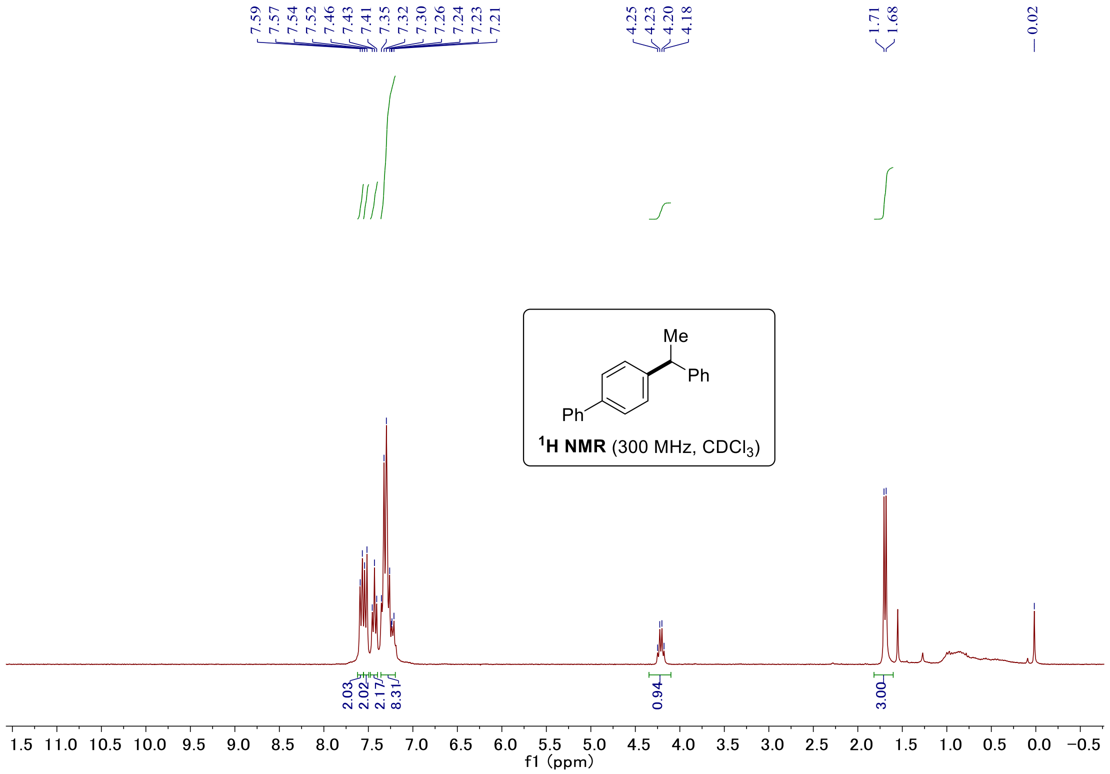

4-(1-Phenylbutyl)biphenyl (3ao)

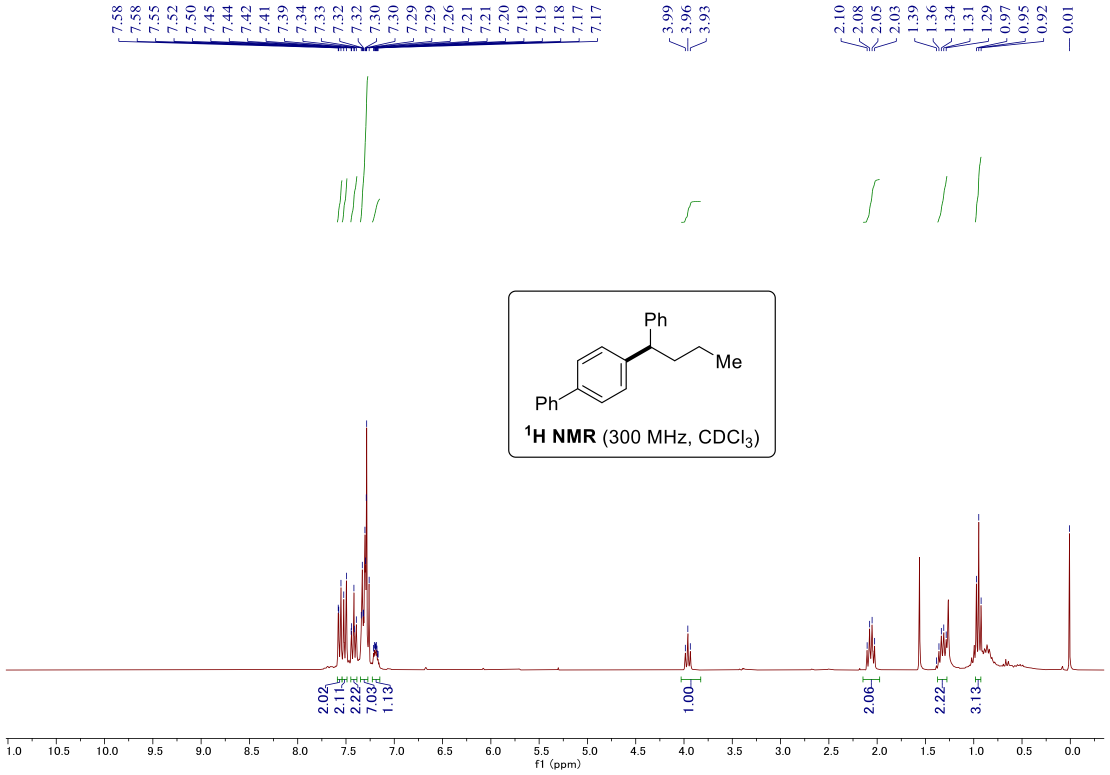

4-(1-Phenylbut-3-en-1-yl)biphenyl (3ap)

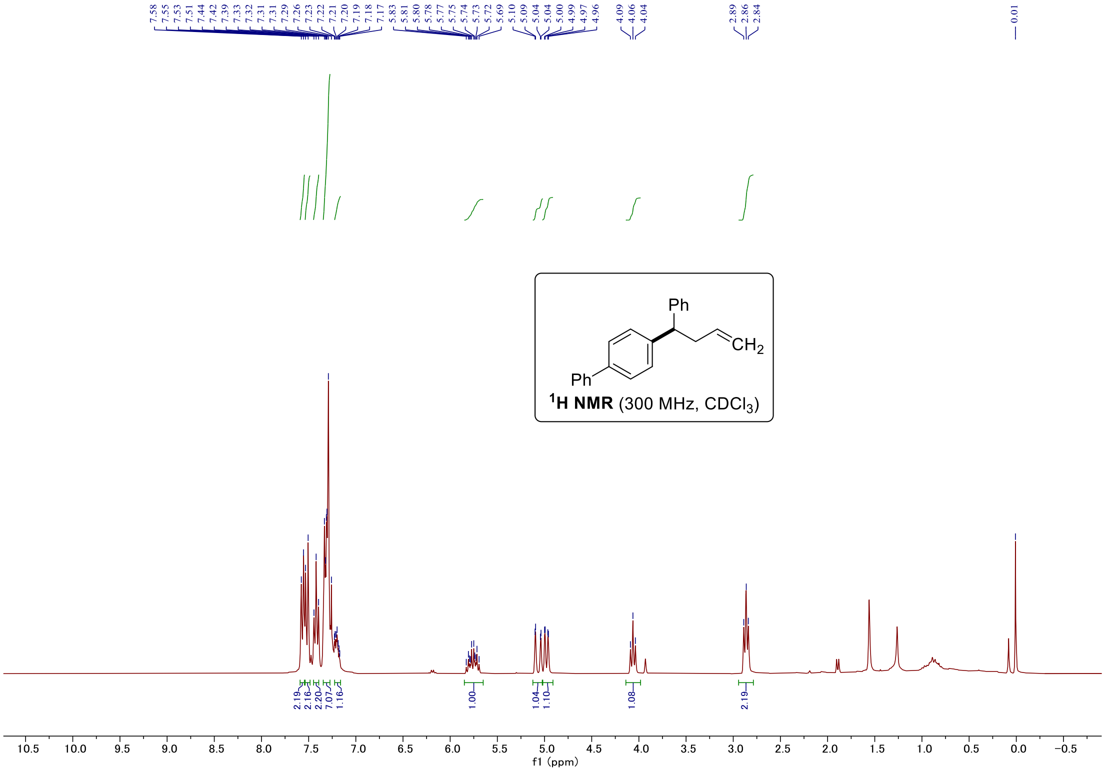

1-(4′-Biphenylyl)-1-phenylpropene (3aq)

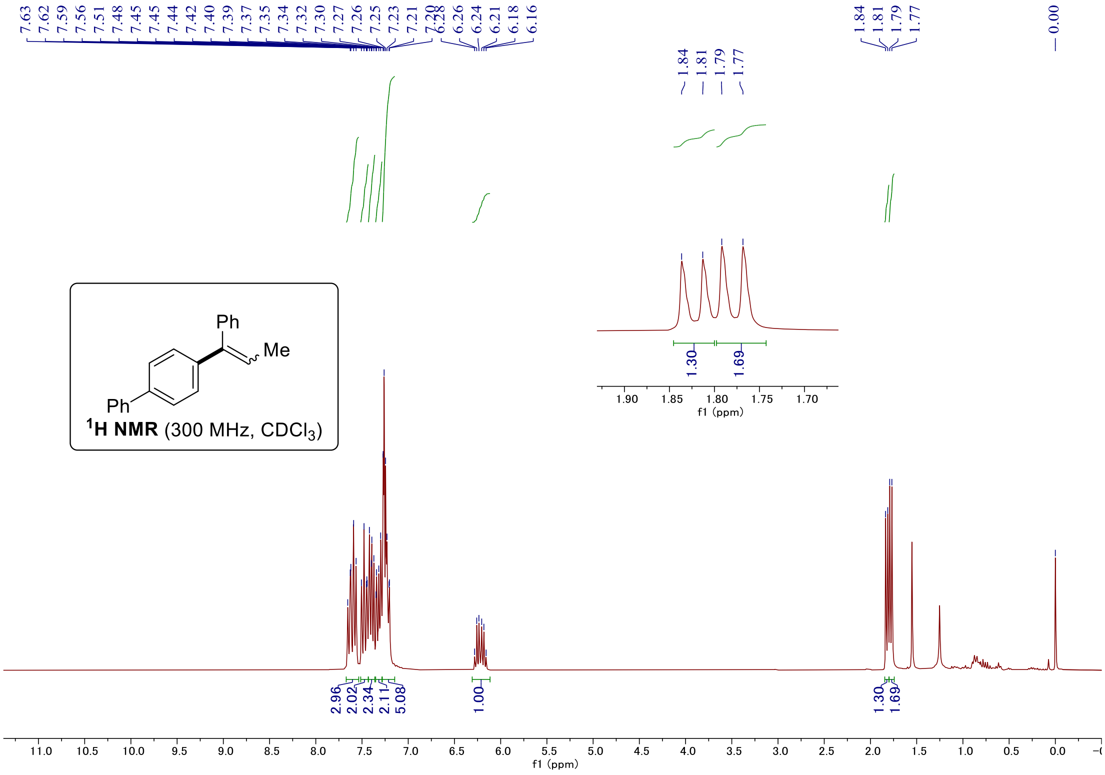

Di(biphenyl-4-yl)methane (3ar)

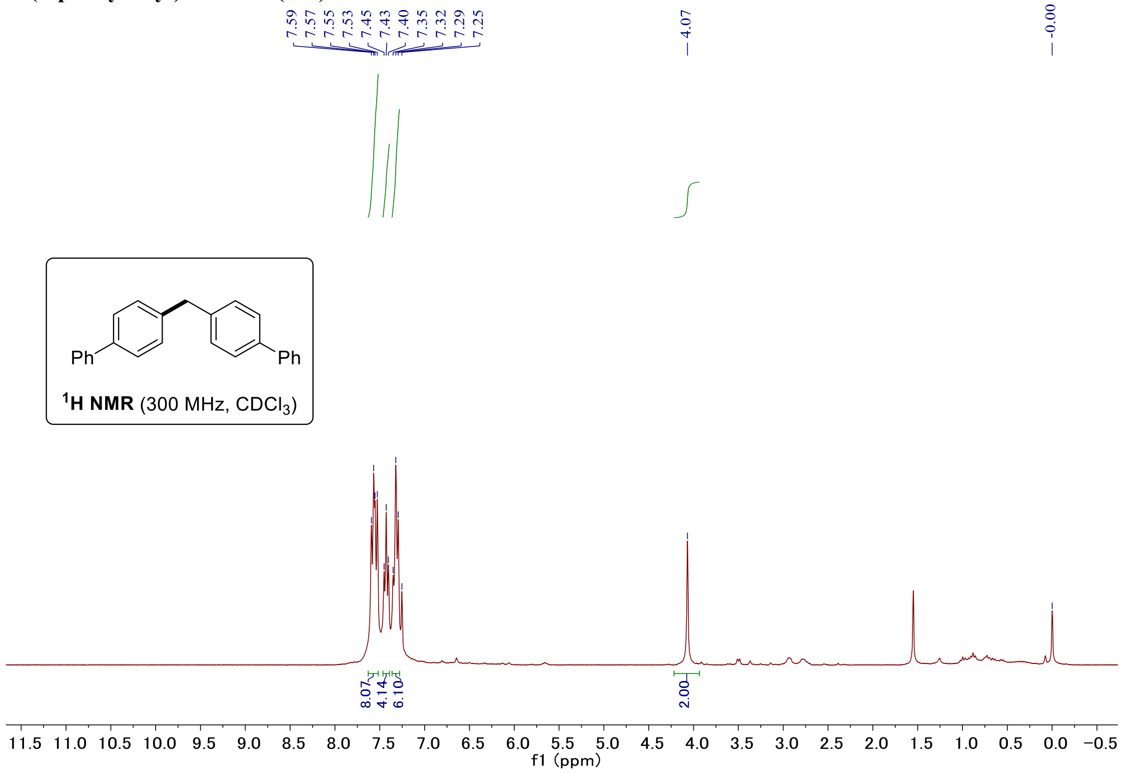

(8*R*,9*S*,13*S*,14*S*)-3-Benzhydryl-17-methoxy-13-methyl-7,8,9,11,12,13,14,15,16,17-decahydro-6*H*-cyclopenta[*a*]phenanthrene (3ra)

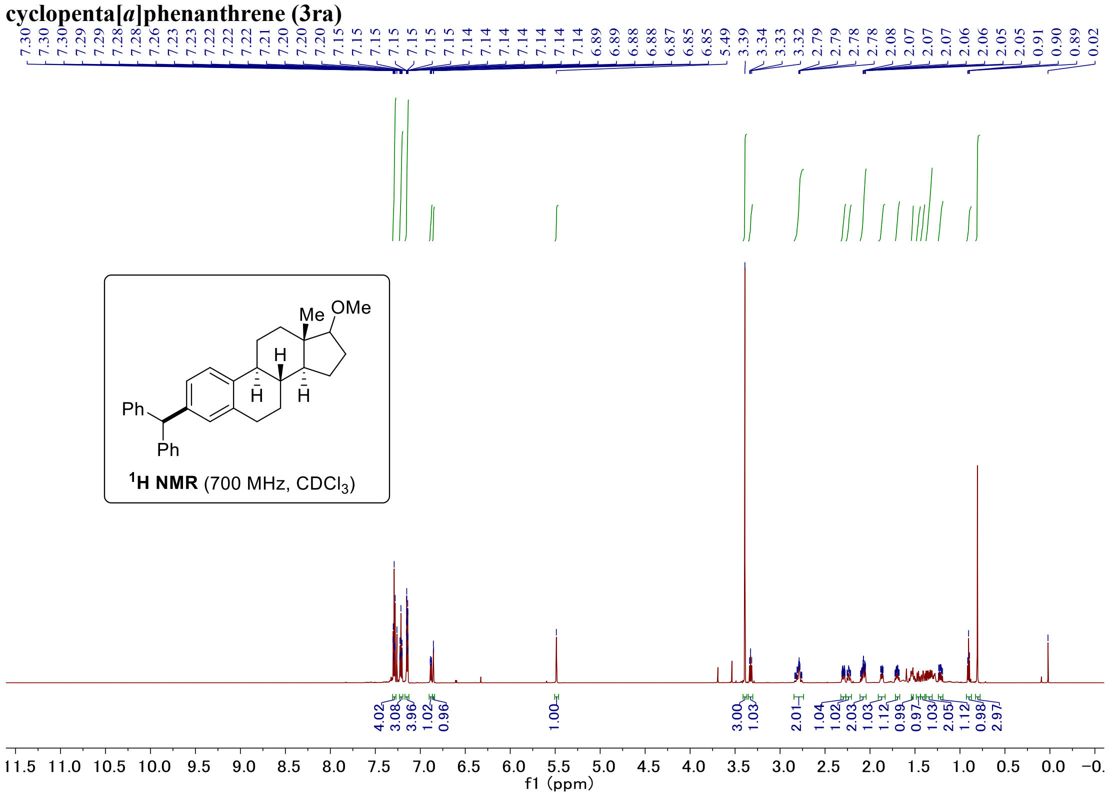

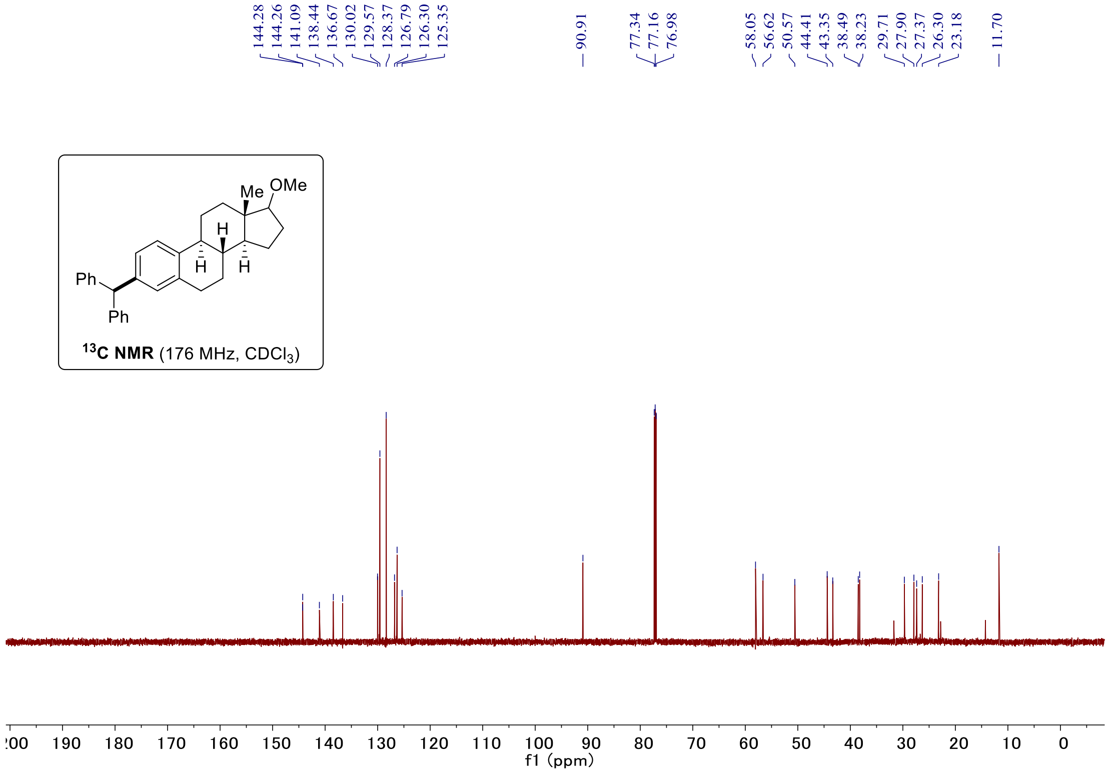

(6*S*,8*R*,9*S*,13*S*,14*S*)-6-([1,1'-Biphenyl]-4-yl)-3,17-dimethoxy-13-methyl-7,8,9,11,12,13,14,15,16,17-decahydro-6*H*-cyclopenta[*a*]phenanthrene (3as)

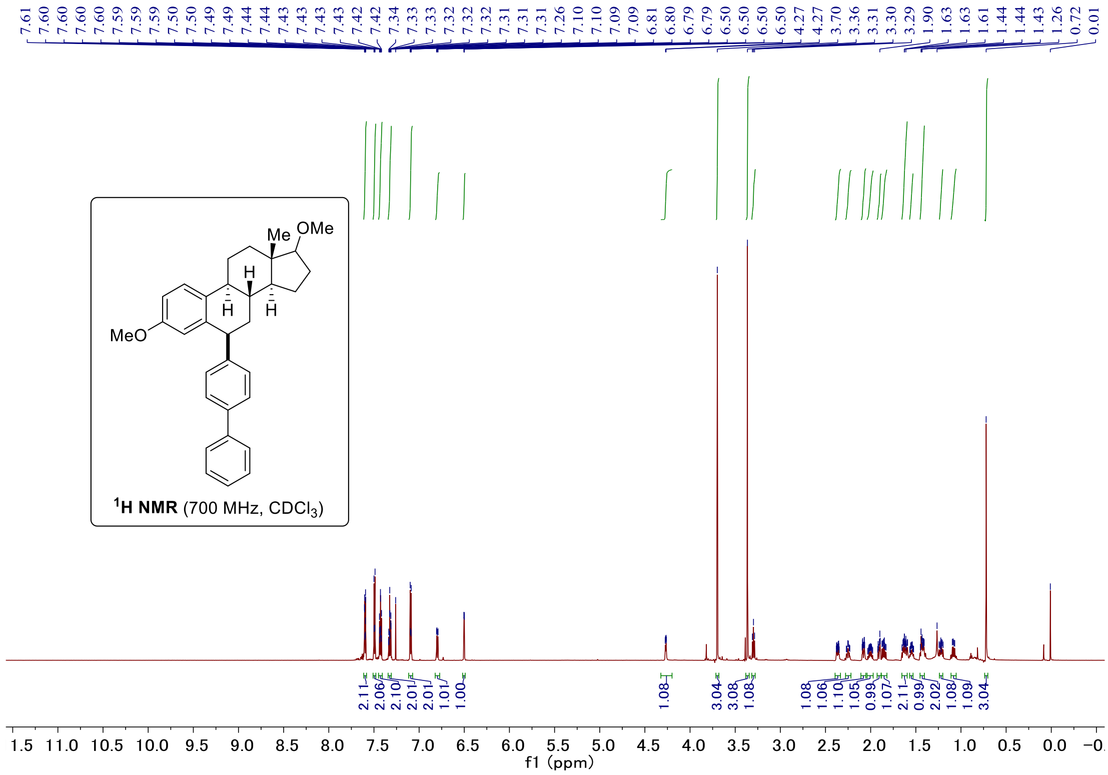

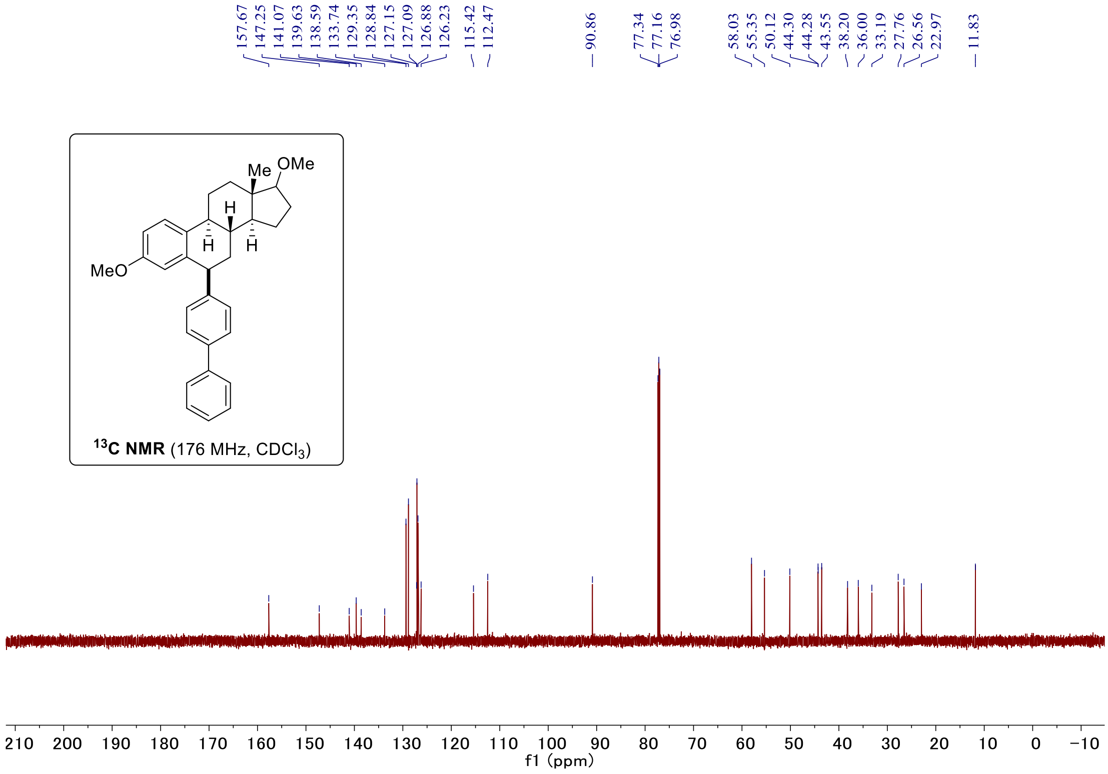

2-(4-((4-Methoxyphenyl)(phenanthren-9-yl)methyl)phenoxy)-*N*,*N*-dimethylethan-1-amine (3st)

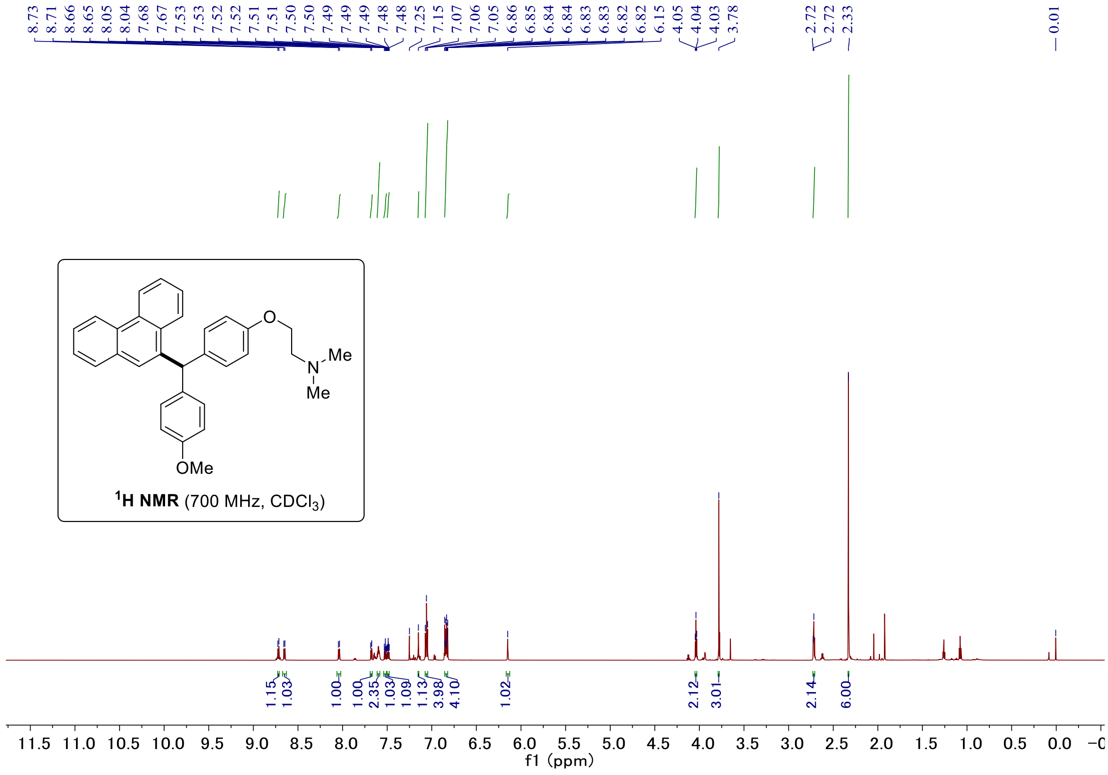

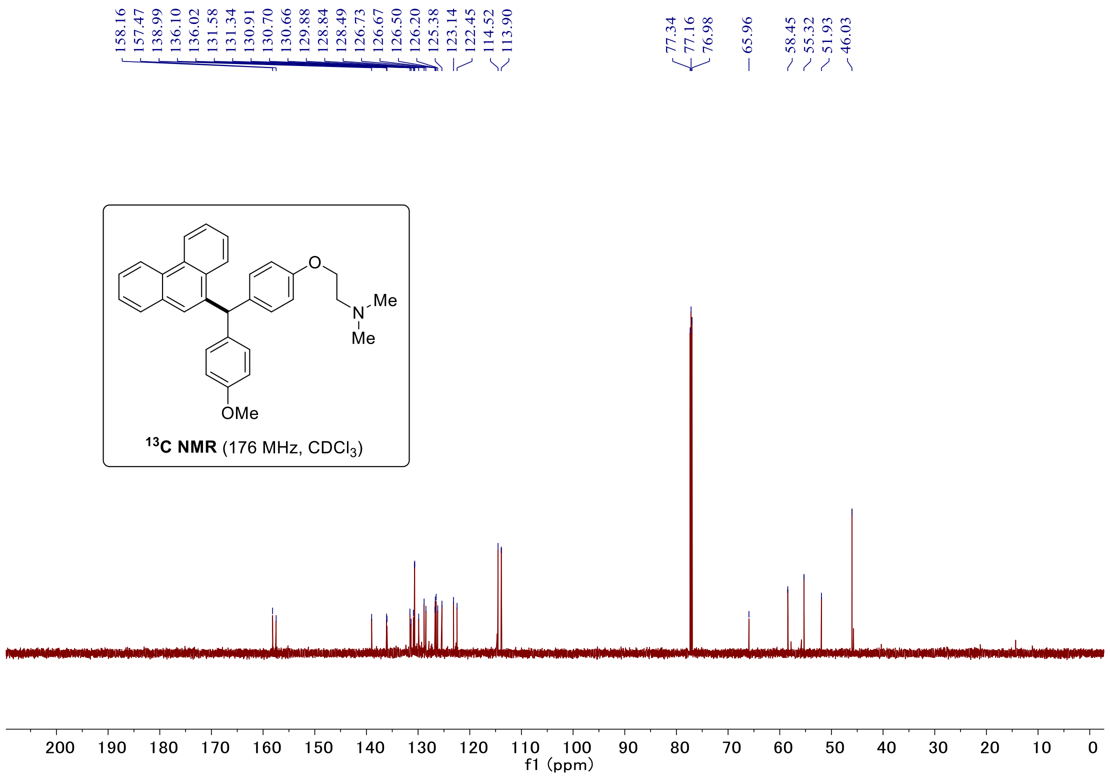

4-Benzhydryl-4'-chlorobiphenyl (3ta)

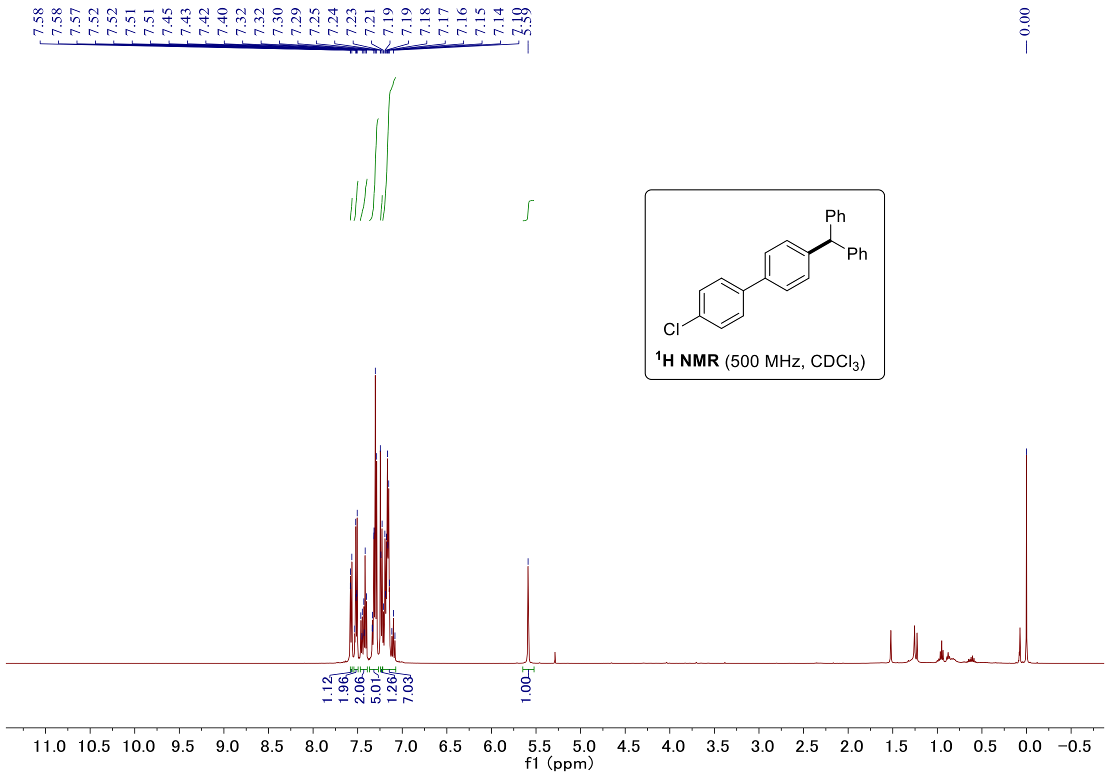

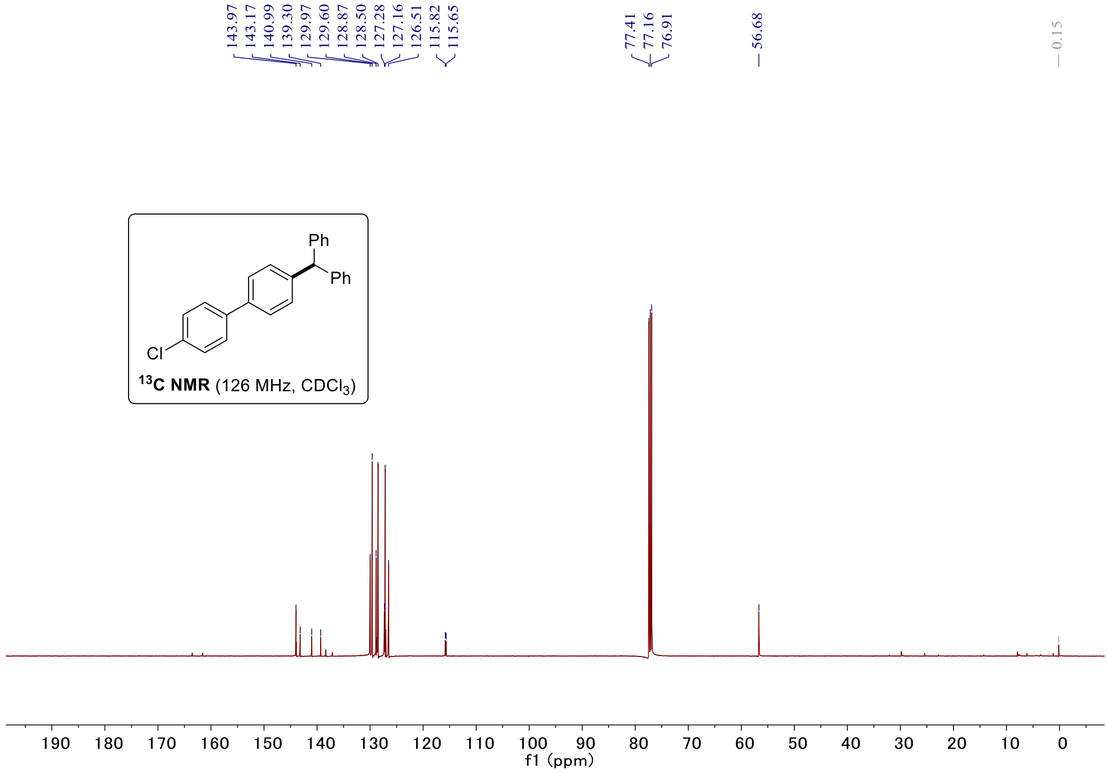

4-Benzhydryl-3-(but-3-en-1-yl)biphenyl (3ua)

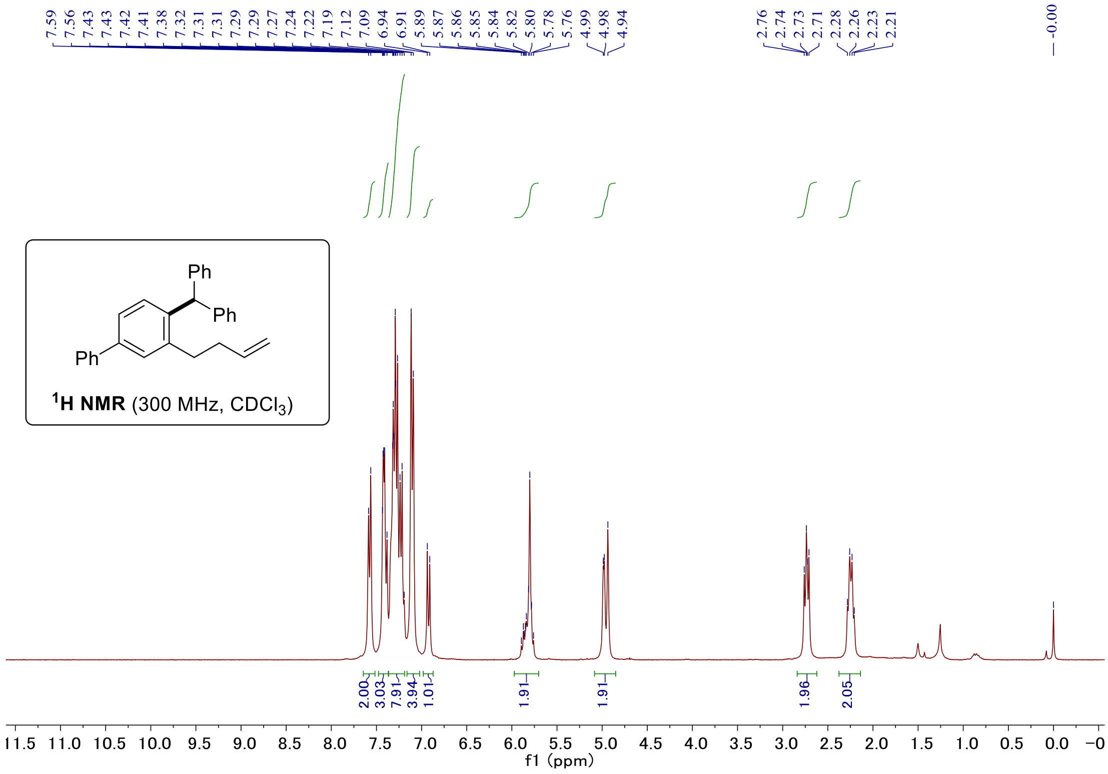

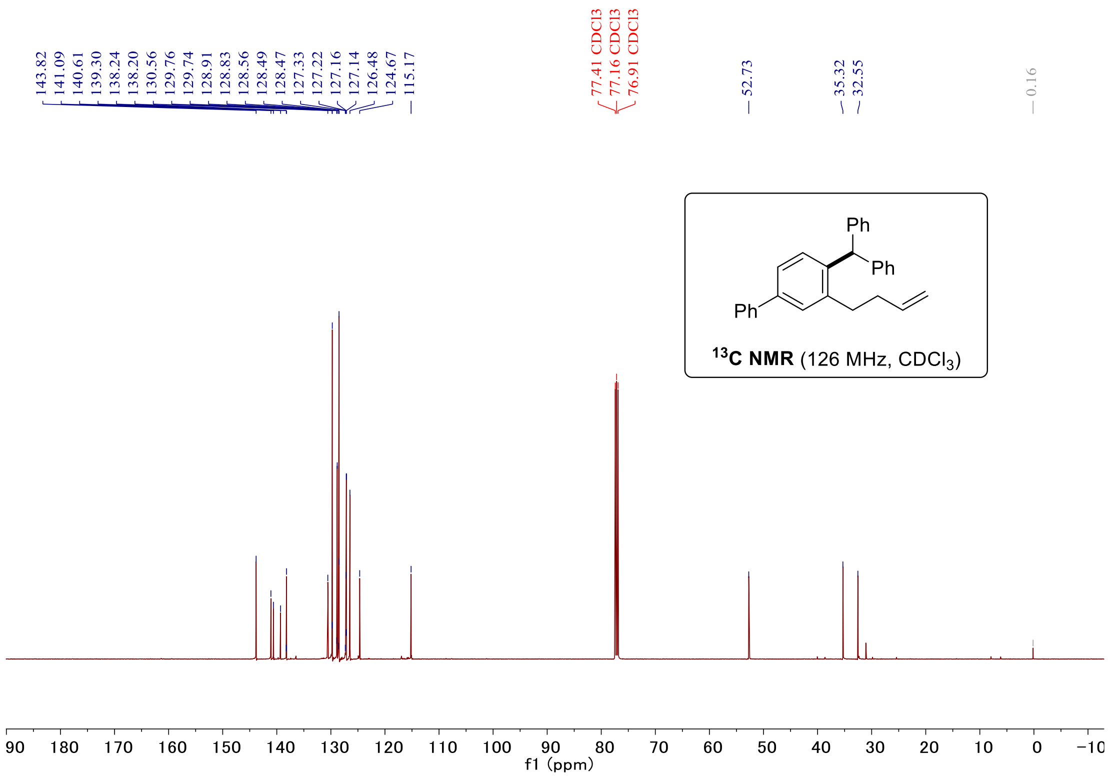

4-(1-Phenylallyl)biphenyl (3aq')

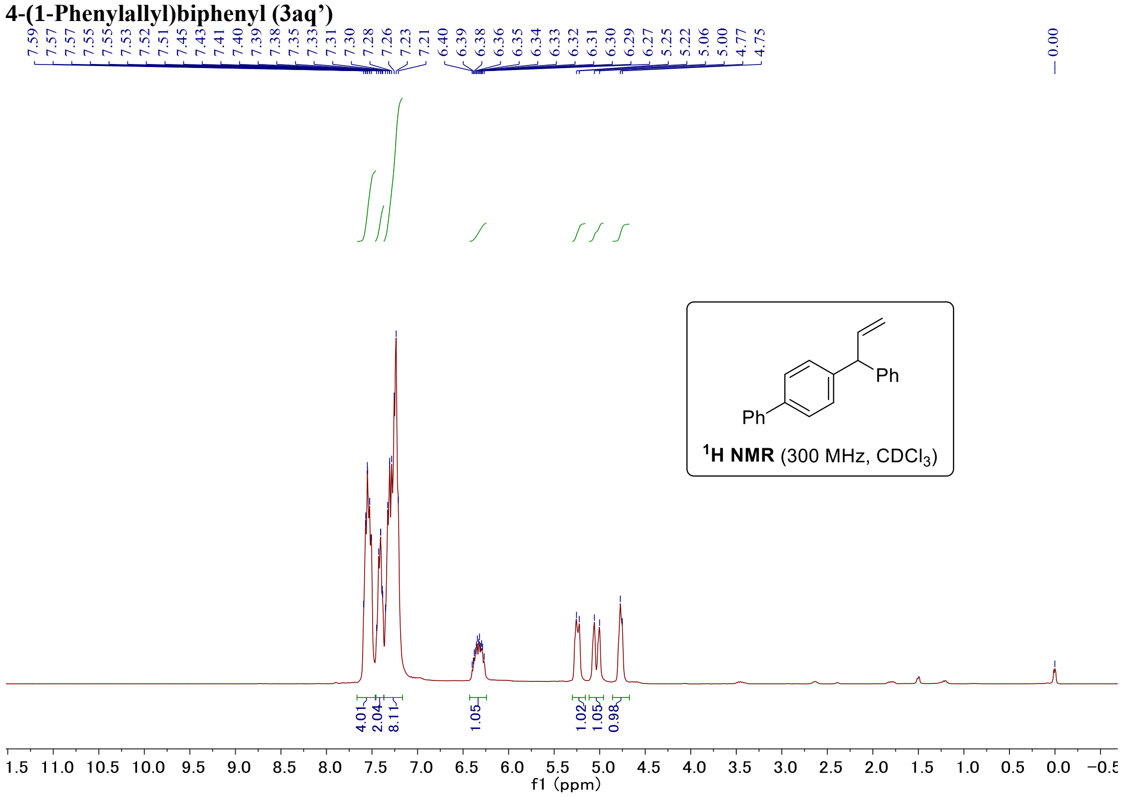

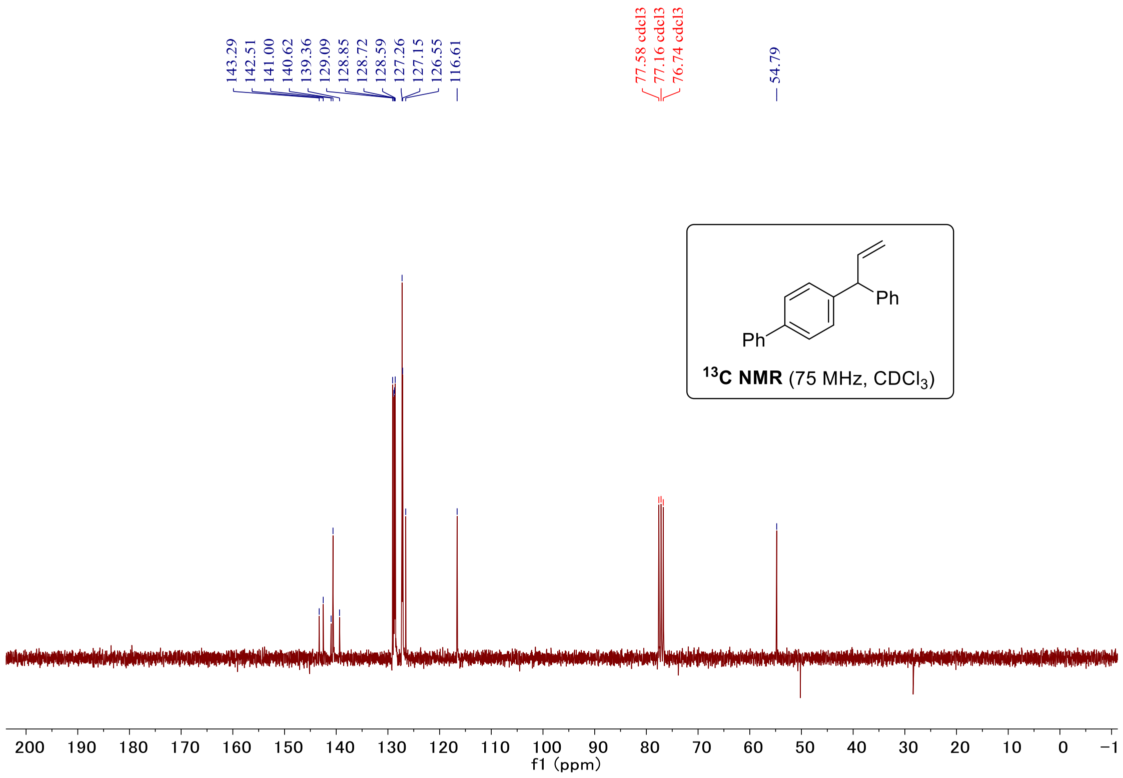

1,1-Diphenyl-2,3-dihydro-1*H*-indene (3v)

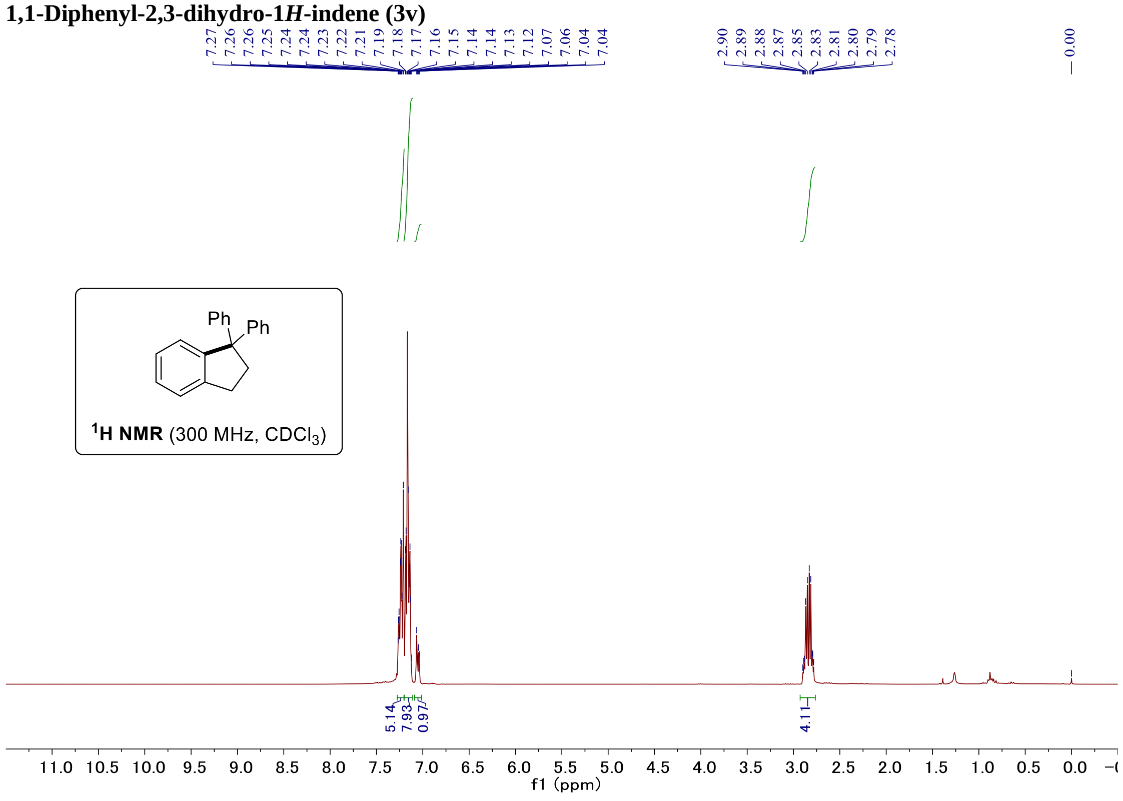

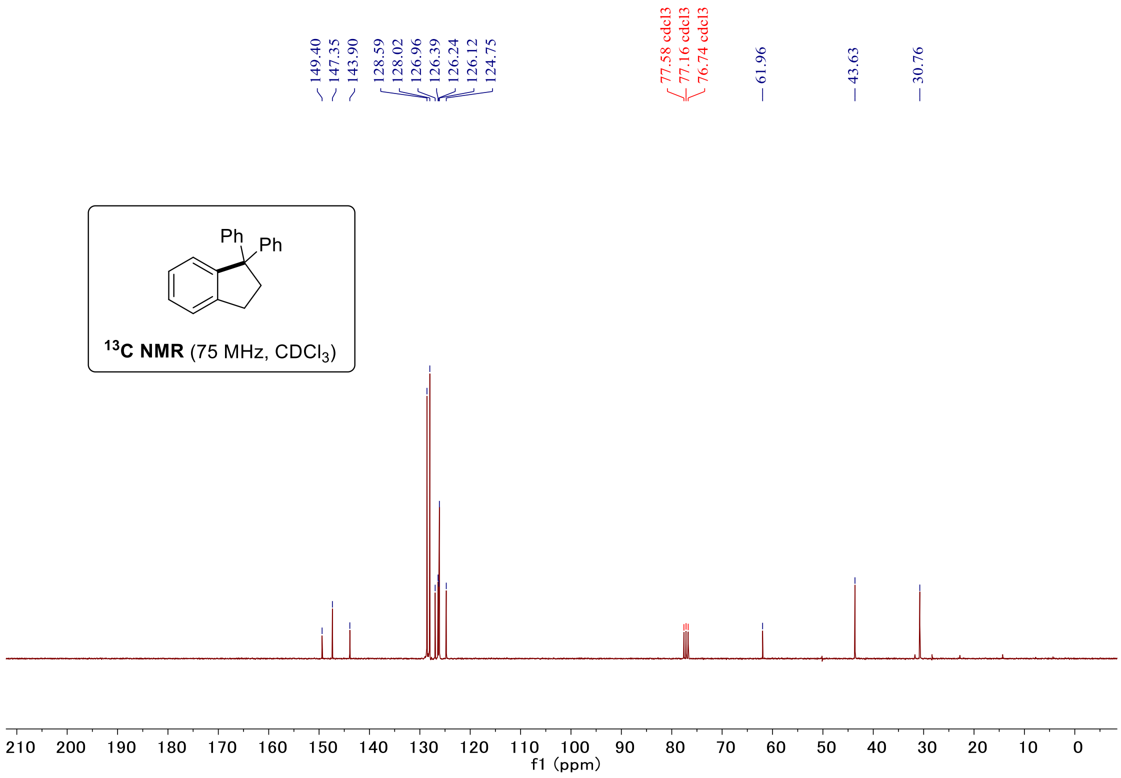

2-(4'-Fluoro-biphenyl-4-yl)-4,4,5,5-tetramethyl-1,3,2-dioxaborolane (5)

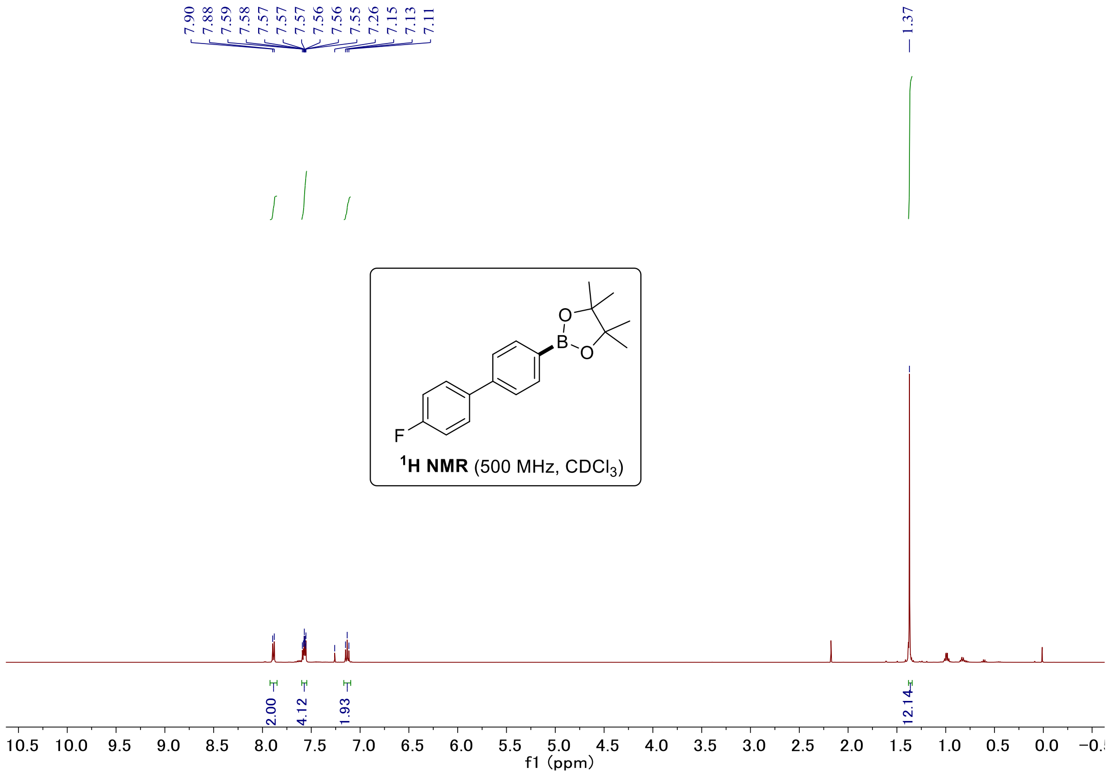

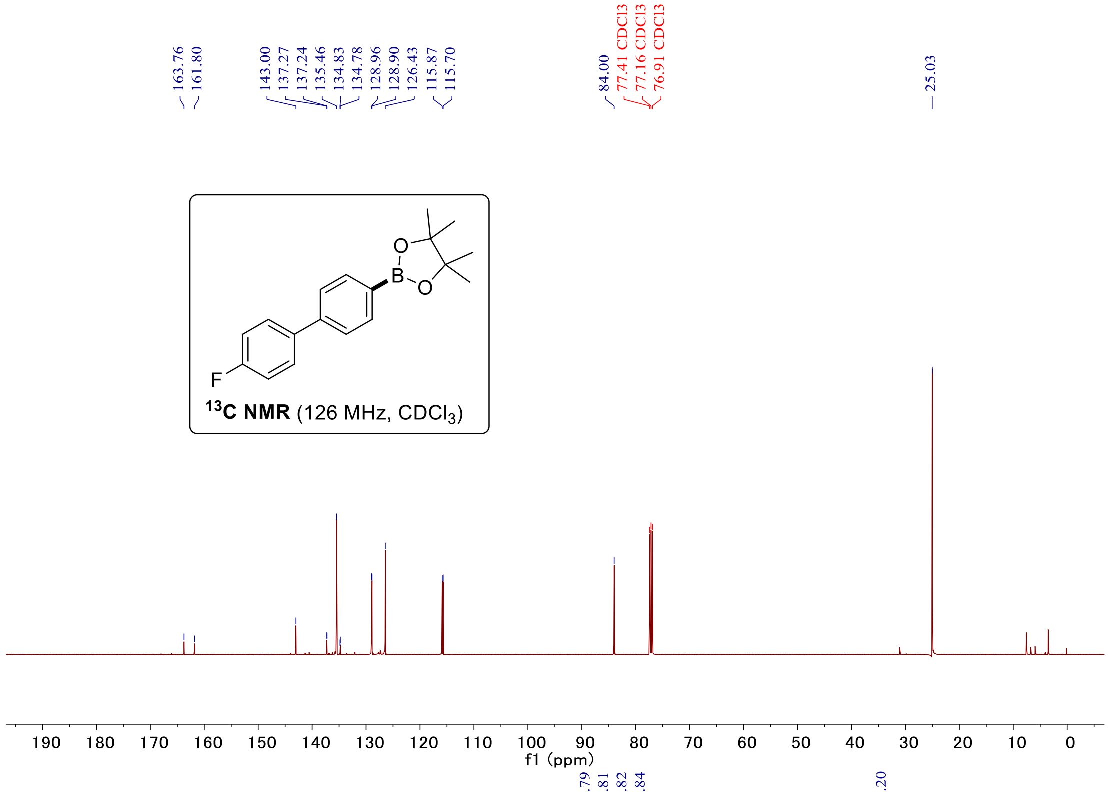
163.76
161.80
143.00
137.27
137.24
135.46
134.83
134.78
128.96
128.90
126.43
115.87
115.70
84.00
77.41 CDCl3
77.16 CDCl3
76.91 CDCl3
25.03
F
O
B
O
^{13}C NMR (126 MHz, CDCl$_3$)
190 180 170 160 150 140 130 120 110 100 90 80 70 60 50 40 30 20 10 0
f1 (ppm)

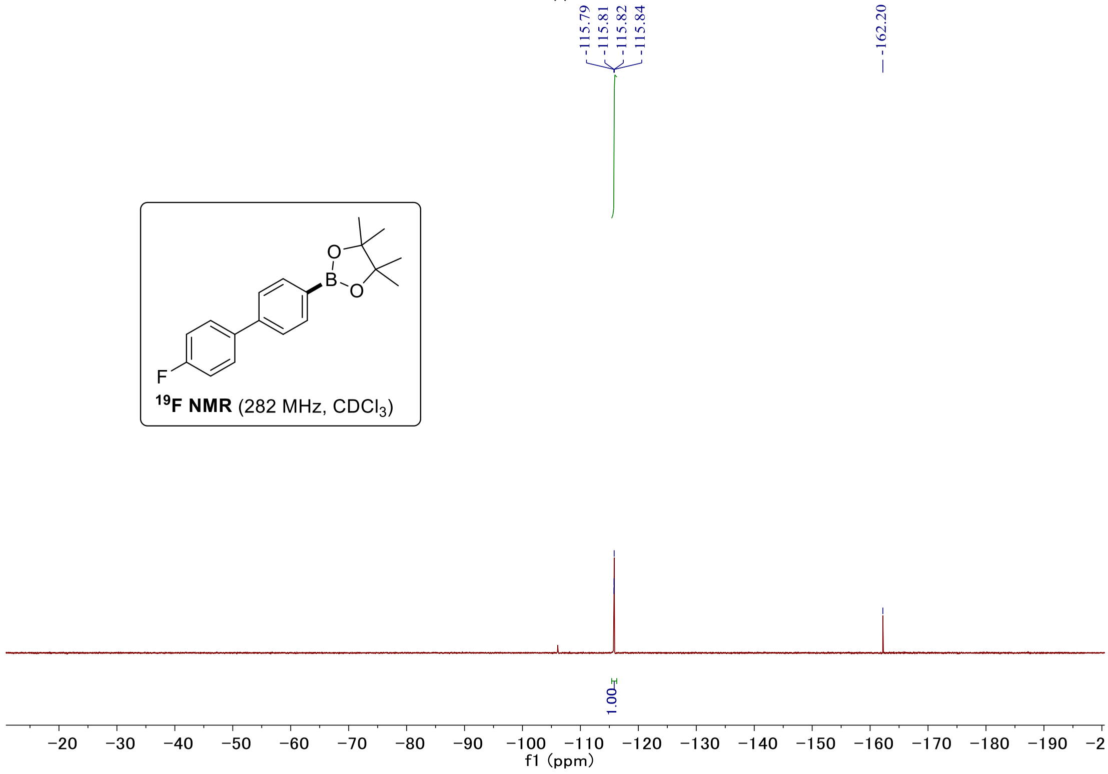
-115.79
-115.81
-115.82
-115.84
-162.20
F
O
B
O
^{19}F NMR (282 MHz, CDCl$_3$)
1.00
−20 −30 −40 −50 −60 −70 −80 −90 −100 −110 −120 −130 −140 −150 −160 −170 −180 −190 −2
f1 (ppm)

2-Benzhydryl-4,4,5,5-tetramethyl-1,3,2-dioxaborolane (10)

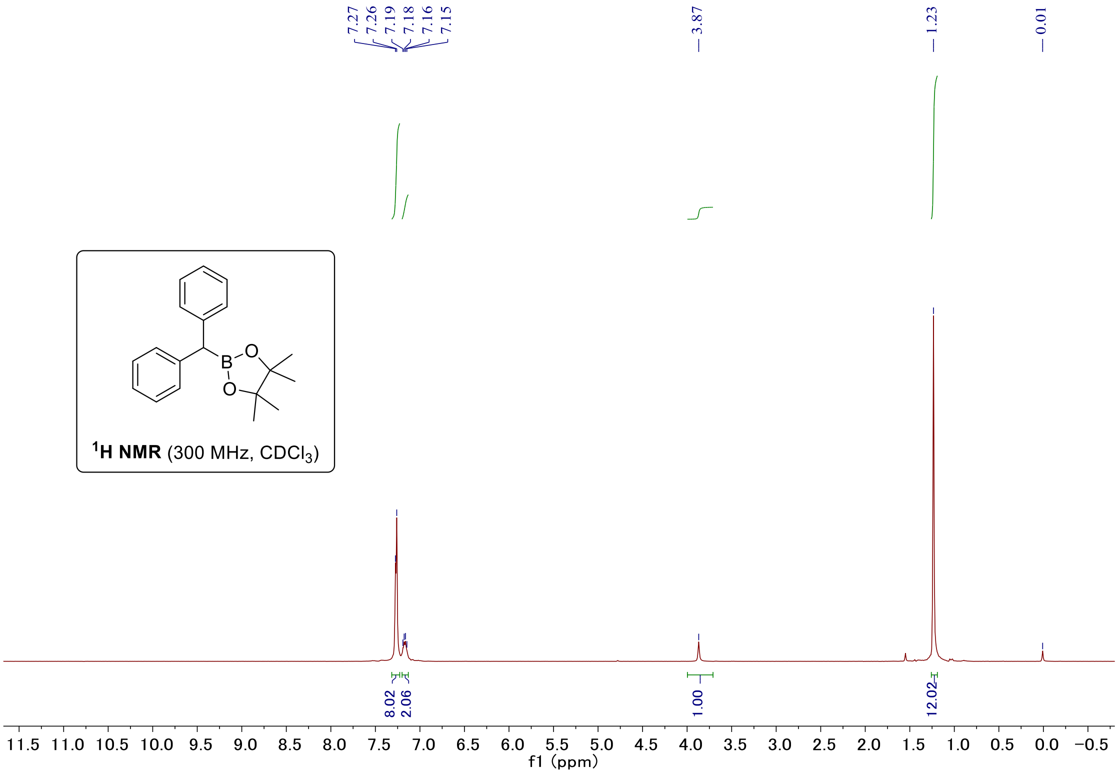

Triethyl((4-methoxyphenyl)(phenyl)methyl)silane (11)

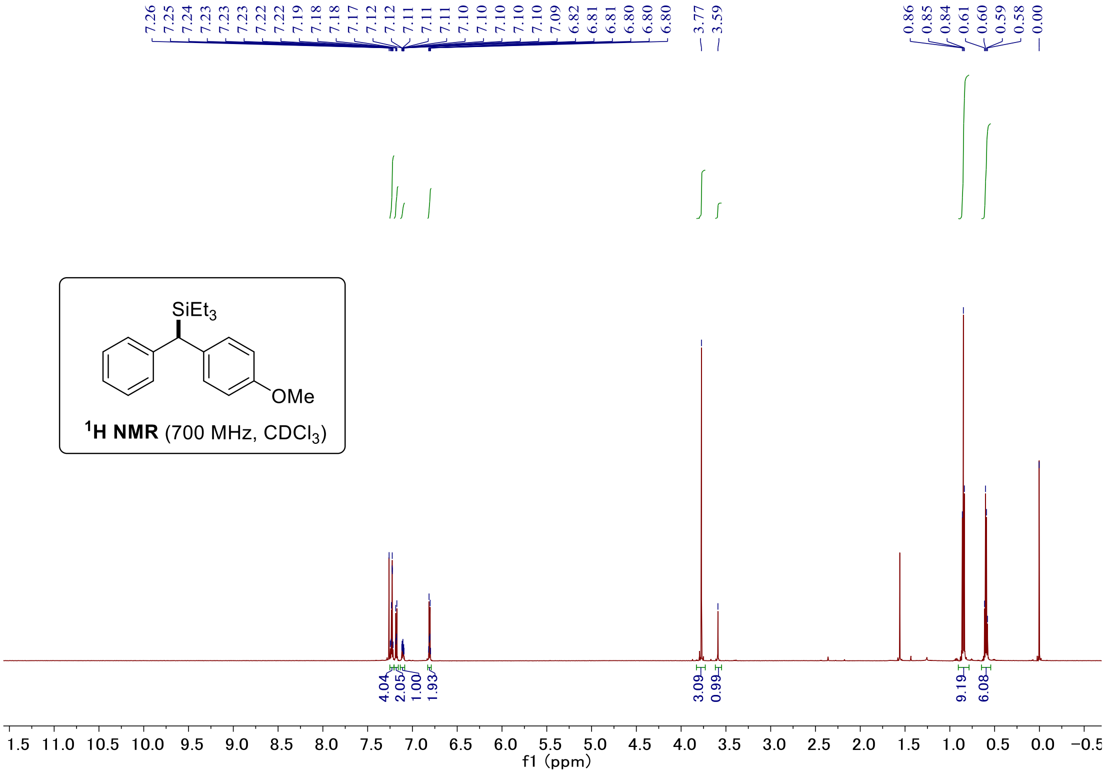

1-(Benzhydryloxy)-2,2,6,6-tetramethylpiperidine (Int-TEMPO)

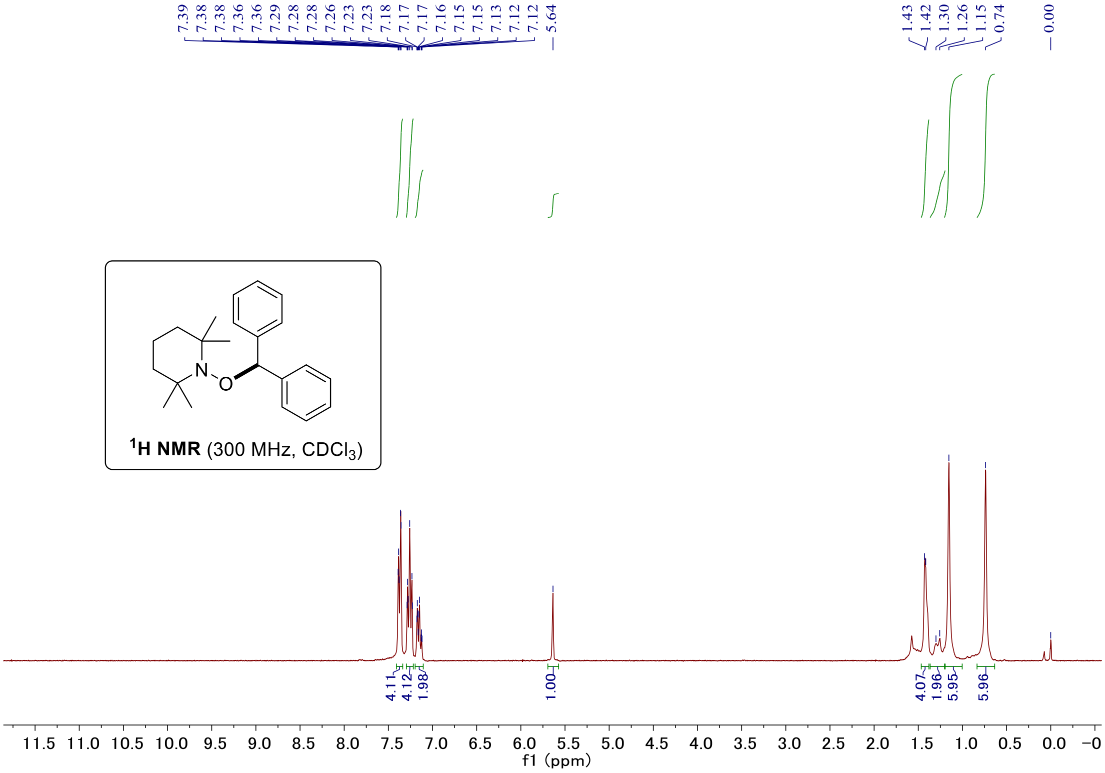

9. References

1. J. Zhou, B. Jiang, Y. Fujihira, Z. Zhao, T. Imai, and N. Shibata, *Nat. Commun.*, 2021, **12**, 3749–3757.
2. N. Ortega, S. Yrban, B. Beiring, and F. Glorius, *Angew. Chem., Int. Ed.*, 2012, **51**, 1710–1713.
3. M. Zupan, J. Iskra, and S. Stavber, *J. Fluorine Chem.,* 1995, **70**, 7–8.
4. T. Niwa, H. Ochiai, and T. Hosoya, *ACS Catal.*, 2017, **7**, 4535–4541.
5. K. Ren, P. H. Li, L. Wang, and X. L. Zhang, *Tetrahedron*, 2011, **67**, 2753–2759.
6. Y. Liu, D. Zhai, X. Zhang, and B. Guan, *Angew. Chem., Int. Ed.*, 2018, **57**, 8245–8249; *Angew. Chem.,* 2018, **130**, 8377–8381
7. A. Ono, N. Suzuki, and J. Kamimura, *Synthesis*, 1987, **8**, 736–738.
8. I. Abdiaj, A. Fontana, M. V. Gomez, A. de la Hoz, and J. Alcázar, *Angew. Chem., Int. Ed.*, 2018, **57**, 8473–8477; *Angew. Chem.*, 2018, **130**, 8609–8613.
9. K. Endo, T. Ishioka, T. Ohkubo, and T. Shibata, *J. Org. Chem.*, 2012, **77**, 7223–7231.
10. F. Chahdoura, C. Pradel, and M. Gómez, *Adv. Synth. Catal.*, 2013, **355**, 3648–3660.
11. E. Alacid, and C. Nájera, *Org. Lett.*, 2008, **10**, 5011–5014.
12. M. J. Burns, I. J. S. Fairlamb, A. R. Kapdi, P. Sehnal, and R. J. K. Tayor, *Org. Lett.*, *2007,* **9**, 5397–5400.
13. T. J. A. Corrie, L. T. Ball, C. A. Russell, and G. C. Lloyd-Jones, *J. Am. Chem. Soc.*, 2017, **139**, 245–254.
14. W. Xu, P. Zheng, and T. Xu, *Org. Lett.*, 2020, **22**, 8643–8647.
15. M. L. Czyz, M. S. Tayor, T. H. Horgren, and A. Polyzos, *ACS Catal.*, 2021, **11**, 5472–5480.
16. Z. Jia, Q. Liu, X. Peng, and H. N. C. Wong, *Nat. Commun*., 2016, **7**, 10614–10621.
17. B. Wang, L. Qin, K. D. Neumann, S. Uppaluri, R. L. Cerny, and S. G. Dimagno, *Org. Lett.,* 2010, **12**, 3352–3355.
18. G. Panda, M. K. Parai, S. K. Das, Shagufta, M. Sinha, V. Chaturvedi, A. K. Srivastava, Y. K. Manju, A. Gaikwad, and S. Sinha, *Eur. J. Med. Chem.,* 2007, **42**, 410–419.
19. P. C. Too, G. H. Chan, Y. L. Tnay, H. Hirao and S. Chiba, *Angew. Chem., Int. Ed.*, 2016, **55**, 3719–3723; *Angew. Chem.*, 2016, **128**, 3783–3787.
20. N. Hussain, G. Frensch, J. Zhang and P. J. Walsh, *Angew. Chem., Int. Ed.*, 2014, **53**, 3693–3697.
21. J. W. Clary, T. J. Rettenmaier, R. Snelling, W. Bryks, J. Banwell, W. T. Wipke and B. Singaram, *J. Org. Chem.*, 2011, **76**, 9602–9610.
22. S. Roesner, C. A. Brown, M. Mohiti, A. P. Pulis, R. Rasappan, D. J. Blair, S. Essafi, D. Leonori and V. K. Aggarwal, *Chem. Commun.*, 2014, **50**, 4053–4055.
23. Z. Zhang, H. Wang, N. Qiu, Y. Kong, W. Zeng, Y. Zhang, and J. Zhao, *J. Org. Chem.,* 2018, **83**, 8710–8715.
24. J. Yu, and R. Kuwano, *Org. Lett.*, 2008, **10**, 973–976.
25. J. Zhang, A. Bellomo, N. Trongsiriwat, T. Jia, P. J. Carroll, S. D. Dreher, M. T. Tudge, H. Yin, J. R. Robinson, E. J. Schelter, and P. J. Walsh, *J. Am. Chem. Soc.*, 2014, **136**, 6276–6287.
26. S. Podder, and S. Roy, *Tetrahedron,* 2007, **63**, 9146–9152.
27. G. Pallikonda, and M. Chakravarty, *J. Org. Chem.*, 2016, **81**, 2135–2142.
28. J. Zhang, A. Bellomo, A. D. Creamer, S. D. Dreher, and P. J. Walsh, *J. Am. Chem. Soc.*, 2012, **134**, 13765–13772.
29. M. R. Harris, L. E. Hanna, M. A. Greene, C. E. Moore, and E. R. Jarvo, *J. Am. Chem. Soc.*, 2013, **135**, 3303–3306.
30. S. Yang, W. Tang, Z. Yang, and J. Xu, *ACS Catal.*, 2018, **8**, 9320–9326.
31. K. B. Urkalan, and M. S. Sigman, *Angew. Chem., Int. Ed.*, 2009, **48**, 3146–3149.
32. Y. He, Y. Cai, and S. Zhu, *J. Am. Chem. Soc.*, 2017, **139**, 1061–1064.
33. E. Grovenstein, J. A. Beres, Y. Cheng, and J. A. Pegolotti, *J. Org. Chem.*, 1972, **37**, 1281–1292.
34. Y. He, C. Liu, L. Yu, and S. Zhu, *Angew. Chem., Int. Ed.*, 2020, **59**, 21530–21534; *Angew. Chem.*, 2020, **132**, 21714–21718.
35. S. H. Shin, D. Cizmeciyan, A. E. Keating, S. I. Khan and M. A. Garcia-Garibay, *J. Am. Chem. Soc.*, 1997, **119**, 1859–1868.
36. P. C. Too, G. H. Chan, Y. L. Tnay, H. Hirao and S. Chiba, *Angew. Chem., Int. Ed.*, 2016, **55**, 3719–3723; *Angew. Chem.*, 2016, **128**, 3783–3787.
37. H. Ochiai, Y. Uetake, T. Niwa and T. Hosoya, *Angew. Chem., Int. Ed.*, 2017, **56**, 2482–2486; *Angew. Chem.*, 2017, **129**, 2522–2526.
38. Z. Liu, J. Huo, T. Fu, H. Tan, F. Ye, M. L. Hossain, and J. Wang, *Chem. Commun.*, 2018, **54**, 11419–11422.
39. C. Bo, Q. Bu, X. Li, G. Ma, D. Wei, C. Guo, B. Dai, and N. Liu, *J. Org. Chem.*, 2020, **85**, 4324–4334.
40. B. J. Qu, Y. H. Xu, W. F. Shi, and B. Rånby, *Macromolecules*, 1992, **25**, 5215–5219.
41. D. C. Doetschman, R. C. Mehlenbacher, and D. Cywar, *Macromolecules,* 1996, **29**, 1807–1816.
42. B. Qu, Y. Xu, and W. Shi, *Macromolecules,* 1992, **25**, 5215–5219.
43. S. W. Bennett, C. Eaborn, A. Hudson, H. A. Hussain, and R. A. Jackson, *J. Organometal. Chem.*, 1969, **16,** 36–38.
